# Tick and Host Derived Compounds Detected in the Cement Complex Substance

**DOI:** 10.3390/biom10040555

**Published:** 2020-04-05

**Authors:** Margarita Villar, Iván Pacheco, Octavio Merino, Marinela Contreras, Lourdes Mateos-Hernández, Eduardo Prado, Dina Karen Barros-Picanço, José Francisco Lima-Barbero, Sara Artigas-Jerónimo, Pilar Alberdi, Isabel G. Fernández de Mera, Agustín Estrada-Peña, Alejandro Cabezas-Cruz, José de la Fuente

**Affiliations:** 1 SaBio, Instituto de Investigación en Recursos Cinegéticos (IREC-CSIC-UCLM-JCCM), Ronda de Toledo s/n, 13005 Ciudad Real, Spain; MargaritaM.Villar@uclm.es (M.V.); ivan.pacheco@uclm.es (I.P.); marinela.contreras@uclm.es (M.C.); lourdes.mateos@vet-alfort.fr (L.M.-H.); dinakarenbarros@gmail.com (D.K.B.-P.); josefranvet@gmail.com (J.F.L.-B.); sara.artigas@uclm.es (S.A.-J.); maria.alberdi@uclm.es (P.A.); mariaisabel.garcia@uclm.es (I.G.F.d.M.); 2 Biochemistry Section, Faculty of Science and Chemical Technologies, and Regional Centre for Biomedical Research (CRIB), University of Castilla-La Mancha, 13071 Ciudad Real, Spain; 3 Facultad de Medicina Veterinaria y Zootecnia, Universidad Autónoma de Tamaulipas, Km 5, Carretera Victoria-Mante, CP 87000 Ciudad Victoria, Tamaulipas, Mexico; mero840125@hotmail.com; 4 UMR BIPAR, INRAE, ANSES, Ecole Nationale Vétérinaire d’Alfort, Université Paris-Est, 94700 Maisons-Alfort, France; alejandro.cabezas@vet-alfort.fr; 5 Department of Applied Physics, Faculty of Chemical Sciences and Technologies, Universidad de Castilla-La Mancha, Avda. Camilo José Cela 10, 13071 Ciudad Real, Spain; eduardo.prado@uclm.es; 6 Sabiotec, Camino de Moledores s/n. 13003, 13071 Ciudad Real, Spain; 7 Facultad de Veterinaria, Universidad de Zaragoza, 50013 Zaragoza, Spain; aestrada@unizar.es; 8 Department of Veterinary Pathobiology, Center for Veterinary Health Sciences, Oklahoma State University, Stillwater, OK 74078, USA

**Keywords:** tick, cement, proteomics, dispersive energy X-ray spectroscopy, sialome, cementome, alpha-gal

## Abstract

Ticks are obligate hematophagous arthropods and vectors of pathogens affecting human and animal health worldwide. Cement is a complex protein polymerization substance secreted by ticks with antimicrobial properties and a possible role in host attachment, sealing the feeding lesion, facilitating feeding and pathogen transmission, and protection from host immune and inflammatory responses. The biochemical properties of tick cement during feeding have not been fully characterized. In this study, we characterized the proteome of *Rhipicephalus microplus* salivary glands (sialome) and cement (cementome) together with their physicochemical properties at different adult female parasitic stages. The results showed the combination of tick and host derived proteins and other biomolecules such as α-Gal in cement composition, which varied during the feeding process. We propose that these compounds may synergize in cement formation, solidification and maintenance to facilitate attachment, feeding, interference with host immune response and detachment. These results advanced our knowledge of the complex tick cement composition and suggested that tick and host derived compounds modulate cement properties throughout tick feeding.

## 1. Introduction

Ticks (Arthropoda: Ixodida) are obligate hematophagous arthropods and vectors of pathogens affecting human and animal health as well as animal welfare and production worldwide [1,2,3,4]. The spread of ticks at different geographical scales increases the risk of pathogen transmission to humans and animals since new colonization events may happen in areas with low awareness towards the diseases caused by these pathogens [5].

Ixodid ticks feed on blood for a relatively long period and an evolutionary adaptation to overcome host inflammatory and immune responses using molecules produced in tick salivary glands or recycled from the host and inoculated with saliva into the feeding site [6,7,8,9,10,11,12,13,14,15,16,17,18,19,20,21,22]. Cattle tick *Rhipicephalus microplus* (Canestrini, 1888) is an Ixodid one-host tick species (all developmental stages remain on the same host) with an impact on cattle industry in tropical and subtropical regions of the world [3,9,10]. However, despite the growing impact of ticks on humans and animals worldwide, effective and environmentally friendly control methods such as vaccines among other interventions are still required to control tick infestations and tick-borne diseases [23,24,25,26].

As recently supported by mechanistic studies [27], feeding of ixodid ticks begins with the secretion through inserted mouthparts of cement salivary proteins [28]. Cement is a complex substance secreted by most ticks of the family Ixodidae including *Rhipicephalus* spp. to anchor their mouthparts to the host skin [29]. The cement has not only adhesive properties but has been proposed to have a possible role in antimicrobial properties, seals the lesion during feeding, facilitates feeding and pathogen transmission, and protects ticks from host immune and inflammatory responses [22,29]. Tick cement has been studied using different methodological approaches to characterize its structure, protein and chemical composition, and functional implications such as decrease in the width of cement cone as it goes deeper into the host skin or after Glycine-rich protein coding gene knockdown in *Amblyomma americanum* [16,20,29,30]. However, the biochemical properties of tick cement during different ectoparasite feeding stages have not been fully characterized.

The objective of this study was to characterize the tick and host derived compounds present in the cement throughout tick feeding. To address this objective, in this study we characterized the proteome of tick *R. microplus* salivary glands (sialome) and cement (cementome) together with physical and chemical properties of the cement collected at different adult female parasitic stages. Our experimental approach using proteomics allowed high throughput identification of tick and host derived proteins in both sialome and cementome. Additionally, the first analysis of the chemical elements using scanning electron microscopy (SEM) combined with dispersive energy X-ray spectroscopy (EDS) allowed the characterization of changes in their composition in tick salivary glands and cement during tick feeding, which correlated with changes in protein profiles. The composition of glycan α-Gal was characterized in tick sialome and cementome with putative functional implications (see Section 3.7). These results advanced our knowledge of the complex tick cement composition and suggested that tick and host derived compounds modulate the biochemical properties of the cement throughout tick feeding.

## 2. Materials and Methods

### 2.1. Ticks

The R. microplus (Susceptible Media Joya strain, CENAPA, Mexico) ticks were obtained from a laboratory colony maintained at the University of Tamaulipas (UAT), Mexico [31]. Tick larvae were fed on cross-bred Bos taurus cattle and collected after repletion to allow for oviposition and hatching in humidity chambers at 12 h light:12 h dark photoperiod, 22–25 °C and 95% relative humidity (RH). Larvae were used for infestations at 15 days after hatching from eggs. Female ticks were manually detached at different time points (50 ticks per replicate, n = 2 biological replicates). The study was conducted in accordance with standards specified in the Guide for Care and Use of Laboratory Animals for the University of Tamaulipas (UAT), Mexico. The protocol was approved by the ethics committee of the UAT (No. CBBA_17_0).

### 2.2. Collection of Tick Cement Cones and Salivary Glands

Tick cement cones were carefully collected using soft tissue forceps from mouthparts of manually detached adult female ticks after feeding on cattle for 14–17 (T1), 18–20 (T2), and 21–25 (T3) days post-infestation (dpi) and corresponding to parasitic stages immediately after molting to adults (T1), secondary cement production (T2), and just before tick detachment (T3). Special attention was taken to remove all host-derived skin or hairs attached to the cement. Salivary glands were extracted from the same dissected ticks. Collected cement cones were rinsed with PBS with 1% of protease inhibitor cocktail M221 (VWR Life Science AMRESCO, OH, USA) and kept in PBS/M221 or RNAlater (Sigma-Aldrich, St. Louis, MO, USA). Salivary glands were dissected from female ticks and washed in ice-cold PBS containing the protease inhibitor cocktail to remove hemolymphs-related cells as previously described [14,32] and kept in RNAlater (Sigma-Aldrich) until analysis.

### 2.3. Protein Extraction from Tick Salivary Glands and Cement

Tick salivary glands and cement samples were resuspended in 200 μL 50 mM Tris-HCl pH 7.4 supplemented with 4% sodium dodecyl sulfate (SDS), 1 mM DTT and complete protease inhibitor cocktail (Roche, Basel, Switzerland) using a 1 mL syringe with a 40 mm needle (Terumo Europe España SL, Alcalá de Henares, Spain). Samples were sonicated for 3 min in an ultrasonic cooled bath, followed by vortexing for 10 sec. After five cycles of sonication-vortex, tick salivary glands and cement lysates were centrifuged at 200× *g* for 5 min. Salivary glands were completely solubilized and salivary glands and cement supernatants were collected. Pellet cement samples were homogenized in lysis buffer (8 M urea, 2 M thiourea, and 2% 3-[(3-cholamidopropyl) dimethylammonio]-1-propanesultonate (CHAPS), following the same protocol until cement samples were completely homogenized. Protein concentration in salivary glands and SDS and urea cement extracts was determined using the Bicinchoninic acid (BCA) Protein Assay Kit (Thermo Fisher Scientific, Waltham, MA, USA) following manufacture’s recommendations. Proteins extracts were methanol/chloroform precipitated and stored at −20 °C until analysis.

### 2.4. Proteomics Analysis of Tick Sialome and Cementome

The sialome and cementome of *R. microplus* female ticks collected at different feeding stages were characterized by proteomics analysis using reverse phase liquid chromatography mass spectrometry (RP–LC–MS/MS) using an ekspertTM nanoLC 415 system coupled on line with a 6600 TripleTOF mass spectrometer (Ab Sciex, Framingham, MA, USA) through information-cyclic data independent acquisition (DIA) followed by sequential windowed data independent acquisition of the total high-resolution mass spectra (SWATH)-mass spectrometry (MS). Fifty micrograms of proteins from each sample were on-gel concentrated by SDS–PAGE and trypsin digested as previously described [33]. Resulting peptides were desalted onto OMIX Pipette tips C18 (Agilent Technologies, Santa Clara, CA, USA), dried-down and stored at −20 °C until analysis. The desalted protein digests were resuspended in 2% acetonitrile, 5% acetic acid in water for analysis. Approximately 4 μg of each protein digest of the two biological replicates were pooled together as a mixed sample from tick salivary glands and SDS and urea-extracted cement samples, respectively, collected at three different feeding stages (T1–T3), which were used for the generation of the reference spectral ion library as part of the SWATH-MS analysis. The peptides were concentrated using a 0.1 × 20 mm C18 RP precolumn (Thermo Fisher Scientific), and then separated using a 0.075 × 250 mm C18 RP column (New Objective, Woburn, MA, USA) operating at 0.3 mL/min. Peptides were eluted using a 120 min gradient from 5% to 30% solvent B in solvent A followed by 15 min gradient from 30% to 60% solvent B in solvent A (Solvent A: 0.1% formic acid in water, solvent B: 0.1% formic acid in acetonitrile) and directly injected into the mass spectrometer for analysis. For IDA experiments, the mass spectrometer was set to scanning full spectra (350–1400 *m*/*z*) using 250 ms accumulation time per spectrum, followed by up to 50 MS/MS scans (100–1500 *m*/*z*). Candidate ions with a charge state between +2 and +5 and counts per second above a minimum threshold of 100 were isolated for fragmentation. One MS/MS spectrum was collected for 100 ms, before adding those precursor ions to the exclusion list for 15 sed (mass spectrometer operated by Analyst TF 1.6, Ab Sciex). Dynamic background subtraction was turned off. MS/MS analyses were recorded in high sensitivity mode with rolling collision energy on and a collision energy spread of 5. For SWATH quantitative analysis, fifty-four independent samples (three technical replicates of each biological replicate from salivary glands, SDS cement and urea cement extracts collected at T1, T2, and T3 dpi) (8 μg each) were subjected to the cyclic data independent acquisition (DIA) of mass spectra using the SWATH variable windows calculator (V 1.0, Ab Sciex) and the SWATH acquisition method editor (Ab Sciex), similar to established methods [34]. A set of 50 overlapping windows was constructed (containing 1 *m*/*z* for the window overlap), covering the precursor mass range of 400–1250 *m*/*z*. For these experiments, a 50 ms survey scan (350–1400 *m*/*z*) was acquired at the beginning of each cycle, and SWATH–MS/MS spectra were collected from 100–1500 *m*/*z* for 70 ms at high sensitivity mode, resulting in a cycle time of 3.6 sec. Collision energy for each window was determined according to the calculation for a charge +2 ion-centered upon the window with a collision energy spread of 15.

### 2.5. Library Generation, Protein Identification, Data Processing, and Relative Quantitation

**Figure 1:**
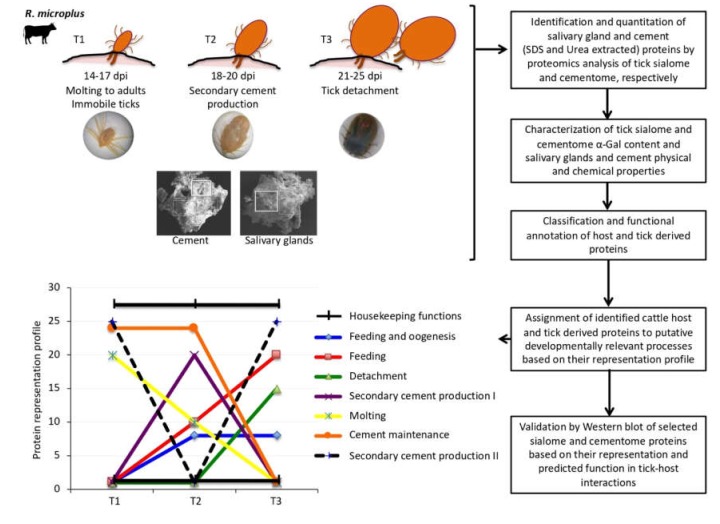
Experimental design. The sialome, cementome, and salivary glands and cement physical and chemical properties were characterized in *R. microplus* adult female ticks during feeding on cattle in samples collected at three time points corresponding to 14–17 dpi (T1; immediately after molting to adults immobile ticks), 18-20 dpi (T2; during secondary cement production), and 21–25 dpi (T3; just prior to tick detachment). After protein extraction, proteomics was used for the identification, quantitation, functional annotation, and assignment to putative developmentally relevant processes based on the representation of sialome and cementome proteins derived from bovine host and tick. Based on representation profiles in arbitrary units, proteins were putatively assigned to different categories of developmental processes including secondary cement production, cement maintenance, feeding, oogenesis, molting, detachment, and housekeeping functions. Cement physical and chemical properties and α-Gal content were also characterized. Finally, selected proteins based on their representation and predicted function in tick-host interactions were analyzed by Western blot.

To create a spectral library of all the detectable peptides in the samples, the IDA MS raw files were combined and subjected to database searches in unison using ProteinPilot software v. 5.0.1 (Ab Sciex) with the Paragon algorithm. Spectra identification was performed by searching against a compiled database containing all the sequences from Uniprot (https://www.uniprot.org) Ixodidae and *Bos taurus* taxa (148,942 and 32,715 entries, respectively, in April, 2019) with the following parameters: iodoacetamide cysteine alkylation, trypsin digestion, gel-based ID as special factor, identification focus on biological modification and thorough ID as search effort. We used the Ixodidae database to improve protein identification for a more comprehensive proteomics analysis because it contains 148,942 entries while for *Rhipicephalus* only 46,429 are available. The detected protein threshold was set at 0.05. An independent False Discovery Rate (FDR) analysis, using the target-decoy approach provided by Protein Pilot (Ab Sciex; https://sciex.com/products/software/proteinpilot-software), was used to assess the quality of identifications. Positive identifications were considered when identified proteins reached a 1% global FDR. For SWATH processing, up to ten peptides with seven transitions per protein were automatically selected by the SWATH Acquisition MicroApp 2.0 in the PeakView 2.2 software (Ab Sciex; https://sciex.com/products/software/peakview-software) with the following parameters: 15 ppm ion library tolerance, 5 min XIC extraction window, 0.01 Da XIC width, and considering only peptides with at least 99% confidence and excluding those which were shared or contained modifications. However, to ensure reliable quantitation, only proteins that had 3 or more peptides available for quantitation were selected for XIC peak area extraction and exported for analysis in the MarkerView 1.3 software (Ab Sciex; https://sciex.com/products/software/markerview-software). Global normalization was performed according to the Total Area Sums (TAS) of all detected proteins in the samples. The Student’s t-test (*p* < 0.05) was used to perform two-sample comparisons between the averaged TASs of all the transitions derived from each protein across the three replicate runs for each sample under comparison in order to identify proteins that were significantly differentially represented between time points (T1–T3). Gene ontology (GO) annotations were obtained using Blast2GO software (http://www.blast2go.org). Host and tick derived proteins identified in the sialome and cementome differentially represented in at least one comparison between the different time points (T1 vs. T2, T2 vs. T3, and T1 vs. T3) were grouped based on the assignment to putative categories of developmentally relevant processes according to their representation profile. Based on representation profiles, proteins were putatively assigned to different categories of developmental processes including secondary cement production I and II, cement maintenance, feeding, feeding and oogenesis, molting, and detachment. Proteins without significant differences in representation at different time points were assigned to housekeeping functions (Figure 1). The assignments to developmental processes were used for protein profile classification and some proteins may be implicated in different tissue-specific processes. Raw proteomics data were deposited at the PeptideAtlas repository (http://www.peptideatlas.org/) with the dataset identifier PASS01524. Results are included in Data S1–S4 and Tables S1 and S2.

### 2.6. Tick salivary Glands and Cement Physical and Chemical Properties

The amino acid composition, theoretical isoelectric point (pI), atomic composition, instability index. and grand average of hydropathicity (GRAVY) of the peptides used to identify all host proteins identified in the cementome but not in the sialome (host), 100 randomly selected tick proteins identified in the sialome but not in the cementome (tick), all host proteins identified in the sialome and cementome (host cement), and all tick proteins identified in the cementome (tick cement) (Data S4) were analyzed using the ProtParam tool (https://web.expasy.org/protparam) [35]. Results are included in Table S3. Individual tick cement and salivary gland samples collected at the same time points (T1–T3) were dehydrated in an incubator at 37 °C for 24 h in preparation for analysis with SEM. The samples were placed and mounted on standard aluminum SEM sample holders with conductive carbon adhesive tabs. The samples were observed and analyzed with a field emission scanning electron microscope (Zeiss GeminiSEM 500, Oberkochen, Germany) operating in high vacuum mode at an acceleration voltage of 15 kV and without metal coating. For the analysis of chemical elements, 3 area scans per sample were included using an 80 mm^2^ EDS detector (Oxford Instruments, Abingdon, United Kingdom). Results are included in Data S5 and S6. The composition of chemical elements in the cement and salivary glands of *R. microplus* were compared at different time points by One-way ANOVA test (https://www.socscistatistics.com/tests/anova/default2.aspx; *p* < 0.05, *n* = 2–4 biological replicates). The correlation analysis between total protein atomic composition in tick-derived proteins identified in the sialome but not in the cementome (Tick), all tick-derived proteins identified in the cementome (Tick cement) and all host-derived proteins identified in the sialome and cementome (Host cement) (Table S3) and T1–T3 average chemical atomic percentage of relative abundance in tick salivary glands and cement (Data S6) was performed using the elements identified in both sialome/cementome and tick salivary glands/cement chemical composition (C, N, O, and S) with Spearman’s Rho test calculator (https://www.socscistatistics.com/tests/spearman/default2.aspx).

### 2.7. Tick Sialome and Cementome α-Gal Content

The Galα1-3Galβ1-(3)4GlcNAc-R (α-Gal; Figure S1) content was analyzed in tick sialome and cementome at different time points (T1–T3). An ELISA test was used to determine the α-Gal levels in protein extracts. Plates were coated with 100 ng of protein per well in carbonate/bicarbonate buffer and incubated overnight at 4 °C and blocked with 1% human serum albumin (Sigma-Aldrich) in PBS for 1 h at room temperature (RT), following three washes with PBS containing 0.05% Tween 20 (PBST). The anti-α-Gal epitope monoclonal antibody (M86; Farmingdale, NY, USA) was added at 1:50 dilution in PBS and incubated for 1 h at 37 °C followed by three washes with PBST. Then, goat anti-mouse IgM (μ-chain specific) peroxidase-conjugated antibodies (Sigma-Aldrich) were added at 1:20000 dilution in PBS. The reaction was visualized by adding 100 μL of 3,3’,5,5´- Tetramethylbenzidine (Promega, Madison, WI, USA) and incubated for 20 min in the dark at RT. The optical density (OD) was measured at 450 nm with a Multiskan FC ELISA reader (Thermo Fisher Scientific). The average value of the blanks (wells without tick protein coating; n = 3) was subtracted from all reads and the average of 3 replicates for each sample was used for further analysis. Protein extracts from human promyelocytic leukemia HL60 cells (ATCC CCL-240) and pork (*Sus scrofa*) kidney were included as negative and positive controls, respectively. A calibration curve with 0.0 to 1.0 ng bovine serum albumin (BSA)-α-Gal (Dextra, Shinfield, UK) and O.D. values at 450 nm was constructed using Microsoft Excel for Mac (v. 16.26) to convert ELISA reader values to α-Gal content per sample (*R^2^* = 0.913; Figure S2). The results (average + S.D. of α-Gal/1 μg protein) were compared between pork kidney positive control, salivary gland or cement samples and HL60 cells negative control and between salivary gland and cement samples at different time points (T1–T3) by Student’s t-test with unequal variance (*p* < 0.05, *n* = 3 biological replicates). Variations in α-Gal levels at different time points (T1–T3) in both sialome and cementome were compared by one-way ANOVA test (https://www.socscistatistics.com/tests/anova/default2.aspx) (*p* < 0.05, *n* = 3 biological replicates).

### 2.8. Network Analysis of Tick Cementome

A network of interactions was used for the integration of data obtained from *R. microplus* proteins identified by proteomics in the cementome at different feeding stages. The methodology to build the network of interactions has been previously described and validated [36]. This network consists of a set of nodes that are connected by edges where nodes are the interacting items, and links between nodes represent the strength with which they interact. In this development, we used the Sparse Correlation for Compositional Data (SparCC) to obtain the strength of association between proteins [37], which infers associations in compositional data by estimating the linear Pearson’s correlations between the log-transformed components. Correlation values from SparCC between co-occurring proteins were used to populate the weights of the links among nodes and build the network. Centrality is a fundamental property of the network because it refers to nodes that connect high score nodes [36,38]. In this context, “high score” applies to other nodes with high importance in the network. We calculated the importance of a node in the “traffic” between different nodes of the network using Betweenness Centrality (BNC), giving a higher score to a node that sits on many shortest paths of other node pairs [36,39]. In our context, this score is an indicator of the relative importance of a protein co-occurring with two or more proteins.

### 2.9. Recombinant Proteins and Antibodies

Recombinant tick histone H4 (Q4PM69), aminopeptidase N (A0A131YLU7) and Glycine-rich superfamily member (A0A224YEQ4) proteins (Table S4) were produced in *Escherichia coli*. Genes coding for histone H4 and aminopeptidase N were cloned by RT-PCR using *R. microplus* RNA and gene-specific primers (Table S4). The Glycine-rich superfamily member was amplified from a synthetic gene optimized for codon usage in *E. coli* (Genscript Corporation, Piscataway, NJ, USA) (Table S4). For the production of the membrane-bound histone H4-*Anaplasma marginale* major surface protein 1a (MSP1a) chimera, recombinant *E. coli* BL21 cells transformed with the pMBXAF3 expression vector were used [40]. In this construct, as for others MSP1a chimeras [41], the inserted histone H4 coding region is fused to MSP1a and is under the control of the inducible tac promoter [40]. The amplified DNA fragments from aminopeptidase N and Glycine-rich superfamily member protein coding regions were cloned into the expression vector pET101 and all the proteins were produced in the *E. coli* strain BL21 using the Champion pET101 Directional TOPO Expression kit (Carlsbad, CA, USA). Recombinant histone H4-MSP1a was purified as previously reported [42]. Briefly, recombinant proteins were fused to Histidine tags for purification by affinity to Ni [43,44]. Transformed *E. coli* strains were induced with IPTG for 4.5 h to produce recombinant proteins, which were purified to >85% of total cell proteins by Ni affinity chromatography as previously described [43,44] using 1 mL HisTrap FF columns mounted on an AKTA-FPLC system (GE Healthcare, Piscataway, NJ, USA) in the presence of 7 M urea lysis buffer. The purified antigens were refolded by dialysis against 1,000 volumes of PBS (137 mM NaCl, 2.7 mM KCl, 10 mM Na_2_HPO_4_, 1.8 mM KH_2_PO_4_, pH 7.4) for 12 h at 4 °C and stored at −20 °C until used.

For histone H4-MSP1a and aminopeptidase N recombinant proteins, two New Zealand white rabbits (*Oryctulagus cuniculus*) were subcutaneously injected at weeks 0, 4, and 6 with 50 μg protein in 0.4 mL Montanide ISA 50 V adjuvant (Seppic, Paris, France). Blood was collected before injection and 2 weeks after the last immunization to prepare preimmune and immune sera, respectively. Serum aliquots were kept at 4 °C for immediate use or at −20 °C for long-term storage. The IgG were purified from serum samples using the Montage antibody purification kit and spin columns with PROSEP-A media (Millipore, Billerica, MA, USA) following the manufacturer’s recommendations. Commercial antibodies against host pan-keratins (ab190625), desmoplakin (ab106342) and alpha-2-HS-glycoprotein (AHSG; ab112528) proteins (Abcam, Cambridge, UK) were used for analysis. In a previously described validation approach [45], these antibodies were used to detect host-derived proteins in fed ticks (Figure S3).

### 2.10. Western Blot or Dot Blot Analysis of Recombinant Proteins and the Tick Sialome and Cementome

#### 2.10.1. Recombinant Proteins (Histone H4-MSP1a, Aminopeptidase N, and Glycine-Rich Superfamily Member Protein; Figure S4)

For Western blot analysis, 10 μg of purified recombinant proteins were separated by electrophoresis on a sodium dodecyl sulfate (SDS)-12% polyacrylamide gel (Life Science, Hercules, CA, USA) and either stained with Coomassie Brilliant Blue or transferred to a nitrocellulose membrane. The membrane was blocked with 5% BSA (Sigma-Aldrich) for 2 h at RT, washed four times with Tris-buffered saline (TBS; 50 mM Tris-Cl, pH 7.5, 150 mM NaCl, 0.5% Tween 20). Pooled sera collected from histone H4, Aminopeptidase N vaccinated rabbits and polyclonal rabbit anti-Glycine-rich superfamily member protein antibodies (Abcam) were used as primary antibodies. Primary antibodies were used at a 1:200 dilution in TBS, and the membrane was incubated overnight at 4 °C and washed four times with TBS. The membrane was then incubated with an anti-rabbit IgG-horseradish peroxidase (HRP) conjugate (Sigma-Aldrich) diluted 1:1000 in TBS with 3% BSA (BSA/TBS). The membrane was washed five times with TBS and finally developed with TMB (3,3´, 5,5´-tetramethylbenzidine) stabilized substrate for HRP (Promega, Madrid, Spain) according to the manufacturer recommendations.

#### 2.10.2. Tick Cementome and Sialome

Cement protein lysate (10 µg) and salivary gland protein lysate (20 µg) were methanol/chloroform precipitated, resuspended in Laemmli sample buffer and separated on an SDS-polyacrylamide precast gel (ClearPage Expedeon, VWR, Radnor, PA, USA). Due to the limited amount of cementome and sialome proteins, Western blot analysis was performed for aminopeptidase N only to avoid the limitations posed by the different representation profiles of protein families such as Histones and Glycine-rich superfamily member proteins (Figure S5). After electrophoresis, proteins were transferred to a nitrocellulose blotting membrane (GE Healthcare Dharmacon Inc., Lafayette, CO, USA), blocked with 3% BSA (Sigma-Aldrich) in TBS (3% BSA/TBS) and incubated overnight at 4 °C with antibodies against tick recombinant aminopeptidase N diluted 1:200 in 3% BSA/TBS. To detect the IgG antibodies bound to tick proteins, membranes were incubated with goat anti-rabbit IgG peroxidase antibody (Sigma-Aldrich) diluted 1:1000 in 3% BSA/TBS. Immunoreactive proteins were visualized by chemiluminescence with Pierce ECL Western Blotting Substrate (Thermo Fisher Scientific). Quantitative analysis was performed using the Fiji ImageJ (https://imagej.nih.gov/ij/download.html) to measure the intensity of the protein bands and after substation of the intensity of the PBS control to compare the results at different time points. For the dot blot analysis of the other selected proteins (Figures S6–S8), cement and salivary gland proteins were applied onto each strip in 6 different dots (volume per dot, 25 μL at 5 μg/μL. Then the dots were allowed to dry by gravity flow, immersed in 50 μL of 3% BSA/TBS for 15 min approximately and filtered by gravity. Membranes were washed two times with TBS and incubated with primary antibodies diluted at different concentrations in 1% BSA/TBS against Histone H4 (1:100), Glycine-rich superfamily member proteins (1:200) and host pan-keratins (1:300), desmoplakin (1:300) and alpha-2-HS-glycoprotein (1:300). To detect the IgG antibodies bound to tick proteins, membranes were incubated with goat anti-rabbit IgG peroxidase antibody (Sigma-Aldrich) diluted 1:1000 in 1% BSA/TBS. Membranes were washed two times with TBS and immunoreactive proteins were visualized with chemiluminescence with Pierce ECL Western blotting substrate (Thermo Fisher Scientific). Quantitative analysis was performed as described above for Western blot analysis.

## 3. Results and Discussion

### 3.1. Experimental Design and Rationale

**Figure 2:**
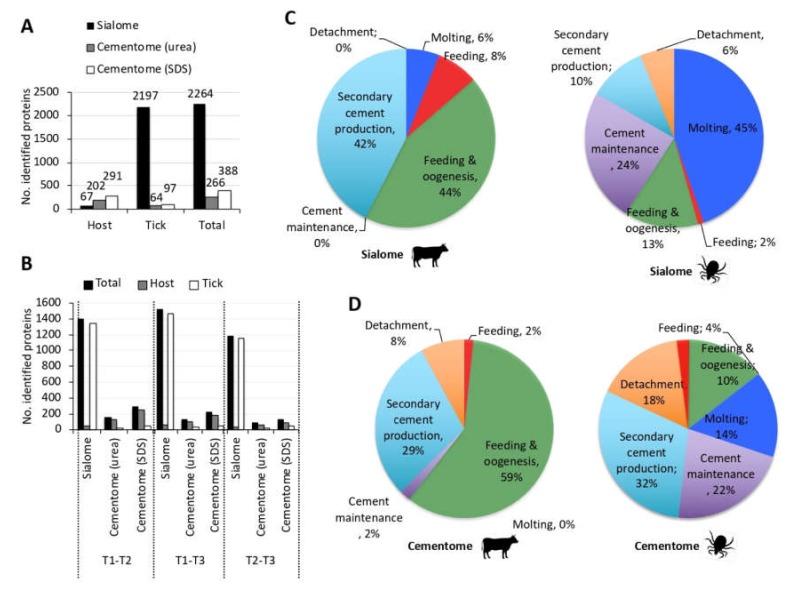
Protein representation profiles in tick sialome and cementome. (**A**) Number of host, tick, and total proteins identified in the sialome and cementome (proteins extracted with urea and sodium dodecyl sulfate (SDS)). (**B**) Host, tick, and total number of differentially represented proteins (*p* < 0.05; Data S4) identified in the sialome and cementome (proteins extracted with urea and SDS). (**C**) Assignment of host and tick derived differentially represented proteins identified in the sialome to putative categories of developmentally relevant processes based on their representation profile. (**D**) Assignment of host and tick derived differentially represented proteins identified in the cementome to putative categories of developmentally relevant processes based on their representation profile. Proteins in the cementome extracted with urea and SDS were grouped together with unique non-redundant entries (Data S4).

*Rhipicephalus microplus* are one-host ticks that complete parasitic stages of their life cycle while feeding on the same host and with an impact on cattle industry in tropical and subtropical regions of the world [3,46]. The *R. microplus* were selected for analysis because little information is available on cement composition in this tick species. The experimental design (Figure 1) included the characterization of sialome, cementome, and salivary gland and cement physical and chemical properties in *R. microplus* adult female ticks during feeding on cattle in samples collected at three time points corresponding to 14–17 dpi (T1; immediately after molting to adults immobile ticks), 18–20 dpi (T2; during secondary cement production), and 21–25 dpi (T3; just prior to tick detachment). *R. microplus* one-host ticks remain attached to the host throughout all parasitic stages with a continuously functioning feeding apparatus during molting [47]. Sampling times were selected based on the well-known life cycle in cattle of the *R. microplus* susceptible Media Joya strain laboratory colony maintained at the UAT, Mexico [31]. The SDS-based protein extraction is commonly used for proteomics analysis of tick samples such as salivary glands, saliva, and cultured cells [12,32,33]. However, probably due to cement composition with previously unknown components, it was not fully dissolved in SDS-containing buffer. Therefore, to improve protein extraction for cementome analysis, a urea-containing buffer previously applied to tick cement analysis [16,30] was used in addition to SDS. This approach resulted in a higher protein identification in the tick cementome (654 proteins) when compared to SDS-based (388 proteins) or urea-based (266 proteins) extraction protocols alone (Figure 2A). After protein extraction, proteomics was used for the identification, quantitation, functional annotation, and assignment to putative developmentally relevant processes based on novel methodology based on the representation profile of sialome and cementome proteins derived from bovine host and tick. Based on representation profiles after significant differences in pairwise comparisons between time points, proteins were putatively assigned to different categories of developmental processes including secondary cement production, cement maintenance, feeding, oogenesis, molting, detachment, and housekeeping functions. Salivary glands, cement physical and chemical properties, and α-Gal content were also characterized. The selection of proteins for analysis was focused on the cementome because little information is available for this complex substance playing a key role during tick parasitic stages [16,29,30,48]. Finally, selected proteins based on their representation and predicted function in tick-host interactions were analyzed by Western blot to further validate proteomics results.

### 3.2. The Representation of R. Microplus Tick and Cattle Host Derived Proteins in the Sialome and Cementome Change during Adult Female Parasitic Stages

Proteins have been identified as the main components of the tick cement [49]. Our experimental approach using proteomics allowed the identification of tick and host derived proteins in the sialome and cementome (2264 and 654 total number of proteins identified in the sialome and cementome, respectively; Figure 2A) (Data S1–S4). These results expand the repertoire of identified cementome tick and host derived proteins, which were previously limited to less than 200 proteins [16,30]. Cattle host-derived proteins were identified in the tick sialome and cementome with higher representation in the later (Figure 2A). As expected, tick-derived proteins were also identified in the sialome and cementome but with over 15-fold representation in the sialome (Figure 2A). Similar results were obtained when only differentially represented proteins (*p* < 0.05 at least between two time points; Data S4) were considered (Figure 2B).

Host and tick derived differentially represented proteins identified in the sialome and cementome were then grouped based on the assignment to putative categories of developmentally relevant processes according to their representation profile (Figure 1, 2C,D). The sialome host-derived proteins were mostly represented (86%) in feeding and oogenesis and secondary cement production, while 69% of tick-derived proteins were represented in the molting and cement maintenance representation profiles (Figure 2C). Additionally, cement maintenance and detachment profiles appeared only in the tick-derived sialome. In the cementome, host-derived proteins were as in the sialome mostly represented (88%) in feeding and oogenesis and secondary cement production (Figure 2D). However, tick-derived cementome profile was different from the sialome with higher representation of secondary cement production and cement maintenance (Figure 2D).

**Figure 3:**
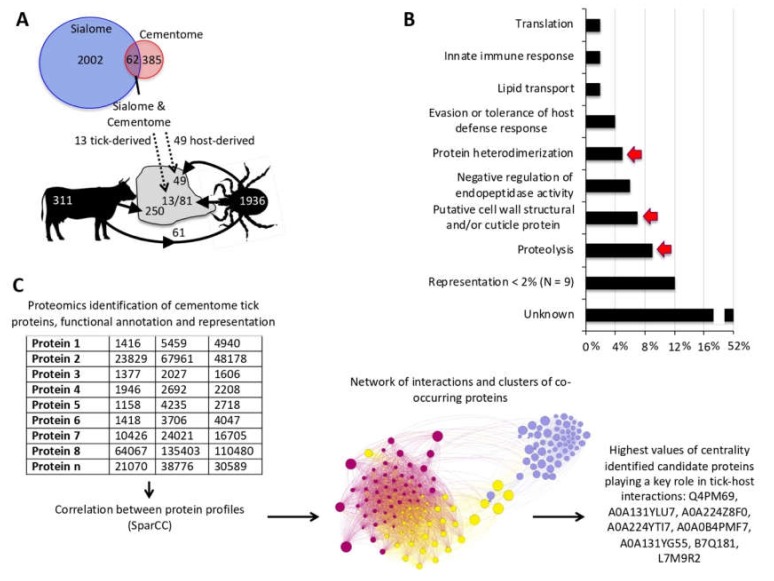
Composition of the tick cementome. (**A**) Of the 62 proteins found in both sialome and cementome, 13 and 49 were tick and host derived proteins, respectively. An additional 68 tick derived proteins present in the cement were not identified in the sialome, resulting in a total of 81 tick-derived proteins in the cementome composition. Other host derived proteins in the cementome were likely contaminants from host cells attached to the tick cement cone. (**B**) Biological processes annotated in tick-derived cementome proteins. Processes containing proteins selected for further analysis are shown with arrows. (**C**) Network analysis of tick-derived cementome proteins. Using co-occurring proteins at different feeding stages, a network was built using correlation values from SparCC. Network centrality was then used to identify the most central proteins in the dataset putatively playing a key role in cement biological processes affecting tick-host interactions.

These results also provided insight into the origin of host and tick derived proteins identified in the sialome and cementome (Figure 3A). Of the 62 proteins found in both sialome and cementome, 13 and 49 were tick and host derived proteins, respectively. In the sialome, 61 host proteins were present of which 49 were secreted into the cement. An additional 250 host-derived proteins were identified in the cement, most of which were likely contaminants from host cells attached to the tick cement cone. Of the 1936 tick proteins identified in the sialome, only 13 appeared as secreted into the cement. However, an additional 68 tick-derived proteins present in the cementome were not identified in the sialome, probably due to the low representation of these proteins in the sialome when compared to the cementome.

Based on their representation profile (Figure 1), proteins present in the sialome and cementome were assigned to secondary cement production, maintenance and/or decomposition for tick detachment, but may also be implicated in tick feeding, molting and/or oogenesis. Proteins in the sialome but not in the cementome may function in different processes such as feeding and oogenesis, and in metabolic pathways involved in the synthesis of metabolite cement components. Proteins in the cementome and/or sialome with similar representation throughout tick feeding and therefore without significant differences in their representation at different time points (Data S1–S4) may be involved in housekeeping functions as well as in other processes. Despite significant differences in protein representation and profiles, some of the tick proteins identified in the cementome may constitute secreted proteins not incorporated into the cement.

### 3.3. Tick-Derived Cementome Proteins Appear to be Involved in Cement Formation, Solidification, Maintenance, Feeding, and Interference with Host Immune Response and Detachment

Tick-derived cementome proteins were selected for consideration in further analyses based on one of the following criteria (Table S1): (a) identified in both sialome and cementome; (b) being among the two proteins with highest representation in feeding, feeding and oogenesis, molting, secondary cement production, cement maintenance or detachment profile categories; and (c) predicted to play a key role in cementome biological processes affecting tick-host interactions due to over 4% representation in biological processes (Figure 3B) or after network analysis (Figure 3C). Network analysis of tick-derived cementome proteins was conducted using interactions and clusters of co-occurring proteins at different feeding stages and correlation values from SparCC. Network centrality index was then used to identify in the dataset the proteins with highest values of centrality and consequently putatively playing a key role in cement biological processes affecting tick-host interactions Figure 3C).

**Figure 4:**
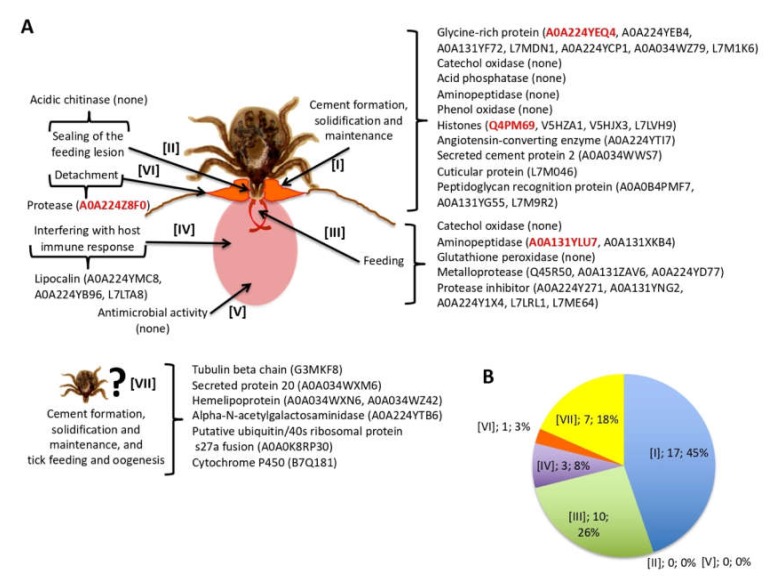
Tick-derived cementome proteins. (**A**) Selected tick-derived cementome proteins (Table S1) were found in most of the processes previously proposed to be present in the tick cementome. Proteins selected for further analyses are highlighted in red. (**B**) Protein representation (process, number of proteins, percentage) in the different processes previously proposed to be present in the tick cementome.

Tick proteins fulfilling these criteria were found in most of the processes previously proposed to be present in the cementome [29] (Figure 4A). Most proteins appeared putatively involved in cement formation, solidification, and maintenance (45%), and tick feeding (26%) (Figure 4B). Other identified proteins had putative functions in detachment and interference with host immune response (Figure 4B). However, acidic chitinase proteins proposed to function in sealing of the feeding lesion [48] or proteins with antimicrobial activity were not identified while 18% of the proteins had an unknown function with possible implication in cement formation, solidification and maintenance, and tick feeding and oogenesis (Figure 4B). The protein representation profiles of selected tick cementome proteins were mainly assigned to secondary cement production, maintenance, and detachment (Table S1).

### 3.4. Host-Derived Cementome Proteins Co-Exist with Tick-Derived Proteins and May Contribute to Cement Structure and Function

**Figure 5:**
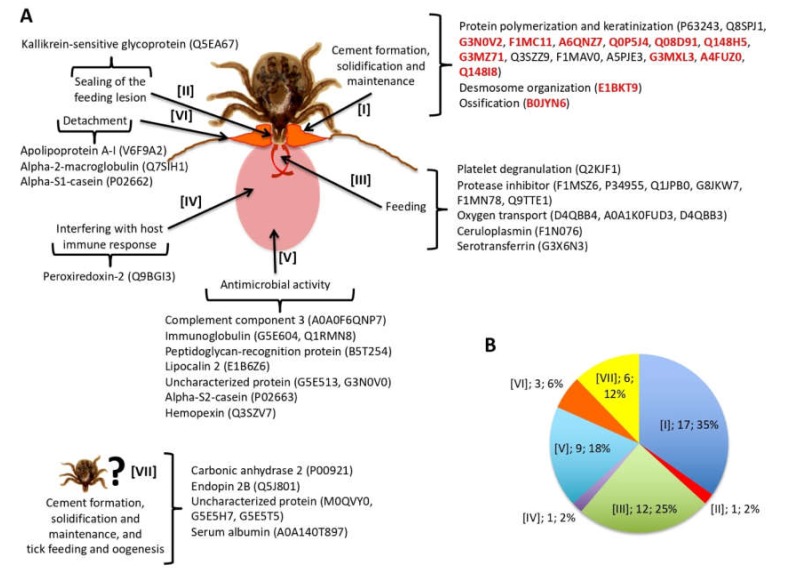
Host-derived cementome proteins. (**A**) Host proteins identified in both sialome and cementome (Table S2) were found in all of the processes previously proposed to be present in the tick cementome. Proteins selected for further analyses are highlighted in red. (**B**) Protein representation (process, number of proteins, and percentage) in the different processes previously proposed to be present in the tick cementome.

As with tick-derived proteins, the analysis was focused on bovine host-derived proteins identified in the cementome. In particular and to reduce the possible contamination with host-derived proteins, the analysis was focused on the 49 host proteins identified in the sialome and secreted into the cementome (Figure 3A, Table S2). Of these 49 proteins, 10 corresponded to keratins of which two were represented in the cementome with more than 1 million TAS at all time points (Table S2). Based on the processes previously proposed to be present in the cementome [29], most host-derived proteins appeared as putatively involved in cement formation, solidification and maintenance (35%), and tick feeding (25%) (Figure 5A,B). Other identified proteins had putative functions in detachment and interference with host immune response (Figure 5B). These results were similar to those found with tick-derived proteins (Figure 4B). However, proteins putatively involved in sealing of the feeding lesion and with antimicrobial activity that were not found in tick-derived proteins (Figure 4B) were present among host-derived proteins (Figure 5B). Additionally, 12% of the host-derived proteins were identified with possible implication in cement formation, solidification and maintenance, and tick feeding and oogenesis (Figure 5B).

These results suggested that in the cementome, host-derived proteins might be involved in the cement structure and function, possibly complementing and synergizing with tick-derived proteins. The cement protein representation profiles of selected host-derived cementome proteins were mainly assigned to feeding and oogenesis, secondary cement production, maintenance, and detachment (Table S2).

### 3.5. Biological Processes Represented in the Tick and Host Cementome Support Proposed Complementation and Synergy in Cement Formation and Function between Tick and Host Derived Proteins

**Figure 6:**
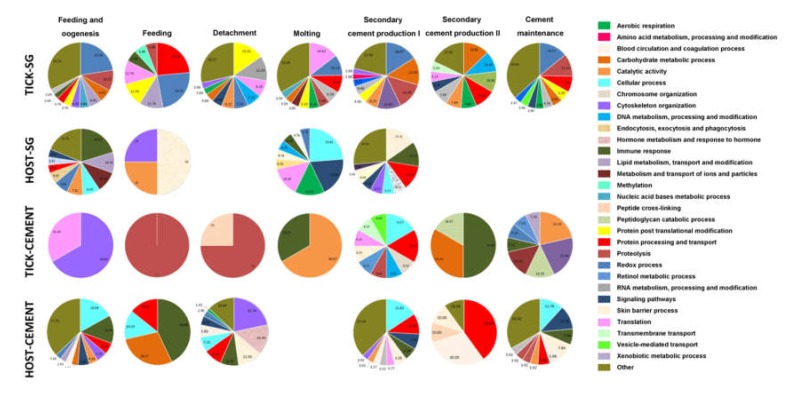
Classification according to biological processes (P) of the host and tick derived proteins identified in the sialome (HOST-SG and TICK-SG) and cementome (HOST-CEMENT and TICK-CEMENT) and putatively assigned to the different categories of developmental processes including secondary cement production I and II, cement maintenance, feeding, feeding and oogenesis, molting, and detachment. All P were considered, but only the ten most abundant (percentage of representation within the total P per sample) are displayed in the figure. The GO P categories were grouped in different P and less abundant categories were grouped as Other (Data S4). A high resolution image was include in Data S4.

To further characterize the differences between tick and host sialome and cementome, a summary of the biological processes (P) affected by these proteins was included within the experimental approach used here for the different developmentally relevant processes (Figure 6 and Data S4). The results showed differences in the P affected by tick and host derived proteins, further supporting the possibility of complementation and synergy between these proteins in cement formation and function. In the cementome, the most represented biological processes (P) (>30%) were translation, cytoskeleton organization, proteolysis, immune response, catalytic activity, carbohydrate metabolism, protein processing and transport, and blood circulation and coagulation (Figure 6). As it has been proposed for cement properties and function [22,29], the cementome P support the presence of antimicrobial properties, feeding and metabolism, and protection from host immune and inflammatory responses.

### 3.6. Physical and Chemical Properties of Tick Salivary Glands and Cement Show Tissue-Specific Differences and Vary during Tick Feeding

**Figure 7:**
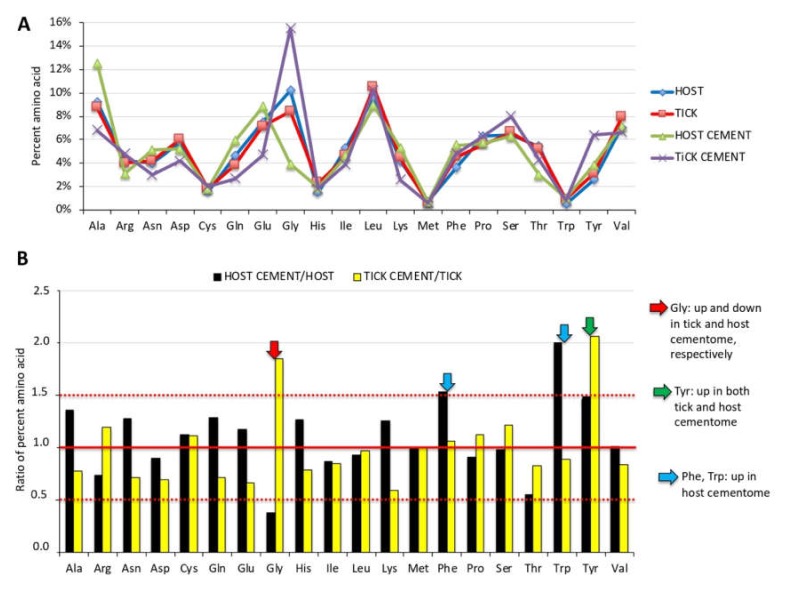
Amino acid composition of tick cementome. (**A**) Amino acid composition of all host proteins identified in the cementome but not in the sialome (HOST), 100 randomly selected tick proteins identified in the sialome but not in the cementome (TICK), all host proteins identified in the sialome and cementome (HOST CEMENT), and all tick proteins identified in the cementome (TICK CEMENT) (Data S6) were analyzed using the ProtParam tool. (**B**) Amino acids with highest changes (0.5 ≥ ratio of percent amino acid ≥ 1.5) in the cementome composition when compared to non-cementome host-derived (host cement/host) or tick-derived (tick cement/tick) proteins.

**Figure 8:**
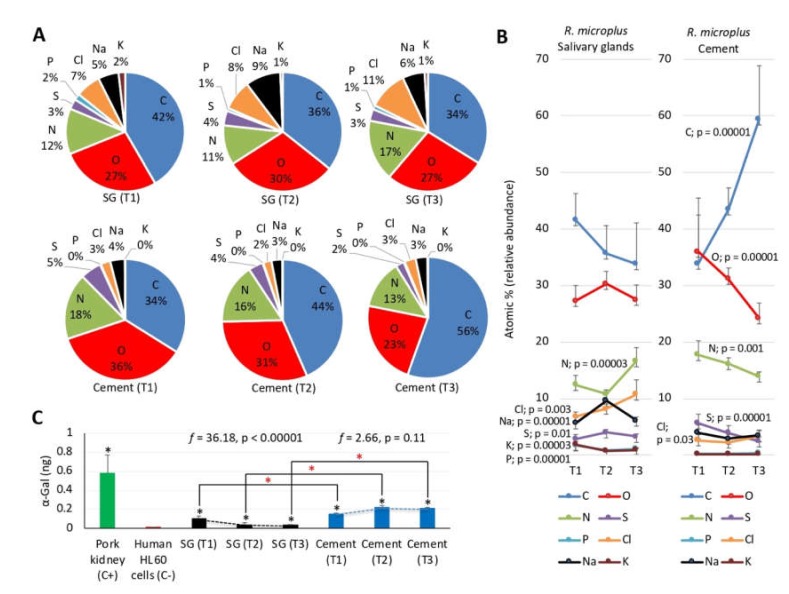
Composition of chemical elements in tick salivary glands and cement. Chemical elements were characterized by SEM combined with EDS analysis in samples from tick salivary glands (SG) and cement. (**A**) Representation (%) of the chemical elements at different feeding stages (T1–T3). (**B**) Changes in the representation (%) of the chemical elements at different feeding stages (T1–T3). The composition of chemical elements was compared at different time points by One-way ANOVA test (https://www.socscistatistics.com/tests/anova/default2.aspx; *p* < 0.05, *n* = 2–4 biological replicates). (**C**) Characterization of α-Gal content in tick SG and cement protein extracts and in comparison with human promyelocytic leukemia HL60 cells (ATCC CCL-240) and pork (*Sus scrofa*) kidney as negative and positive controls, respectively. A calibration curve with 0.0–1.0 ng α-Gal and O.D. values at 450 nm was constructed using Microsoft Excel for Mac (v. 16.26) to convert ELISA reader values to α-Gal content per sample (*R^2^* = 0.913). The results (average + S.D. of α-Gal/1 μg protein) were compared between pork kidney positive control, salivary gland or cement samples and HL60 cells negative control (black * *p* < 0.05, *n* = 3 biological replicates) and between salivary gland and cement samples at different time points (T1–T3) (red * *p* < 0.05, *n* = 3 biological replicates) by Student’s t-test with unequal variance. Variations in α-Gal levels at different time points (T1–T3) in both sialome and cementome were compared by one-way ANOVA test (https://www.socscistatistics.com/tests/anova/default2.aspx) (*f* and *p* values are shown, *n* = 3 biological replicates).

The *R. microplus* cement physical and chemical properties were analyzed (Figure 7 and Figure 8, Table S3, Data S5 and S6). The analysis of amino acid composition of tick cementome showed that Gly was the amino acid with highest representation in the tick-derived proteins and lowest representation in the host-derived proteins (Figure 7A,B). This finding correlated with previous reports [16,29,30] and the high representation of Glycine-rich superfamily member proteins (three proteins with more than three million TAS) found here in the tick cementome, which are putatively involved in cement formation, solidification, and maintenance (Figure 4A,B, Table S1, Data S4). Additionally, Tyr representation was augmented in both host and tick derived proteins in the cementome when compared to non-cementome host and tick derived proteins, respectively (Figure 7B). Other amino acids such as Phe and Trp were represented at higher levels in host-derived cementome than in non-cementome proteins (Figure 7B).

The amino acid profile of dragline silk in *Argiope trifasciata* has a high Gly content (approximately 40%), which correlates with the Gly content (36.5%–43.9%) of the two major components of the spider dragline silk, MaSp1 and MaSp2 proteins [50]. A family of low-molecular weight Cysteine-rich proteins have been also characterized as involved in dragline silk formation [51]. However, Cys was an amino acid with a relative low representation (1.8%–2.0%) in tick cement (Table S3).

The analysis of peptides used to identify host and tick derived proteins (Data S7) evidenced some distinctive characteristics of the cementome tick-derived proteins (Table S3). These characteristics included a higher pI suggesting a more basic protein composition, lower aliphatic index associated with lower thermostability of globular proteins [52], and a lower GRAVY index suggesting the presence of less hydrophilic proteins [53].

The SEM combined with EDS was used for the analysis of chemical elements in *R. microplus* salivary glands and cement cones (Figure 8A,B, Data S5 and S6). The results showed the presence with high relative abundance (>10 atomic %) of C, O, and N in all samples and feeding stages (T1–T3) (Figure 8A). Other elements such as S, P, Cl, Na, and K were present with low relative abundance (<1 atomic %) in most samples and feeding stages (Figure 8A). These results were similar to those recently reported in tick exoskeleton using a similar experimental approach [45]. The analysis of the composition of chemical elements in the cement and salivary glands of *R. microplus* at different time points showed significant variations in both samples but with differences in the chemical elements and profiles (Figure 8B). While C and O relative abundance did not change in the salivary glands, it increased and decreased, respectively in the cement. For N and S, relative abundance increased at T2 or T3 in the salivary glands but decreased in the cement. However, Cl relative abundance increased at T3 in both salivary glands and cement. Other elements such as Na, K, and P showed significant variations in the salivary glands only. Elements Br and Zn were only rarely found in some samples and were not included in the analysis (Data S6). As expected considering that proteins are the major source of certain chemical elements in both tick salivary glands and cement, a positive correlation (*r_s_* = 1, *p* = 0) was obtained between the relative abundance of C, N, O, and S in the salivary glands and cement and the atomic composition of sialome tick-derived proteins and cementome tick and host derived proteins, respectively (Table S3). Furthermore, the profile of the highly represented Gly (C_2_H_5_NO_2_)-rich superfamily member proteins in the cementome that have been implicated in tick response to oxidative stress [20] showed a decrease in protein representation during tick feeding (e.g., A0A224YEQ4; Data S4), which correlated with changes in the composition of O and N chemical elements (Figure 8B). Glycine-rich proteins are highly represented in the sialome of different tick species and were also shown to decrease in abundance during feeding of adult female *A. americanum* [54]. Changes in the composition of cement chemical elements during tick feeding may be also related to physiological mechanisms such as the increase in the intensity of respiratory patterns in *R. sanguineus* ticks during feeding [55]. Differences in the diversity and representation of chemical elements in *R. microplus* uninfected fed female ticks between previously reported results in the exoskeleton (C, O, and N elements with >5 atomic % and *n* = 10 elements with >0.2 atomic % in at least two samples) [45] and the results reported here in the cement cone (C, O, N, and S elements with >5 atomic % and *n* = 7 elements with >0.2 atomic % in at least two samples; Data S6) using the same methodological approach (SEM–EDS) suggested that these two complex substances are formed by different proteins and/or other components.

Previous analyses of chemical composition identified the presence of N in canine *fascia lata* and hair fiber [56,57], thus suggesting that this tick cement element may have a host origin [58]. Trace elements such as Se, Cu, Mn, Zn, and Co present in mammalian cover hair [59] were not identified in the tick cement, thus suggesting a low contribution of the host to tick cement chemical composition. In addition to tick and host derived proteins, chemical composition may be affected by cofactors such as nicotinamide adenine dinucleotide phosphate (NADPH; C_21_H_26_N_7_O_17_P_3_). Reduced NAPDH is an essential electron donor that provides the reducing power regulating multiple anabolic reactions, including those responsible for the biosynthesis of all major cell components in all organisms including ticks [60,61,62]. Increased levels of NADPH in ticks have been associated with response to *Anaplasma phagocytophilum* pathogen infection and tolerance to oxidative stress [61,62]. The source of other chemical elements such as Na, K, Cl, and P, which may contribute to cement biomineralization and coating [29,63] probably come from environmental sources (e.g., water, air, and soil).

### 3.7. The Glycan α-Gal Content is Higher in the Cementome than in the Sialome throughout Tick Feeding

Glycans are constituents of matrix proteins, which are important for maintenance of tissue structure, porosity, integrity, and matrix organization through binding to other glycoproteins [64]. We focused the study on the glycan α-Gal (C_12_H_8_O_11_; Figure S1) that is present in glycoproteins and glycolipids from tick saliva that mediate the alpha-Gal syndrome (AGS) characterized by delayed anaphylaxis to red meat consumption, and immediate anaphylaxis to tick bites, xenotransplantation, and certain drugs such as cetuximab [65,66,67,68,69,70,71,72,73,74,75,76]. Several tick species including *Rhipicephalus* spp. have been associated with the AGS and the production of tick proteins with α-Gal modifications has been demonstrated in various tick species including *R. microplus* [70,75]. The results showed that the α-Gal content in *R. microplus* tick sialome and cementome was higher than in proteins from α-Gal-negative human HL60 cells (black asterisks in Figure 8C). The comparison between samples showed higher α-Gal content in the cementome than in the sialome at all time points (red asterisks in Figure 8C). Furthermore, the α-Gal content at different tick feeding stages (T1–T3) decreased with tick feeding in the sialome but did not change in the cementome (Figure 8C). The results suggested that although host-derived proteins or lipids may contribute to α-Gal content in both salivary glands and cement, the fact that α-Gal levels decreased with tick feeding in the sialome but remained higher in the cementome support that at least some of these components are not storage but synthesized and secreted from the salivary glands. These results suggested a possible role for α-Gal-containing compounds in cement composition.

### 3.8. Western Blot Analysis of Selected Tick and Cattle Host Derived Cementome Proteins Support Proteomics Results

Herein, we used a validated label-free relative quantitation by sequential window acquisition of all theoretical mass spectra (SWATH) approach for proteomics analysis [77]. Nevertheless, selected tick and host derived proteins were used for providing additional support to proteomics data using Western blot or dot blot analyses in both cementome and sialome (Figures S5–S8). Tick-derived cementome proteins fulfilling three to four of the selection criteria were selected for analysis (Histones, Glycine-rich superfamily member proteins, and aminopeptidase N; Table S1). These proteins were putatively involved in cement formation, solidification and maintenance, tick feeding, and detachment (Figure 4A), and showed representation profiles assigned to molting, secondary cement production, cement maintenance, feeding, and detachment (Figures S5 and S6). These proteins were also annotated into the biological processes of protein heterodimerization, putative cell wall structural and/or cuticle protein, and proteolysis (Figure 3B). Host-derived alpha-2-HS-glycoprotein, desmoplakin and pan-keratins identified in the sialome and cementome were selected for analysis because were putatively involved in cement formation, solidification, and maintenance (Figure 5B and Table S2). These proteins showed representation profiles assigned to secondary cement production, detachment, and feeding and oogenesis (Figures S7 and S8).

The quantitative results of Western blot or dot blot analyses of both sialome and cementome at different feeding times showed protein profiles similar to those described by proteomics analysis (Figures S5–S8). Despite the limitations posed by protein families such as Histones, Glycine-rich superfamily member proteins, and pan-keratins with multiple profiles, the results provided additional support for proteomics dataset and the possibility of using these antibodies for additional studies.

## 4. Conclusions

Our experimental approach using proteomics allowed the high throughput identification of tick and host derived proteins in both sialome (2264 proteins) and cementome (654 proteins) of *R. microplus* female ticks collected at different feeding stages. Innovative experimental approaches may provide new insights to complex biological questions [78]. Based on a novel methodology for proteomics data analysis using the protein representation profiles after significant differences in pairwise comparisons between time points, proteins were putatively assigned to different categories of developmental processes. This methodology provides a functional relevant alternative to other methodologies such as the analyses using QuiXoT [32] or normalized spectral abundance factors (NSAF) and z-score statistics [54]. Additionally, the first analysis of the chemical elements using SEM combined with EDS allowed the characterization of changes in their composition in tick salivary glands and cement during tick feeding, which correlated with changes in protein profiles. The glycan α-Gal content in the tick sialome and cementome also showed differences between samples and time points and suggested a possible role for α-Gal-containing compounds in cement composition. These results advanced our knowledge of the tick cement composition. The dynamics of cement composition throughout adult tick feeding stages suggested that tick and host derived compounds modulate the biochemical properties of the cement complex substance. Future experiments should focus on providing experimental evidence for the role of the different compounds in modulating tick cement biochemical properties and function.

Both cattle host and tick derived proteins were identified in the *R. microplus* sialome and cementome. Some of the tick-derived proteins identified in this study in the *R. microplus* cementome have been previously reported and characterized in the tick sialome/sialotranscriptome (e.g., Table S5). Similarly, host-derived proteins such as immunoglobulins and apolipoprotein A-I identified here in the cementome have been previously described in the sialome [6,12,54,79,80,81,82,83,84,85] and in unfed ticks [86,87]. However, the cementome protein content described here supports a highly complex composition of both tick and host derived proteins involved in multiple biological processes such as cement formation, solidification and maintenance, feeding, interference with host immune response, and detachment playing a key role in tick biology. Additionally, pending functional studies, we propose that host-derived cementome proteins may complement and synergize with tick-derived proteins in cement structure and function.

**Figure 9:**
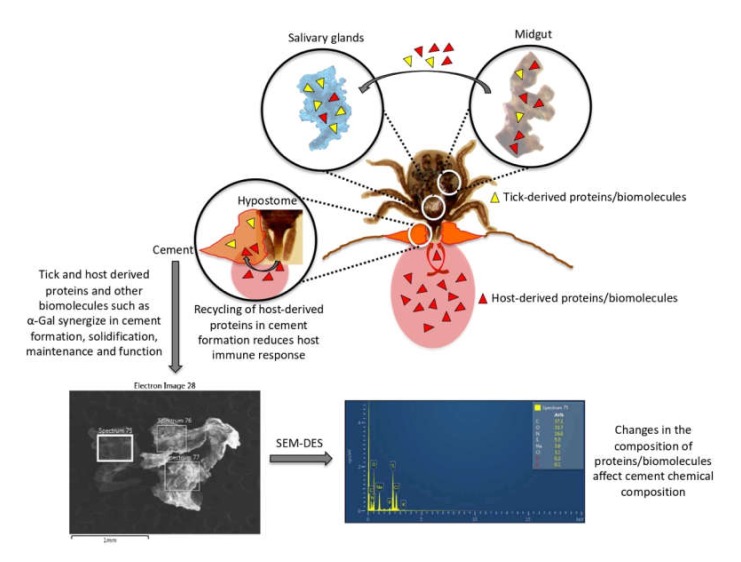
The combination of tick and host derived proteins in the cementome resulted in an evolutionary adaptation to long-lasting ectoparasitic blood feeding. Our hypothesis is that ticks evolved to combine tick and host derived proteins and other biomolecules such as α-Gal to synergize in cement formation, solidification, and maintenance to facilitate attachment, feeding, interference with host immune response, and detachment. Changes in the composition of proteins/biomolecules affect cement chemical composition. In addition to the role of cementome proteins in the inhibition/interference with host immune response, the recycling of host-derived proteins in cement formation reduces host immune response as these proteins are recognized as self-antigens. Representative images of SEM–EDS analysis are shown, and all results are displayed in Data S5.

Based on these results, our hypothesis is that ticks evolved to combine tick and host derived proteins and other biomolecules such as α-Gal to synergize in cement formation, solidification and maintenance to facilitate attachment, feeding, interference with host immune response, and detachment (Figure 9). In addition to the role of cementome proteins in the inhibition/interference with host immune response, the recycling of host-derived proteins in cement formation reduces host immune response as these proteins are recognized as self-antigens (Figure 8). In this way, the combination of tick and host derived proteins in the cementome resulted in an evolutionary adaptation to long-lasting ectoparasitic blood feeding.

Future studies using these data could be focused on the characterization of salivary gland proteins in early feeding stages of *R. microplus*. Finally, based on currently available information and the results obtained in this study, we predict that the characterization of the tick cementome will contribute to developing vaccines targeting tick adhesion and feeding for the control of tick infestations and pathogen transmission (Table S6) [14,20,88,89,90] and new applications in medicine and industry as a biological glue [21,29,91].

## Supplementary Materials

The following are available online at https://www.mdpi.com/2218-273X/10/4/555/s1, Supporting Information [Table S1: *R. microplus* tick-derived proteins identified in the cementome. Table S2: Cattle host-derived proteins identified in *R. microplus* tick cementome. Table S3: Comparative analysis of amino acid composition of cattle host and tick *R. microplus* derived proteins identified in the cementome. Table S4: Recombinant tick proteins used for validation by Western blot analysis. Table S5: Examples of tick-derived proteins identified in this study in the cementome and previously characterized and reported in the sialome/sialotranscriptome. Table S6: Published results about the potential role of selected tick cementome proteins for novel vaccine development. Figure S1: Chemical structure and atomic composition of Galα1-3Galβ1-(3)4GlcNAc-R (α-Gal). Figure S2: ELISA test for characterizing α-Gal levels. Figure S3: Validation of commercial antibodies against host proteins by immunofluorescence assay. Figure S4: Western blot analysis of recombinant tick proteins. Figure S5: Western blot analysis of tick-derived aminopeptidase N protein in the *R. microplus* cementome (Cement) and sialome (SG). Figure S6: Dot blot analysis of tick-derived histones and Glycine-rich superfamily member proteins in the *R. microplus* cementome (Cement) and sialome (SG). Figure S7: Dot blot analysis of host-derived desmoplakin and alpha-2-HS-glycoprotein in the *R. microplus* cementome (Cement) and sialome (SG). Figure S8: Dot blot analysis of host-derived pan-keratins in the *R. microplus* cementome (Cement) and sialome (SG)]. Data S1: Proteomics analysis of *R. microplus* tick sialome. Data S2: Proteomics analysis of *R. microplus* tick cementome with urea-extracted proteins. Data S3: Proteomics analysis of *R. microplus* tick cementome with SDS-extracted proteins. Data S4: Protein profile of tick sialome and cementome throughout tick *R. microplus* parasitic stages. Data S5: Composition of chemical elements in *R. microplus* tick cement and salivary glands. Data S6: Statistical analysis of chemical element composition in *R. microplus* tick cement and salivary glands. Data S7: Peptide sequences for tick *R. microplus* and cattle host derived proteins identified in the sialome and cementome. Host-derived proteins in the cementome were included only if identified in both sialome and cementome.
