# Supplementary material for: Tick and Host Derived Compounds Detected in the Cement Complex Substance
Source: Biomolecules. 2020 Apr 5;10(4):555. doi: 10.3390/biom10040555 (PMC7226240; doi:10.3390/biom10040555)
Supplement: Supplementary file 1 [file biomolecules-10-00555-s001.zip › Supporting Information.pdf]

**Supporting Information**

**Tables S1-S4 and Figures S1-S8**

# Tick and host derived compounds modulate the biochemical properties of the cement complex substance

Margarita Villar[1,2§], Iván Pacheco[1§], Octavio Merino[3§], Marinela Contreras[1§], Lourdes Mateos-Hernández[1,4], Eduardo Prado[5], Dina Karen Barros-Picanço[1], José Francisco Lima-Barbero[1,6], Sara Artigas-Jerónimo[1], Pilar Alberdi[1], Isabel G. Fernández de Mera[1], Agustín Estrada-Peña[7], Alejandro Cabezas-Cruz[4], José de la Fuente[1,8]

[1]SaBio, Instituto de Investigación en Recursos Cinegéticos (IREC-CSIC-UCLM-JCCM), Ronda de Toledo s/n, 13005 Ciudad Real, Spain.

[2]Biochemistry Section, Faculty of Science and Chemical Technologies, and Regional Centre for Biomedical Research (CRIB), University of Castilla-La Mancha, 13071, Ciudad Real, Spain.

[3]Facultad de Medicina Veterinaria y Zootecnia, Universidad Autónoma de Tamaulipas, Km 5, Carretera Victoria-Mante, CP 87000 Ciudad Victoria, Tamaulipas, Mexico.

[4]UMR BIPAR, INRA, ANSES, Ecole Nationale Vétérinaire d'Alfort, Université Paris-Est, Maisons-Alfort, 94700, France.

[5]Department of Applied Physics, Faculty of Chemical Sciences and Technologies, Universidad de Castilla-La Mancha, Avda. Camilo José Cela 10, 13071 Ciudad Real, Spain.

[6]Sabiotec, Camino de Moledores s/n. 13003, 13071 Ciudad Real, Spain.

[7]Facultad de Veterinaria, Universidad de Zaragoza, 50013 Zaragoza, Spain.

[8]Department of Veterinary Pathobiology, Center for Veterinary Health Sciences, Oklahoma State University, Stillwater, OK 74078, USA.

[§]Equal contribution.

Email: jose_delafuente@yahoo.com / josedejesus.fuente@uclm.es

**Table S1.** *R. microplus* tick-derived proteins identified in the cementome.

| **Protein ID, name and (criteria for selection)** | **Representation profile category** | **Biological Process** |
|---|---|---|
| V5HZA1<br>Histone H2A<br>(a, c) | SG: Feeding & oogenesis<br>CE: Feeding & oogenesis | Protein heterodimerization |
| V5HJX3<br>Histone H2A<br>(b, c) | CE: Feeding & oogenesis | Protein heterodimerization |
| L7LVH9<br>Histone H3<br>(a, c) | SG: Feeding & oogenesis<br>CE: Detachment | Protein heterodimerization |
| Q4PM69¶*<br>Histone H4<br>(a, b, c, d) | SG: Molting<br>CE: Secondary cement production I | Protein heterodimerization |
| A0A131XKB4<br>Puromycin-sensitive aminopeptidase<br>(a, c) | SG: Cement maintenance<br>CE: Detachment | Proteolysis |
| Q45R50<br>Salivary gland metalloprotease<br>(a, c) | SG: Molting<br>CE: Cement maintenance | Proteolysis |
| A0A131YLU7/A0A224Y1G3*<br>Aminopeptidase N<br>(b, c, d) | CE: Feeding<br>SG: Detachment | Proteolysis |
| A0A224Z8F0<br>Transglutaminase/protease like protein<br>(b, c, d) | CE: Detachment | Proteolysis |
| A0A131ZAV6<br>Reprolysin<br>(c) | CE: Detachment | Proteolysis |
| A0A224YTI7<br>Angiotensin-converting enzyme<br>(c, d) | CE: Secondary cement production I | Proteolysis |
| A0A224YD77<br>Gluzincin<br>(c) | CE: Cement maintenance | Proteolysis |
| A0A131YF72<br>Glycine-rich superfamily member<br>(b, c) | CE: Detachment | Putative cell wall structural and/or cuticle protein |
| A0A034WWS7<br>Secreted cement protein 2<br>(b, c) | CE: Molting | Putative cell wall structural and/or cuticle protein |
| L7MDN1<br>Putative glycine rich protein | CE: Molting | Putative cell wall structural and/or cuticle protein<br>63% identity (E=6.8e-40) to *Amblyomma* |

| | | |
|---|---|---|
| (b, c) | | *parvum* Putative nuclear pore proteinucleoporin 62 (A0A023G321) |
| A0A224YCP1<br>Glycine-rich superfamily member<br>(b, c) | CE: Cement maintenance | Putative cell wall structural and/or cuticle protein |
| A0A224YEB4<br>Glycine-rich superfamily member<br>(b, c) | CE: Secondary cement production II | Putative cell wall structural and/or cuticle protein |
| A0A034WZ79<br>Glycine-rich protein 3<br>(b, c) | CE: Secondary cement production II | Putative cell wall structural and/or cuticle protein |
| L7M1K6<br>Putative glycine rich protein<br>(b, c) | CE: Feeding | Putative cell wall structural and/or cuticle protein |
| A0A224YEQ4¶*<br>Glycine rich superfamily member<br>(a, b, c) | SG: Molting<br>CE: Cement maintenance | Putative cell wall structural and/or cuticle protein<br>76% identity (E=0.0) to *Tabanus bromius* Putative alpha-1 collagen type iii (A0A0K8TMJ9) |
| A0A224Y271<br>Pancreatic trypsin inhibitor<br>(c) | CE: Molting | Negative regulation of endopeptidase activity |
| A0A131YNG2<br>Pancreatic trypsin inhibitor<br>(c) | CE: Cement maintenance | Negative regulation of endopeptidase activity |
| A0A224Y1X4<br>Pancreatic trypsin inhibitor<br>(c) | CE: Cement maintenance | Negative regulation of endopeptidase activity |
| L7LRL1<br>Putative bilaris<br>(c) | CE: Molting | Negative regulation of endopeptidase activity |
| L7ME64<br>Putative bilaris<br>(c) | CE: Cement maintenance | Negative regulation of endopeptidase activity |
| A0A224YMC8<br>Lipocalin<br>(c) | CE: Molting | Evasion or tolerance of host defense response |
| A0A224YB96<br>Lipocalin<br>(c) | CE: Secondary cement production II | Evasion or tolerance of host defense response |
| L7LTA8<br>Putative group II salivary lipocalin<br>(c) | CE: Secondary cement production II | Evasion or tolerance of host defense response |
| G3MKF8<br>Tubulin beta chain<br>(a) | SG: Feeding & oogenesis<br>CE: Feeding & oogenesis | Cytoskeleton organization & microtubule-based process |
| A0A034WXM6<br>Secreted protein 20<br>(a) | SG: Feeding & oogenesis<br>CE: Detachment | Unknown<br>43% identity (E= 4.8e-55) to *Odontobuthus doriae* (Yellow Iranian scorpion) Venom |

| | | |
|---|---|---|
| | | protein VP6 (A0A0U3YCW0) |
| A0A034WXN6<br>Hemelipoprotein HeLp 2<br>(a) | SG: Cement maintenance<br>CE: Cement maintenance | Lipid transport |
| A0A034WZ42<br>Hemelipoprotein HeLp<br>(a) | SG: Secondary cement production I<br>CE: Cement maintenance | Lipid transport |
| A0A224YTB6<br>Alpha-N-acetylgalactosaminidase<br>(a) | SG: Secondary cement production I<br>CE: Secondary cement production II | Carbohydrate metabolic process |
| L7M046<br>Cuticular protein<br>(a) | SG: Molting<br>CE: Secondary cement production II | Chitin metabolic process |
| A0A0K8RP30<br>Putative ubiquitin/40s ribosomal protein s27a fusion<br>(b) | CE: Feeding and oogenesis | Unknown |
| A0A0B4PMF7<br>Peptidoglycan recognition protein<br>(d) | CE: Cement maintenance | Peptidoglycan catabolic process |
| A0A131YG55<br>Peptidoglycan recognition protein<br>(d) | CE: Cement maintenance | Peptidoglycan catabolic process |
| B7Q181<br>Cytochrome P450<br>(d) | CE: Cement maintenance | Metabolic process |
| L7M9R2<br>Putative animal peptidoglycan recognition protein logous to bacteriophage t3 lysozyme<br>(d) | CE: Secondary cement production II | Innate immune response<br>Peptidoglycan catabolic process |

Tick cementome proteins were first selected based on one of the following criteria: (a) identified in both sialome and cementome, (b) being among the two proteins with highest representation in feeding, feeding and oogenesis, molting, secondary cement production, cement maintenance or detachment categories, and predicted to play a key role in tick-host interactions due to (c) over 4% representation in biological processes (Figure 4B) or (d) after network analysis. ¶These proteins appeared also as one of the two proteins with highest representation in secondary cement production (Q4PM69 and A0A224YU86) or cement maintenance (A0A224YEQ4). *Proteins selected for validation correspond to those fulfilling 3 to 4 of the selection criteria a-d. Abbreviations: SG, salivary gland; CE, cement.

**Table S2.** Cattle host-derived proteins identified in *R. microplus* tick cementome.

| Protein ID and name | Representation profile category | Biological Process |
|---|---|---|
| P00921<br>Carbonic anhydrase 2 | SG: Feeding & oogenesis<br>CE: Feeding & oogenesis | Angiotensin-activated signaling pathway<br>Positive regulation of dipeptide transmembrane transport<br>Regulation of anion transport<br>Regulation of intracellular Ph |
| F1MSZ6 Antithrombin-III | SG: Feeding & oogenesis<br>CE: Feeding & oogenesis | Negative regulation of endopeptidase activity<br>Regulation of blood coagulation, intrinsic pathway |
| P34955 Alpha-1-antiproteinase | SG: Feeding & oogenesis<br>CE: Feeding & oogenesis | Negative regulation of endopeptidase activity |
| P63243 Receptor of activated protein C kinase 1 | SG: Secondary cement production<br>CE: Feeding & oogenesis | Cell cycle<br>Cellular response to glucose stimulus<br>Cellular response to growth factor stimulus<br>Gastrulation<br>Negative regulation of cell growth<br>Negative regulation of gene expression<br>Negative regulation of hydrogen peroxide-induced neuron death<br>Negative regulation of peptidyl-serine phosphorylation<br>Negative regulation of phagocytosis<br>Negative regulation of protein kinase B signaling<br>Negative regulation of Wnt signaling pathway<br>Pigmentation<br>Positive regulation of apoptotic process<br>Positive regulation of cell migration<br>Positive regulation of cyclic-nucleotide phosphodiesterase activity<br>Positive regulation of gastrulation<br>Positive regulation of Golgi to plasma membrane protein transport<br>Positive regulation of GTPase activity<br>Positive regulation of intrinsic apoptotic signaling pathway<br>Positive regulation of mitochondrial depolarization<br>Positive regulation of proteasomal ubiquitin-dependent protein catabolic process<br>Positive regulation of protein homooligomerization<br>Positive regulation of protein phosphorylation<br>Protein ubiquitination<br>Regulation of cell cycle |

| | | |
|---|---|---|
| | | Regulation of cell división<br>Regulation of establishment of cell polarity<br>Regulation of protein localization<br>Rescue of stalled ribosome<br>Rhythmic process |
| Q1JPB0 Leukocyte elastase inhibitor | SG: Feeding<br>CE: Feeding & oogenesis | Negative regulation of endopeptidase activity |
| Q2KJF1 Alpha-1B-glycoprotein Immunoglobulin supergene family | SG: Feeding & oogenesis<br>CE: Feeding & oogenesis | Neutrophil degranulation<br>Platelet degranulation |
| Q8SPJ1 Junction plakoglobin | SG: Secondary cement production<br>CE: Feeding & oogenesis | Bundle of His cell-Purkinje myocyte adhesion involved in cell communication<br>Cell migration<br>Cellular response to indole-3-methanol<br>Desmosome assembly<br>Detection of mechanical stimulus<br>Endothelial cell-cell adhesión<br>Negative regulation of blood vessel endothelial cell migration<br>Positive regulation of angiogénesis<br>Positive regulation of cell-matrix adhesión<br>Positive regulation of DNA-binding transcription factor activity<br>Positive regulation of protein import into nucleus<br>Positive regulation of transcription by RNA polymerase II<br>Protein localization to plasma membrane<br>Regulation of cell population proliferation<br>Regulation of heart rate by cardiac conduction<br>Regulation of ventricular cardiac muscle cell action potential<br>Skin development |
| Q9BGI3Peroxiredoxin-2 | SG: Feeding & oogenesis<br>CE: Feeding & oogenesis | Activation of MAPK activity<br>Cell redox homeostasis<br>Homeostasis of number of cells<br>Hydrogen peroxide catabolic process<br>Leukocyte activation<br>Negative regulation of extrinsic apoptotic signaling pathway in absence of ligand<br>Negative regulation of lipopolysaccharide-mediated signaling pathway<br>Negative regulation of NF-kappaB transcription factor activity<br>Negative regulation of reactive oxygen species metabolic process<br>Negative regulation of T cell differentiation<br>Positive regulation of blood coagulation<br>Regulation of hydrogen peroxide metabolic process<br>Removal of superoxide radicals<br>Respiratory burst involved in inflammatory |

| | | |
|---|---|---|
| | | response<br>Response to lipopolysaccharide<br>Response to oxidative stress<br>T cell proliferation<br>Thymus development |
| A0A0F6QNP7 Complement component 3 | SG: Feeding & oogenesis<br>CE: Feeding & oogenesis | Complement activation<br>Inflammatory response |
| G5E604Uncharacterized protein | SG: Feeding & oogenesis<br>CE: Feeding & oogenesis | Immune response<br>Immunoglobulin production |
| B0JYN6* Alpha-2-HS-glycoprotein | SG: Feeding & oogenesis<br>CE: Feeding & oogenesis | Acute-phase response<br>Negative regulation of bone mineralization<br>Ossification<br>Positive regulation of phagocytosis<br>Regulation of inflammatory response |
| B5T254 Peptidoglycan-recognition protein | SG: Secondary cement production<br>CE: Feeding & oogenesis | Innate immune response<br>Peptidoglycan catabolic process |
| D4QBB4 Globin A1 | SG: Feeding & oogenesis<br>CE: Feeding & oogenesis | Oxygen transport |
| E1B6Z6Lipocalin 2 | SG: Feeding & oogenesis<br>CE: Feeding & oogenesis | Defense response to bacterium<br>Extrinsic apoptotic signaling pathway in absence of ligand<br>Innate immune response<br>Positive regulation of cold-induced thermogenesis<br>Response to virus<br>Sequestering of iron ion<br>Siderophore transport |
| E1BKT9* Desmoplakin | SG: Secondary cement production<br>CE: Feeding & oogenesis | Adherens junction organization<br>Bundle of His cell-Purkinje myocyte adhesion involved in cell communication<br>Cell-cell adhesion<br>Desmosome organization<br>Intermediate filament cytoskeleton organization<br>Intermediate filament organization<br>Keratinocyte differentiation<br>Peptide cross-linking<br>Protein localization to adherens junction<br>Regulation of heart rate by cardiac conduction<br>Regulation of ventricular cardiac muscle cell action potential<br>Skin development<br>Ventricular compact myocardium morphogenesis |

| | | |
|---|---|---|
| | | Wound healing |
| F1N076Ceruloplasmin | SG: Feeding & oogenesis<br>CE: Feeding & oogenesis | Cellular iron ion homeostasis<br>Copper ion transport<br>Iron ion homeostasis<br>Iron ion transport |
| G3N0V2[¶]*Keratin 1 | SG: Secondary cement production<br>CE: Feeding & oogenesis | Complement activation, lectin pathway<br>Establishment of skin barrier<br>Negative regulation of inflammatory response<br>Peptide cross-linking<br>Protein heterotetramerization |
| G5E513Uncharacterized protein | SG: Feeding & oogenesis<br>CE: Feeding & oogenesis | B cell receptor signaling pathway<br>Complement activation, classical pathway<br>Defense response to bacterium<br>Innate immune response<br>Phagocytosis, engulfment<br>Phagocytosis, recognition<br>Positive regulation of B cell activation |
| G8JKW7 Uncharacterized protein | SG: Feeding & oogenesis<br>CE: Feeding & oogenesis | Negative regulation of endopeptidase activity |
| Q5EA67 Inter-alpha (Globulin) inhibitor H4 (Plasma Kallikrein-sensitive glycoprotein) | SG: Feeding & oogenesis<br>CE: Feeding & oogenesis | Hyaluronan metabolic process |
| Q5J801 Endopin 2B | SG: Feeding & oogenesis<br>CE: Feeding & oogenesis | Neural regulation |
| V6F9A2 Apolipoprotein A-I preproprotein | SG: Feeding & oogenesis<br>CE: Feeding & oogenesis | Adrenal gland development<br>Blood vessel endothelial cell migration<br>Cholesterol biosynthetic process<br>Cholesterol efflux<br>Cholesterol homeostasis<br>Cholesterol import<br>Endothelial cell proliferation<br>Glucocorticoid metabolic process<br>G protein-coupled receptor signaling pathway<br>High-density lipoprotein particle assembly<br>High-density lipoprotein particle remodeling<br>Integrin-mediated signaling pathway<br>Lipid storage<br>Lipoprotein biosynthetic process<br>Lipoprotein metabolic process<br>Negative regulation of cell adhesion molecule production<br>Negative regulation of cytokine secretion |

| | | |
|---|---|---|
| | | involved in immune response<br>Negative regulation of heterotypic cell-cell adhesion<br>Negative regulation of hydrolase activity<br>Negative regulation of inflammatory response<br>Negative regulation of interleukin-1 beta secretion<br>Negative regulation of tumor necrosis factor-mediated signaling pathway<br>Negative regulation of very-low-density lipoprotein particle remodeling<br>Peptidyl-methionine modification<br>Phosphatidylcholine biosynthetic process<br>Phospholipid efflux<br>Phospholipid homeostasis<br>Positive regulation of cholesterol efflux<br>Positive regulation of cholesterol esterification<br>Positive regulation of hydrolase activity<br>Positive regulation of lipid biosynthetic process<br>Positive regulation of phagocytosis<br>Positive regulation of phospholipid efflux<br>Positive regulation of Rho protein signal transduction<br>Positive regulation of stress fiber assembly<br>Positive regulation of substrate adhesion-dependent cell spreading<br>Protein oxidation<br>Protein stabilization<br>Regulation of Cdc42 protein signal transduction<br>Regulation of cholesterol transport<br>Regulation of intestinal cholesterol absorption<br>Regulation of protein phosphorylation<br>Reverse cholesterol transport<br>Triglyceride catabolic process<br>Triglyceride homeostasis<br>Very-low-density lipoprotein particle remodeling<br>Vitamin transport |
| F1MN78 Serpin B4 | SG: Secondary cement production<br>CE: Feeding & oogenesis | Negative regulation of peptidase activity<br>Protection from natural killer cell mediated cytotoxicity<br>Regulation of proteolysis |
| A0A1K0FUD3Globin C1 | SG: Feeding & oogenesis<br>CE: Feeding & oogenesis | Oxygen transport<br>Transport |
| F1MC11[¶]* Keratin, type I cytoskeletal 14 | SG: Secondary cement production<br>CE: Feeding & oogenesis | Aging<br>Epithelial cell differentiation<br>Hair cycle<br>Intermediate filament bundle assembly |
| Q7SIH1 Alpha-2-macroglobulin | SG: Feeding & oogenesis | Blood coagulation, intrinsic pathway<br>Extracellular matrix disassembly |

| | | |
|---|---|---|
| | CE: Feeding & oogenesis | Negative regulation of complement activation, lectin pathway<br>Platelet degranulation<br>Regulation of small GTPase mediated signal transduction<br>Stem cell differentiation |
| M0QVY0Uncharacterized protein | SG: Secondary cement production<br>CE: Feeding & oogenesis | Unknown |
| Q1RMN8Immunoglobulin light chain, lambda gene cluster | SG: Secondary cement production<br>CE: Feeding & oogenesis | B cell receptor signaling pathway<br>Complement activation, classical pathway<br>Defense response to bacterium<br>Innate immune response<br>Leukocyte migration<br>Phagocytosis, recognition and engulfment<br>Positive regulation of B cell activation |
| G5E5H7Uncharacterized protein | SG: Secondary cement production<br>CE: Feeding | Unknown |
| A6QNZ7*Keratin 10 (Epidermolytic hyperkeratosis; keratosis palmaris et plantaris) | SG: Secondary cement production<br>CE: Feeding | Keratinocyte differentiation<br>Peptide cross-linking<br>Protein heterotetramerization |
| P02662Alpha-S1-casein | SG: Secondary cement production<br>CE: Detachment | Negative regulation of apoptotic process<br>Negative regulation of gene expression<br>Negative regulation of supramolecular fiber organization<br>Positive regulation of androst-4-ene-3,17-dione biosynthetic process<br>Positive regulation of androstenedione secretion<br>Positive regulation of progesterone biosynthetic process<br>Positive regulation of progesterone secretion<br>Protein stabilization<br>Response to 11-deoxycorticosterone<br>Response to dehydroepiandrosterone<br>Response to estradiol<br>Response to growth hormone<br>Response to progesterone |
| P02663Alpha-S2-casein | SG: Secondary cement production<br>CE: Detachment | Defense response to bacterium<br>Response to 11-deoxycorticosterone<br>Response to dehydroepiandrosterone<br>Response to estradiol<br>Response to growth hormone<br>Response to progesterone |
| Q0P5J4*Keratin, type I cytoskeletal 25 | SG: Secondary cement production<br>CE: Detachment | Aging<br>Cytoskeleton organization<br>Hair follicle morphogenesis<br>Intermediate filament organization |

| | | |
|---|---|---|
| Q08D91* Keratin, type II cytoskeletal 75 | SG: Secondary cement production<br>CE: Detachment | Hematopoietic progenitor cell differentiation<br>Protein polymerization |
| Q148H5* Keratin, type II cytoskeletal 71 | SG: Feeding<br>CE: Detachment | Hair follicle morphogenesis<br>Intermediate filament organization<br>Protein polymerization |
| A0A140T897 Serum albumin | SG: Feeding & oogenesis<br>CE: Detachment | Cellular response to starvation<br>Maintenance of mitochondrion location<br>Negative regulation of apoptotic process<br>Negative regulation of protein oligomerization |
| A5PJE3Fibrinogen alpha chain | SG: Feeding & oogenesis<br>CE: Detachment | Platelet activation<br>Protein polymerization |
| D4QBB3 Hemoglobin beta | SG: Feeding & oogenesis<br>CE: Detachment | Oxygen transport<br>Transport |
| G3MZ71* Keratin 2 | SG: Secondary cement production<br>CE: Detachment | Intermediate filament organization<br>Keratinization<br>Keratinocyte activation<br>Keratinocyte development<br>Keratinocyte migration<br>Keratinocyte proliferation<br>Peptide cross-linking<br>Positive regulation of epidermis development |
| Q3SZZ9FGG protein | SG: Feeding & oogenesis<br>CE: Detachment | Platelet activation<br>Protein polymerization |
| Q9TTE1Serpin A3-1 | SG: Feeding & oogenesis<br>CE: Secondary cement production | Negative regulation of endopeptidase activity |
| G3N0V0 Uncharacterized protein | SG: Feeding & oogenesis<br>CE: Secondary cement production | B cell receptor signaling pathway<br>Complement activation, classical pathway<br>Defense response to bacterium<br>Innate immune response<br>Phagocytosis, engulfment<br>Phagocytosis, recognition<br>Positive regulation of B cell activation |
| Q3SZV7Hemopexin | SG: Secondary cement production<br>CE: Secondary cement production | Cellular iron ion homeostasis<br>Heme metabolic process<br>Hemoglobin metabolic process<br>Positive regulation of humoral immune response mediated by circulating immunoglobulin<br>Positive regulation of immunoglobulin production<br>Positive regulation of interferon-gamma-mediated signaling pathway<br>Positive regulation of tyrosine phosphorylation of STAT protein |

| | | |
|---|---|---|
| G5E5T5Uncharacterized protein | SG: Feeding<br>CE: Secondary cement production | Unknown |
| G3X6N3Serotransferrin | SG: Feeding & oogenesis<br>CE: Secondary cement production | Cellular iron ion homeostasis<br>Cellular response to iron ion<br>Positive regulation of receptor-mediated endocytosis<br>Regulation of iron ion transport |
| F1MAV0 Fibrinogen beta chain | SG: Feeding & oogenesis<br>CE: Secondary cement production | Platelet activation<br>Protein polymerization |
| G3MXL3* Keratin 3 | SG: Secondary cement production<br>CE: Secondary cement production | Cornification<br>Epithelial cell differentiation<br>Intermediate filament cytoskeleton organization<br>Keratinization |
| A4FUZ0* Keratin, type II cuticular Hb3 | SG: Feeding<br>CE: Secondary cement production | Aging<br>Cornification<br>Epidermis development<br>Keratinization |
| Q148I8* Keratin 31 | SG: Secondary cement production<br>CE: Secondary cement production | Cornification<br>Epidermis development<br>Keratinization |

Cattle host-derived proteins identified in *R. microplus* tick cementome were selected based on the identification in both sialome and cementome. Abbreviations: SG, salivary gland; CE, cement. ¶Proteins represented with more than 1 million TAS at all time points. *Proteins selected for validation correspond to those putatively involved in cement formation, solidification and maintenance.

**Table S3.** Comparative analysis of amino acid composition of cattle host and tick *R. microplus* derived proteins identified in the cementome.

| **Parameter** | **Host** | **Tick** | **Host cement** | **Tick cement** |
|---|---|---|---|---|
| Theoretical pI | 4.75 | 4.88 | 4.76 | 5.73 |
| Amino acid composition | | | | |
| Ala (A) | 9.2% | 8.7% | 12.5% | 6.8% |
| Arg (R) | 4.2% | 4.0% | 3.1% | 4.8% |
| Asn (N) | 4.0% | 4.2% | 5.1% | 3.0% |
| Asp (D) | 5.8% | 6.0% | 5.2% | 4.2% |
| Cys (C) | 1.6% | 1.8% | 1.8% | 2.0% |
| Gln (Q) | 4.6% | 3.8% | 5.9% | 2.7% |
| Glu (E) | 7.5% | 7.1% | 8.8% | 4.7% |
| Gly (G) | 10.2% | 8.4% | 3.9% | 15.5% |
| His (H) | 1.5% | 2.3% | 1.9% | 1.8% |
| Ile (I) | 5.3% | 4.6% | 4.6% | 3.9% |
| Leu (L) | 9.6% | 10.5% | 8.9% | 10.2% |
| Lys (K) | 4.2% | 4.4% | 5.3% | 2.6% |
| Met (M) | 0.7% | 0.6% | 0.7% | 0.6% |
| Phe (F) | 3.6% | 4.5% | 5.5% | 4.8% |
| Pro (P) | 6.3% | 5.6% | 5.7% | 6.3% |
| Ser (S) | 6.4% | 6.6% | 6.3% | 8.0% |
| Thr (T) | 5.4% | 5.2% | 3.0% | 4.3% |
| Trp (W) | 0.5% | 0.9% | 1.0% | 0.8% |
| Tyr (Y) | 2.6% | 3.1% | 3.8% | 6.4% |
| Val (V) | 7.0% | 7.9% | 7.1% | 6.6% |
| Pyl (O) | 0.0% | 0.0% | 0.0% | 0.0% |
| Sec (U) | 0.0% | 0.0% | 0.0% | 0.0% |
| Asn or Asx (B) | n = 10.0% | n = 0.0% | n = 0.0% | n = 0.0% |
| Gln or Glu (Z) | n = 0.0% | n = 0.0% | n = 0.0% | n = 0.0% |
| Unknown (X) | n = 0.0% | n = 0.0% | n = 0.0% | n = 0.0% |
| Percent of negatively charged residues (D + E) | 13.3% | 13.2% | 14.0% | 8.9% |
| Percent of positively charged residues (R + K) | 8.4% | 8.4% | 8.4% | 7.4% |
| Ratio of negatively to positively charged residues | 1.6 | 1.6 | 1.7 | 1.2 |
| Atomic composition* | Unknown | | | |
| Carbon C | | 31.9% | 32.2% | 32.5% |
| Hydrogen H | | 49.6% | 49.4% | 49.2% |
| Nitrogen N | | 8.5% | 8.4% | 8.6% |
| Oxygen O | | 9.8% | 9.8% | 9.6% |
| Sulfur S | | 0.2% | 0.2% | 0.2% |
| Instability index** | 37.91 Stable | 37.47 Stable | 37.30 Stable | 36.74 Stable |
| Aliphatic index** | 87.31 | 90.29 | 85.60 | 81.02 |
| Grand average of hydropathicity (GRAVY)** | -0.220 | -0.147 | -0.245 | -0.073 |

Data for all cattle host-derived proteins identified in the cementome but not in the sialome (Host), 100 randomly selected tick-derived proteins identified in the sialome but not in the cementome (Tick), all host-derived proteins identified in the sialome and cementome (Host cement), and all tick-derived proteins identified in the cementome (Tick cement) (Data S7) were analyzed using the ProtParam tool (https://web.expasy.org/protparam/).
*When at least one ambiguous position (B, Z or X) is found in the sequence considered, the atomic composition cannot be computed.
**Calculated for the sum of all peptide sequences.

**Table S4.** Recombinant tick proteins used for validation by Western blot analysis.

| Protein name and ID | DNA and RT-PCR primer sequences | Protein sequence |
|---|---|---|
| Q4PM69<br>Histone H4 | >Complete cds<br>atgtctggccgtggcaaaggtggcaaggggctc<br>ggaaaaggaggcgccaagcgtcatcgcaaggtg<br>ctccgagacaacatccaggggatcacaaagccc<br>gccattcgtcgtctggctcgccgtggtggcgtc<br>aagcgtatctccggtctgatctacgaggagacc<br>cgaggcgtgctcaaagtcttcctggagaacgtg<br>atccgcgacgccgtcacctacacggaacacgcc<br>aagagaaaaacggtcaccgccatggacgtcgtc<br>tacgcgctgaagagacaaggacgtaccctgtac<br>gggttcggcggctag | MSGRGKGGKGLGK<br>GGAKRHRKVLRDN<br>IQGITKPAIRRLA<br>RRGGVKRISGLIY<br>EETRGVLKVFLEN<br>VIRDAVTYTEHAK<br>RKTVTAMDVVYAL<br>KRQGRTLYGFGG |
| Q4PM69<br>Histone H4 | >Primer sequences: 5′ – 3′<br>Forward (F) and Reverse (R)<br>F: CTCGAGATGTCTGGCCGTGGCAAAG<br>R: GAATTCGCCGCCGAACCCGTACAGGG | |
| A0A131YLU7<br>Aminopeptidase N | >Peptidase M domain<br>atggtggattattcgttatcaattggtccaaag<br>atcttggaattctacgagaagtacttctcggaa<br>aagtatccactcccaaagactgacatggtggca<br>ctgccggactttaatgctggcgccatggagaat<br>tggggactggtcaccttccgagaaacggctcta<br>ctcttcaacccgaacgagtcgtcggccggaaac<br>aagcagcgcgtcgccgtcgtcgtctcacatgaa<br>ctcgcacatcagtggtttggaaacttagttacc<br>atggagtggtgggacgacctgtggctcaatgaa<br>ggcttcgccacctacgttgagtatctgggcgtg<br>gatttcgttcacaaggactgggagatggcacaa<br>cagttcatcggcgaagaactgcagccagtgatg<br>gagctcgactccctcaagtcgtcgcaccctgtc<br>tcggttccagtctacaaccctgacgaaatcatt<br>gaaaactttgacaagatatcttacggaaagggg<br>gcatccataattcgtatgatgaacttcttcctc<br>acagaaccggtcttcaggaaaggtgtctcgact<br>tacctgaagaaacgctctttcaacaacgctcgc<br>caggatgatctttgggcggaactcaccatggct<br>caaaatgaaagcaaccgcgtggacgtcaagact<br>gtgatggattcgtggactctc | MVDYSLSIGPKIL<br>EFYEKYFSEKYPL<br>PKTDMVALPDFNA<br>GAMENWGLVTFRE<br>TALLFNPNESSAG<br>NKQRVAVVVSHEL<br>AHQWFGNLVTMEW<br>WDDLWLNEGFATY<br>VEYLGVDFVHKDW<br>EMAQQFIGEELQP<br>VMELDSLKSSHPV<br>SVPVYNPDEIIEN<br>FDKISYGKGASII<br>RMMNFFLTEPVFR<br>KGVSTYLKKRSFN<br>NARQDDLWAELTM<br>AQNESNRVDVKTV<br>MDSWTL |
| A0A131YLU7<br>Aminopeptidase N | >Primer sequences: 5′ – 3′<br>Forward (F) and Reverse (R)<br>F: CTCGAGATGGTGGATTATTCGTTA<br>R: GAATTCGAGAGTCCACGAATCCAT | |
| A0A224YEQ4<br>Glycine-rich superfamily member | >Optimized sequence<br>atgtatccgggtagcttcggtggcgtgctgggt<br>cgtctgagcggcagctatccgggtagcctgggt<br>ggcgttctgggtggcctgtacggcagctatccg<br>ggtcgtttcggtggcgcgctgagcggtctgtat<br>ggcagctatccgggtagctttggtggctacctg<br>ggtggcctgagcggttacatgtatccgggcagc<br>tttggtggcgtgctgggccgtctgagcggtagc<br>tatccgggcagcctgggtggcgttctgggtggc<br>ctgtatggtagctatccgggccgtttcggtggc<br>gcgctgtctggcttatatggcagctatccgggt | MYPGSFGGVLGRL<br>SGSYPGSLGGVLG<br>GLYGSYPGRFGGA<br>LSGLYGSYPGSFG<br>GYLGGLSGYMYPG<br>SFGGVLGRLSGSY<br>PGSLGGVLGGLYG<br>SYPGRFGGALSGL<br>YGSYPGSFGGYLG<br>GLSGYMYPGSFGG<br>VLGRLSGSYPGSL |

| | | |
|---|---|---|
| | agctttggtggctacctgggtggcctgtctggc<br>tatatgtatccgggcagcttcggtggcgtgtta<br>ggtcgtctgtctggcagctatccgggtagcctg<br>ggtggcgttctgggtggcctgtacggcagctat<br>ccgggtcgtttcggtggcgcgctgagtggcctt<br>tatggcagctatccgggtagctttggtggctac<br>ctgggtggcctgagtggctacatgtatccgggc<br>agctttggtggcgtgttaggccgtctgtctggt<br>agctatccgggcagcctgggtggcgttctgggt<br>ggcctgtacggtagctatccgggccgtttcggt<br>ggcgcgctgagtgggttgtatggcagctatccg<br>ggtagctttggtggctacctgggtggcctgagt<br>gggtacatgtatccgggcagcttcggtggcgtg<br>cttggtcgtctgtccggcagctatccgggtagc<br>ctgggtggcgttctgggtggcctgtacggcagc<br>tatccgggtcgtttcggtggcgcgctgagtggt<br>ttatatggcagctatccgggtagctttggtggc<br>tacctgggtggcctgagtggttatatgtatccg<br>ggcagctttggtggcgtgctaggccgtctgagt<br>ggtagctatccgggtagcctgggtggtgtgctg<br>ggtggcctgtacggtagctatccgggccgtttc<br>ggtggcgcgctgagtggactctatggcagctat<br>ccgggtagctttggtggctacctgggtggcctg<br>agtggatacatgtatccgggtagcttcggtggc<br>gtgttaggtcgtctgagtggcagctatccgggt<br>agcctgggtggtgtgctgggtggcctgtacggc<br>agctatccgggtcgtttcggtggcgcgctgagt<br>gggctatatggcagctatccgggtagctttggt<br>ggctacctgggtggcctgagtgggtacatgtat<br>ccgggtagctttggtggcgtgttgggccgtctg<br>tctggtagctatccgggtagcctgggtggtgtg<br>ctgggtggcctgtacggtagctatccgggccgt<br>ttcggtggcgcgctgagtgggctgtatggcagc<br>tatccgggtagctttggtggctacctgggtggc<br>ctgagtgggtacatgtatccgggtagcttcggt<br>ggcgtgctaggtcgtctgagcggcagctatccg<br>ggtagcctgggtggtgtgctgggtggcctgtac<br>ggcagctatccgggtcgtttcggtggcgcgctg<br>agtgggctttatggcagctatccgggtagcttt<br>ggtggctacctgggtggcctgagtgggtacatg<br>tatccgggtagcttcggtggcgtgctcggccgt<br>ctgtccggtagctatccgggtagcctgggtggt<br>gtgctgggtggcctgtacggtagctatccgggc<br>cgtttcggtggcgcgctgagtgggctctatggc<br>agctatccgggtagctttggtggctacctgggt<br>ggcctgagtgggtac | GGVLGGLYGSYPG<br>RFGGALSGLYGSY<br>PGSFGGYLGGLSG<br>YMYPGSFGGVLGR<br>LSGSYPGSLGGVL<br>GGLYGSYPGRFGG<br>ALSGLYGSYPGSF<br>GGYLGGLSGYMYP<br>GSFGGVLGRLSGS<br>YPGSLGGVLGGLY<br>GSYPGRFGGALSG<br>LYGSYPGSFGGYL<br>GGLSGYMYPGSFG<br>GVLGRLSGSYPGS<br>LGGVLGGLYGSYP<br>GRFGGALSGLYGS<br>YPGSFGGYLGGLS<br>GYMYPGSFGGVLG<br>RLSGSYPGSLGGV<br>LGGLYGSYPGRFG<br>GALSGLYGSYPGS<br>FGGYLGGLSGYMY<br>PGSFGGVLGRLSG<br>SYPGSLGGVLGGL<br>YGSYPGRFGGALS<br>GLYGSYPGSFGGY<br>LGGLSGYMYPGSF<br>GGVLGRLSGSYPG<br>SLGGVLGGLYGSY<br>PGRFGGALSGLYG<br>SYPGSFGGYLGGL<br>SGYMYPGSFGGVL<br>GRLSGSYPGSLGG<br>VLGGLYGSYPGRF<br>GGALSGLYGSYPG<br>SFGGYLGGLSGY |
| A0A224YEQ4 Glycine-rich superfamily member | >Primer sequences: 5′ – 3′ Forward (F) and Reverse (R)<br>F: CACCATGTATCCGGGTAGCTTCGG<br>R: CCCACTCAGGCCACCCAGGT | |

**Table S5.** Examples of tick-derived proteins identified in this study in the cementome and previously characterized and reported in the sialome/sialotranscriptome.

| **Protein names** | **References** |
|---|---|
| Secreted protein 20 Hemelipoprotein HeLp/ HeLp 2 | Tirloni L, Reck J, Terra RM, Martins JR, Mulenga A, Sherman NE, Fox JW, Yates JR 3rd, Termignoni C, Pinto AF, Vaz Ida S Jr. Proteomic Analysis of Cattle Tick *Rhipicephalus* (*Boophilus*) *microplus* Saliva: A Comparison between Partially and Fully Engorged Females. PLoS One. 2014 Apr 24;9(4):e94831. |
| Puromycin-sensitive aminopeptidase | Ribeiro JM, Slovák M, Francischetti IM. An insight into the sialome of *Hyalomma excavatum.* Ticks Tick Borne Dis. 2017 Feb;8(2):201-207.<br>Ayllón N, Villar M, Galindo RC, Kocan KM, Šíma R, López JA, Vázquez J, Alberdi P, Cabezas-Cruz A, Kopáček P, de la Fuente J. Systems biology of tissue-specific response to *Anaplasma phagocytophilum* reveals differentiated apoptosis in the tick vector *Ixodes scapularis*. PLoS Genet. 2015 Mar 27;11(3):e1005120. |
| Tubulin beta chain | Karim S, Singh P, Ribeiro JM. A Deep Insight into the Sialotranscriptome of the Gulf Coast Tick, *Amblyomma maculatum.* PLoS One. 2011;6(12):e28525. |
| Myosin heavy chain 6/7<br>Glycine-rich superfamily member<br>Alpha-N-acetylgalactosaminidase<br>Transglutaminase/protease like protein | de Castro MH, de Klerk D, Pienaar R, Rees DJG, Mans BJ. Sialotranscriptomics of *Rhipicephalus zambeziensis* reveals intricate expression profiles of secretory proteins and suggests tight temporal transcriptional regulation during blood-feeding. Parasit Vectors. 2017 Aug 10;10(1):384.<br>Ayllón N, Villar M, Galindo RC, Kocan KM, Šíma R, López JA, Vázquez J, Alberdi P, Cabezas-Cruz A, Kopáček P, de la Fuente J. Systems biology of tissue-specific response to *Anaplasma phagocytophilum* reveals differentiated apoptosis in the tick vector *Ixodes scapularis*. PLoS Genet. 2015 Mar 27;11(3):e1005120. |
| Cuticular protein | Tan AW, Francischetti IM, Slovak M, Kini RM, Ribeiro JM. Sexual differences in the sialomes of the zebra tick, *Rhipicephalus pulchellus.* J Proteomics. 2015 Mar 18;117:120-44.<br>Ayllón N, Villar M, Galindo RC, Kocan KM, Šíma R, López JA, Vázquez J, Alberdi P, Cabezas-Cruz A, Kopáček P, de la Fuente J. Systems biology of tissue-specific response to *Anaplasma phagocytophilum* reveals differentiated apoptosis in the tick vector *Ixodes scapularis*. PLoS Genet. 2015 Mar 27;11(3):e1005120. |
| Aminopeptidase N | de Castro MH, de Klerk D, Pienaar R, Latif AA, Rees DJ, Mans BJ. De novo assembly and annotation of the salivary gland transcriptome of *Rhipicephalus appendiculatus* male and female ticks during blood feeding. Ticks Tick Borne Dis. 2016 Jun;7(4):536-48.<br>Ayllón N, Villar M, Galindo RC, Kocan KM, Šíma R, López JA, Vázquez J, Alberdi P, Cabezas-Cruz A, Kopáček P, de la Fuente J. Systems biology of tissue-specific response to *Anaplasma phagocytophilum* reveals differentiated apoptosis in the tick vector *Ixodes scapularis*. PLoS Genet. 2015 Mar 27;11(3):e1005120. |

| | |
|---|---|
| Aminopeptidase<br>Glycine-rich superfamily member<br>Myosin | Lewis LA, Radulović ŽM, Kim TK, Porter LM, Mulenga A. Identification of 24 h *Ixodes scapularis* immunogenic tick saliva proteins. Ticks Tick Borne Dis. 2015 Apr;6(3):424-34.<br>de Castro MH, de Klerk D, Pienaar R, Latif AA, Rees DJ, Mans BJ. De novo assembly and annotation of the salivary gland transcriptome of *Rhipicephalus appendiculatus* male and female ticks during blood feeding. Ticks Tick Borne Dis. 2016 Jun;7(4):536-48.<br>Esteves E, Maruyama SR, Kawahara R, Fujita A, Martins LA, Righi AA, Costa FB, Palmisano G, Labruna MB, Sá-Nunes A, Ribeiro JMC, Fogaça AC. Analysis of the Salivary Gland Transcriptome of Unfed and Partially Fed *Amblyomma sculptum* Ticks and Descriptive Proteome of the Saliva. Front Cell Infect Microbiol. 2017 Nov 21;7:476.<br>Ayllón N, Villar M, Galindo RC, Kocan KM, Šíma R, López JA, Vázquez J, Alberdi P, Cabezas-Cruz A, Kopáček P, de la Fuente J. Systems biology of tissue-specific response to *Anaplasma phagocytophilum* reveals differentiated apoptosis in the tick vector *Ixodes scapularis*. PLoS Genet. 2015 Mar 27;11(3):e1005120.<br>Bullard R, Sharma SR, Das PK, Morgan SE, Karim S. Repurposing of Glycine-rich proteins in abiotic and biotic stresses in the Lone-Star tick (*Amblyomma americanum*). Front Physiol. 2019 Jun 18;10:744.<br>Kim TK, Tirloni L, Pinto AFM, Diedrich JK, Moresco JJ, Yates JR 3rd, da Silva Vaz I Jr, Mulenga A. Time-resolved proteomic profile of *Amblyomma americanum* tick saliva during feeding. PLoS Negl Trop Dis. 2020;14(2):e0007758. |
| Salivary gland metalloprotease Reprolysin | Barnard AC, Nijhof AM, Gaspar AR, Neitz AW, Jongejan F, Maritz-Olivier C. Expression profiling, gene silencing and transcriptional networking of metzincin metalloproteases in the cattle tick, *Rhipicephalus (Boophilus) microplus*. Vet Parasitol. 2012 May 25;186(3-4):403-14. Anatriello E, Ribeiro JM, de Miranda-Santos IK, Brandão LG, Anderson JM, Valenzuela JG, Maruyama SR, Silva JS, Ferreira BR. An insight into the sialotranscriptome of the brown dog tick, *Rhipicephalus sanguineus*. BMC Genomics. 2010 Jul 22;11:450.<br>Ayllón N, Villar M, Galindo RC, Kocan KM, Šíma R, López JA, Vázquez J, Alberdi P, Cabezas-Cruz A, Kopáček P, de la Fuente J. Systems biology of tissue-specific response to *Anaplasma phagocytophilum* reveals differentiated apoptosis in the tick vector *Ixodes scapularis*. PLoS Genet. 2015 Mar 27;11(3):e1005120. |
| Lipocalin | Tirloni L, Kim TK, Pinto AFM, Yates JR 3rd, da Silva Vaz |

| | |
|---|---|
| | I Jr, Mulenga A. Tick-Host Range Adaptation: Changes in Protein Profiles in Unfed Adult *Ixodes scapularis* and *Amblyomma americanum* Saliva Stimulated to Feed on Different Hosts. Front Cell Infect Microbiol. 2017 Dec 19;7:517.<br>Esteves E, Maruyama SR, Kawahara R, Fujita A, Martins LA, Righi AA, Costa FB, Palmisano G, Labruna MB, Sá-Nunes A, Ribeiro JMC, Fogaça AC. Analysis of the Salivary Gland Transcriptome of Unfed and Partially Fed *Amblyomma sculptum* Ticks and Descriptive Proteome of the Saliva. Front Cell Infect Microbiol. 2017 Nov 21;7:476.<br>Manzano-Román R, Díaz-Martín V, Oleaga A, Obolo-Mvoulouga P, Pérez-Sánchez R. TSGP4 from *Ornithodoros moubata*: molecular cloning, phylogenetic analysis and vaccine efficacy of a new member of the lipocalin clade of cysteinyl leukotriene scavengers. Vet Parasitol. 2016 Aug 30;227:130-7.<br>Neelakanta G, Sultana H, Sonenshine DE, Andersen JF. Identification and characterization of a histamine-binding lipocalin-like molecule from the relapsing fever tick *Ornithodoros turicata*. Insect Mol Biol. 2018 Apr;27(2):177-187.<br>de Castro MH, de Klerk D, Pienaar R, Latif AA, Rees DJ, Mans BJ. De novo assembly and annotation of the salivary gland transcriptome of *Rhipicephalus appendiculatus* male and female ticks during blood feeding. Ticks Tick Borne Dis. 2016 Jun;7(4):536-48.<br>Anatriello E, Ribeiro JM, de Miranda-Santos IK, Brandão LG, Anderson JM, Valenzuela JG, Maruyama SR, Silva JS, Ferreira BR. An insight into the sialotranscriptome of the brown dog tick, *Rhipicephalus sanguineus*. BMC Genomics. 2010 Jul 22;11:450.<br>Francischetti IM, My Pham V, Mans BJ, Andersen JF, Mather TN, Lane RS, Ribeiro JM. The transcriptome of the salivary glands of the female western black-legged tick *Ixodes pacificus* (Acari: Ixodidae). Insect Biochem Mol Biol. 2005 Oct;35(10):1142-61.<br>Ayllón N, Villar M, Galindo RC, Kocan KM, Šíma R, López JA, Vázquez J, Alberdi P, Cabezas-Cruz A, Kopáček P, de la Fuente J. Systems biology of tissue-specific response to *Anaplasma phagocytophilum* reveals differentiated apoptosis in the tick vector *Ixodes scapularis*. PLoS Genet. 2015 Mar 27;11(3):e1005120.<br>Kim TK, Tirloni L, Pinto AFM, Diedrich JK, Moresco JJ, Yates JR 3rd, da Silva Vaz I Jr, Mulenga A. Time-resolved proteomic profile of *Amblyomma americanum* tick saliva during feeding. PLoS Negl Trop Dis. 2020;14(2):e0007758. |
| Pancreatic trypsin inhibitor | Tirloni L, Kim TK, Coutinho ML, Ali A, Seixas A, Termignoni C, Mulenga A, da Silva Vaz I Jr. The putative role of *Rhipicephalus microplus* salivary serpins in the tick- |

| | |
|---|---|
| | host relationship. Insect Biochem Mol Biol. 2016 Apr;71:12-28.<br>Zhang H, Qiao R, Gong H, Cao J, Zhou Y, Zhou J. Identification and anticoagulant activity of a novel Kunitz-type protein HA11 from the salivary gland of the tick *Hyalomma asiaticum*. Exp Appl Acarol. 2017 Jan;71(1):71-85. |
| Peptidoglycan recognition protein | Wang J, Song X, Wang M. Peptidoglycan recognition proteins in hematophagous arthropods. Dev Comp Immunol. 2018 Jun;83:89-95.<br>Shaw DK, Wang X, Brown LJ, Chávez AS, Reif KE, Smith AA, Scott AJ, McClure EE, Boradia VM, Hammond HL, Sundberg EJ, Snyder GA, Liu L, DePonte K, Villar M, Ueti MW, de la Fuente J, Ernst RK, Pal U, Fikrig E, Pedra JH. Infection-derived lipids elicit an immune deficiency circuit in arthropods. Nat Commun. 2017 Feb 14;8:14401. |
| Cytochrome P450 | Rosario-Cruz R, Almazan C, Miller RJ, Dominguez-Garcia DI, Hernandez-Ortiz R, de la Fuente J. Genetic basis and impact of tick acaricide resistance. Front Biosci (Landmark Ed). 2009 Jan 1;14:2657-65.<br>Ayllón N, Villar M, Galindo RC, Kocan KM, Šíma R, López JA, Vázquez J, Alberdi P, Cabezas-Cruz A, Kopáček P, de la Fuente J. Systems biology of tissue-specific response to *Anaplasma phagocytophilum* reveals differentiated apoptosis in the tick vector *Ixodes scapularis*. PLoS Genet. 2015 Mar 27;11(3):e1005120. |
| Angiotensin-converting enzyme | Jarmey JM, Riding GA, Pearson RD, McKenna RV, Willadsen P. Carboxydipeptidase from *Boophilus microplus*: a "concealed" antigen with similarity to angiotensin-converting enzyme. Insect Biochem Mol Biol. 1995 Oct;25(9):969-74.<br>Riding GA, Jarmey J, McKenna RV, Pearson R, Cobon GS, Willadsen P. A protective "concealed" antigen from *Boophilus microplus*. Purification, localization, and possible function. J Immunol. 1994 Dec 1;153(11):5158-66. |

**Table S6.** Published results about the potential role of selected tick cementome proteins for novel vaccine development.

| Protein name and ID | Results | Reference |
|---|---|---|
| Secreted protein 20 A0A034WXM6 | Potential vaccine antigen for the control of tick paralysis caused by *Ixodes holocyclus* | Masina et al., 1999 |
| Myosin heavy chain 6/7 A0A224YU86 | Protective response against tick, *Haemaphysalis qinghaiensis* | Gao et al., 2007 |
| Salivary gland metalloprotease Reprolysin Q45R50 A0A131ZAV6 | Protective response against ticks, *Ixodes ricinus*, *Haemaphysalis longicornis* and *R. microplus* | Decrem et al., 2008 Imamura et al., 2009 Ali et al., 2015 |
| Alpha-N-acetylgalactosaminidase A0A224YTB6 | Characterization as a potential vaccine antigen in *Clonorchis sinensis* (human clonorchiasis) | Lee et al., 2012 |
| Cuticular protein L7M046 | Protective response against *Haemonchus contortus* (human and animal haemonchosis) and *Wuchereria bancrofti* (human lymphatic filariasis) | Boisvenue et al., 1991 Arunkumar et al., 2014 |
| Aminopeptidase N A0A131YLU7 | Protective response as a mosquito-based vaccine against malaria | Mathias et al., 2012 |
| Transglutaminase/protease like protein A0A224Z8F0 | Protective response against human lymphatic filariasis | Immanuel et al., 2017 |
| Lipocalins A0A224YMC8 A0A224YB96 L7LTA8 | Protective response against tick, *Ixodes ricinus* and *A. phagocytophilum* infection | Contreras et al., 2017 |
| Angiotensin-converting enzyme A0A224YTI7 | Protective response against tick, *R. microplus* | Willadsen et al., 1996 Lambertz et al., 2012 |
| Tubulin beta chain G3MKF8 | Protective response against *Trypanosoma evansi* challenge | Tewari et al., 2015 |

Data refers to selected tick cementome proteins identified in this study (Table S1). Results have not been reported for histones H2A, H3, H4 (V5HZA1, V5HJX3, L7LVH9, Q4PM69), hemelipoproteins HeLp and HeLp 2 (A0A034WXN6, A0A034WZ42), Glycine-rich superfamily members (A0A224YEQ4, A0A224YEB4, A0A034WZ79, L7MDN1, A0A224YCP1, L7M1K6, A0A131YF72, A0A034WWS7), putative ubiquitin/40s ribosomal protein s27a fusion (A0A0K8RP30), puromycin-sensitive aminopeptidase (A0A131XKB4), pancreatic trypsin inhibitors (A0A224Y271, A0A131YNG2, A0A224Y1X4), putative bilaris (L7LRL1, L7ME64), gluzincin (A0A224YD77), peptidoglycan recognition proteins (A0A0B4PMF7, A0A131YG55), Cytochrome P450 (B7Q181), and putative animal peptidoglycan recognition protein logous to bacteriophage t3 lysozyme (L7M9R2).

**References included in Table S6**


Ali A, Parizi LF, Guizzo MG, Tirloni L, Seixas A, Vaz Ida S Jr, Termignoni C. Immunoprotective potential of a *Rhipicephalus (Boophilus) microplus* metalloprotease. Vet Parasitol. 2015 Jan 15;207(1-2):107-14. doi: 10.1016/j.vetpar.2014.11.007

Arunkumar C, Pandiaraja P, Prince PR, Kaliraj P. Immunological characterization of recombinant *Wuchereria bancrofti* cuticular collagen (COL-4) as putative vaccine candidate for human lymphatic filariasis. Asian Pac J Trop Med. 2014 Jul;7(7):505-12. doi: 10.1016/S1995-7645(14)60084-5

Boisvenue RJ, Stiff MI, Tonkinson LV, Cox GN. Protective studies in sheep immunized with cuticular collagen proteins and peptides of *Haemonchus contortus*. Parasite Immunol. 1991 May;13(3):227-40.

Contreras, M., Alberdi, P., Fernández de Mera, I.G., Krull, C., Nijhof, A., Villar, M., de la Fuente, J. 2017. Vaccinomics approach to the identification of candidate protective antigens for the control of tick vector infestations and *Anaplasma phagocytophilum* infection. Frontiers in Cellular and Infection Microbiology 7: 360.

Decrem Y, Mariller M, Lahaye K, Blasioli V, Beaufays J, Zouaoui Boudjeltia K, Vanhaeverbeek M, Cérutti M, Brossard M, Vanhamme L, Godfroid E. The impact of gene knock-down and vaccination against salivary metalloproteases on blood feeding and egg laying by *Ixodes ricinus*. Int J Parasitol. 2008 Apr;38(5):549-60.

Gao J, Luo J, Fan R, Guan G, Ren Q, Ma M, Sugimoto C, Bai Q, Yin H. Molecular characterization of a myosin alkali light chain-like protein, a "concealed" antigen from the hard tick *Haemaphysalis qinghaiensis*. Vet Parasitol. 2007 Jun 20;147(1-2):140-9.

Imamura S, da Silva Vaz I Jr, Konnai S, Yamada S, Nakajima C, Onuma M, Ohashi K. Effect of vaccination with a recombinant metalloprotease from *Haemaphysalis longicornis*. Exp Appl Acarol. 2009 Aug;48(4):345-58. doi: 10.1007/s10493-009-9245-3

Immanuel C, Ramanathan A, Balasubramaniyan M, Khatri VK, Amdare NP, Rao DN, Reddy MVR, Perumal K. Immunoprophylaxis of multi-antigen peptide (MAP) vaccine for human lymphatic filariasis. Immunol Res. 2017 Jun;65(3):729-738. doi: 10.1007/s12026-017-8911-5

Lambertz C, Chongkasikit N, Jittapalapong S, Gauly M. Immune Response of Bos indicus Cattle against the Anti-Tick Antigen Bm91 Derived from Local *Rhipicephalus (Boophilus) microplus* Ticks and Its Effect on Tick Reproduction under Natural Infestation. J Parasitol Res. 2012;2012:907607. doi: 10.1155/2012/907607

Lee MR, Yoo WG, Kim YJ, Kim DW, Cho SH, Hwang KY, Ju JW, Lee WJ. Molecular characterization of an α-N-acetylgalactosaminidase from *Clonorchis sinensis*. Parasitol Res. 2012 Nov;111(5):2149-56. doi: 10.1007/s00436-012-3063-y. Erratum in: Parasitol Res. 2012 Dec;111(6):2483.

Masina S, Broady KW. Tick paralysis: development of a vaccine. Int J Parasitol. 1999 Apr;29(4):535-41.

Mathias DK, Plieskatt JL, Armistead JS, Bethony JM, Abdul-Majid KB, McMillan A, Angov E, Aryee MJ, Zhan B, Gillespie P, Keegan B, Jariwala AR, Rezende W, Bottazzi ME, Scorpio DG, Hotez PJ, Dinglasan RR. Expression, Immunogenicity, Histopathology, and Potency of a Mosquito-Based Malaria Transmission-Blocking Recombinant Vaccine. Infect Immun. 2012 Apr;80(4):1606-14. doi: 10.1128/IAI.06212-11

Tewari AK, Kurup SP, Baidya S, Barta JR, Sharma B. Protective antibody and cytokine responses in mice following immunization with recombinant beta-tubulin and subsequent *Trypanosoma evansi* challenge. Parasit Vectors. 2015 Nov 9;8:580. doi: 10.1186/s13071-015-1189-3

Willadsen P, Smith D, Cobon G, McKenna RV. Comparative vaccination of cattle against *Boophilus microplus* with recombinant antigen Bm86 alone or in combination with recombinant Bm91. Parasite Immunol. 1996 May;18(5):241-6.

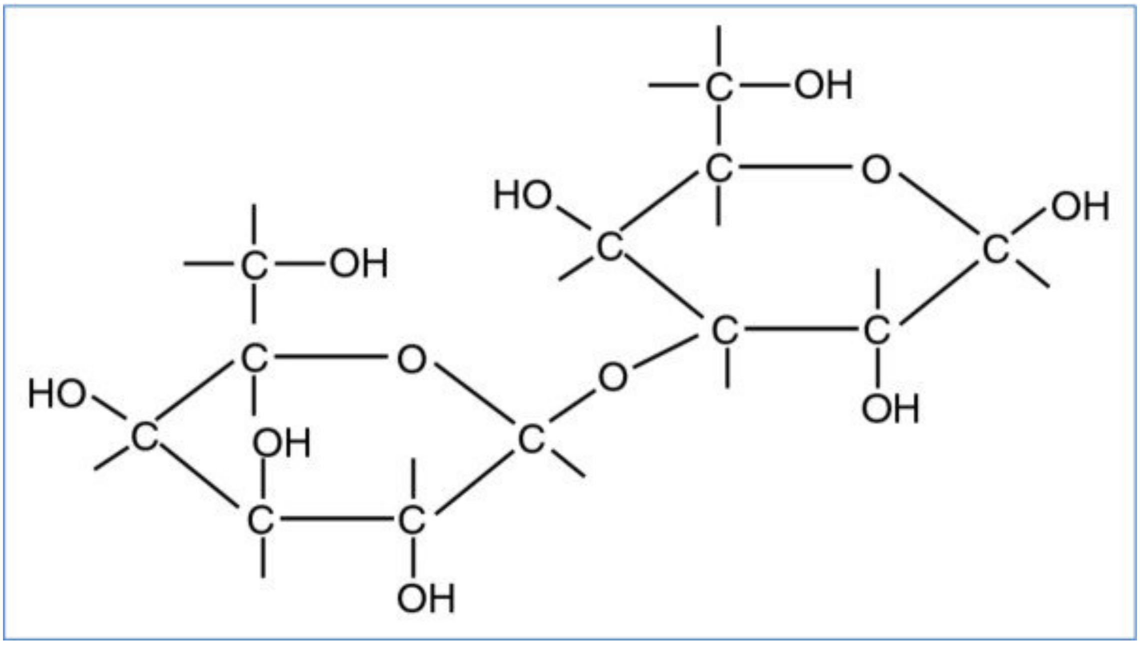


| Atomic composition | |
|---|---|
| Carbon C | 38.7% |
| Oxygen O | 35.5% |
| Hydrogen H | 25.8% |

**Figure S1.** Chemical structure and atomic composition of Galα1-3Galβ1-(3)4GlcNAc-R (α-Gal; $C_{12}H_8O_{11}$).

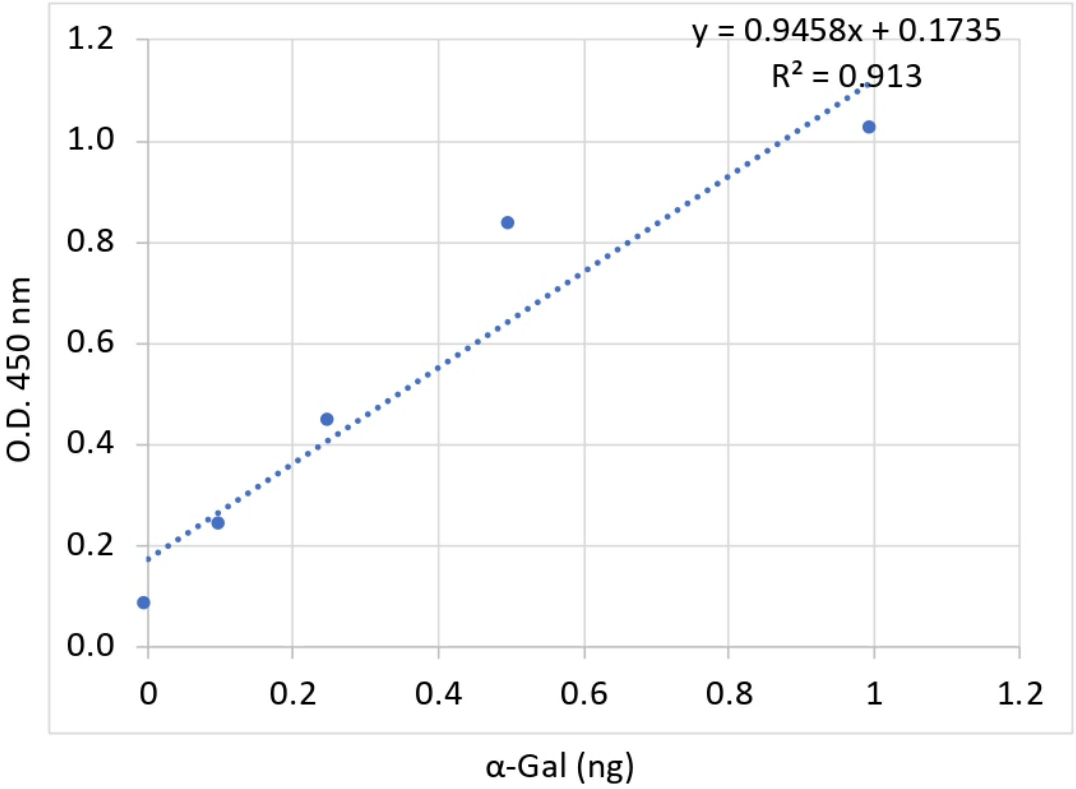


**Figure S2.** ELISA test for characterizing α-Gal levels. The α-Gal levels were determined by ELISA using BSA-α-Gal and *R. microplus* sialome and cementome proteins. The average value of the blanks (wells without sample proteins; N = 5) was subtracted from all reads and the average of 9 replicates for each sample was used for analysis. A calibration curve with 0.0 to 1.0 ng α-Gal and O.D. values at 450 nm was constructed using Microsoft Excel for Mac (v. 16.26) to convert ELISA reader values to α-Gal content per sample.

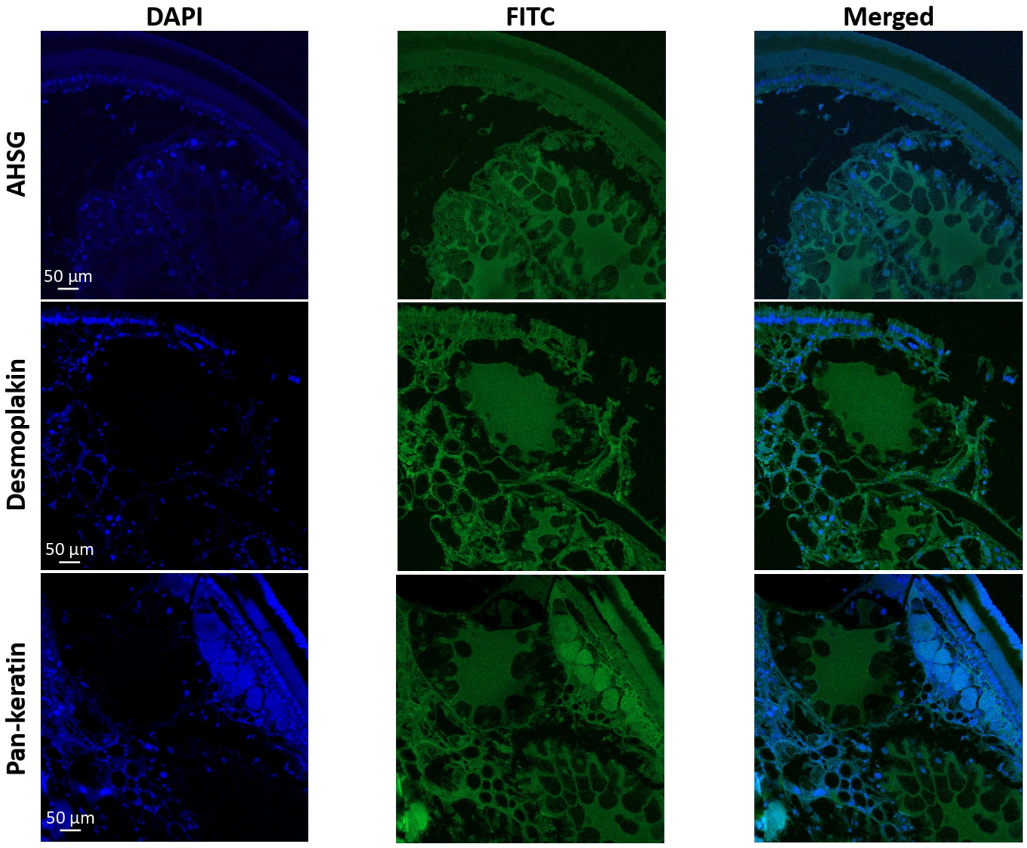


**Figure S3.** Validation of commercial antibodies against host proteins by immunofluorescence assay. Female *Ixodes scapularis* ticks fed on uninfected sheep and fixed with 4% paraformaldehyde in 0.2 M sodium cacodylate buffer were embedded in paraffin and used to prepare sections on glass slides as previously described (Ayllón et al., PLoS Genetics 2015;11: e1005120 and de la Fuente et al., Computational and Structural Biotechnology Journal 2020;18: 253-257). The paraffin was removed from the sections with xylene and then hydrated by successive 2 min washes with a graded series of 100, 95, 80, 75, and 50% ethanol. The slides were treated with Proteinase K (Dako, Barcelona, Spain) for 7 min, washed with PBS and incubated with 3% BSA (Sigma-Aldrich) in PBS for 1 h at RT. Then the slides were incubated with antibodies against desmoplakin (ab106342) and pan-keratin (ab190625) (Abcam, Cambridge, UK) diluted 1:100 in 3% BSA/PBS for 14 h at 4 °C. After additional washes in PBS, the sections were incubated with FITC conjugated goat anti-rabbit IgG secondary antibodies (Sigma-Aldrich), diluted 1:80 in 3% BSA/PBS, for 1 h at RT. Finally, the slides were mounted using Prolong Gold antifade reagent with DAPI reagent (Molecular Probes, Eugene, OR, USA). The slides were examined using a Zeiss LSM 800 laser scanning confocal microscope (Carl Zeiss, Oberkochen, Germany) with a 10x objective.

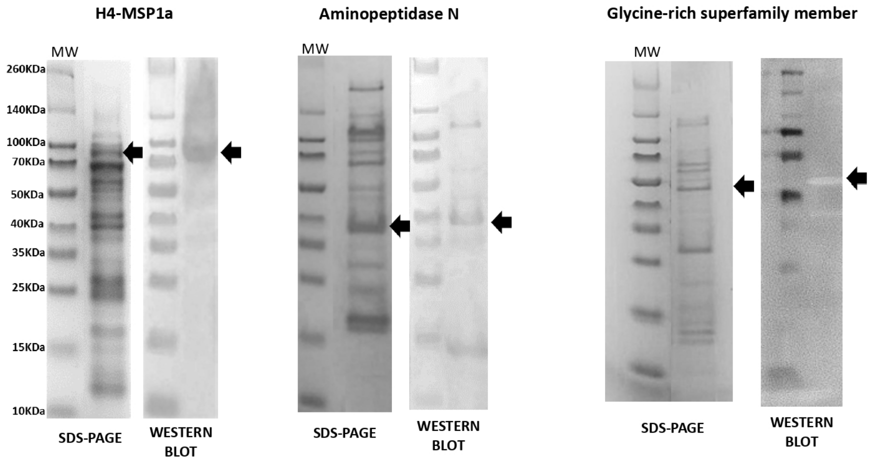


**Figure S4.** Western blot analysis of recombinant tick proteins. The recombinant histone H4-MSP1a, aminopeptidase N, and Glycine-rich superfamily member proteins were produced in *E. coli*. Samples were taken after purification. Ten µg proteins were loaded per well in an SDS-12% polyacrylamide gel. The gel was stained with Coomassie Brilliant Blue. The position of the recombinant proteins is indicated with arrows. For Western blot analysis, recombinant proteins were separated by electrophoresis and transferred to a nitrocellulose membrane. The membrane was incubated with pooled sera collected from histone H4, aminopeptidase N vaccinated rabbits and commercial rabbit anti-Glycine-rich superfamily member protein antibodies (Abcam). The position of the recombinant proteins in the Western blot is indicated with arrows. Abbreviation: MW, molecular weight markers (Spectra multicolor broad range protein ladder; Thermo Fisher Scientific).

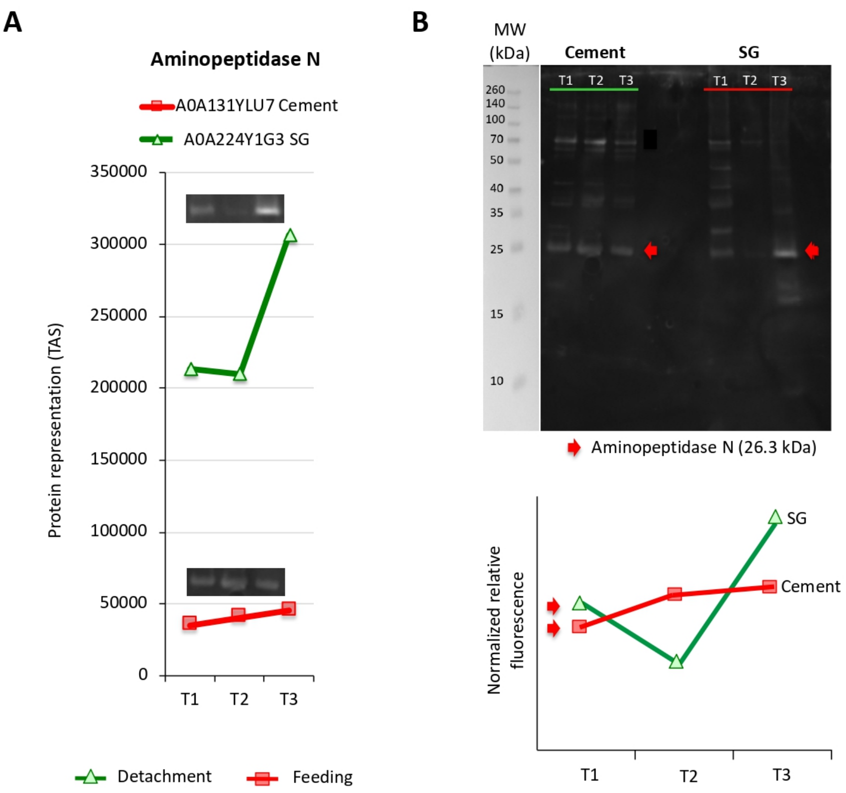


**Figure S5.** Western blot analysis of tick-derived aminopeptidase N protein in the *R. microplus* cementome (Cement) and sialome (SG). (A) Based on proteomics representation profiles, proteins were putatively assigned to different categories of developmental processes (Figure 1). (B) Cement protein lysate (10 µg) and salivary gland protein lysate (20 µg) were methanol/chloroform precipitated, resuspended in Laemmli sample buffer and separated on an SDS-polyacrylamide precast gel (ClearPage Expedeon, VWR, Radnor, PA, USA). After electrophoresis, proteins were transferred to a nitrocellulose blotting membrane (GE Healthcare Dharmacon Inc., Lafayette, CO, USA), blocked with 3% BSA (Sigma-Aldrich) in TBS and incubated overnight at 4 °C with antibodies against tick recombinant aminopeptidase N, diluted 1:200 in 3% BSA/TBS. To detect the IgG antibodies bound to tick proteins, membranes were incubated with goat anti-rabbit IgG peroxidase antibody (Sigma-Aldrich) diluted 1:1000 in 3% BSA/TBS. Immunoreactive proteins were visualized with chemiluminescence with Pierce ECL Western Blotting Substrate (Thermo Fisher Scientific). Quantitative analysis was performed using the Fiji ImageJ (https://imagej.nih.gov/ij/download.html) to measure the intensity of the protein bands and after substation of the intensity of the PBS control to compare the results at different time points. The results of the Western blot reproduced those obtained with proteomics analysis.

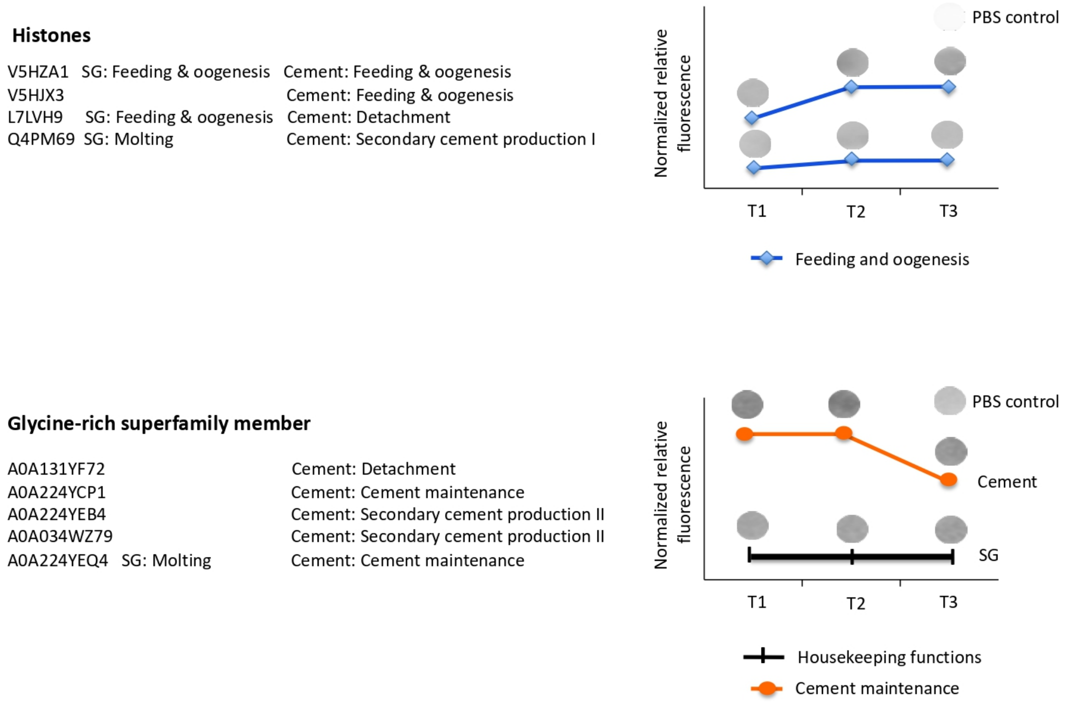


**Figure S6.** Dot blot analysis of tick-derived histones and Glycine-rich superfamily member proteins in the *R. microplus* cementome (Cement) and sialome (SG). Cement and salivary gland proteins were applied onto each strip in 6 different dots (volume per dot, 25 µl at 5 µg/µl. Then the dots were allowed to dry by gravity flow, immersed in 50 µl of 3% BSA/TBS for 15 min approximately and filtered by gravity. Membranes were washed two times with TBS and incubated with primary antibodies diluted at different concentrations in 1% BSA/TBS against Histone H4 (1:100) and Glycine-rich superfamily member proteins (1:200) To detect the IgG antibodies bound to tick proteins, membranes were incubated with goat anti-rabbit IgG peroxidase antibody (Sigma-Aldrich) diluted 1:1000 in 1% BSA/TBS. Membranes were washed two times with TBS and immunoreactive proteins were visualized with chemiluminescence with Pierce ECL Western Blotting Substrate (Thermo Fisher Scientific). Quantitative analysis was performed using the Fiji ImageJ (https://imagej.nih.gov/ij/download.html) to measure the intensity of the protein bands and after substation of the intensity of the PBS control to compare the results at different time points. Based on proteomics representation profiles, proteins were putatively assigned to different categories of developmental processes (Figure 1). Because these proteins constitute a protein family they were assigned to multiple developmental processes. Accordingly, the results of the dot blot reproduced some of the profiles obtained with proteomics analysis.

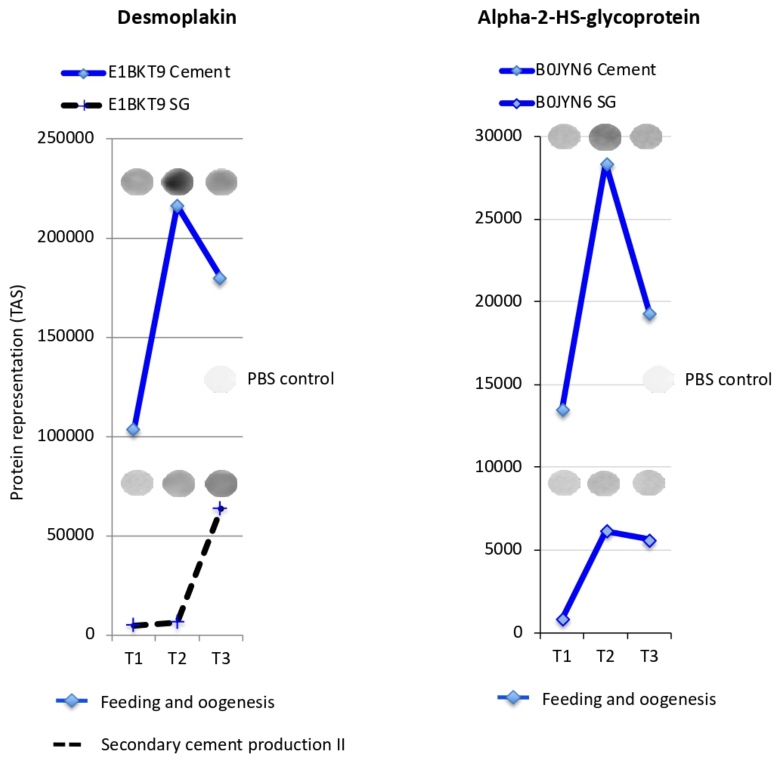


**Figure S7.** Dot blot analysis of host-derived desmoplakin and alpha-2-HS-glycoprotein in the *R. microplus* cementome (Cement) and sialome (SG). Cement and salivary gland proteins were applied onto each strip in 6 different dots (volume per dot, 25 µl at 5 µg/µl. Then the dots were allowed to dry by gravity flow, immersed in 50 µl of 3% BSA/TBS for 15 min approximately and filtered by gravity. Membranes were washed two times with TBS and incubated with primary antibodies diluted at different concentrations in 1% BSA/TBS against desmoplakin (1:300) and alpha-2-HS-glycoprotein (1:300). To detect the IgG antibodies bound to tick proteins, membranes were incubated with goat anti-rabbit IgG peroxidase antibody (Sigma-Aldrich) diluted 1:1000 in 1% BSA/TBS. Membranes were washed two times with TBS and immunoreactive proteins were visualized with chemiluminescence with Pierce ECL Western Blotting Substrate (Thermo Fisher Scientific). Quantitative analysis was performed using the Fiji ImageJ (https://imagej.nih.gov/ij/download.html) to measure the intensity of the protein bands and after substation of the intensity of the PBS control to compare the results at different time points. Based on proteomics representation profiles, proteins were putatively assigned to different categories of developmental processes (Figure 1). Because these proteins constitute a protein family they were assigned to multiple developmental processes. The results of the dot blot reproduced those obtained with proteomics analysis.

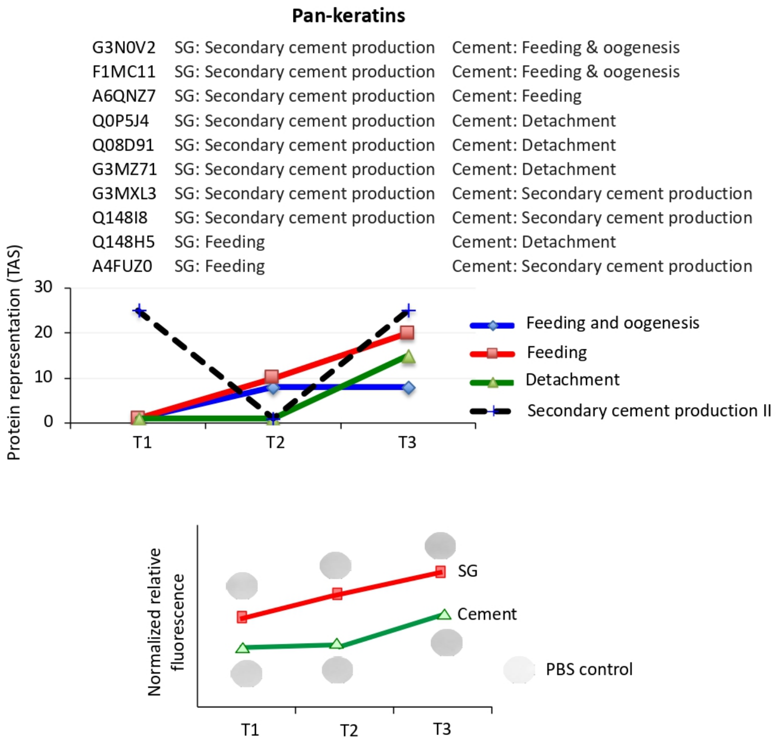


**Figure S8.** Dot blot analysis of host-derived pan-keratins in the *R. microplus* cementome (Cement) and sialome (SG). Cement and salivary gland proteins were applied onto each strip in 6 different dots (volume per dot, 25 µl at 5 µg/µl. Then the dots were allowed to dry by gravity flow, immersed in 50 µl of 3% BSA/TBS for 15 min approximately and filtered by gravity. Membranes were washed two times with TBS and incubated with primary antibodies diluted 1:300 in 1% BSA/TBS against pan-keratins. To detect the IgG antibodies bound to tick proteins, membranes were incubated with goat anti-rabbit IgG peroxidase antibody (Sigma-Aldrich) diluted 1:1000 in 1% BSA/TBS. Membranes were washed two times with TBS and immunoreactive proteins were visualized with chemiluminescence with Pierce ECL Western Blotting Substrate (Thermo Fisher Scientific). Quantitative analysis was performed using the Fiji ImageJ (https://imagej.nih.gov/ij/download.html) to measure the intensity of the protein bands and after substation of the intensity of the PBS control to compare the results at different time points. Based on proteomics representation profiles, proteins were putatively assigned to different categories of developmental processes (Figure 1). Because these proteins constitute a protein family they were assigned to multiple developmental processes. Accordingly, the results of the dot blot reproduced some of the profiles obtained with proteomics analysis.
