# Supplementary material for: Tick and Host Derived Compounds Detected in the Cement Complex Substance
Source: Biomolecules. 2020 Apr 5;10(4):555. doi: 10.3390/biom10040555 (PMC7226240; doi:10.3390/biom10040555)

Data S5. Composition of chemical elements in *R. microplus* tick cement and salivary glands.

The *R. microplus* tick cement and salivary gland samples were dehydrated in an incubator at 37 °C for 24 h in preparation for analysis with a scanning electron microscope (SEM). The samples were placed and mounted on standard aluminum SEM sample holders with conductive carbon adhesive tabs. The samples were observed and analyzed with a field emission scanning electron microscope (Zeiss GeminiSEM 500, Oberkochen, Germany) operating in high vacuum mode at an acceleration voltage of 15 kV and without metal coating. Three area scans per sample were used using an 80 mm2 dispersive energy spectroscopy (EDS) detector (Oxford Instruments, Abingdon, United Kingdom).

29/11/2019 09:57:44
Project 1
Cemento Mexico T1
CM1 15 Kv

Electron Image 28

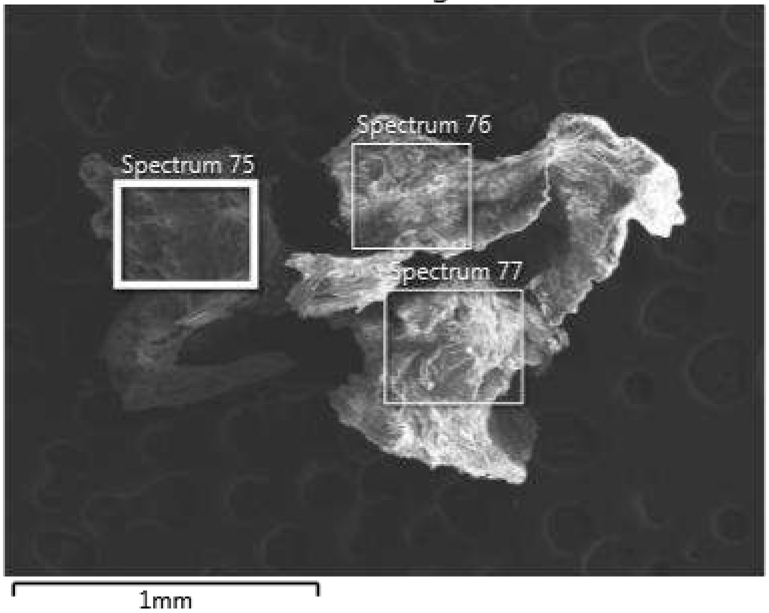

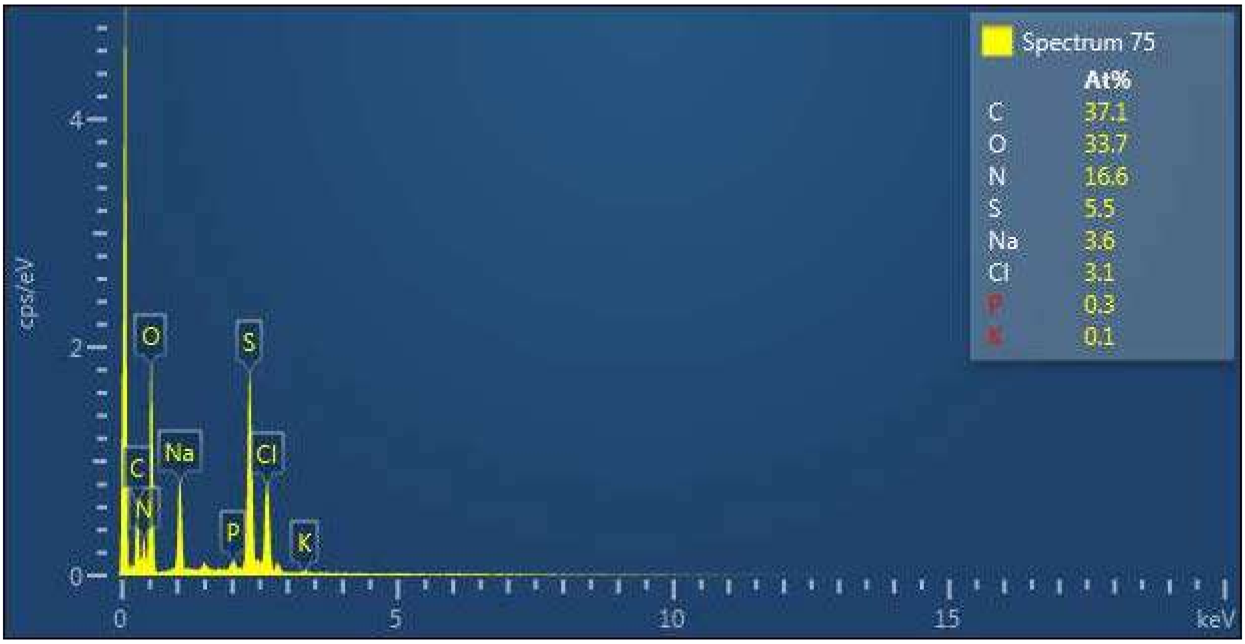

Label:	Spectrum 75
Source:	Acquired
Created:	29/11/2019 09:57:44
Livetime:	60.4s
Process Time:	4
Accelerating Voltage:	15.00kV
Magnification:	45 x
Working Distance:	9.9mm
Specimen Tilt (degrees):	0.0
Elevation (degrees):	35.0
Azimuth (degrees):	0.0
Number Of Channels:	2048
Energy Range (keV):	20 keV
Energy per Channel (eV):	10.0eV
Detector Type Id:	29
Detector Type:	X-Max
Window Type:	SATW
Pulse Pile Up Correction:	Succeeded
Primary Detector:	2617
Primary Detector Serial Number:	77871-X080

Element	Line Type	Apparent Concentration	k Ratio	Wt%	Wt% Sigma	Standard Label	Factory Standard	Standard Calibration Date
C	K series	1.15	0.01148	27.82	1.31	C Vit	Yes	
N	K series	3.09	0.00549	14.55	1.05	BN	Yes	
O	K series	5.33	0.01794	33.70	0.84	SiO2	Yes	
Na	K	1.43	0.00602	5.24	0.18	Albite	Yes	

	series							
P	K series	0.17	0.00094	0.49	0.07	GaP	Yes	
S	K series	2.62	0.02255	11.10	0.30	FeS2	Yes	
Cl	K series	1.46	0.01276	6.88	0.22	NaCl	Yes	
K	K series	0.05	0.00040	0.21	0.07	KBr	Yes	
Total:				100.00				

Electron Image 28

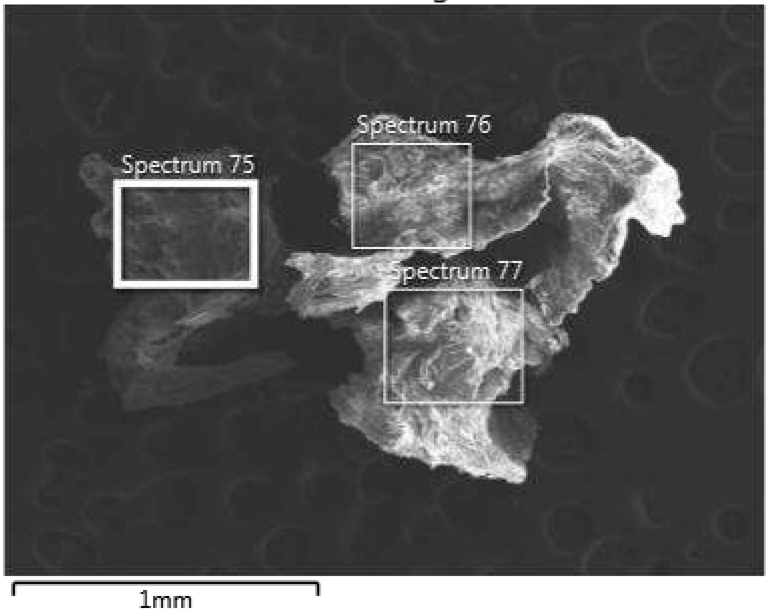

29/11/2019 09:58:53
Project 1
Cemento Mexico T1
CM1 15 Kv

Electron Image 28

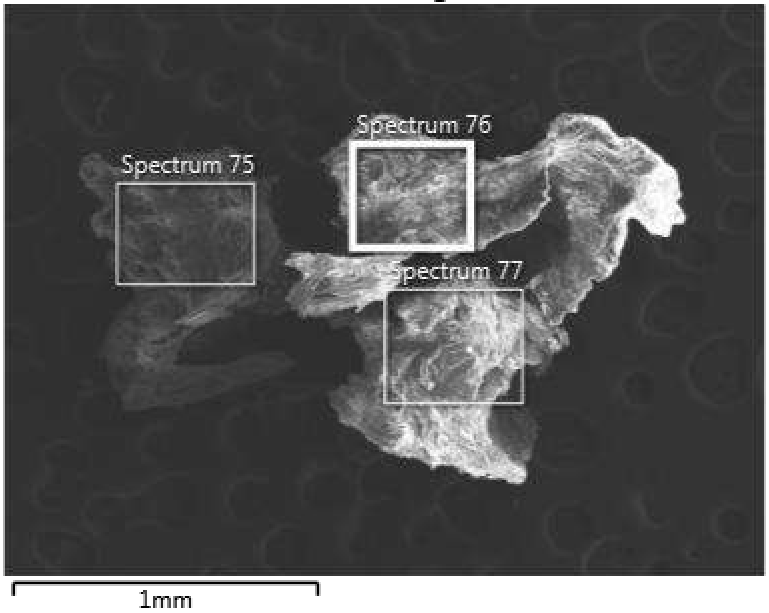

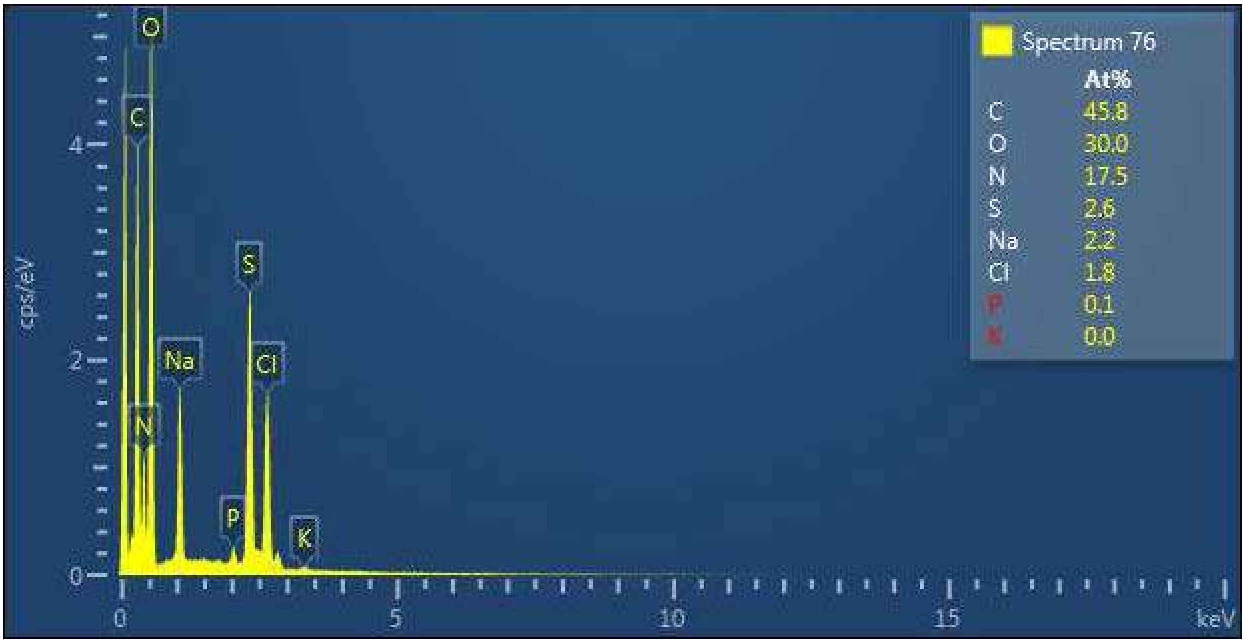

Label:	Spectrum 76
Source:	Acquired
Created:	29/11/2019 09:58:53
Livetime:	60.5s
Process Time:	4
Accelerating Voltage:	15.00kV
Magnification:	45 x
Working Distance:	9.9mm
Specimen Tilt (degrees):	0.0
Elevation (degrees):	35.0
Azimuth (degrees):	0.0
Number Of Channels:	2048
Energy Range (keV):	20 keV
Energy per Channel (eV):	10.0eV
Detector Type Id:	29
Detector Type:	X-Max
Window Type:	SATW
Pulse Pile Up Correction:	Succeeded
Primary Detector:	2617
Primary Detector Serial Number:	77871-X080

Element	Line Type	Apparent Concentration	k Ratio	Wt%	Wt% Sigma	Standard Label	Factory Standard	Standard Calibration Date
C	K series	7.45	0.07455	37.22	0.61	C Vit	Yes	
N	K series	10.58	0.01884	16.59	0.63	BN	Yes	
O	K series	14.92	0.05020	32.45	0.44	SiO2	Yes	
Na	K	2.83	0.01192	3.41	0.08	Albite	Yes	

	series							
P	K series	0.33	0.00182	0.31	0.03	GaP	Yes	
S	K series	4.03	0.03471	5.55	0.10	FeS2	Yes	
Cl	K series	2.96	0.02584	4.39	0.09	NaCl	Yes	
K	K series	0.06	0.00054	0.09	0.03	KBr	Yes	
Total:				100.00				

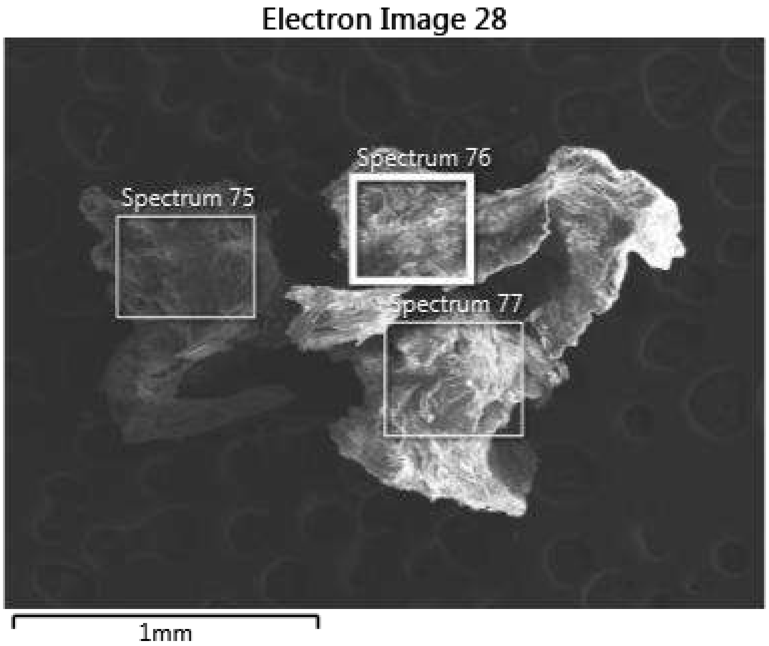

29/11/2019 10:00:04
Project 1
Cemento Mexico T1
CM1 15 Kv

Electron Image 28

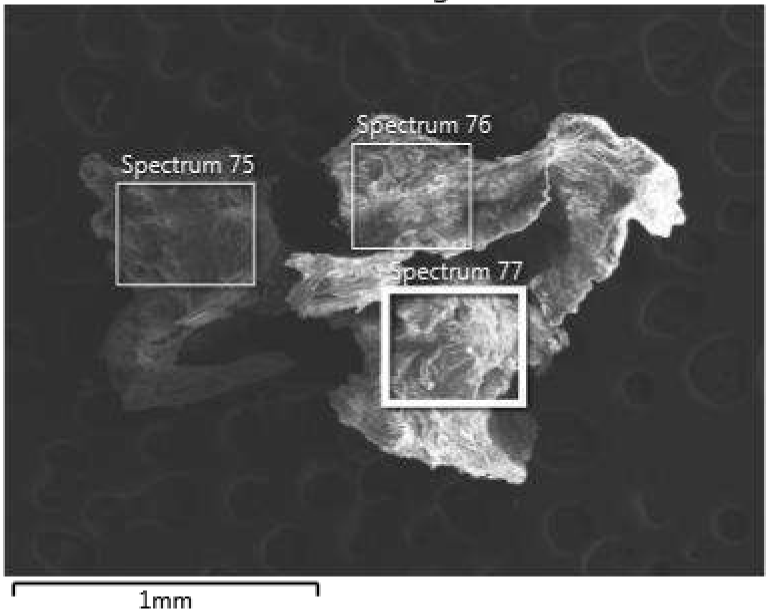

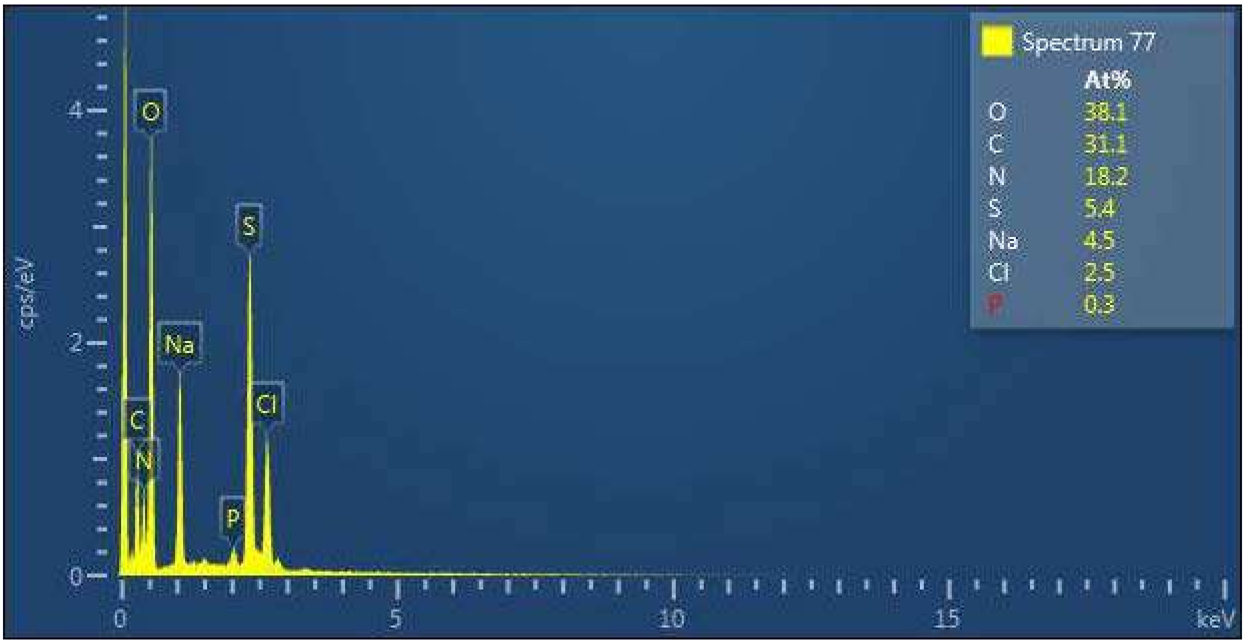

Label:	Spectrum 77
Source:	Acquired
Created:	29/11/2019 10:00:04
Livetime:	60.5s
Process Time:	4
Accelerating Voltage:	15.00kV
Magnification:	45 x
Working Distance:	9.9mm
Specimen Tilt (degrees):	0.0
Elevation (degrees):	35.0
Azimuth (degrees):	0.0
Number Of Channels:	2048
Energy Range (keV):	20 keV
Energy per Channel (eV):	10.0eV
Detector Type Id:	29
Detector Type:	X-Max
Window Type:	SATW
Pulse Pile Up Correction:	Succeeded
Primary Detector:	2617
Primary Detector Serial Number:	77871-X080

Element	Line Type	Apparent Concentration	k Ratio	Wt%	Wt% Sigma	Standard Label	Factory Standard	Standard Calibration Date
C	K series	1.72	0.01716	23.21	0.71	C Vit	Yes	
N	K series	6.59	0.01173	15.82	0.71	BN	Yes	
O	K series	10.92	0.03675	37.83	0.56	SiO2	Yes	
Na	K	2.89	0.01222	6.36	0.15	Albite	Yes	

	series							
P	K series	0.30	0.00167	0.51	0.05	GaP	Yes	
S	K series	4.26	0.03668	10.67	0.19	FeS2	Yes	
Cl	K series	2.02	0.01768	5.60	0.14	NaCl	Yes	
Total:				100.00				

Electron Image 28

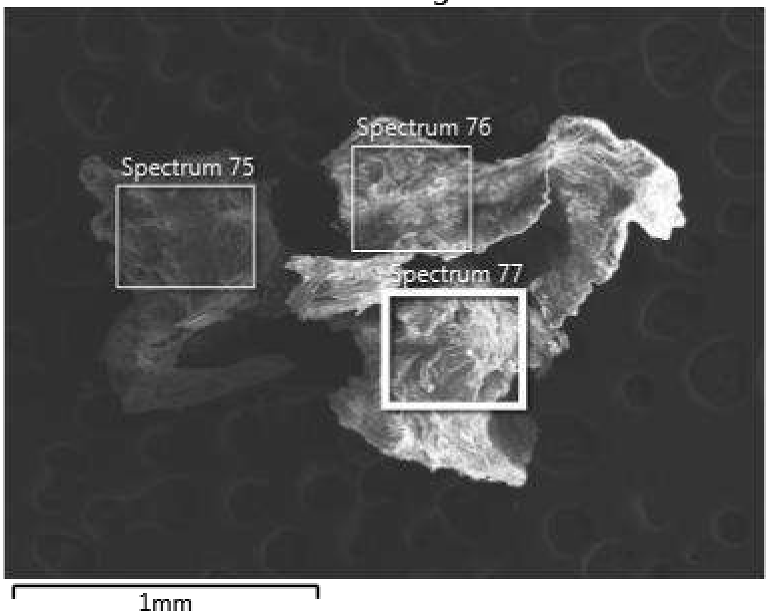

29/11/2019 10:03:41
Project 1
Cemento Mexico T1
CM2 15 Kv

Electron Image 29

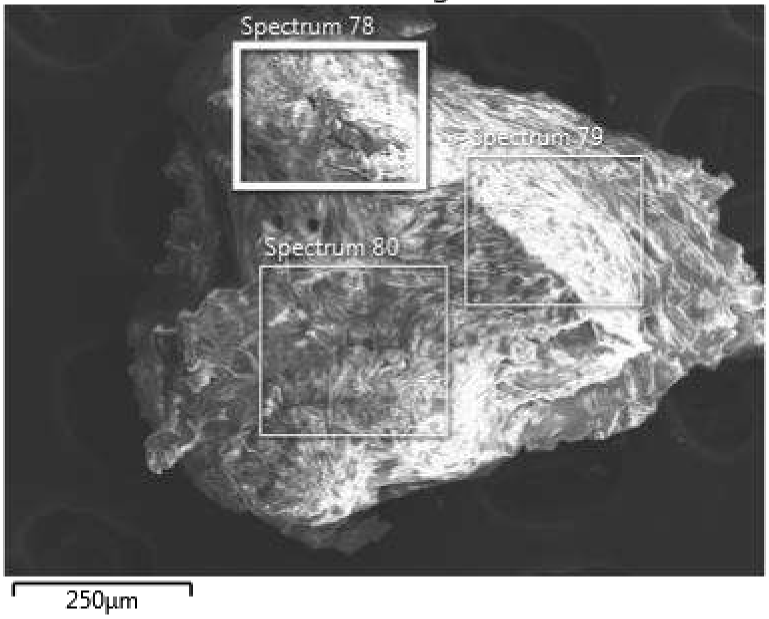

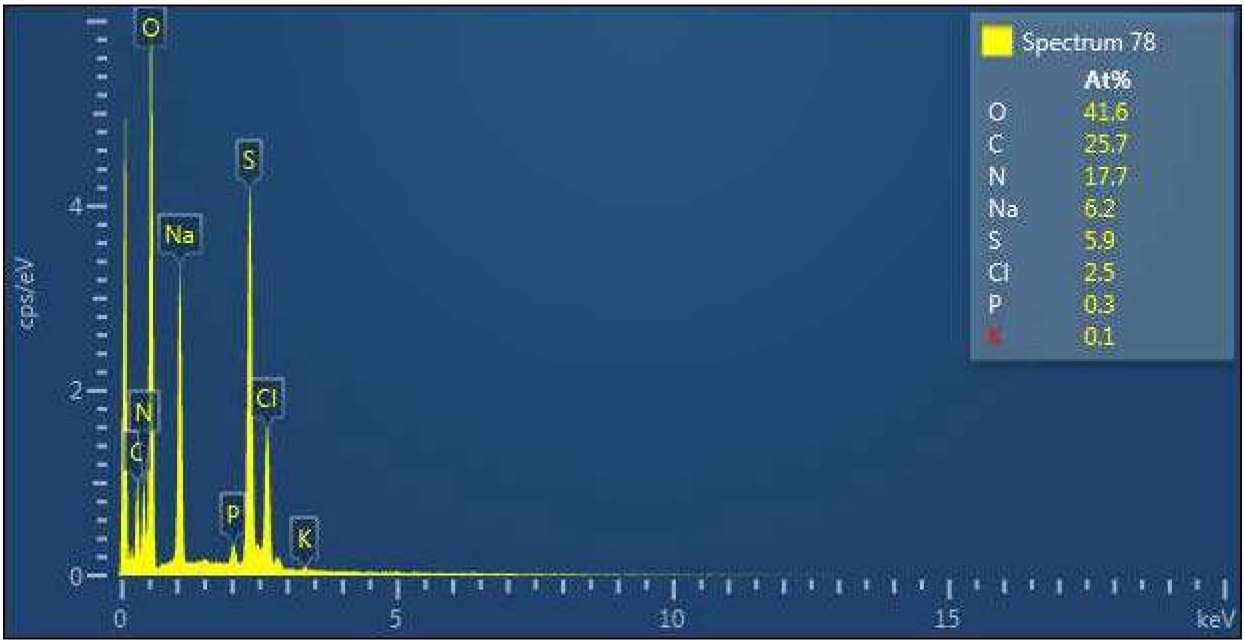

Label:	Spectrum 78
Source:	Acquired
Created:	29/11/2019 10:03:41
Livetime:	60.4s
Process Time:	4
Accelerating Voltage:	15.00kV
Magnification:	105 x
Working Distance:	9.9mm
Specimen Tilt (degrees):	0.0
Elevation (degrees):	35.0
Azimuth (degrees):	0.0
Number Of Channels:	2048
Energy Range (keV):	20 keV
Energy per Channel (eV):	10.0eV
Detector Type Id:	29
Detector Type:	X-Max
Window Type:	SATW
Pulse Pile Up Correction:	Succeeded
Primary Detector:	2617
Primary Detector Serial Number:	77871-X080

Element	Line Type	Apparent Concentration	k Ratio	Wt%	Wt% Sigma	Standard Label	Factory Standard	Standard Calibration Date
C	K series	1.84	0.01839	18.61	0.80	C Vit	Yes	
N	K series	9.56	0.01702	15.00	0.57	BN	Yes	
O	K series	17.52	0.05895	40.14	0.54	SiO2	Yes	
Na	K	5.55	0.02341	8.62	0.16	Albite	Yes	

	series							
P	K series	0.47	0.00264	0.58	0.05	GaP	Yes	
S	K series	6.42	0.05535	11.47	0.18	FeS2	Yes	
Cl	K series	2.75	0.02405	5.43	0.12	NaCl	Yes	
K	K series	0.08	0.00067	0.15	0.04	KBr	Yes	
Total:				100.00				

Electron Image 29

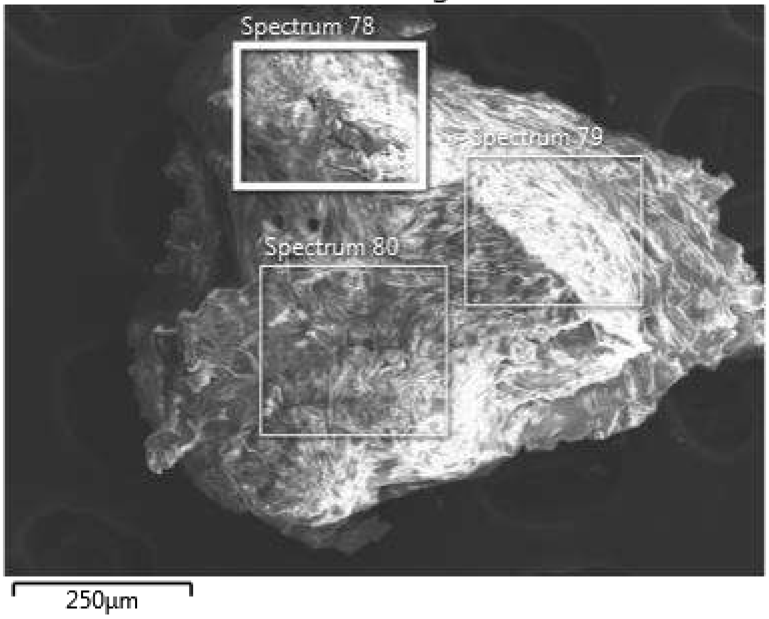

29/11/2019 10:04:49
Project 1
Cemento Mexico T1
CM2 15 Kv

Electron Image 29

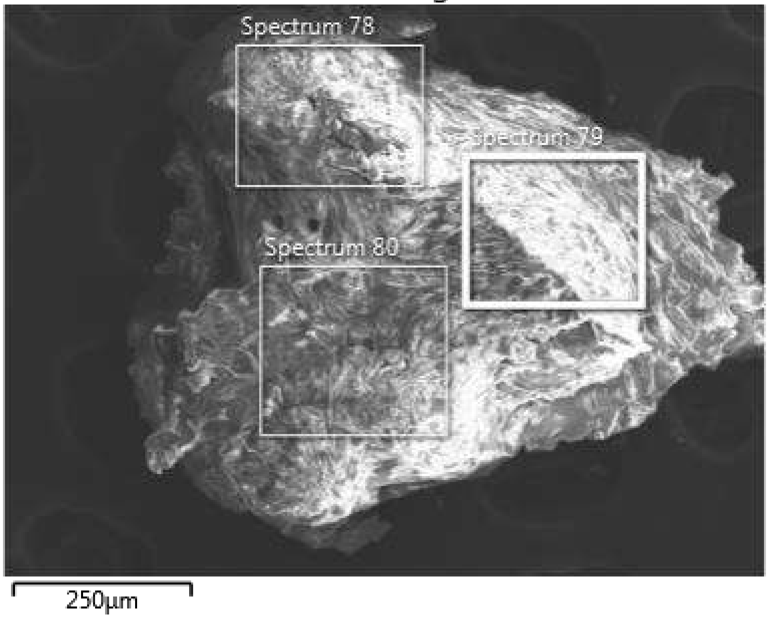

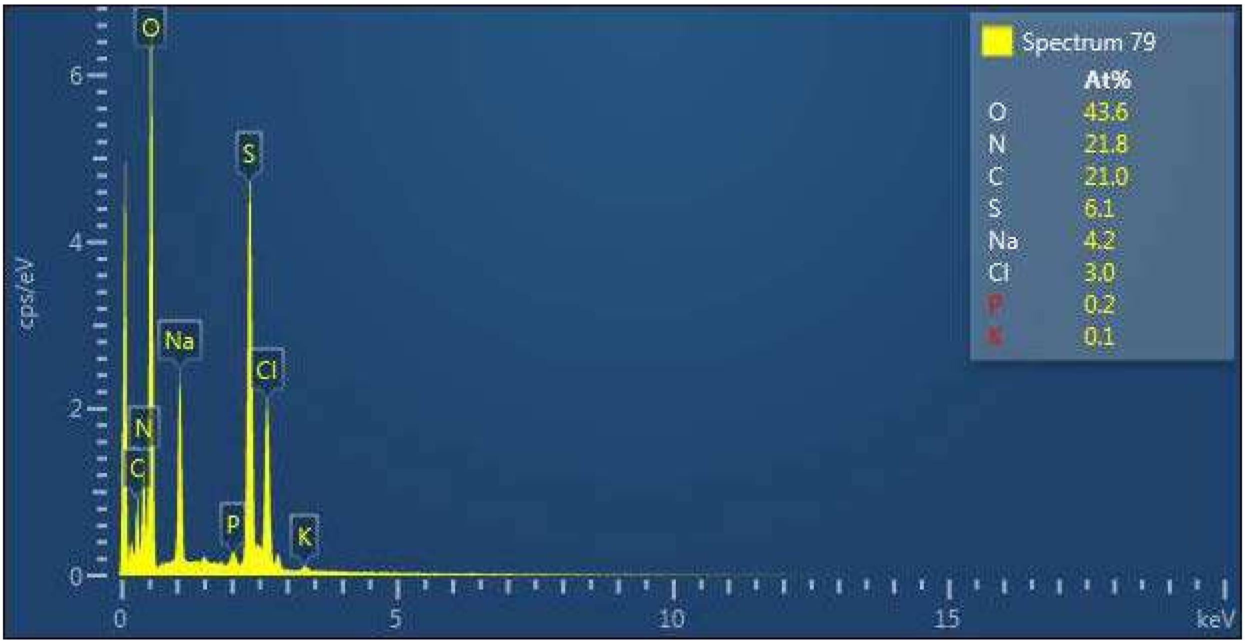

Label:	Spectrum 79
Source:	Acquired
Created:	29/11/2019 10:04:49
Livetime:	60.3s
Process Time:	4
Accelerating Voltage:	15.00kV
Magnification:	105 x
Working Distance:	9.9mm
Specimen Tilt (degrees):	0.0
Elevation (degrees):	35.0
Azimuth (degrees):	0.0
Number Of Channels:	2048
Energy Range (keV):	20 keV
Energy per Channel (eV):	10.0eV
Detector Type Id:	29
Detector Type:	X-Max
Window Type:	SATW
Pulse Pile Up Correction:	Succeeded
Primary Detector:	2617
Primary Detector Serial Number:	77871-X080

Element	Line Type	Apparent Concentration	k Ratio	Wt%	Wt% Sigma	Standard Label	Factory Standard	Standard Calibration Date
C	K series	1.60	0.01598	15.17	0.78	C Vit	Yes	
N	K series	13.74	0.02446	18.31	0.55	BN	Yes	
O	K series	19.44	0.06541	41.89	0.53	SiO2	Yes	
Na	K	3.96	0.01672	5.75	0.12	Albite	Yes	

	series							
P	K series	0.33	0.00184	0.36	0.04	GaP	Yes	
S	K series	7.34	0.06321	11.82	0.18	FeS2	Yes	
Cl	K series	3.62	0.03167	6.48	0.12	NaCl	Yes	
K	K series	0.13	0.00114	0.23	0.04	KBr	Yes	
Total:				100.00				

Electron Image 29

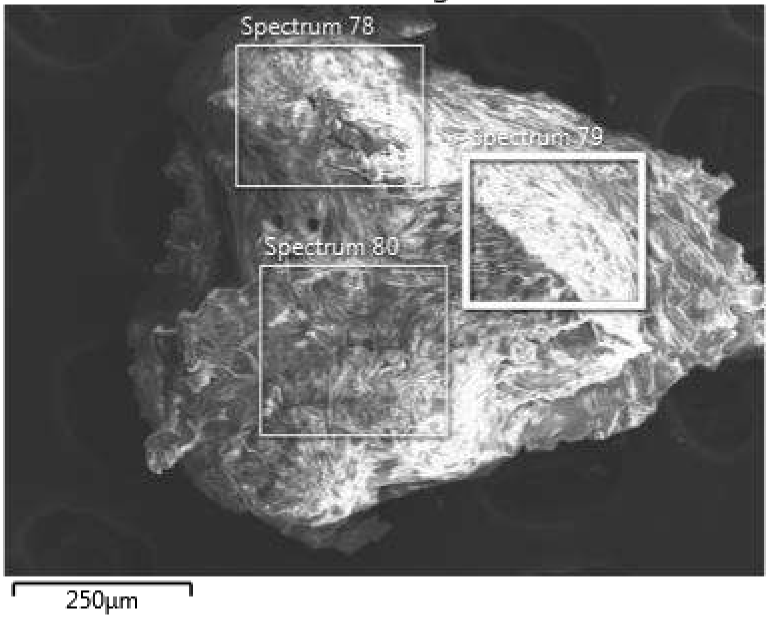

29/11/2019 10:05:58
Project 1
Cemento Mexico T1
CM2 15 Kv

Electron Image 29

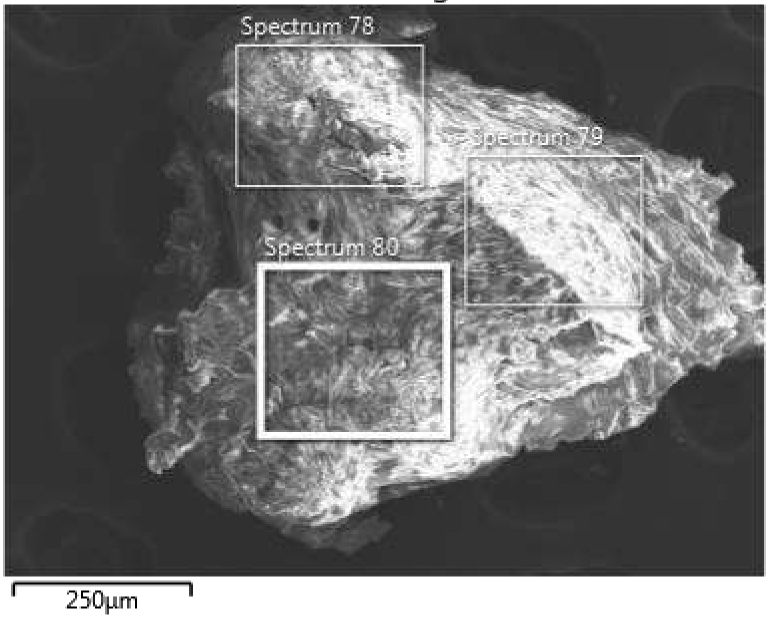

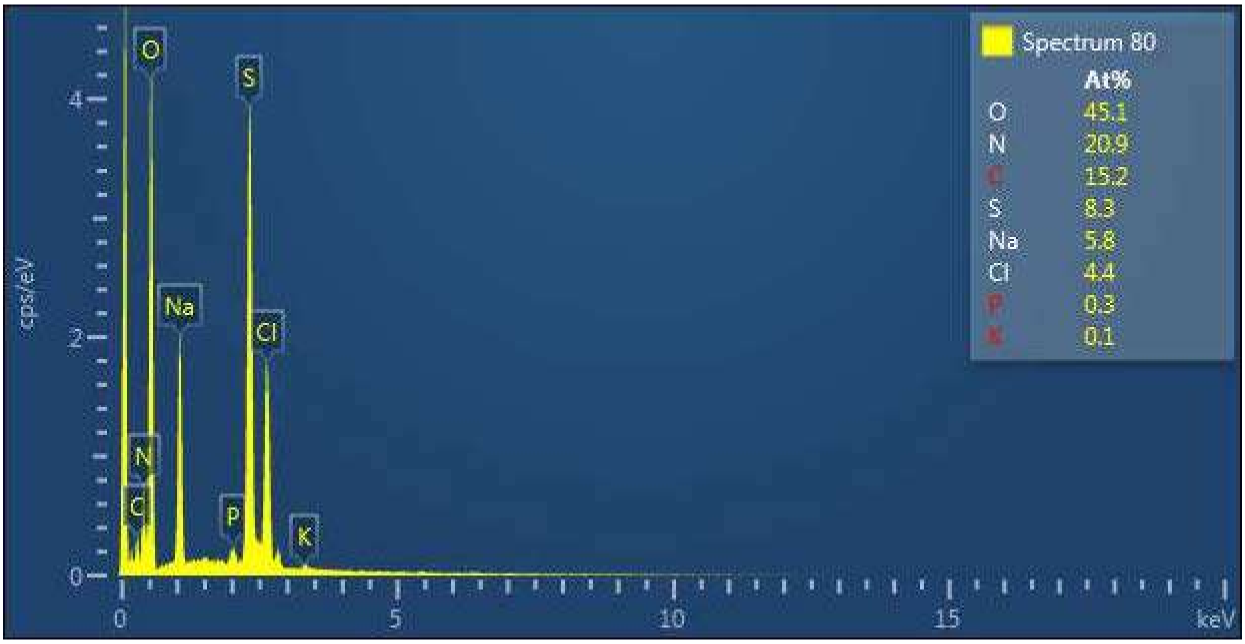

Label:	Spectrum 80
Source:	Acquired
Created:	29/11/2019 10:05:58
Livetime:	60.5s
Process Time:	4
Accelerating Voltage:	15.00kV
Magnification:	105 x
Working Distance:	9.9mm
Specimen Tilt (degrees):	0.0
Elevation (degrees):	35.0
Azimuth (degrees):	0.0
Number Of Channels:	2048
Energy Range (keV):	20 keV
Energy per Channel (eV):	10.0eV
Detector Type Id:	29
Detector Type:	X-Max
Window Type:	SATW
Pulse Pile Up Correction:	Succeeded
Primary Detector:	2617
Primary Detector Serial Number:	77871-X080

Element	Line Type	Apparent Concentration	k Ratio	Wt%	Wt% Sigma	Standard Label	Factory Standard	Standard Calibration Date
C	K series	0.55	0.00553	10.38	1.07	C Vit	Yes	
N	K series	7.71	0.01372	16.60	0.67	BN	Yes	
O	K series	12.38	0.04165	40.90	0.66	SiO2	Yes	
Na	K	3.37	0.01421	7.51	0.17	Albite	Yes	

	series							
P	K series	0.28	0.00154	0.48	0.06	GaP	Yes	
S	K series	5.99	0.05163	15.09	0.27	FeS2	Yes	
Cl	K series	3.10	0.02705	8.81	0.19	NaCl	Yes	
K	K series	0.09	0.00075	0.24	0.06	KBr	Yes	
Total:				100.00				

Electron Image 29

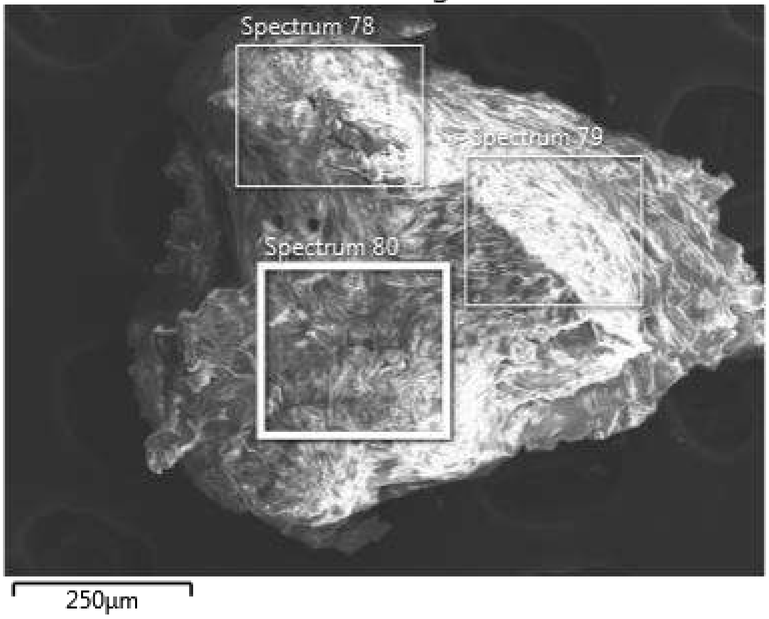

29/11/2019 10:08:40
Project 1
Cemento Mexico T1
CM3 15 Kv

Electron Image 30

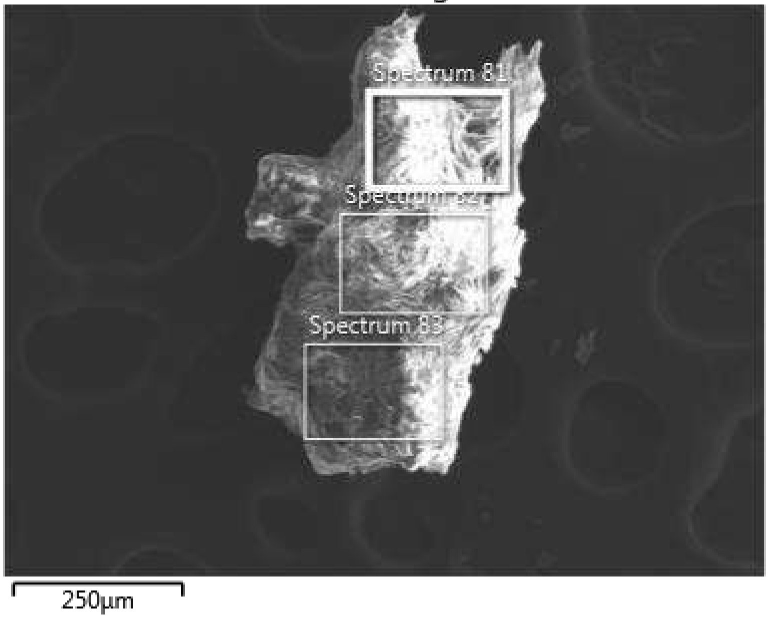

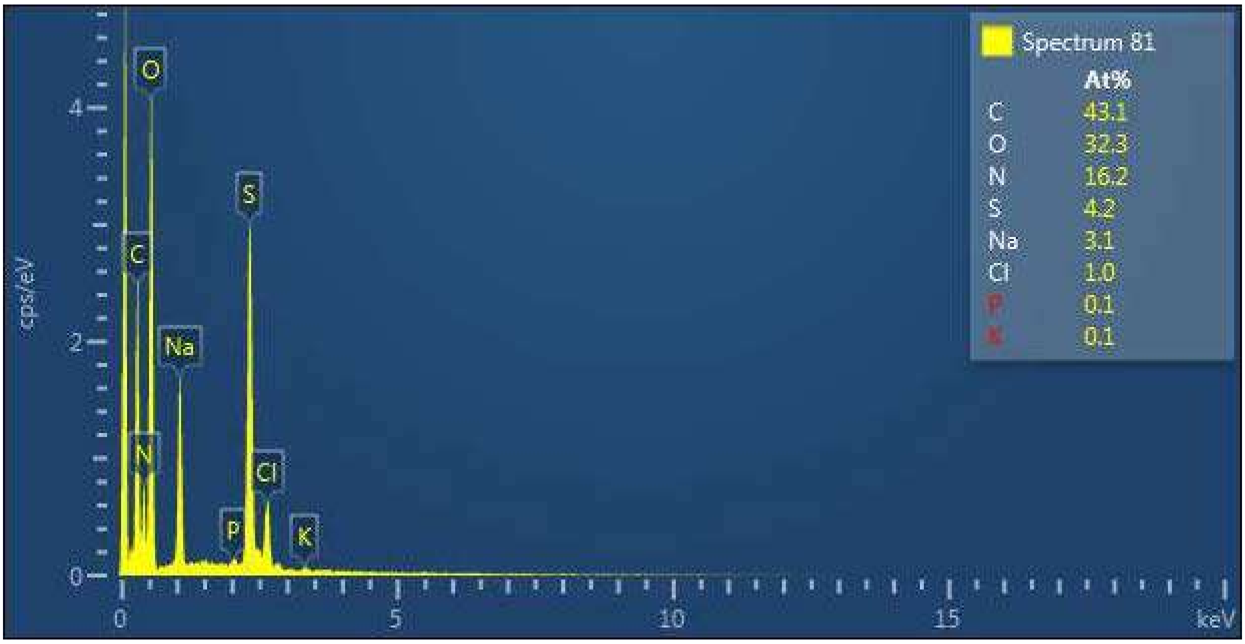

Label:	Spectrum 81
Source:	Acquired
Created:	29/11/2019 10:08:40
Livetime:	60.1s
Process Time:	4
Accelerating Voltage:	15.00kV
Magnification:	100 x
Working Distance:	9.9mm
Specimen Tilt (degrees):	0.0
Elevation (degrees):	35.0
Azimuth (degrees):	0.0
Number Of Channels:	2048
Energy Range (keV):	20 keV
Energy per Channel (eV):	10.0eV
Detector Type Id:	29
Detector Type:	X-Max
Window Type:	SATW
Pulse Pile Up Correction:	Succeeded
Primary Detector:	2617
Primary Detector Serial Number:	77871-X080

Element	Line Type	Apparent Concentration	k Ratio	Wt%	Wt% Sigma	Standard Label	Factory Standard	Standard Calibration Date
C	K series	4.59	0.04589	34.39	0.73	C Vit	Yes	
N	K series	7.15	0.01273	15.06	0.73	BN	Yes	
O	K series	11.98	0.04031	34.29	0.54	SiO2	Yes	
Na	K	2.82	0.01189	4.72	0.11	Albite	Yes	

	series							
P	K series	0.12	0.00070	0.16	0.04	GaP	Yes	
S	K series	4.64	0.03994	8.93	0.16	FeS2	Yes	
Cl	K series	1.06	0.00928	2.24	0.08	NaCl	Yes	
K	K series	0.10	0.00086	0.20	0.04	KBr	Yes	
Total:				100.00				

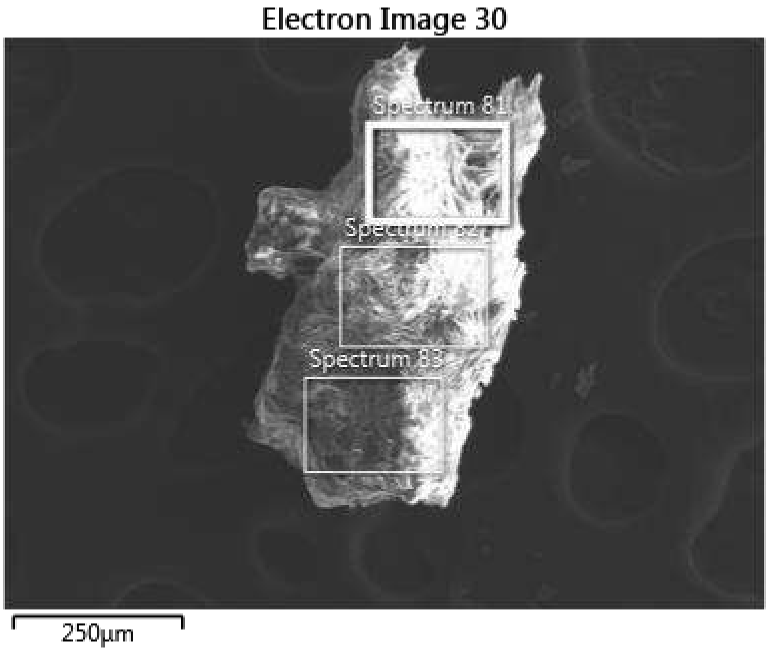

29/11/2019 10:09:50
Project 1
Cemento Mexico T1
CM3 15 Kv

Electron Image 30

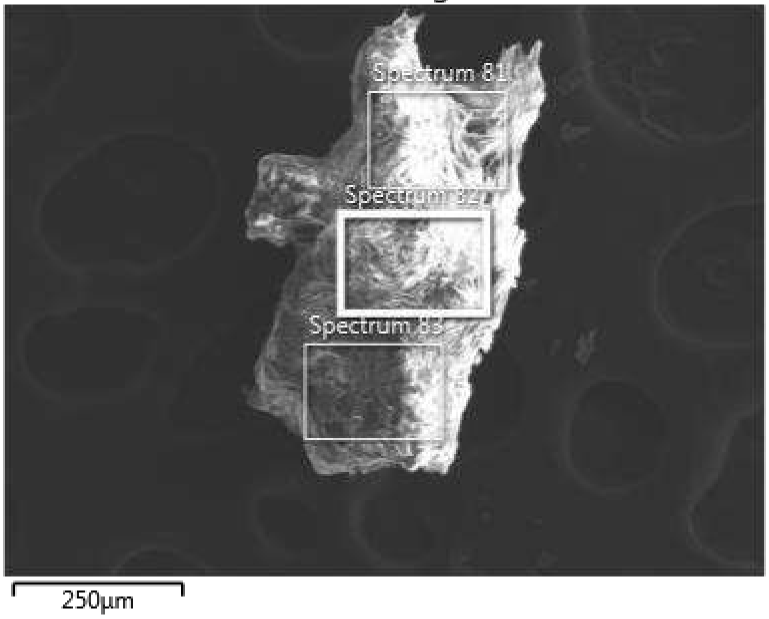

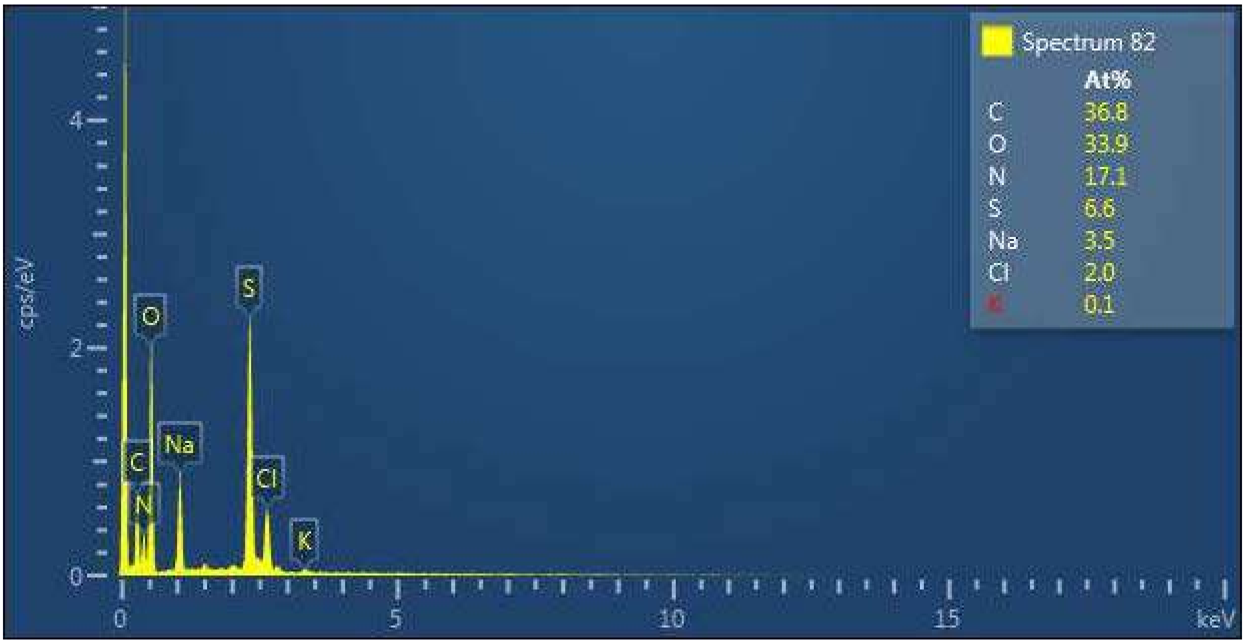

Label:	Spectrum 82
Source:	Acquired
Created:	29/11/2019 10:09:50
Livetime:	60.2s
Process Time:	4
Accelerating Voltage:	15.00kV
Magnification:	100 x
Working Distance:	9.9mm
Specimen Tilt (degrees):	0.0
Elevation (degrees):	35.0
Azimuth (degrees):	0.0
Number Of Channels:	2048
Energy Range (keV):	20 keV
Energy per Channel (eV):	10.0eV
Detector Type Id:	29
Detector Type:	X-Max
Window Type:	SATW
Pulse Pile Up Correction:	Succeeded
Primary Detector:	2617
Primary Detector Serial Number:	77871-X080

Element	Line Type	Apparent Concentration	k Ratio	Wt%	Wt% Sigma	Standard Label	Factory Standard	Standard Calibration Date
C	K series	1.29	0.01286	27.81	1.23	C Vit	Yes	
N	K series	3.49	0.00622	15.02	0.98	BN	Yes	
O	K series	5.83	0.01960	34.07	0.79	SiO2	Yes	
Na	K	1.47	0.00620	5.03	0.17	Albite	Yes	

	series							
S	K series	3.41	0.02934	13.39	0.33	FeS2	Yes	
Cl	K series	1.00	0.00872	4.41	0.16	NaCl	Yes	
K	K series	0.07	0.00058	0.28	0.07	KBr	Yes	
Total:				100.00				

Electron Image 30

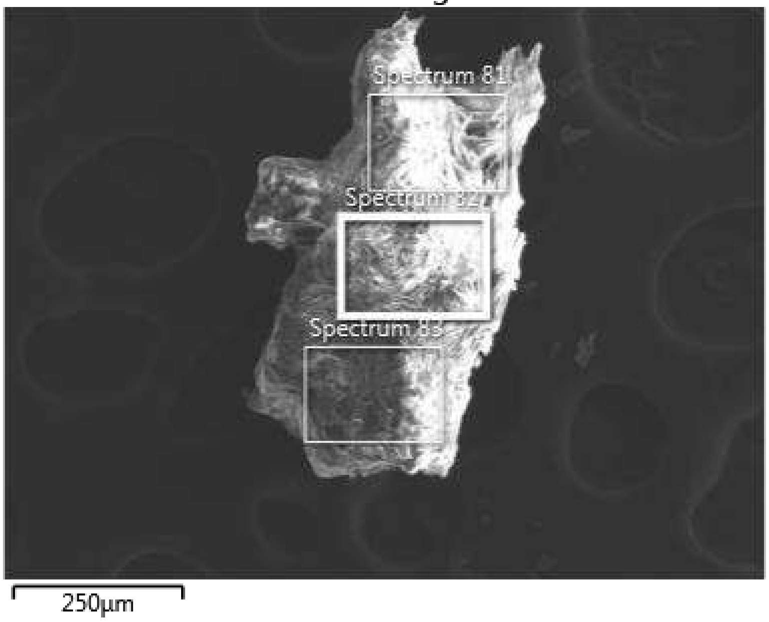

29/11/2019 10:10:59
Project 1
Cemento Mexico T1
CM3 15 Kv

Electron Image 30

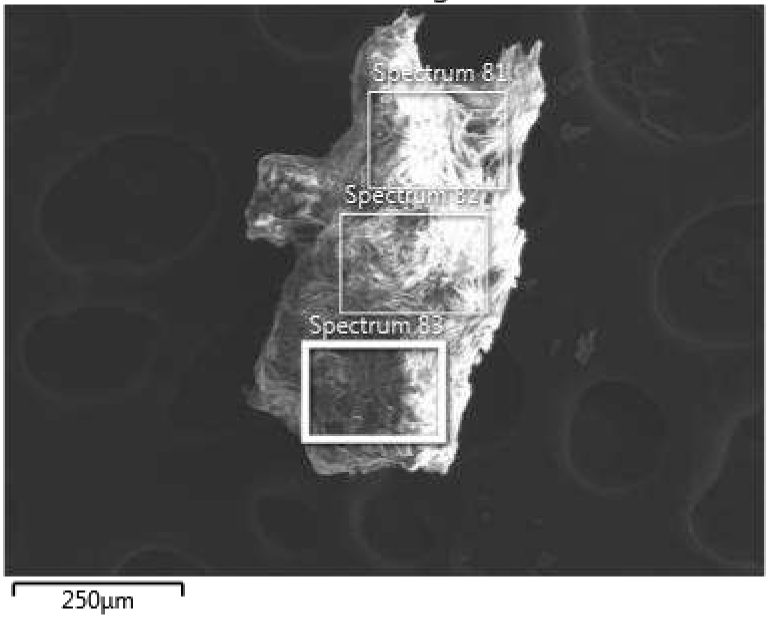

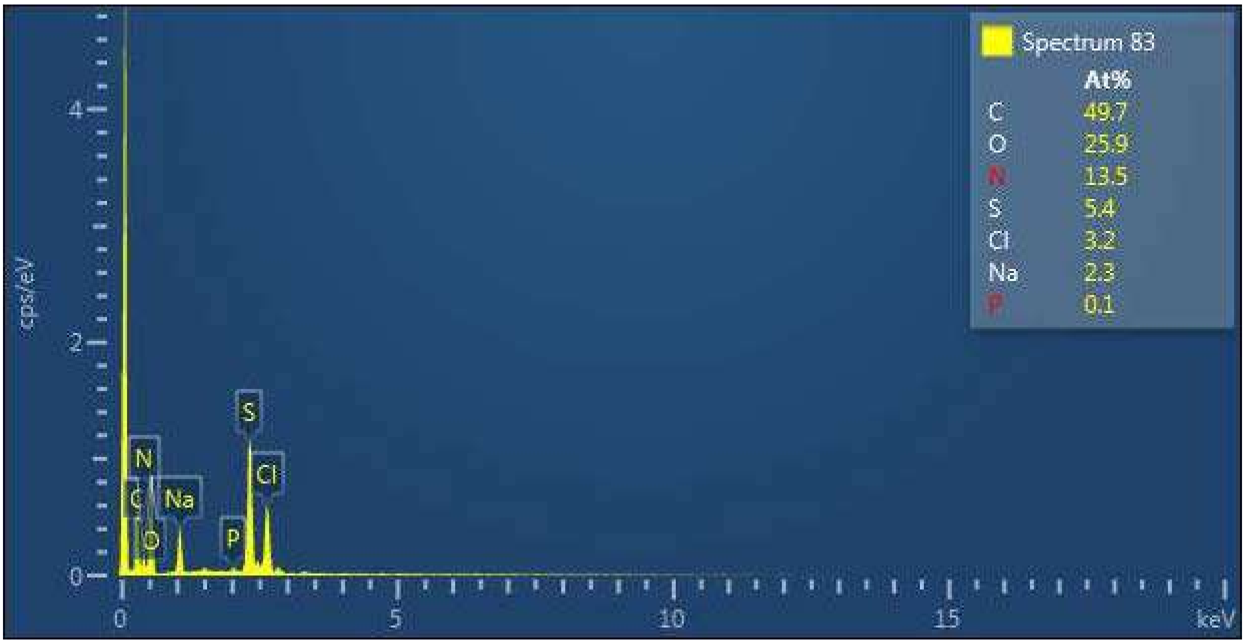

Label:	Spectrum 83
Source:	Acquired
Created:	29/11/2019 10:10:59
Livetime:	60.4s
Process Time:	4
Accelerating Voltage:	15.00kV
Magnification:	100 x
Working Distance:	9.9mm
Specimen Tilt (degrees):	0.0
Elevation (degrees):	35.0
Azimuth (degrees):	0.0
Number Of Channels:	2048
Energy Range (keV):	20 keV
Energy per Channel (eV):	10.0eV
Detector Type Id:	29
Detector Type:	X-Max
Window Type:	SATW
Pulse Pile Up Correction:	Succeeded
Primary Detector:	2617
Primary Detector Serial Number:	77871-X080

Element	Line Type	Apparent Concentration	k Ratio	Wt%	Wt% Sigma	Standard Label	Factory Standard	Standard Calibration Date
C	K series	1.11	0.01114	38.72	1.25	C Vit	Yes	
N	K series	1.43	0.00254	12.24	1.40	BN	Yes	
O	K series	2.59	0.00873	26.87	0.84	SiO2	Yes	
Na	K	0.65	0.00275	3.44	0.17	Albite	Yes	

	series							
P	K series	0.05	0.00030	0.23	0.07	GaP	Yes	
S	K series	1.81	0.01561	11.25	0.35	FeS2	Yes	
Cl	K series	1.05	0.00914	7.25	0.27	NaCl	Yes	
Total:				100.00				

Electron Image 30

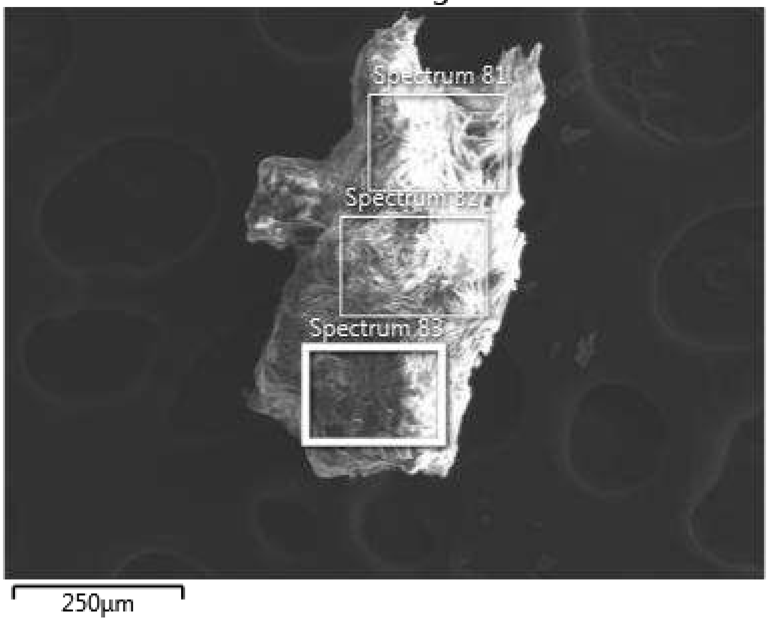

29/11/2019 10:14:48
Project 1
Cemento Mexico T2
CM1 15 Kv

Electron Image 31

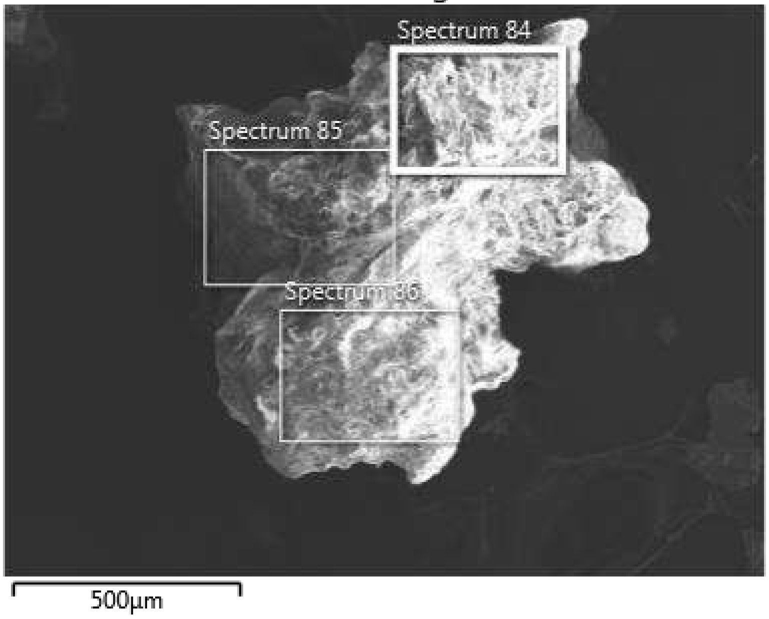

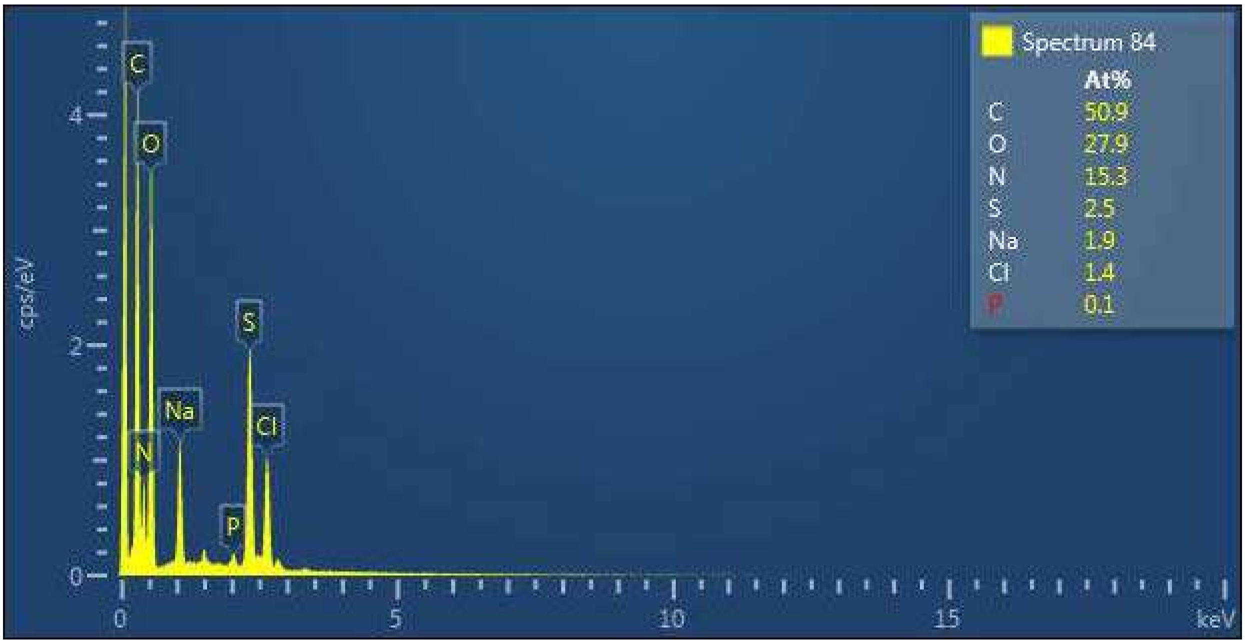

Label:	Spectrum 84
Source:	Acquired
Created:	29/11/2019 10:14:48
Livetime:	60.4s
Process Time:	4
Accelerating Voltage:	15.00kV
Magnification:	67 x
Working Distance:	9.9mm
Specimen Tilt (degrees):	0.0
Elevation (degrees):	35.0
Azimuth (degrees):	0.0
Number Of Channels:	2048
Energy Range (keV):	20 keV
Energy per Channel (eV):	10.0eV
Detector Type Id:	29
Detector Type:	X-Max
Window Type:	SATW
Pulse Pile Up Correction:	Succeeded
Primary Detector:	2617
Primary Detector Serial Number:	77871-X080

Element	Line Type	Apparent Concentration	k Ratio	Wt%	Wt% Sigma	Standard Label	Factory Standard	Standard Calibration Date
C	K series	6.84	0.06844	42.16	0.60	C Vit	Yes	
N	K series	6.52	0.01161	14.76	0.79	BN	Yes	
O	K series	10.44	0.03514	30.77	0.47	SiO2	Yes	
Na	K	1.89	0.00797	3.00	0.09	Albite	Yes	

	series							
P	K series	0.22	0.00121	0.27	0.04	GaP	Yes	
S	K series	3.01	0.02597	5.53	0.11	FeS2	Yes	
Cl	K series	1.77	0.01547	3.50	0.09	NaCl	Yes	
Total:				100.00				

Electron Image 31

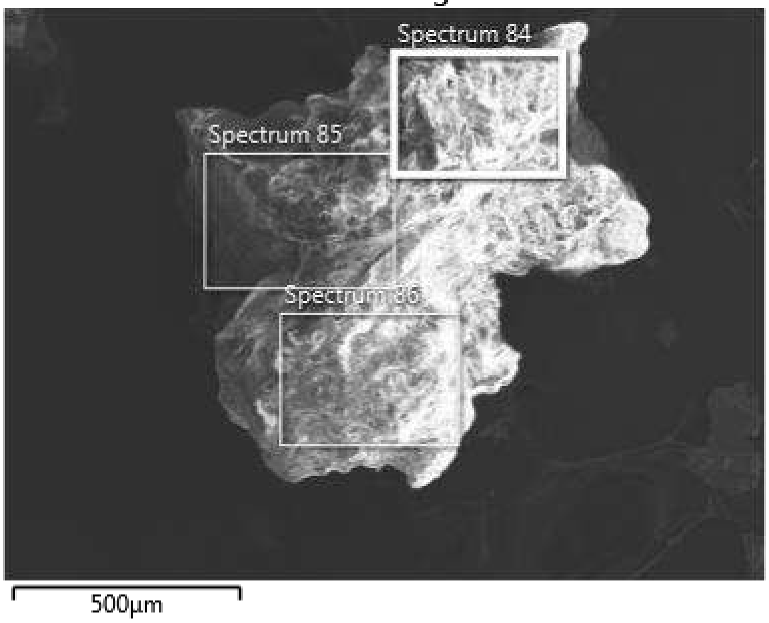

29/11/2019 10:15:57
Project 1
Cemento Mexico T2
CM1 15 Kv

Electron Image 31

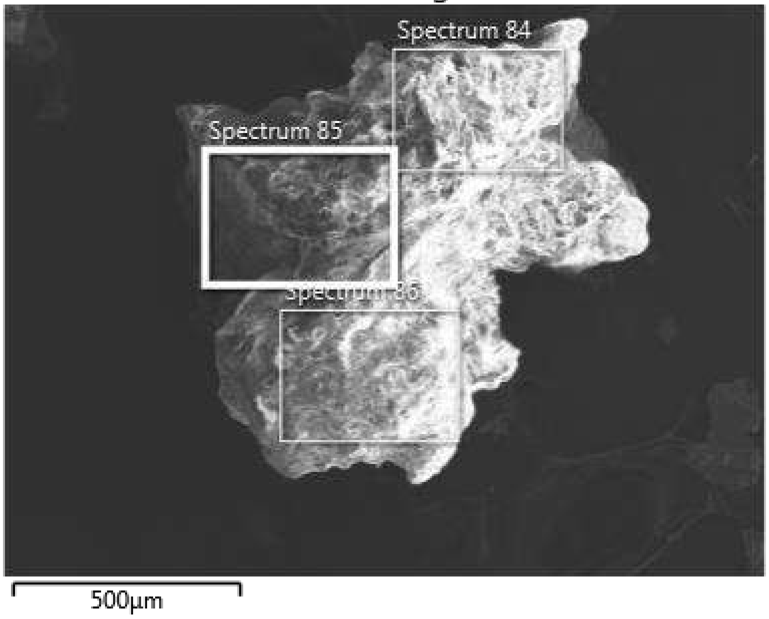

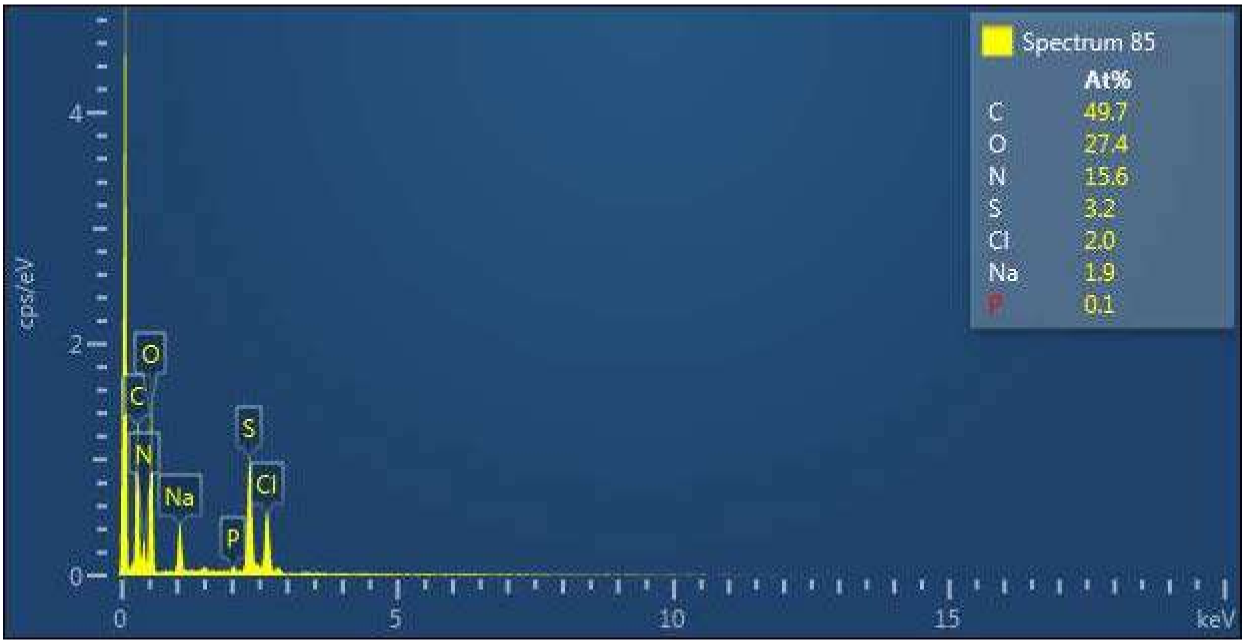

Label:	Spectrum 85
Source:	Acquired
Created:	29/11/2019 10:15:57
Livetime:	60.4s
Process Time:	4
Accelerating Voltage:	15.00kV
Magnification:	67 x
Working Distance:	9.9mm
Specimen Tilt (degrees):	0.0
Elevation (degrees):	35.0
Azimuth (degrees):	0.0
Number Of Channels:	2048
Energy Range (keV):	20 keV
Energy per Channel (eV):	10.0eV
Detector Type Id:	29
Detector Type:	X-Max
Window Type:	SATW
Pulse Pile Up Correction:	Succeeded
Primary Detector:	2617
Primary Detector Serial Number:	77871-X080

Element	Line Type	Apparent Concentration	k Ratio	Wt%	Wt% Sigma	Standard Label	Factory Standard	Standard Calibration Date
C	K series	2.22	0.02218	40.44	0.97	C Vit	Yes	
N	K series	2.56	0.00457	14.76	1.22	BN	Yes	
O	K series	3.98	0.01340	29.71	0.73	SiO2	Yes	
Na	K	0.77	0.00324	3.03	0.13	Albite	Yes	

	series							
P	K series	0.09	0.00053	0.29	0.06	GaP	Yes	
S	K series	1.51	0.01305	6.92	0.21	FeS2	Yes	
Cl	K series	0.97	0.00851	4.84	0.17	NaCl	Yes	
Total:				100.00				

Electron Image 31

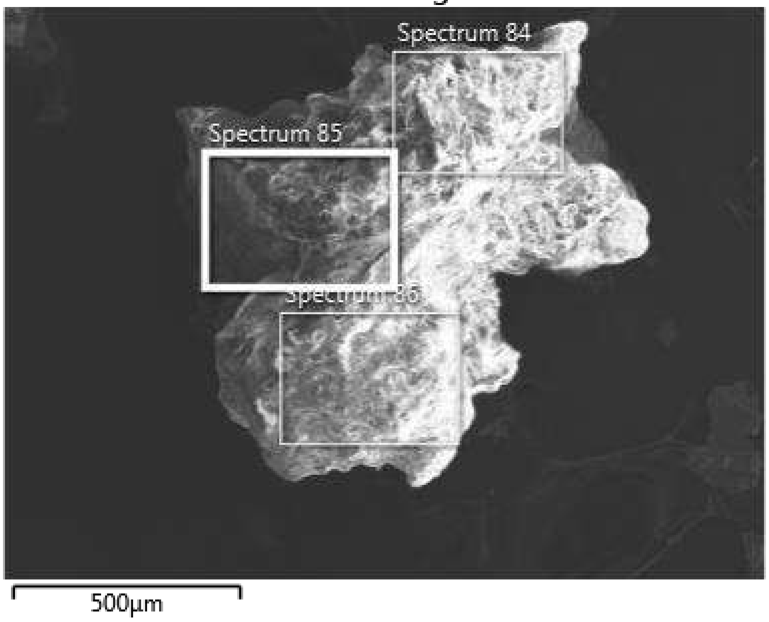

29/11/2019 10:17:05
Project 1
Cemento Mexico T2
CM1 15 Kv

Electron Image 31

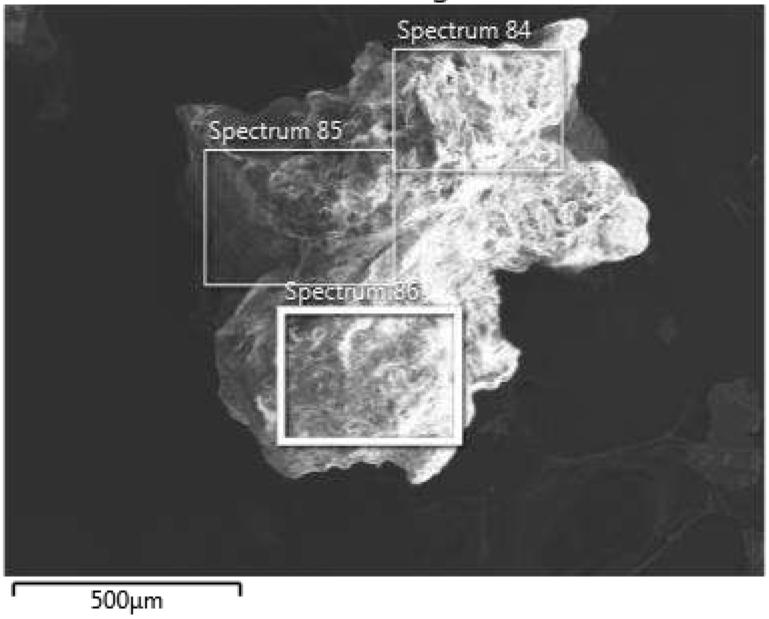

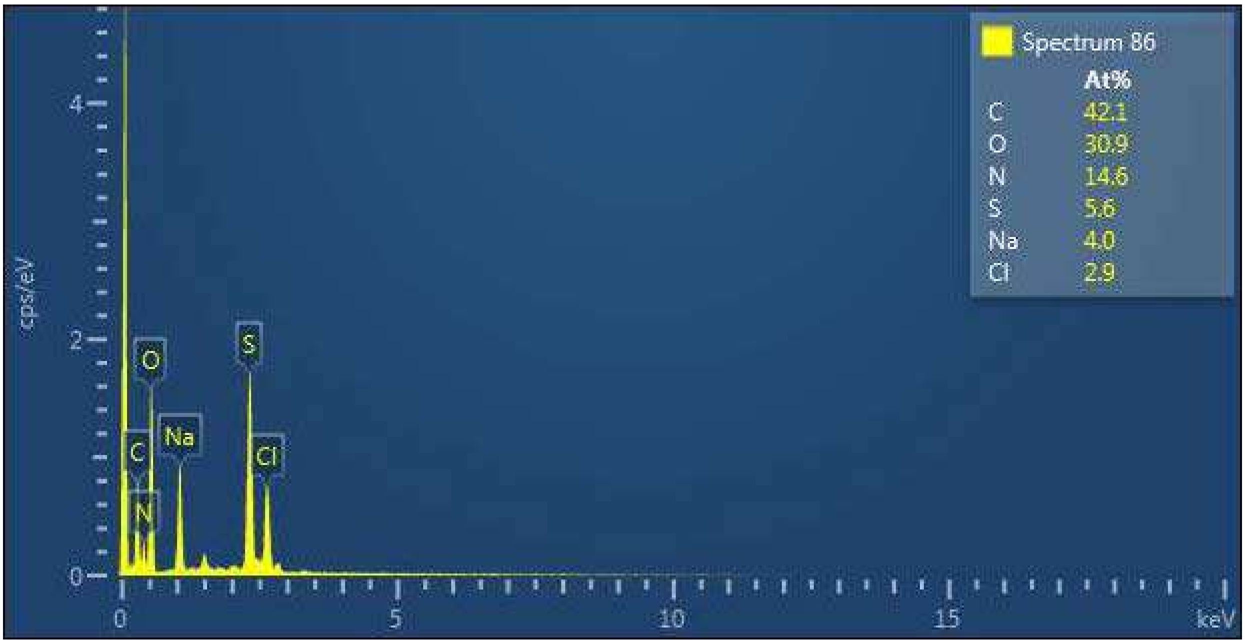

Label:	Spectrum 86
Source:	Acquired
Created:	29/11/2019 10:17:05
Livetime:	60.4s
Process Time:	4
Accelerating Voltage:	15.00kV
Magnification:	67 x
Working Distance:	9.9mm
Specimen Tilt (degrees):	0.0
Elevation (degrees):	35.0
Azimuth (degrees):	0.0
Number Of Channels:	2048
Energy Range (keV):	20 keV
Energy per Channel (eV):	10.0eV
Detector Type Id:	29
Detector Type:	X-Max
Window Type:	SATW
Pulse Pile Up Correction:	Succeeded
Primary Detector:	2617
Primary Detector Serial Number:	77871-X080

Element	Line Type	Apparent Concentration	k Ratio	Wt%	Wt% Sigma	Standard Label	Factory Standard	Standard Calibration Date
C	K series	1.31	0.01309	32.02	0.98	C Vit	Yes	
N	K series	2.46	0.00439	12.92	1.07	BN	Yes	
O	K series	4.71	0.01585	31.30	0.71	SiO2	Yes	
Na	K	1.56	0.00658	5.82	0.19	Albite	Yes	

	series							
S	K series	2.61	0.02246	11.39	0.28	FeS2	Yes	
Cl	K series	1.34	0.01175	6.54	0.20	NaCl	Yes	
Total:				100.00				

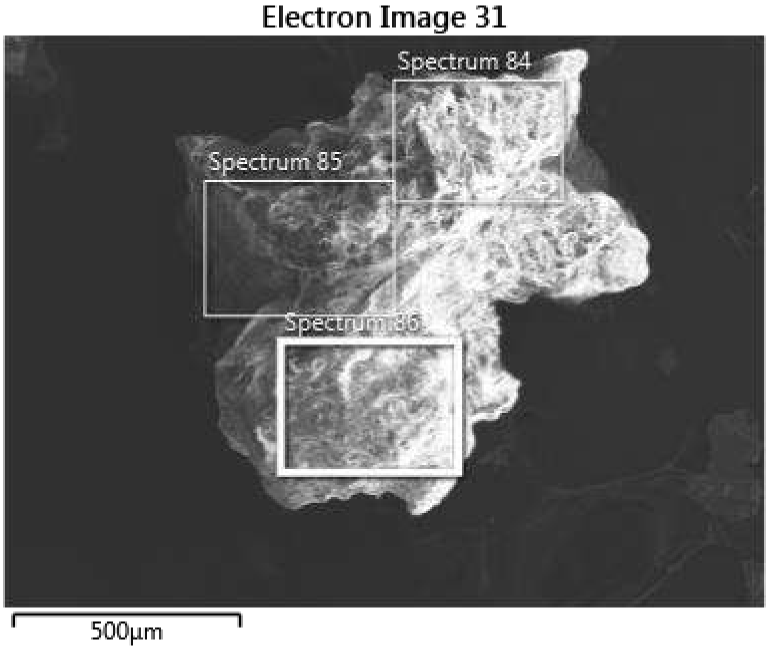

29/11/2019 10:19:18
Project 1
Cemento Mexico T2
CM2 15 Kv

Electron Image 32

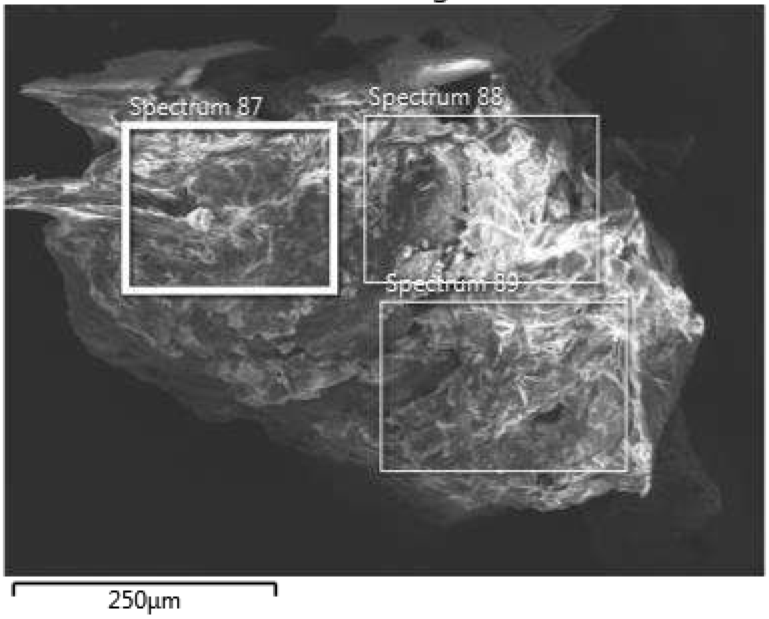

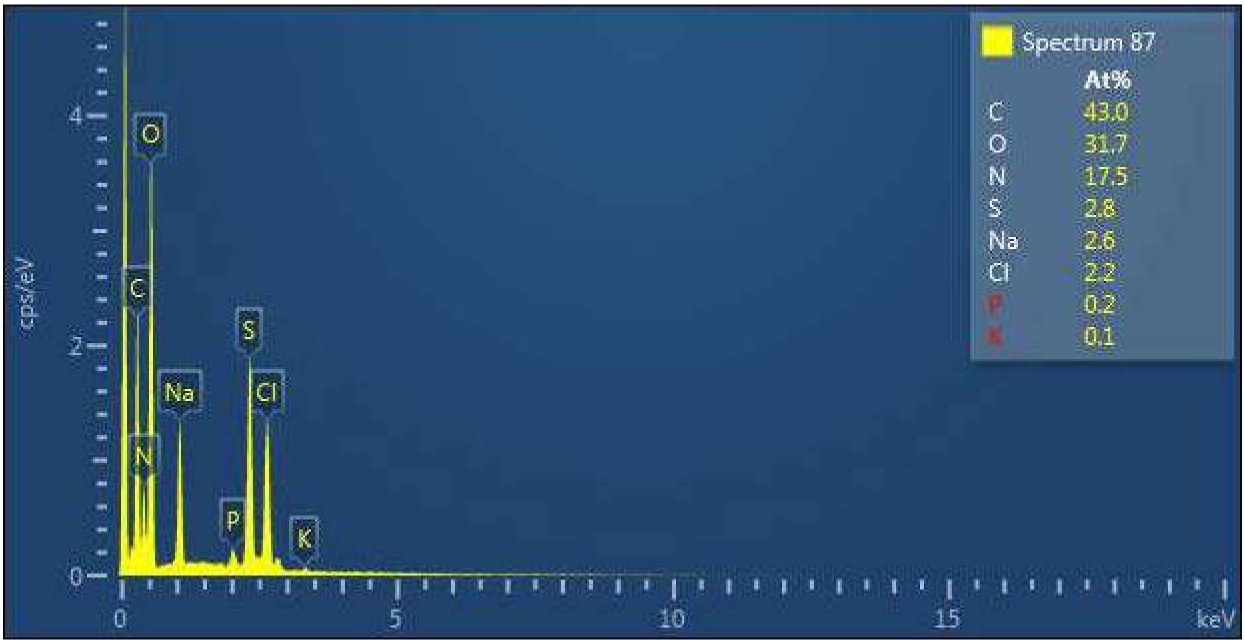

Label:	Spectrum 87
Source:	Acquired
Created:	29/11/2019 10:19:18
Livetime:	60.4s
Process Time:	4
Accelerating Voltage:	15.00kV
Magnification:	155 x
Working Distance:	9.9mm
Specimen Tilt (degrees):	0.0
Elevation (degrees):	35.0
Azimuth (degrees):	0.0
Number Of Channels:	2048
Energy Range (keV):	20 keV
Energy per Channel (eV):	10.0eV
Detector Type Id:	29
Detector Type:	X-Max
Window Type:	SATW
Pulse Pile Up Correction:	Succeeded
Primary Detector:	2617
Primary Detector Serial Number:	77871-X080

Element	Line Type	Apparent Concentration	k Ratio	Wt%	Wt% Sigma	Standard Label	Factory Standard	Standard Calibration Date
C	K series	4.10	0.04105	34.33	0.78	C Vit	Yes	
N	K series	7.02	0.01250	16.25	0.77	BN	Yes	
O	K series	10.48	0.03525	33.75	0.57	SiO2	Yes	
Na	K	2.13	0.00899	3.92	0.11	Albite	Yes	

	series							
P	K series	0.31	0.00172	0.44	0.04	GaP	Yes	
S	K series	2.81	0.02424	5.90	0.13	FeS2	Yes	
Cl	K series	2.33	0.02033	5.26	0.13	NaCl	Yes	
K	K series	0.07	0.00062	0.16	0.04	KBr	Yes	
Total:				100.00				

Electron Image 32

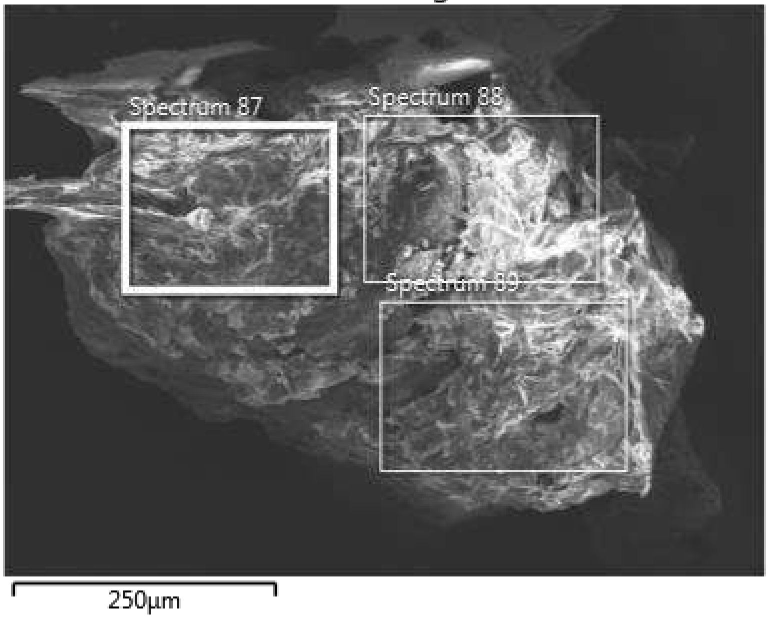

29/11/2019 10:20:26
Project 1
Cemento Mexico T2
CM2 15 Kv

Electron Image 32

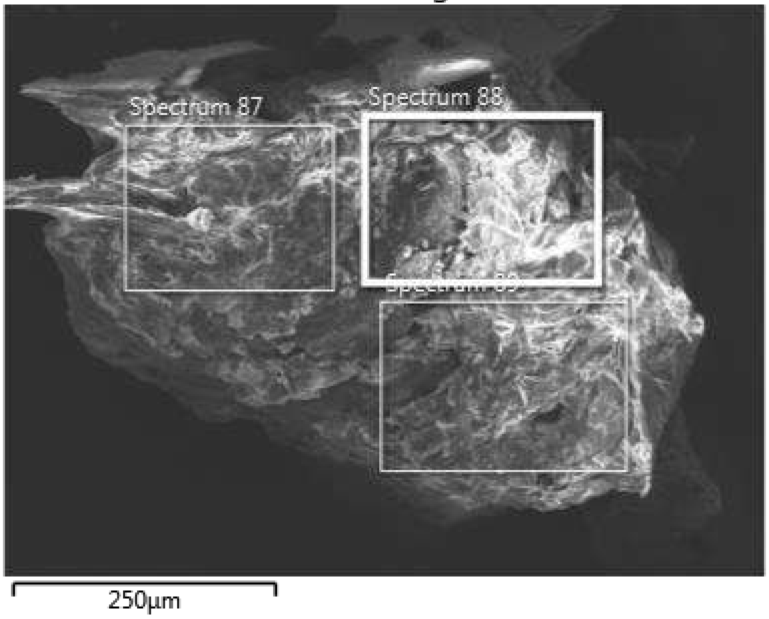

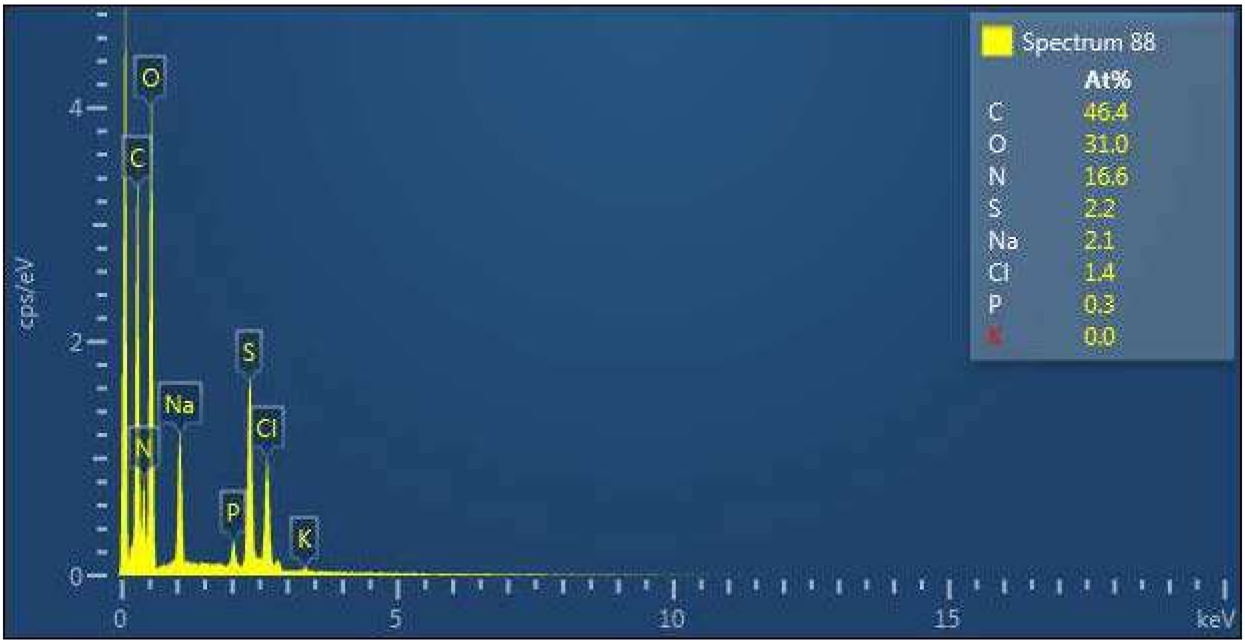

Label:	Spectrum 88
Source:	Acquired
Created:	29/11/2019 10:20:26
Livetime:	60.0s
Process Time:	4
Accelerating Voltage:	15.00kV
Magnification:	155 x
Working Distance:	9.9mm
Specimen Tilt (degrees):	0.0
Elevation (degrees):	35.0
Azimuth (degrees):	0.0
Number Of Channels:	2048
Energy Range (keV):	20 keV
Energy per Channel (eV):	10.0eV
Detector Type Id:	29
Detector Type:	X-Max
Window Type:	SATW
Pulse Pile Up Correction:	Succeeded
Primary Detector:	2617
Primary Detector Serial Number:	77871-X080

Element	Line Type	Apparent Concentration	k Ratio	Wt%	Wt% Sigma	Standard Label	Factory Standard	Standard Calibration Date
C	K series	6.18	0.06182	38.11	0.69	C Vit	Yes	
N	K series	7.50	0.01335	15.89	0.75	BN	Yes	
O	K series	11.64	0.03918	33.89	0.53	SiO2	Yes	
Na	K	1.98	0.00835	3.29	0.09	Albite	Yes	

	series							
P	K series	0.45	0.00254	0.59	0.04	GaP	Yes	
S	K series	2.55	0.02198	4.83	0.11	FeS2	Yes	
Cl	K series	1.61	0.01409	3.27	0.09	NaCl	Yes	
K	K series	0.06	0.00054	0.12	0.04	KBr	Yes	
Total:				100.00				

Electron Image 32

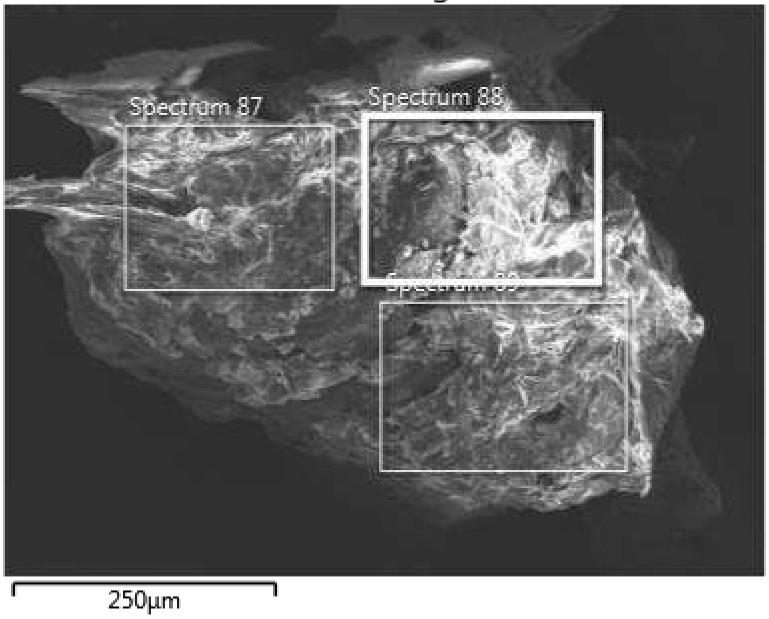

29/11/2019 10:21:33
Project 1
Cemento Mexico T2
CM2 15 Kv

Electron Image 32

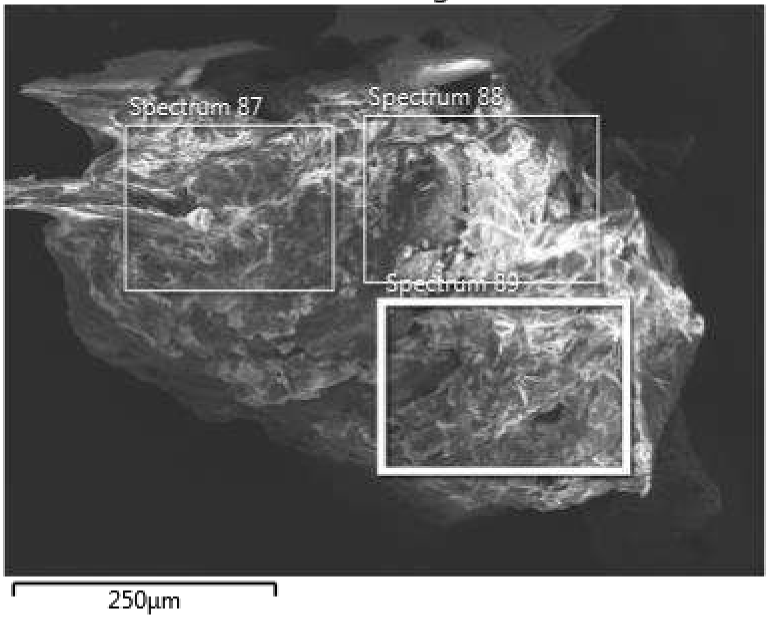

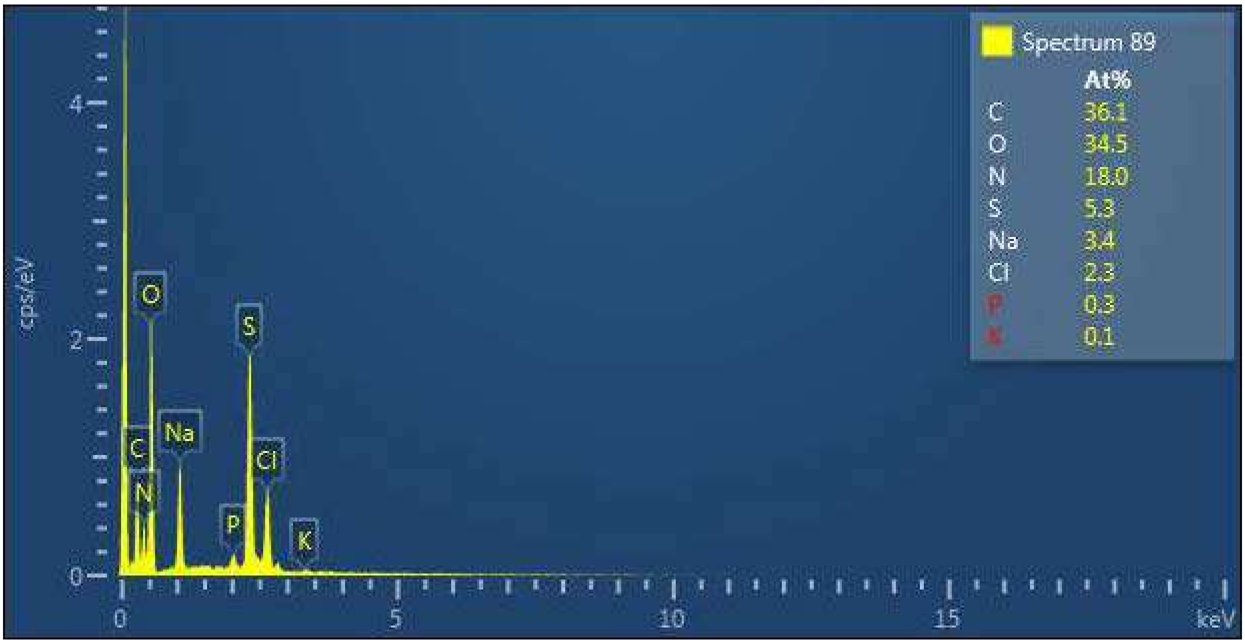

Label:	Spectrum 89
Source:	Acquired
Created:	29/11/2019 10:21:33
Livetime:	60.3s
Process Time:	4
Accelerating Voltage:	15.00kV
Magnification:	155 x
Working Distance:	9.9mm
Specimen Tilt (degrees):	0.0
Elevation (degrees):	35.0
Azimuth (degrees):	0.0
Number Of Channels:	2048
Energy Range (keV):	20 keV
Energy per Channel (eV):	10.0eV
Detector Type Id:	29
Detector Type:	X-Max
Window Type:	SATW
Pulse Pile Up Correction:	Succeeded
Primary Detector:	2617
Primary Detector Serial Number:	77871-X080

Element	Line Type	Apparent Concentration	k Ratio	Wt%	Wt% Sigma	Standard Label	Factory Standard	Standard Calibration Date
C	K series	1.45	0.01454	27.48	1.18	C Vit	Yes	
N	K series	4.16	0.00740	15.97	0.95	BN	Yes	
O	K series	6.48	0.02181	34.99	0.78	SiO2	Yes	
Na	K	1.55	0.00652	4.94	0.16	Albite	Yes	

	series							
P	K series	0.24	0.00135	0.61	0.07	GaP	Yes	
S	K series	2.91	0.02509	10.67	0.27	FeS2	Yes	
Cl	K series	1.26	0.01101	5.10	0.17	NaCl	Yes	
K	K series	0.06	0.00053	0.24	0.06	KBr	Yes	
Total:				100.00				

Electron Image 32

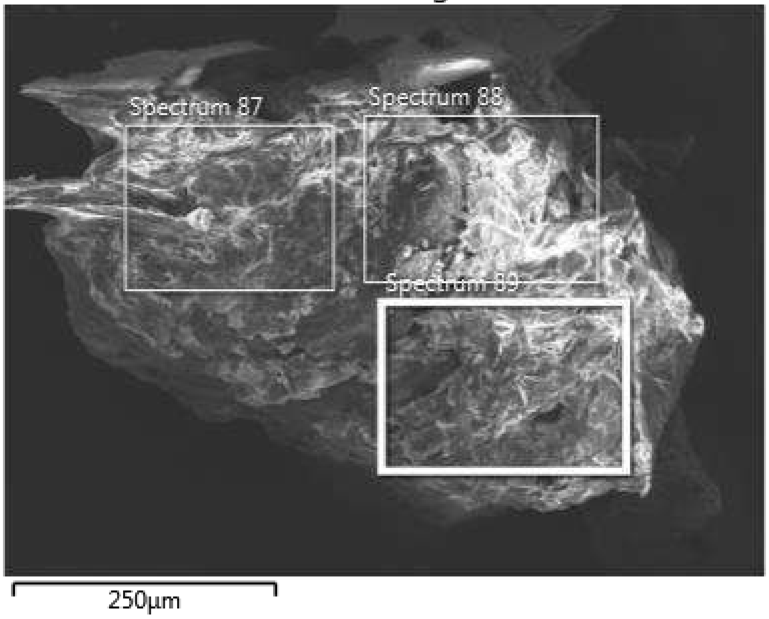

29/11/2019 10:24:42
Project 1
Cemento Mexico T2
CM3 15 Kv

Electron Image 33

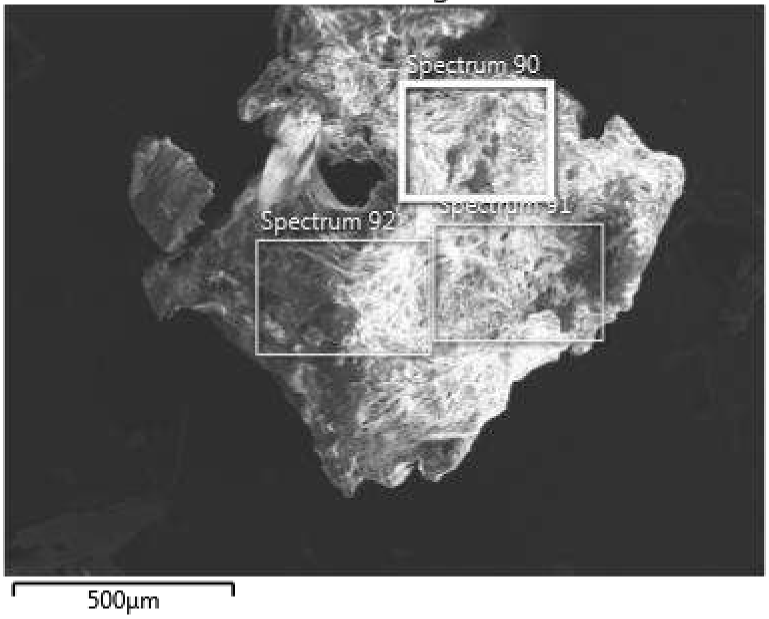

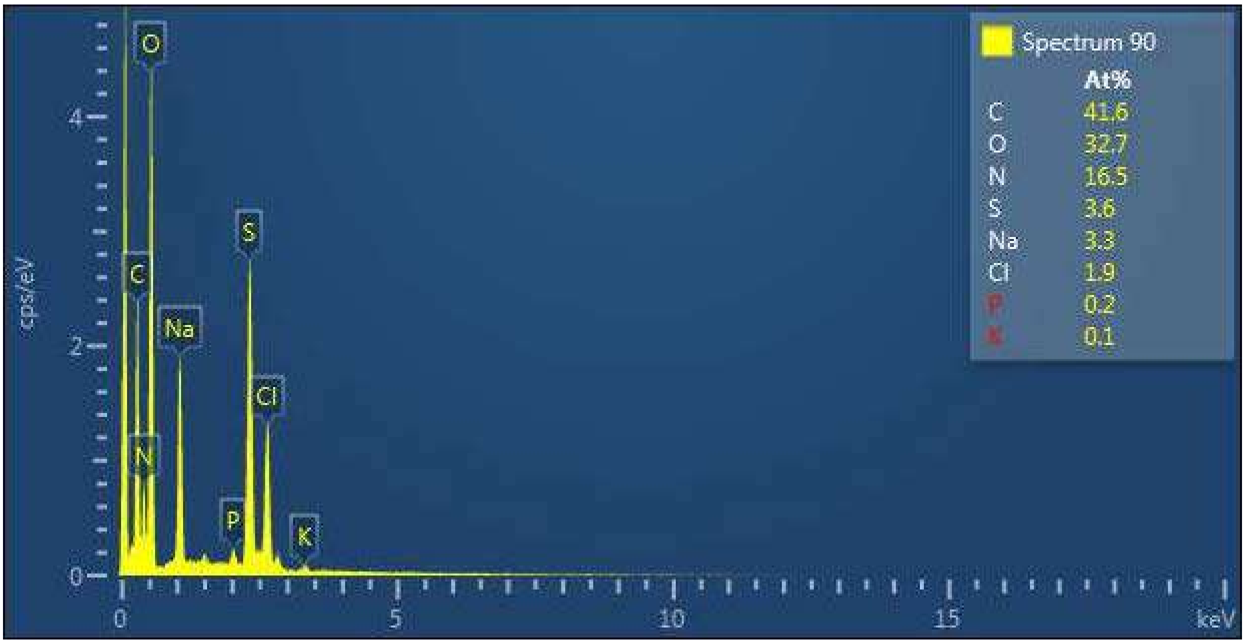

Label:	Spectrum 90
Source:	Acquired
Created:	29/11/2019 10:24:42
Livetime:	60.3s
Process Time:	4
Accelerating Voltage:	15.00kV
Magnification:	65 x
Working Distance:	9.9mm
Specimen Tilt (degrees):	0.0
Elevation (degrees):	35.0
Azimuth (degrees):	0.0
Number Of Channels:	2048
Energy Range (keV):	20 keV
Energy per Channel (eV):	10.0eV
Detector Type Id:	29
Detector Type:	X-Max
Window Type:	SATW
Pulse Pile Up Correction:	Succeeded
Primary Detector:	2617
Primary Detector Serial Number:	77871-X080

Element	Line Type	Apparent Concentration	k Ratio	Wt%	Wt% Sigma	Standard Label	Factory Standard	Standard Calibration Date
C	K series	4.29	0.04293	32.76	0.74	C Vit	Yes	
N	K series	7.71	0.01373	15.19	0.69	BN	Yes	
O	K series	12.77	0.04298	34.36	0.52	SiO2	Yes	
Na	K	3.17	0.01339	4.99	0.11	Albite	Yes	

	series							
P	K series	0.29	0.00160	0.35	0.04	GaP	Yes	
S	K series	4.22	0.03638	7.64	0.14	FeS2	Yes	
Cl	K series	2.27	0.01984	4.46	0.11	NaCl	Yes	
K	K series	0.13	0.00112	0.25	0.04	KBr	Yes	
Total:				100.00				

Electron Image 33

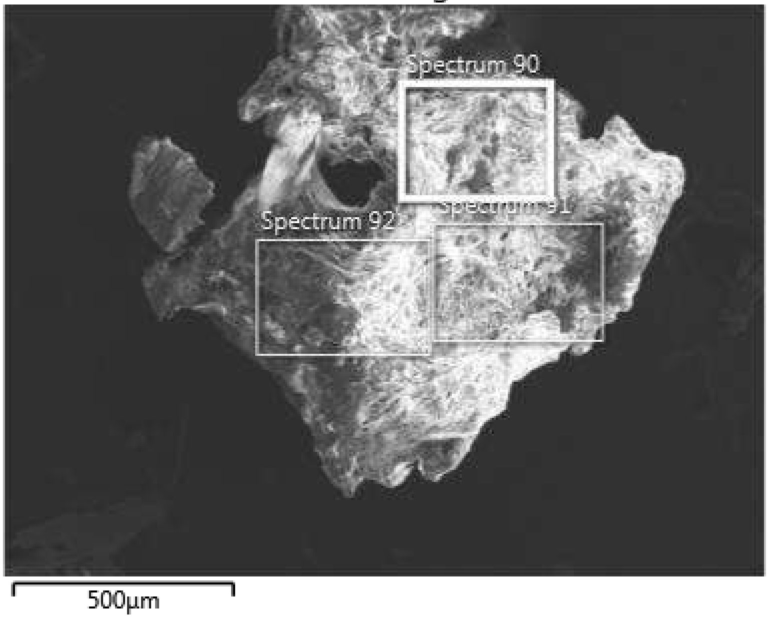

29/11/2019 10:25:51
Project 1
Cemento Mexico T2
CM3 15 Kv

Electron Image 33

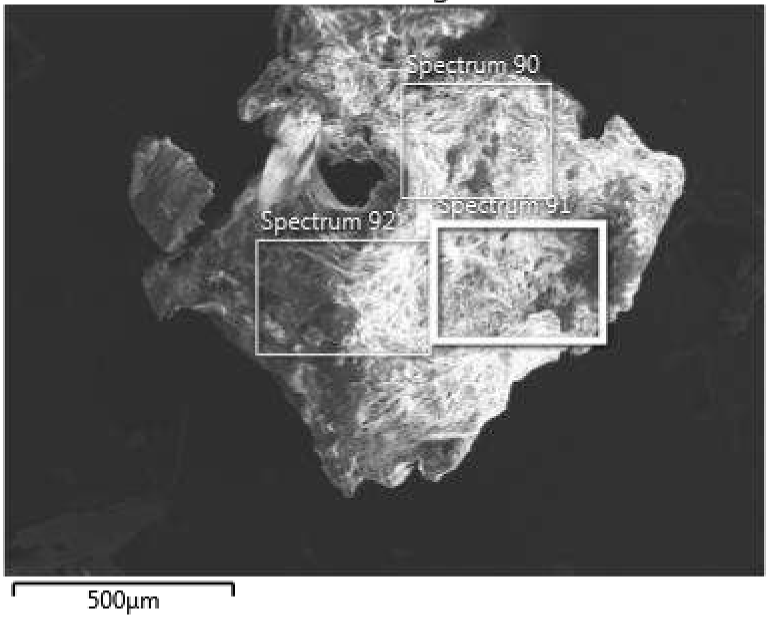

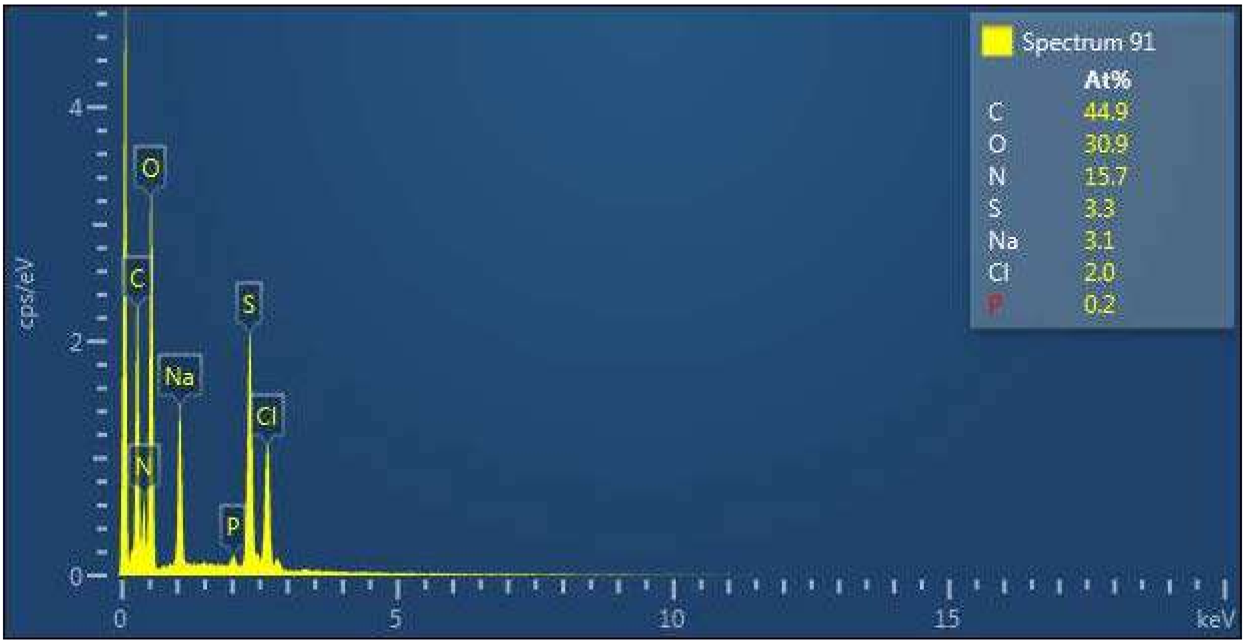

Label:	Spectrum 91
Source:	Acquired
Created:	29/11/2019 10:25:51
Livetime:	60.4s
Process Time:	4
Accelerating Voltage:	15.00kV
Magnification:	65 x
Working Distance:	9.9mm
Specimen Tilt (degrees):	0.0
Elevation (degrees):	35.0
Azimuth (degrees):	0.0
Number Of Channels:	2048
Energy Range (keV):	20 keV
Energy per Channel (eV):	10.0eV
Detector Type Id:	29
Detector Type:	X-Max
Window Type:	SATW
Pulse Pile Up Correction:	Succeeded
Primary Detector:	2617
Primary Detector Serial Number:	77871-X080

Element	Line Type	Apparent Concentration	k Ratio	Wt%	Wt% Sigma	Standard Label	Factory Standard	Standard Calibration Date
C	K series	3.83	0.03829	35.83	0.66	C Vit	Yes	
N	K series	5.60	0.00997	14.59	0.80	BN	Yes	
O	K series	9.51	0.03200	32.80	0.52	SiO2	Yes	
Na	K	2.39	0.01009	4.70	0.12	Albite	Yes	

	series							
P	K series	0.21	0.00115	0.32	0.04	GaP	Yes	
S	K series	3.06	0.02638	6.95	0.14	FeS2	Yes	
Cl	K series	1.96	0.01712	4.82	0.12	NaCl	Yes	
Total:				100.00				

Electron Image 33

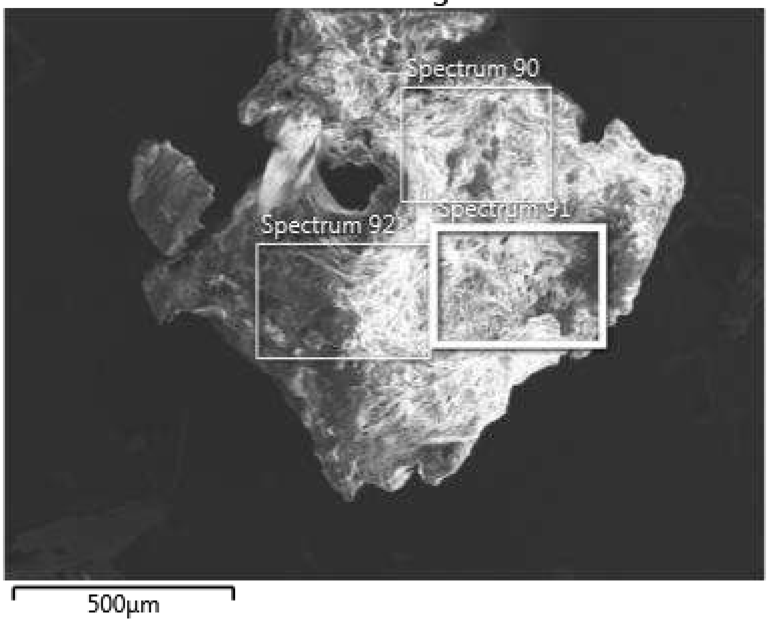

29/11/2019 10:26:59
Project 1
Cemento Mexico T2
CM3 15 Kv

Electron Image 33

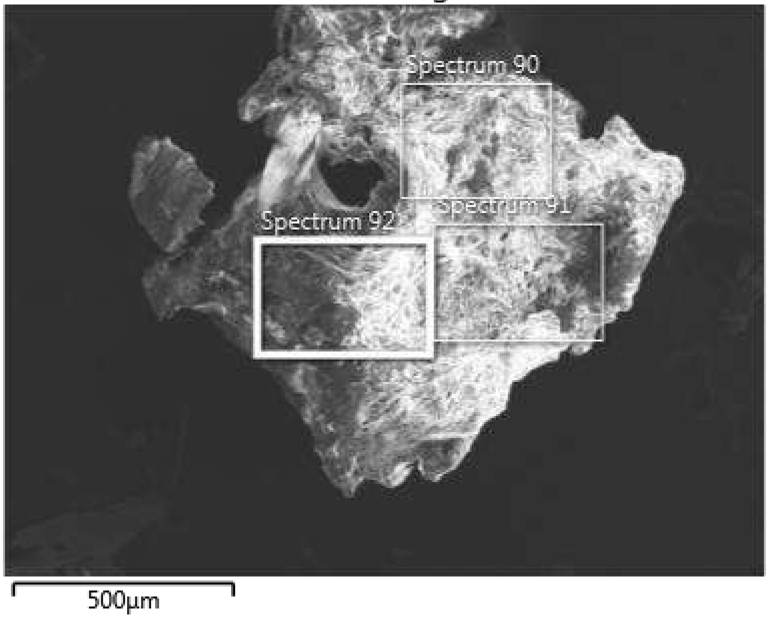

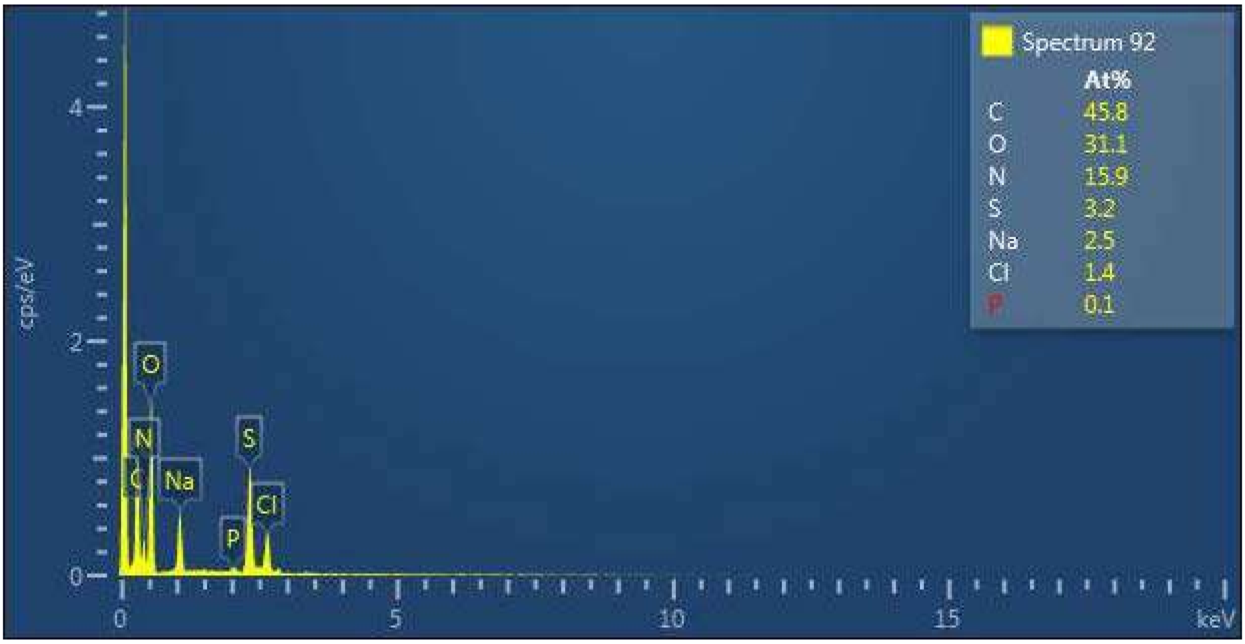

Label:	Spectrum 92
Source:	Acquired
Created:	29/11/2019 10:26:59
Livetime:	60.0s
Process Time:	4
Accelerating Voltage:	15.00kV
Magnification:	65 x
Working Distance:	9.9mm
Specimen Tilt (degrees):	0.0
Elevation (degrees):	35.0
Azimuth (degrees):	0.0
Number Of Channels:	2048
Energy Range (keV):	20 keV
Energy per Channel (eV):	10.0eV
Detector Type Id:	29
Detector Type:	X-Max
Window Type:	SATW
Pulse Pile Up Correction:	Succeeded
Primary Detector:	2617
Primary Detector Serial Number:	77871-X080

Element	Line Type	Apparent Concentration	k Ratio	Wt%	Wt% Sigma	Standard Label	Factory Standard	Standard Calibration Date
C	K series	1.97	0.01966	37.02	0.98	C Vit	Yes	
N	K series	2.61	0.00465	15.01	1.23	BN	Yes	
O	K series	4.34	0.01461	33.48	0.80	SiO2	Yes	
Na	K	0.88	0.00371	3.89	0.16	Albite	Yes	

	series							
P	K series	0.09	0.00049	0.30	0.06	GaP	Yes	
S	K series	1.36	0.01170	6.88	0.21	FeS2	Yes	
Cl	K series	0.62	0.00542	3.41	0.15	NaCl	Yes	
Total:				100.00				

Electron Image 33

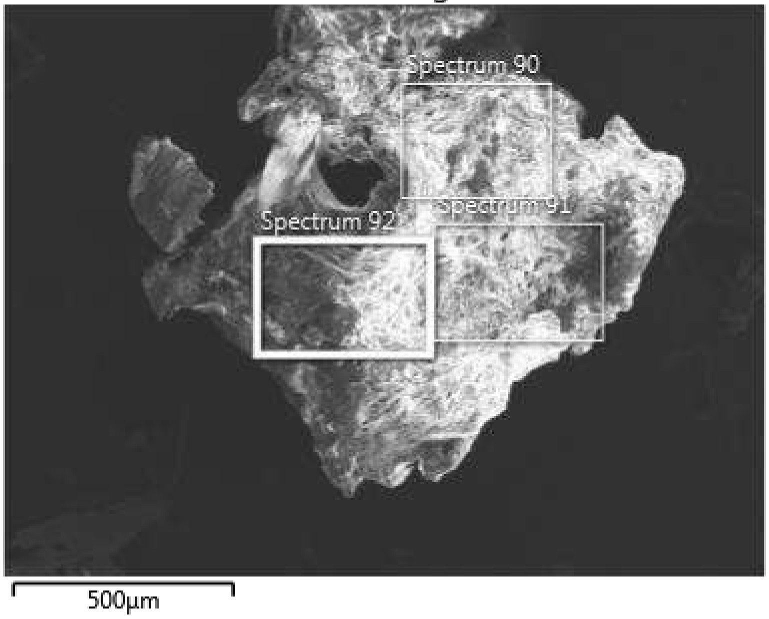

29/11/2019 10:29:46
Project 1
Cemento Mexico T3
CM1 15 Kv

Electron Image 34

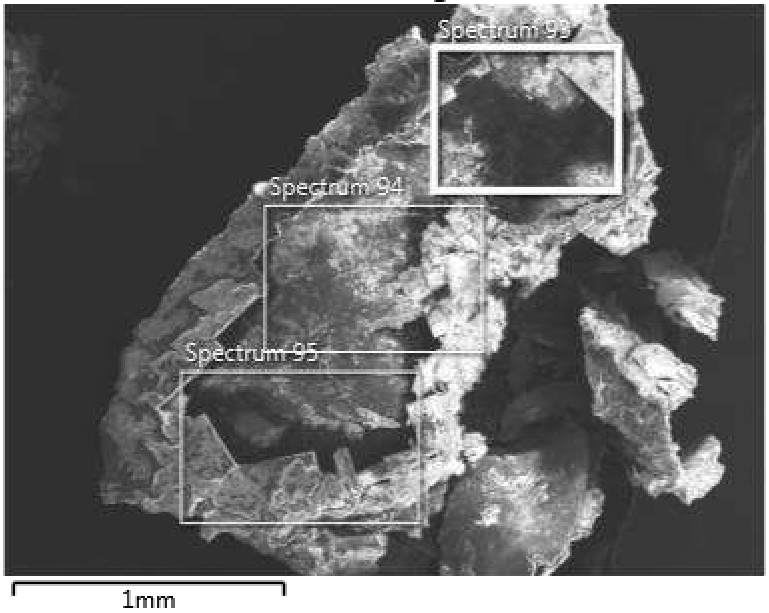

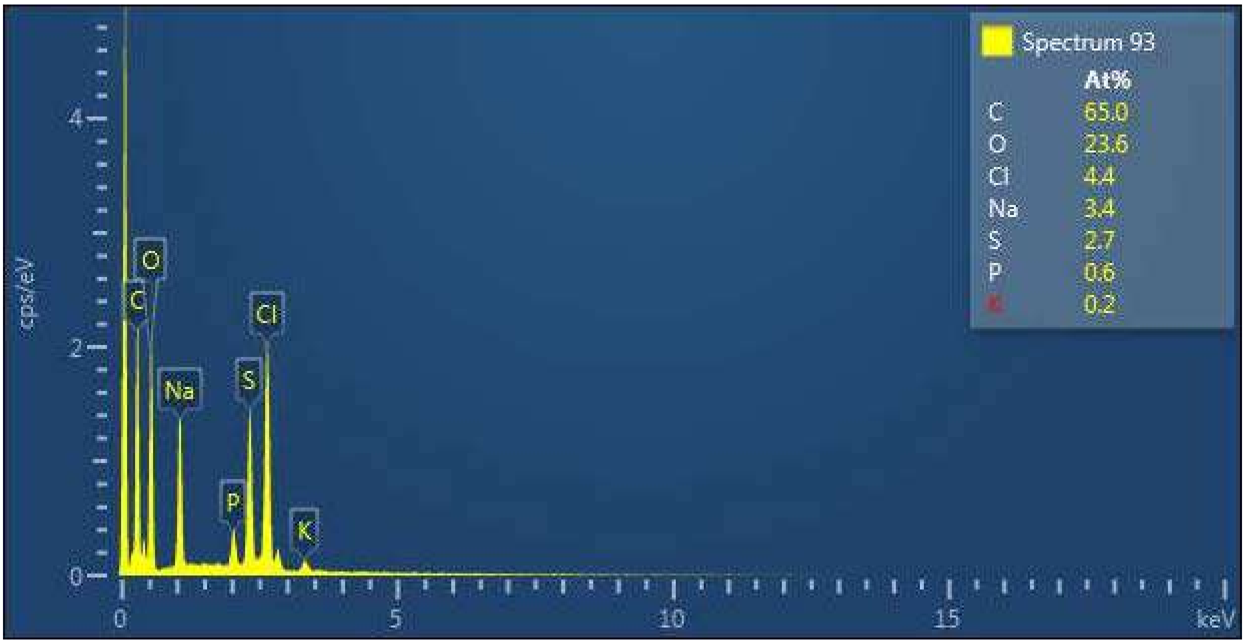

Label:	Spectrum 93
Source:	Acquired
Created:	29/11/2019 10:29:46
Livetime:	60.6s
Process Time:	4
Accelerating Voltage:	15.00kV
Magnification:	40 x
Working Distance:	9.9mm
Specimen Tilt (degrees):	0.0
Elevation (degrees):	35.0
Azimuth (degrees):	0.0
Number Of Channels:	2048
Energy Range (keV):	20 keV
Energy per Channel (eV):	10.0eV
Detector Type Id:	29
Detector Type:	X-Max
Window Type:	SATW
Pulse Pile Up Correction:	Succeeded
Primary Detector:	2617
Primary Detector Serial Number:	77871-X080

Element	Line Type	Apparent Concentration	k Ratio	Wt%	Wt% Sigma	Standard Label	Factory Standard	Standard Calibration Date
C	K series	3.54	0.03544	51.70	0.86	C Vit	Yes	
O	K series	5.81	0.01956	25.05	0.55	SiO2	Yes	
Na	K series	2.35	0.00992	5.25	0.15	Albite	Yes	
P	K	0.68	0.00380	1.27	0.07	GaP	Yes	

	series							
S	K series	2.09	0.01800	5.73	0.15	FeS2	Yes	
Cl	K series	3.48	0.03043	10.36	0.23	NaCl	Yes	
K	K series	0.22	0.00189	0.65	0.07	KBr	Yes	
Total:				100.00				

Electron Image 34

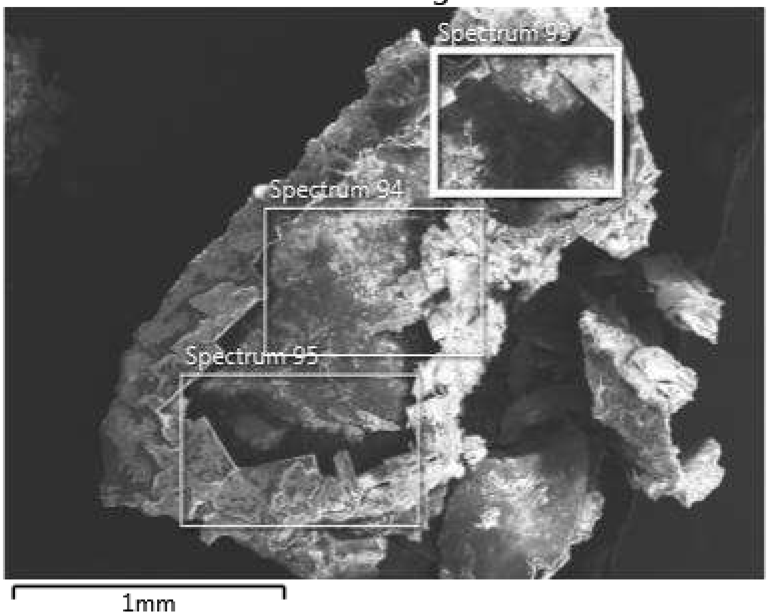

29/11/2019 10:30:54
Project 1
Cemento Mexico T3
CM1 15 Kv

Electron Image 34

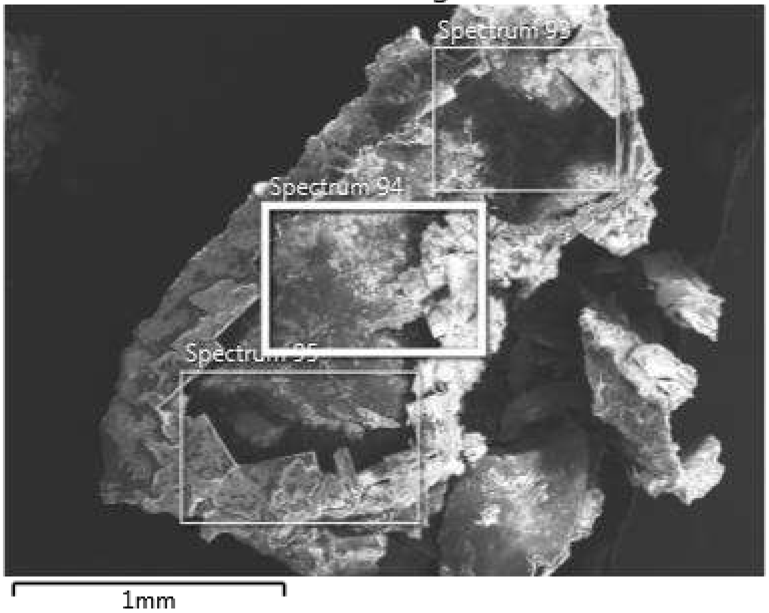

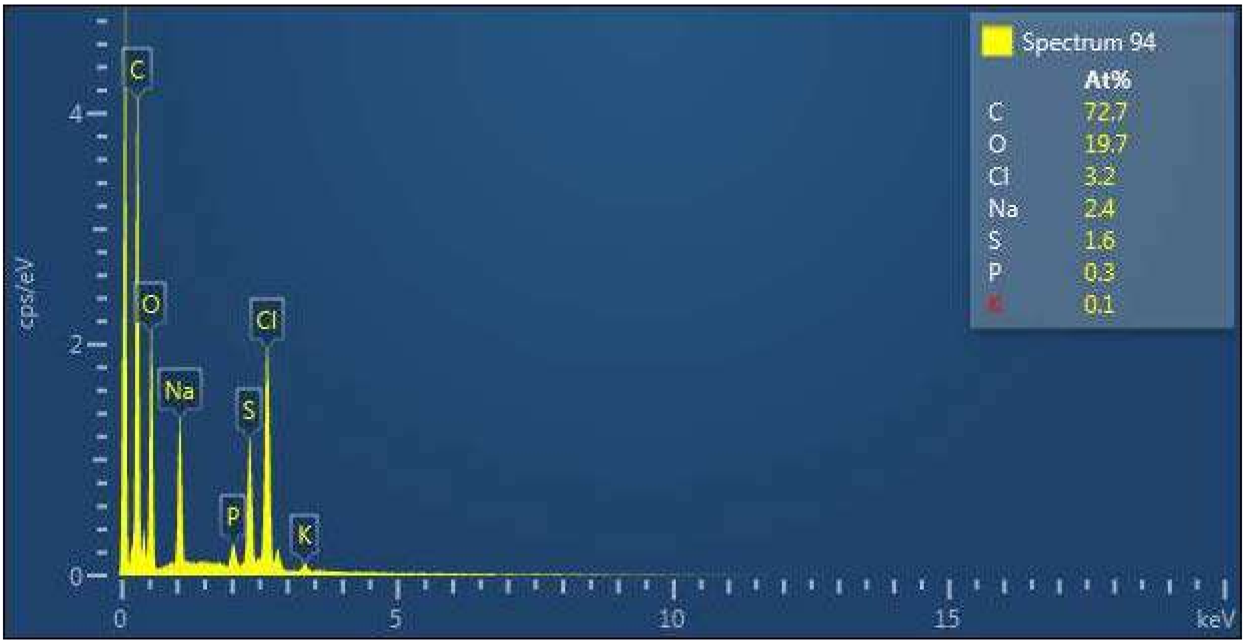

Label:	Spectrum 94
Source:	Acquired
Created:	29/11/2019 10:30:54
Livetime:	60.2s
Process Time:	4
Accelerating Voltage:	15.00kV
Magnification:	40 x
Working Distance:	9.9mm
Specimen Tilt (degrees):	0.0
Elevation (degrees):	35.0
Azimuth (degrees):	0.0
Number Of Channels:	2048
Energy Range (keV):	20 keV
Energy per Channel (eV):	10.0eV
Detector Type Id:	29
Detector Type:	X-Max
Window Type:	SATW
Pulse Pile Up Correction:	Succeeded
Primary Detector:	2617
Primary Detector Serial Number:	77871-X080

Element	Line Type	Apparent Concentration	k Ratio	Wt%	Wt% Sigma	Standard Label	Factory Standard	Standard Calibration Date
C	K series	7.20	0.07203	61.35	0.58	C Vit	Yes	
O	K series	6.16	0.02072	22.13	0.43	SiO2	Yes	
Na	K series	2.26	0.00953	3.94	0.10	Albite	Yes	
P	K	0.43	0.00238	0.63	0.05	GaP	Yes	

	series							
S	K series	1.72	0.01478	3.69	0.10	FeS2	Yes	
Cl	K series	3.41	0.02979	7.88	0.16	NaCl	Yes	
K	K series	0.17	0.00141	0.38	0.05	KBr	Yes	
Total:				100.00				

Electron Image 34

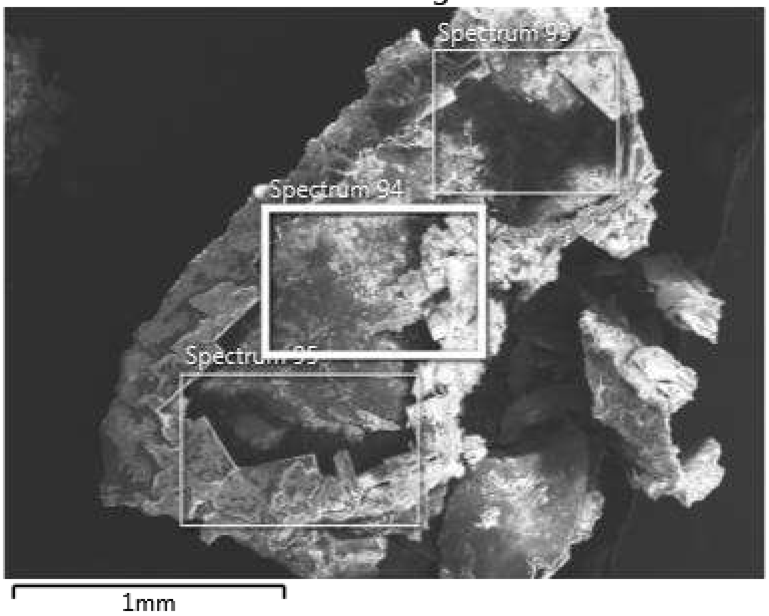

29/11/2019 10:32:02
Project 1
Cemento Mexico T3
CM1 15 Kv

Electron Image 34

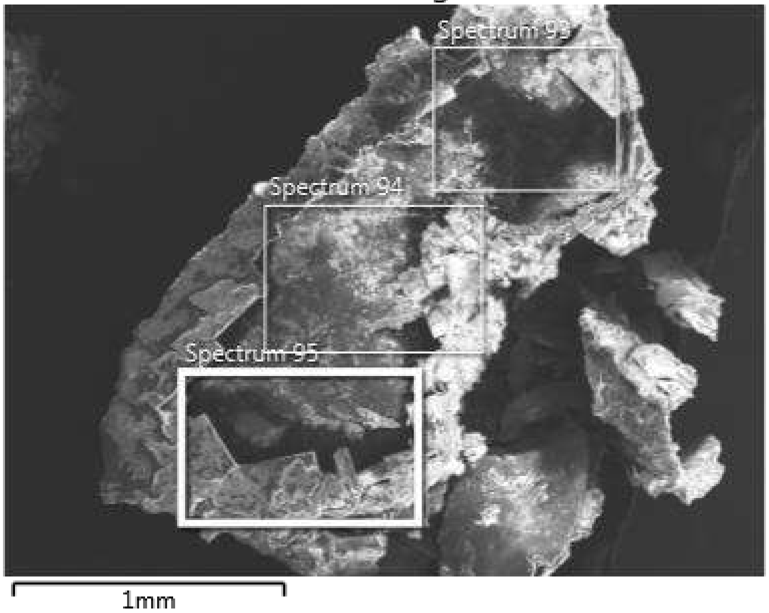

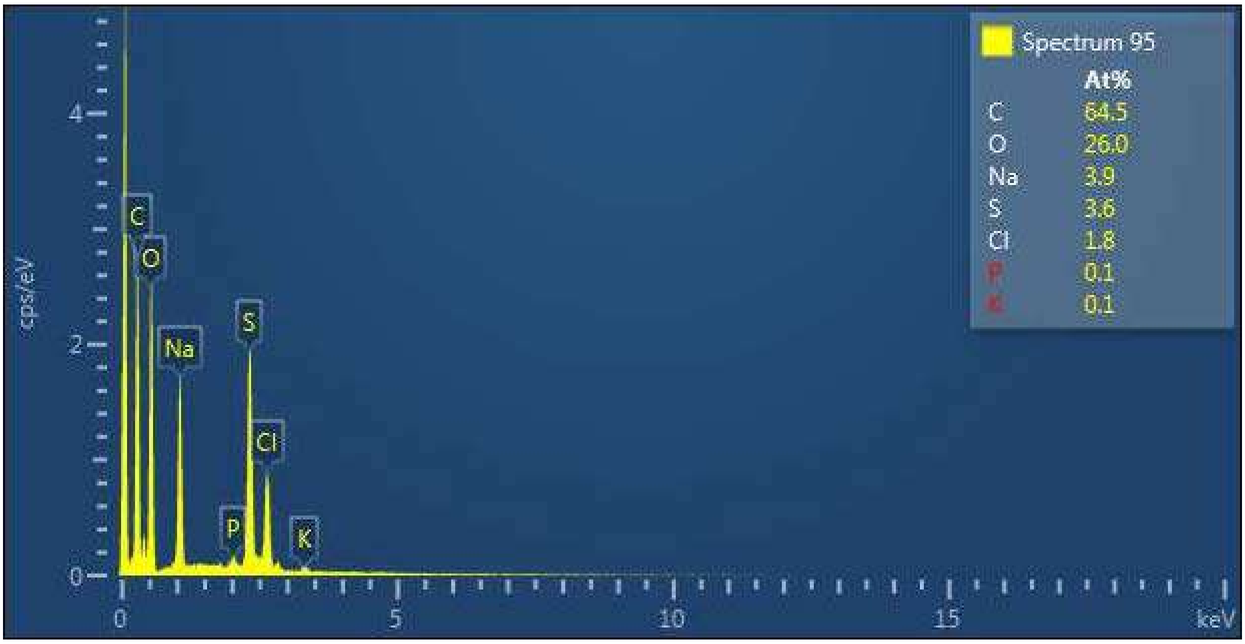

Label:	Spectrum 95
Source:	Acquired
Created:	29/11/2019 10:32:02
Livetime:	60.4s
Process Time:	4
Accelerating Voltage:	15.00kV
Magnification:	40 x
Working Distance:	9.9mm
Specimen Tilt (degrees):	0.0
Elevation (degrees):	35.0
Azimuth (degrees):	0.0
Number Of Channels:	2048
Energy Range (keV):	20 keV
Energy per Channel (eV):	10.0eV
Detector Type Id:	29
Detector Type:	X-Max
Window Type:	SATW
Pulse Pile Up Correction:	Succeeded
Primary Detector:	2617
Primary Detector Serial Number:	77871-X080

Element	Line Type	Apparent Concentration	k Ratio	Wt%	Wt% Sigma	Standard Label	Factory Standard	Standard Calibration Date
C	K series	4.88	0.04877	52.75	0.72	C Vit	Yes	
O	K series	7.40	0.02492	28.31	0.52	SiO2	Yes	
Na	K series	2.87	0.01211	6.13	0.15	Albite	Yes	
P	K	0.17	0.00097	0.31	0.05	GaP	Yes	

	series							
S	K series	3.01	0.02595	7.87	0.17	FeS2	Yes	
Cl	K series	1.52	0.01329	4.34	0.12	NaCl	Yes	
K	K series	0.11	0.00093	0.30	0.05	KBr	Yes	
Total:				100.00				

Electron Image 34

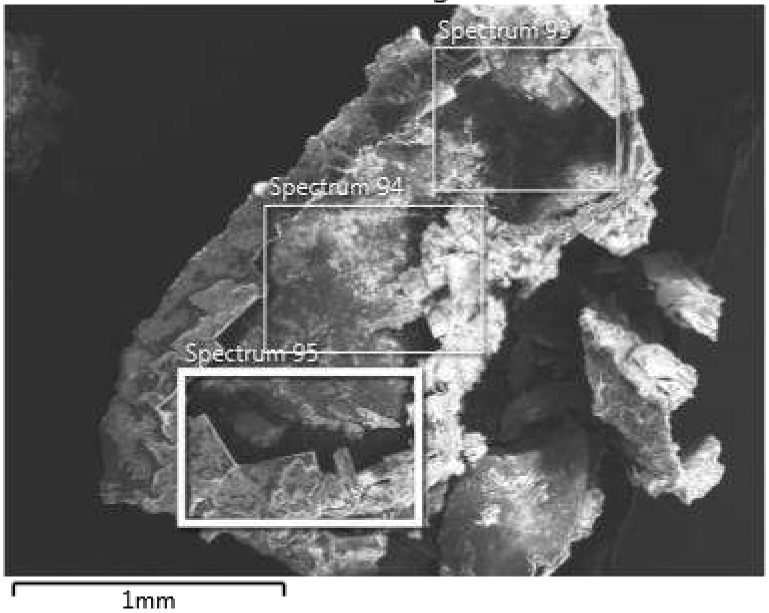

29/11/2019 10:38:02
Project 1
Cemento Mexico T3
CM2 15 Kv

Electron Image 35

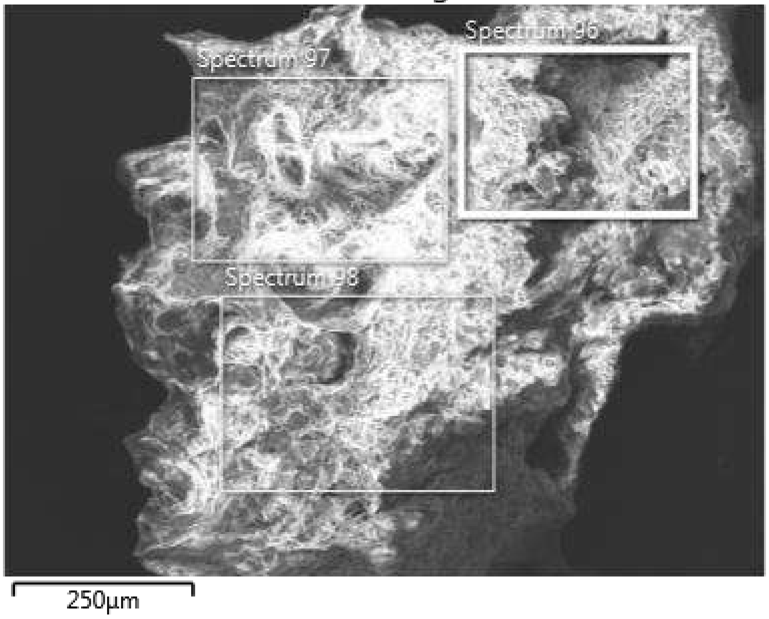

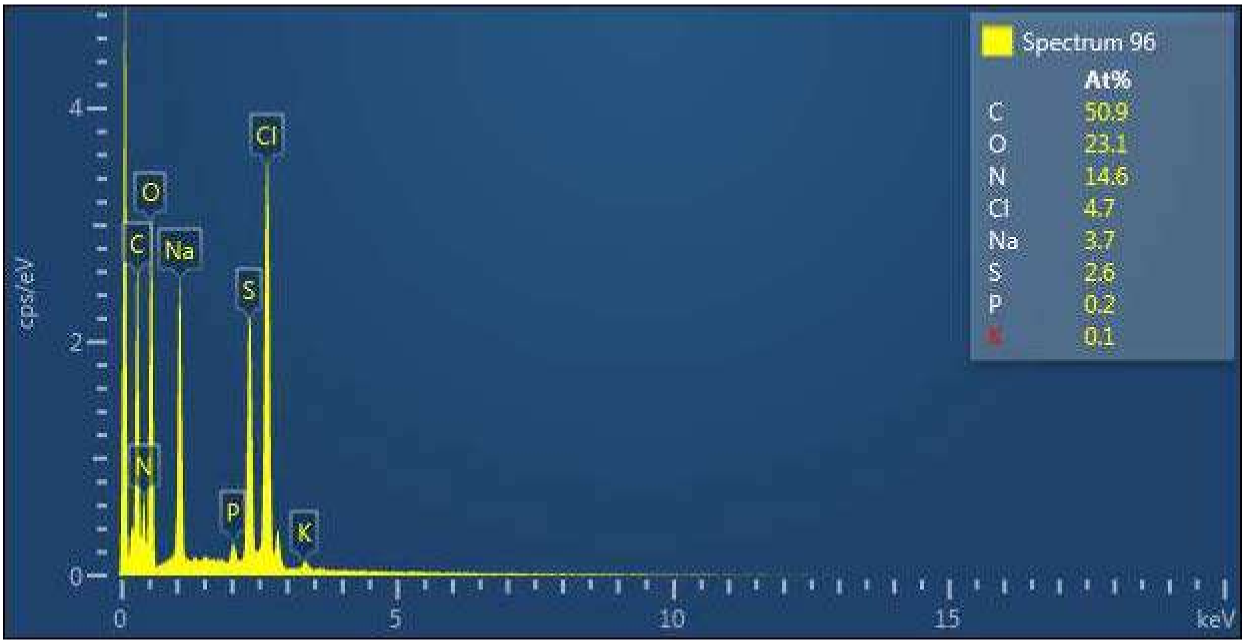

Label:	Spectrum 96
Source:	Acquired
Created:	29/11/2019 10:38:02
Livetime:	60.0s
Process Time:	4
Accelerating Voltage:	15.00kV
Magnification:	106 x
Working Distance:	9.9mm
Specimen Tilt (degrees):	0.0
Elevation (degrees):	35.0
Azimuth (degrees):	0.0
Number Of Channels:	2048
Energy Range (keV):	20 keV
Energy per Channel (eV):	10.0eV
Detector Type Id:	29
Detector Type:	X-Max
Window Type:	SATW
Pulse Pile Up Correction:	Succeeded
Primary Detector:	2617
Primary Detector Serial Number:	77871-X080

Element	Line Type	Apparent Concentration	k Ratio	Wt%	Wt% Sigma	Standard Label	Factory Standard	Standard Calibration Date
C	K series	4.71	0.04706	39.93	0.80	C Vit	Yes	
N	K series	6.01	0.01070	13.38	0.74	BN	Yes	
O	K series	8.83	0.02970	24.14	0.45	SiO2	Yes	
Na	K	4.16	0.01754	5.53	0.12	Albite	Yes	

	series							
P	K series	0.37	0.00206	0.40	0.04	GaP	Yes	
S	K series	3.45	0.02974	5.48	0.12	FeS2	Yes	
Cl	K series	6.30	0.05507	10.86	0.20	NaCl	Yes	
K	K series	0.17	0.00140	0.28	0.04	KBr	Yes	
Total:				100.00				

Electron Image 35

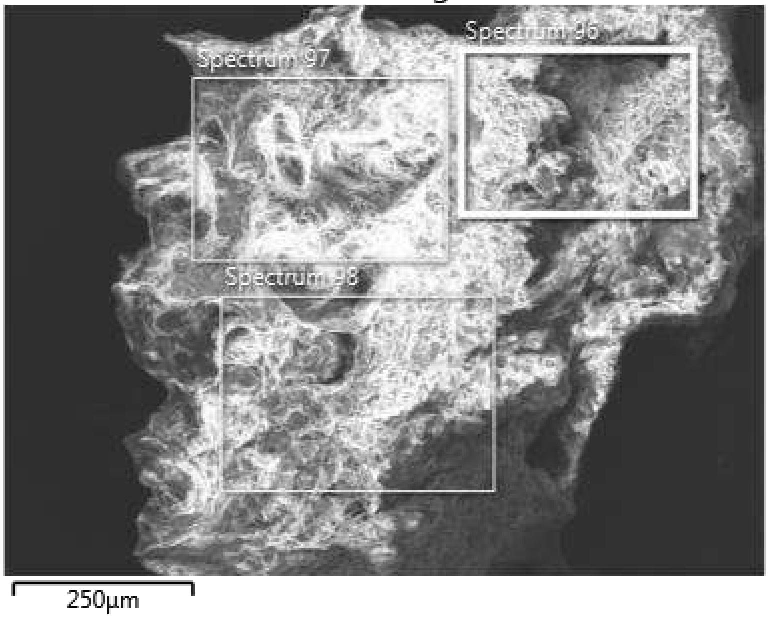

29/11/2019 10:39:09
Project 1
Cemento Mexico T3
CM2 15 Kv

Electron Image 35

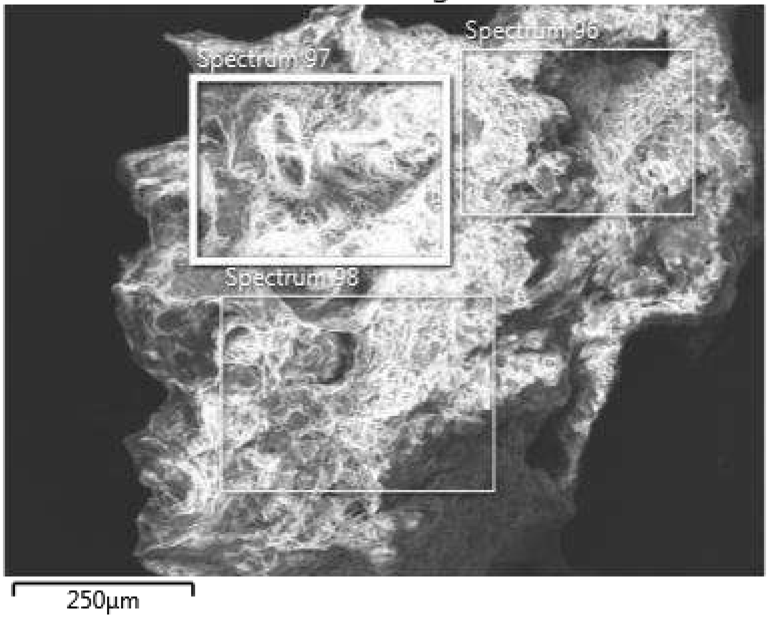

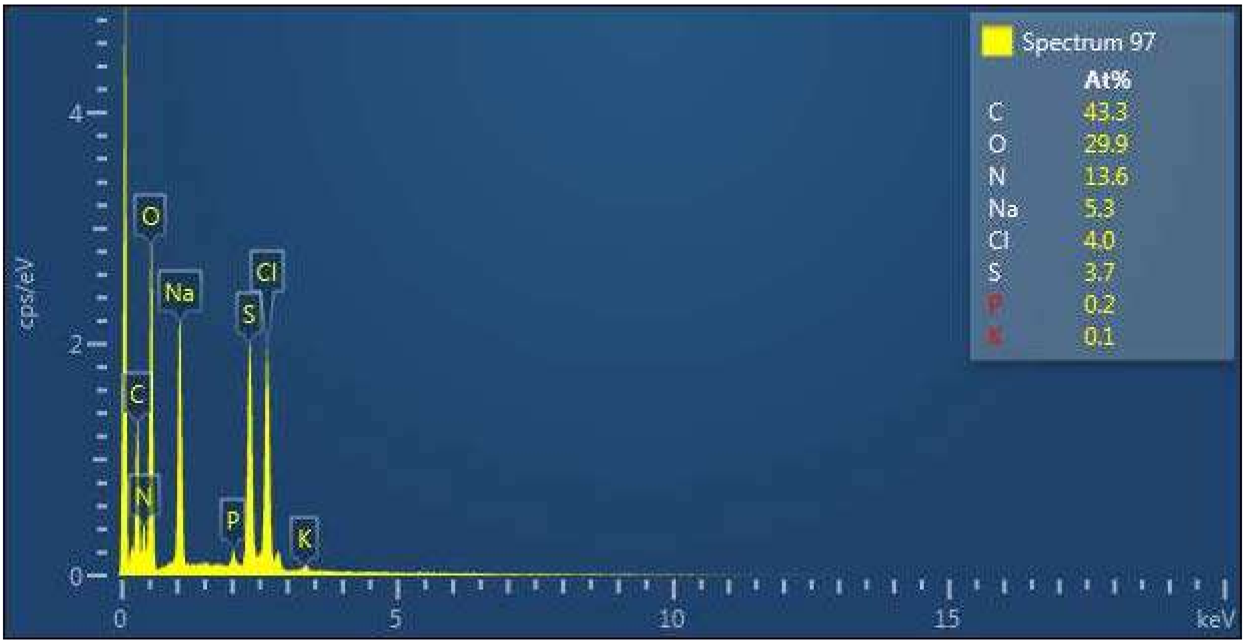

Label:	Spectrum 97
Source:	Acquired
Created:	29/11/2019 10:39:09
Livetime:	60.3s
Process Time:	4
Accelerating Voltage:	15.00kV
Magnification:	106 x
Working Distance:	9.9mm
Specimen Tilt (degrees):	0.0
Elevation (degrees):	35.0
Azimuth (degrees):	0.0
Number Of Channels:	2048
Energy Range (keV):	20 keV
Energy per Channel (eV):	10.0eV
Detector Type Id:	29
Detector Type:	X-Max
Window Type:	SATW
Pulse Pile Up Correction:	Succeeded
Primary Detector:	2617
Primary Detector Serial Number:	77871-X080

Element	Line Type	Apparent Concentration	k Ratio	Wt%	Wt% Sigma	Standard Label	Factory Standard	Standard Calibration Date
C	K series	2.46	0.02462	32.93	0.98	C Vit	Yes	
N	K series	4.03	0.00718	12.02	0.83	BN	Yes	
O	K series	8.20	0.02760	30.23	0.60	SiO2	Yes	
Na	K	3.75	0.01582	7.71	0.18	Albite	Yes	

	series							
P	K series	0.27	0.00150	0.45	0.05	GaP	Yes	
S	K series	3.04	0.02621	7.43	0.17	FeS2	Yes	
Cl	K series	3.36	0.02934	8.93	0.20	NaCl	Yes	
K	K series	0.11	0.00096	0.29	0.05	KBr	Yes	
Total:				100.00				

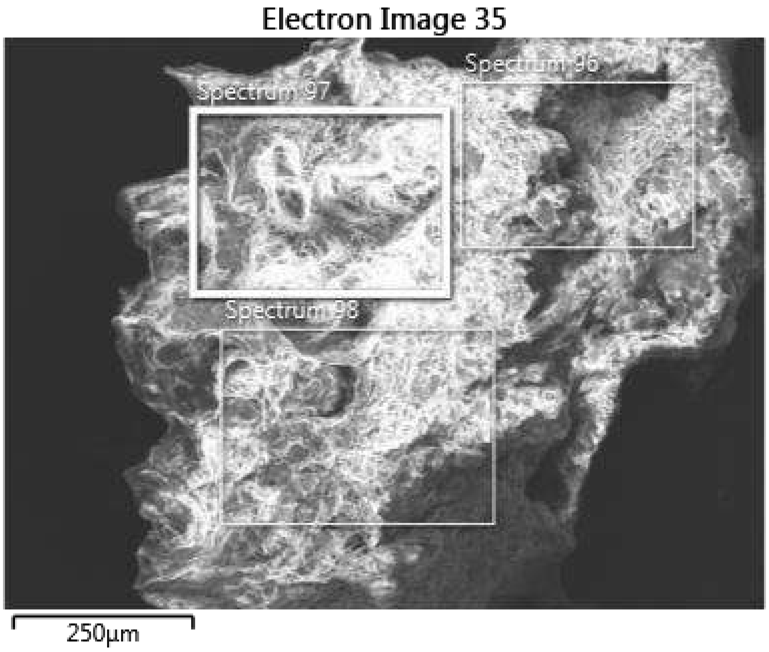

29/11/2019 10:40:16
Project 1
Cemento Mexico T3
CM2 15 Kv

Electron Image 35

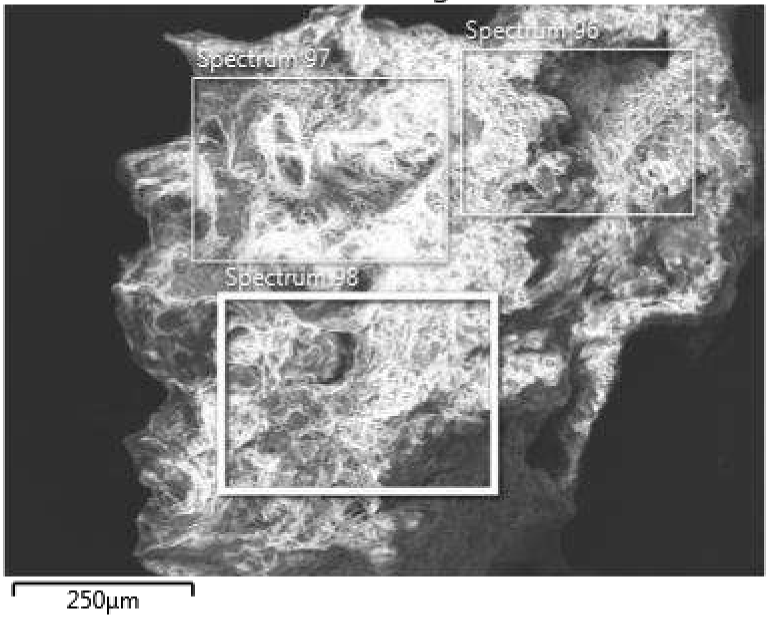

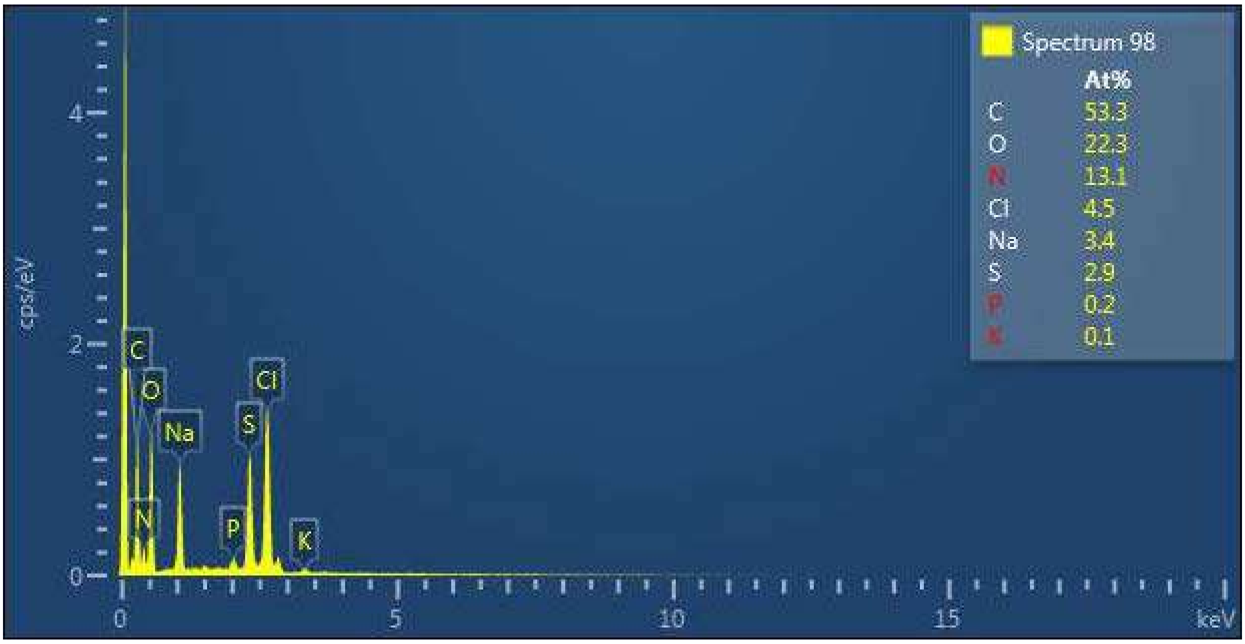

Label:	Spectrum 98
Source:	Acquired
Created:	29/11/2019 10:40:16
Livetime:	60.4s
Process Time:	4
Accelerating Voltage:	15.00kV
Magnification:	106 x
Working Distance:	9.9mm
Specimen Tilt (degrees):	0.0
Elevation (degrees):	35.0
Azimuth (degrees):	0.0
Number Of Channels:	2048
Energy Range (keV):	20 keV
Energy per Channel (eV):	10.0eV
Detector Type Id:	29
Detector Type:	X-Max
Window Type:	SATW
Pulse Pile Up Correction:	Succeeded
Primary Detector:	2617
Primary Detector Serial Number:	77871-X080

Element	Line Type	Apparent Concentration	k Ratio	Wt%	Wt% Sigma	Standard Label	Factory Standard	Standard Calibration Date
C	K series	2.05	0.02050	41.98	1.23	C Vit	Yes	
N	K series	2.15	0.00383	12.03	1.15	BN	Yes	
O	K series	3.54	0.01190	23.40	0.69	SiO2	Yes	
Na	K	1.61	0.00681	5.14	0.18	Albite	Yes	

	series							
P	K series	0.19	0.00107	0.50	0.07	GaP	Yes	
S	K series	1.58	0.01362	6.04	0.19	FeS2	Yes	
Cl	K series	2.54	0.02217	10.54	0.30	NaCl	Yes	
K	K series	0.09	0.00078	0.37	0.07	KBr	Yes	
Total:				100.00				

Electron Image 35

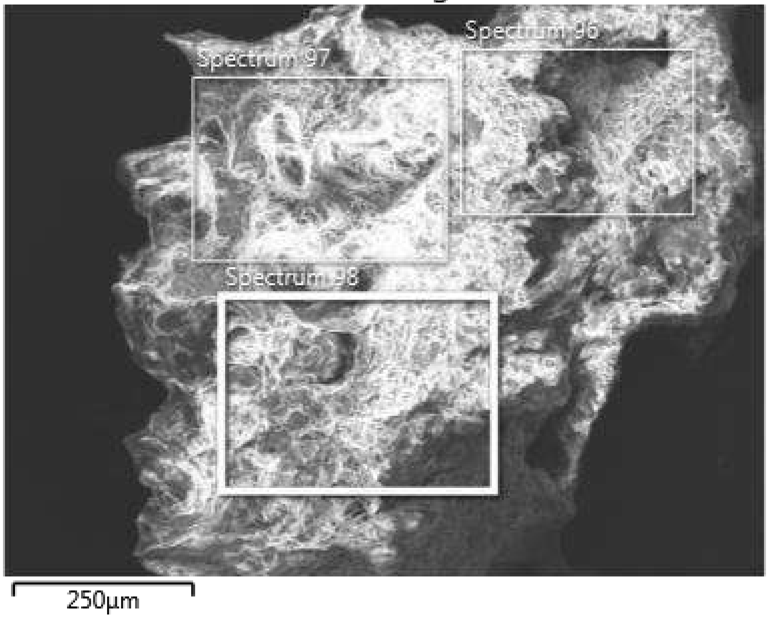

29/11/2019 10:44:46
Project 1
Cemento Mexico T3
CM3 15 Kv

Electron Image 36

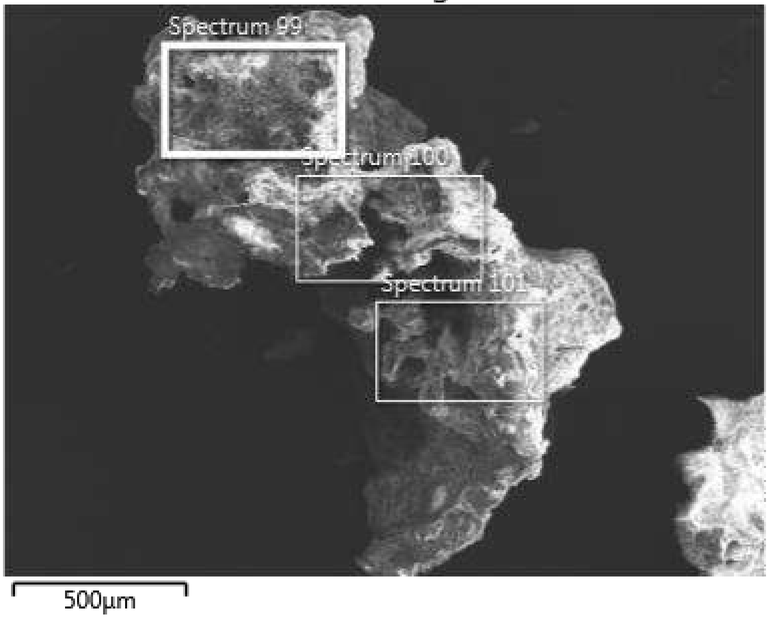

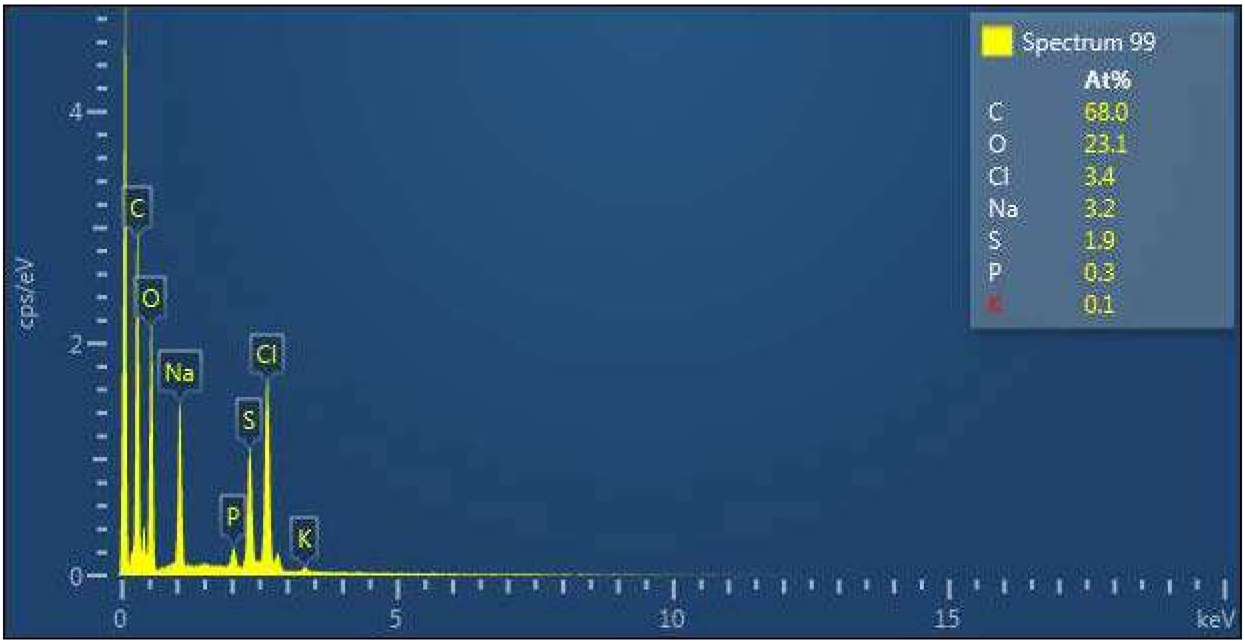

Label:	Spectrum 99
Source:	Acquired
Created:	29/11/2019 10:44:46
Livetime:	60.3s
Process Time:	4
Accelerating Voltage:	15.00kV
Magnification:	51 x
Working Distance:	9.9mm
Specimen Tilt (degrees):	0.0
Elevation (degrees):	35.0
Azimuth (degrees):	0.0
Number Of Channels:	2048
Energy Range (keV):	20 keV
Energy per Channel (eV):	10.0eV
Detector Type Id:	29
Detector Type:	X-Max
Window Type:	SATW
Pulse Pile Up Correction:	Succeeded
Primary Detector:	2617
Primary Detector Serial Number:	77871-X080

Element	Line Type	Apparent Concentration	k Ratio	Wt%	Wt% Sigma	Standard Label	Factory Standard	Standard Calibration Date
C	K series	5.12	0.05118	56.19	0.71	C Vit	Yes	
O	K series	6.30	0.02121	25.39	0.51	SiO2	Yes	
Na	K series	2.44	0.01029	5.12	0.13	Albite	Yes	
P	K	0.37	0.00207	0.65	0.05	GaP	Yes	

	series							
S	K series	1.62	0.01395	4.16	0.12	FeS2	Yes	
Cl	K series	2.98	0.02602	8.23	0.18	NaCl	Yes	
K	K series	0.09	0.00077	0.25	0.05	KBr	Yes	
Total:				100.00				

Electron Image 36

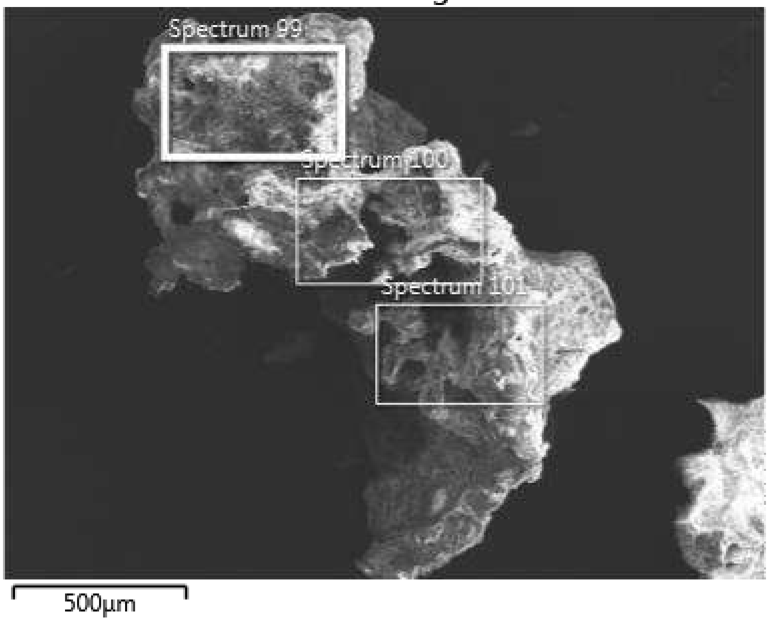

29/11/2019 10:45:54
Project 1
Cemento Mexico T3
CM3 15 Kv

Electron Image 36

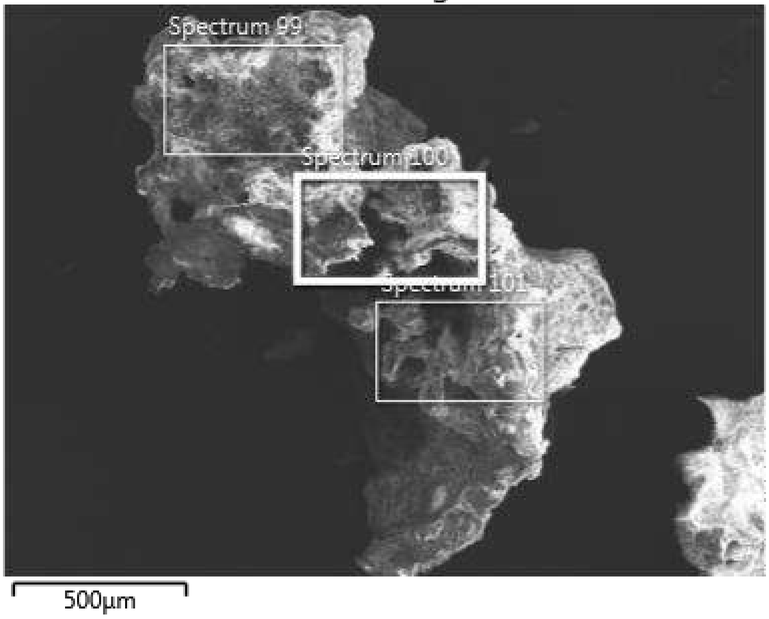

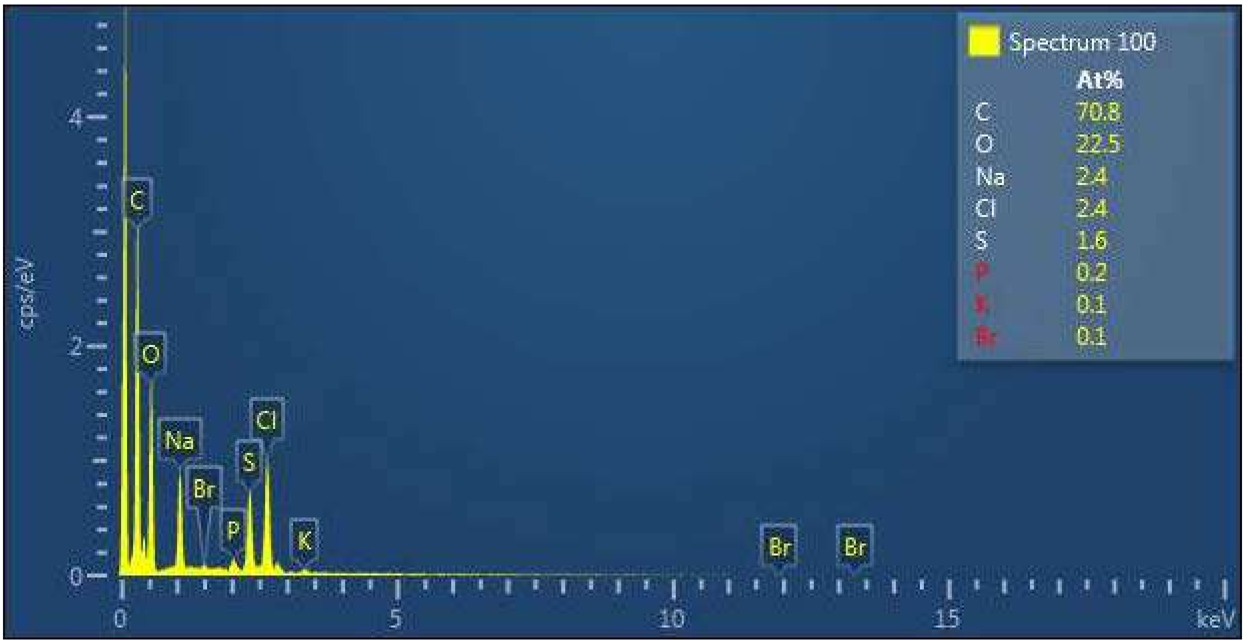

Label:	Spectrum 100
Source:	Acquired
Created:	29/11/2019 10:45:54
Livetime:	60.0s
Process Time:	4
Accelerating Voltage:	15.00kV
Magnification:	51 x
Working Distance:	9.9mm
Specimen Tilt (degrees):	0.0
Elevation (degrees):	35.0
Azimuth (degrees):	0.0
Number Of Channels:	2048
Energy Range (keV):	20 keV
Energy per Channel (eV):	10.0eV
Detector Type Id:	29
Detector Type:	X-Max
Window Type:	SATW
Pulse Pile Up Correction:	Succeeded
Primary Detector:	2617
Primary Detector Serial Number:	77871-X080

Element	Line Type	Apparent Concentration	k Ratio	Wt%	Wt% Sigma	Standard Label	Factory Standard	Standard Calibration Date
C	K series	5.36	0.05358	60.14	0.70	C Vit	Yes	
O	K series	4.98	0.01675	25.44	0.56	SiO2	Yes	
Na	K series	1.50	0.00633	3.98	0.13	Albite	Yes	
P	K	0.20	0.00109	0.43	0.05	GaP	Yes	

	series							
S	K series	1.09	0.00942	3.54	0.12	FeS2	Yes	
Cl	K series	1.72	0.01501	5.94	0.16	NaCl	Yes	
K	K series	0.07	0.00063	0.25	0.05	KBr	Yes	
Br	L series	0.07	0.00062	0.29	0.09	KBr	Yes	
Total:				100.00				

Electron Image 36

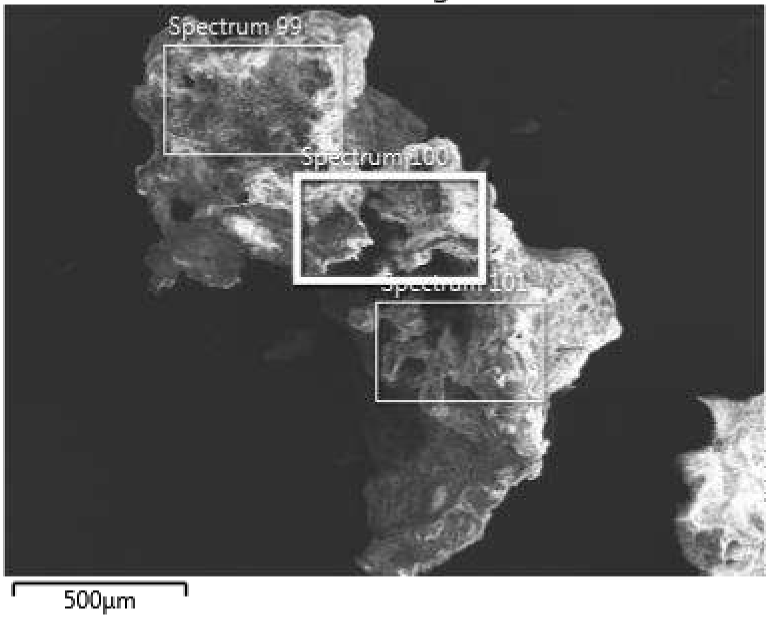

29/11/2019 10:47:02
Project 1
Cemento Mexico T3
CM3 15 Kv

Electron Image 36

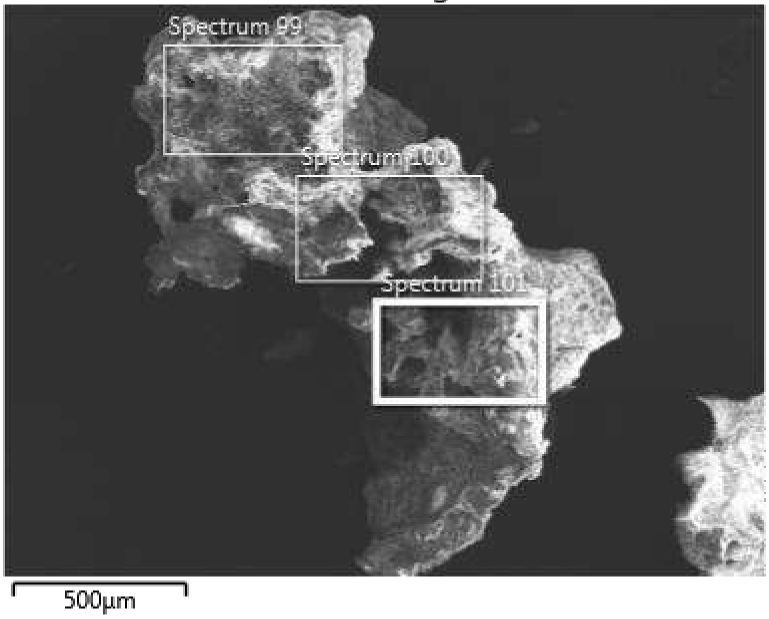

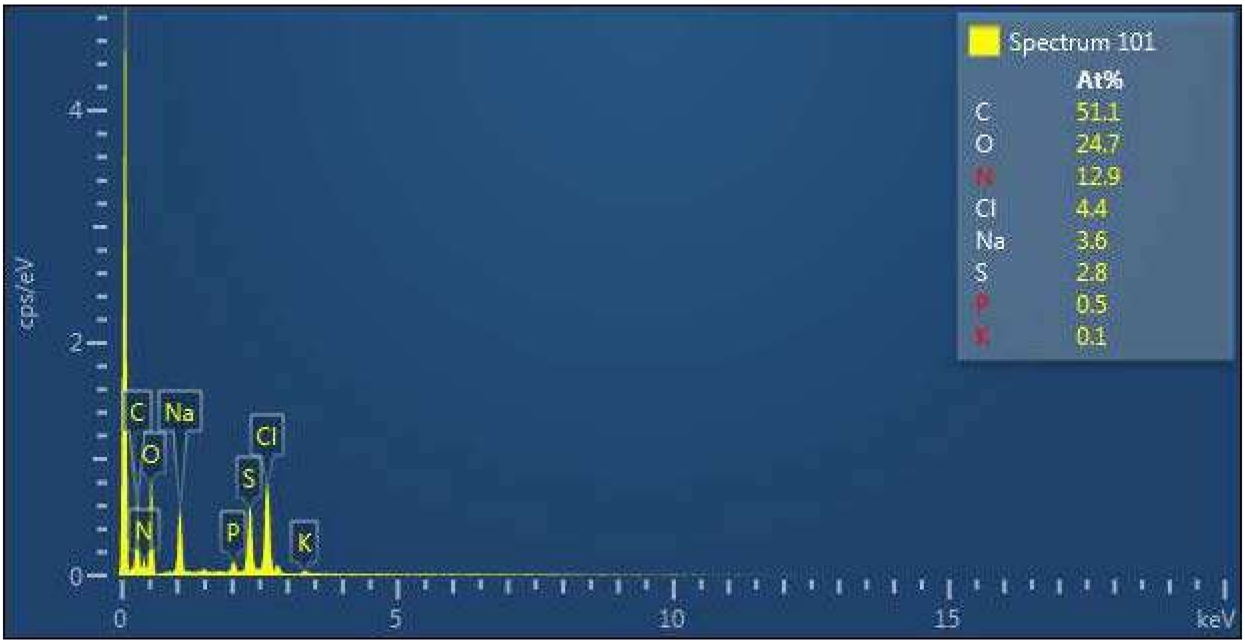

Label:	Spectrum 101
Source:	Acquired
Created:	29/11/2019 10:47:02
Livetime:	60.2s
Process Time:	4
Accelerating Voltage:	15.00kV
Magnification:	51 x
Working Distance:	9.9mm
Specimen Tilt (degrees):	0.0
Elevation (degrees):	35.0
Azimuth (degrees):	0.0
Number Of Channels:	2048
Energy Range (keV):	20 keV
Energy per Channel (eV):	10.0eV
Detector Type Id:	29
Detector Type:	X-Max
Window Type:	SATW
Pulse Pile Up Correction:	Succeeded
Primary Detector:	2617
Primary Detector Serial Number:	77871-X080

Element	Line Type	Apparent Concentration	k Ratio	Wt%	Wt% Sigma	Standard Label	Factory Standard	Standard Calibration Date
C	K series	1.14	0.01139	39.95	1.61	C Vit	Yes	
N	K series	1.28	0.00228	11.78	1.45	BN	Yes	
O	K series	2.34	0.00789	25.74	0.93	SiO2	Yes	
Na	K	0.96	0.00406	5.32	0.24	Albite	Yes	

	series							
P	K series	0.20	0.00113	0.92	0.10	GaP	Yes	
S	K series	0.88	0.00760	5.82	0.25	FeS2	Yes	
Cl	K series	1.42	0.01240	10.16	0.38	NaCl	Yes	
K	K series	0.05	0.00039	0.32	0.10	KBr	Yes	
Total:				100.00				

Electron Image 36

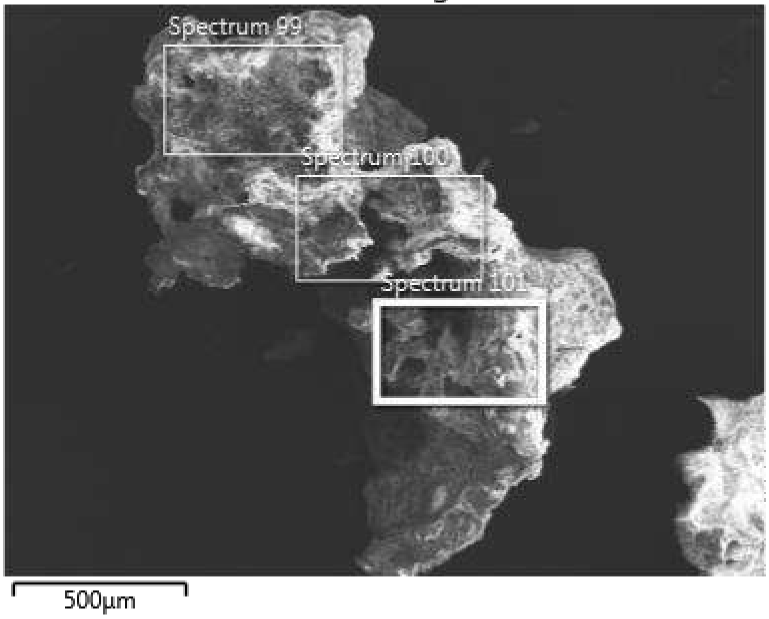

29/11/2019 10:48:59
Project 1
Cemento Mexico T3
CM4 15 Kv

Electron Image 37

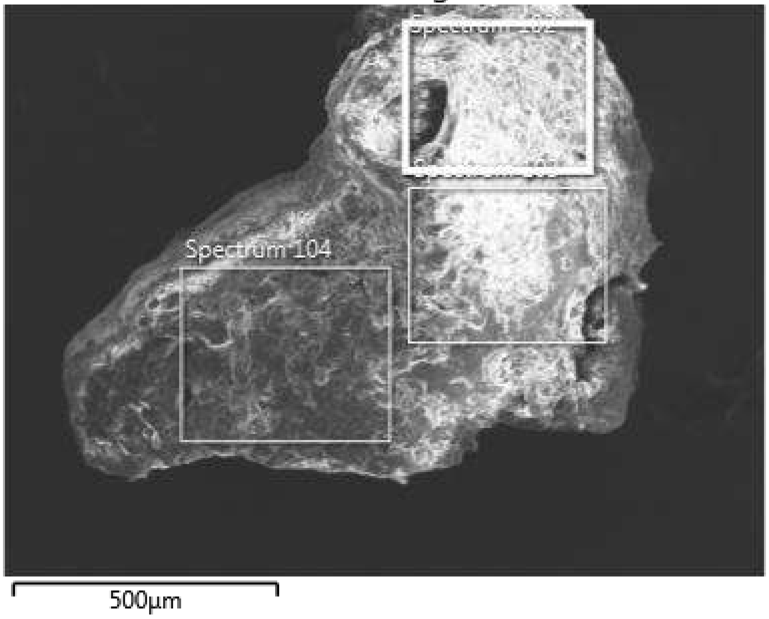

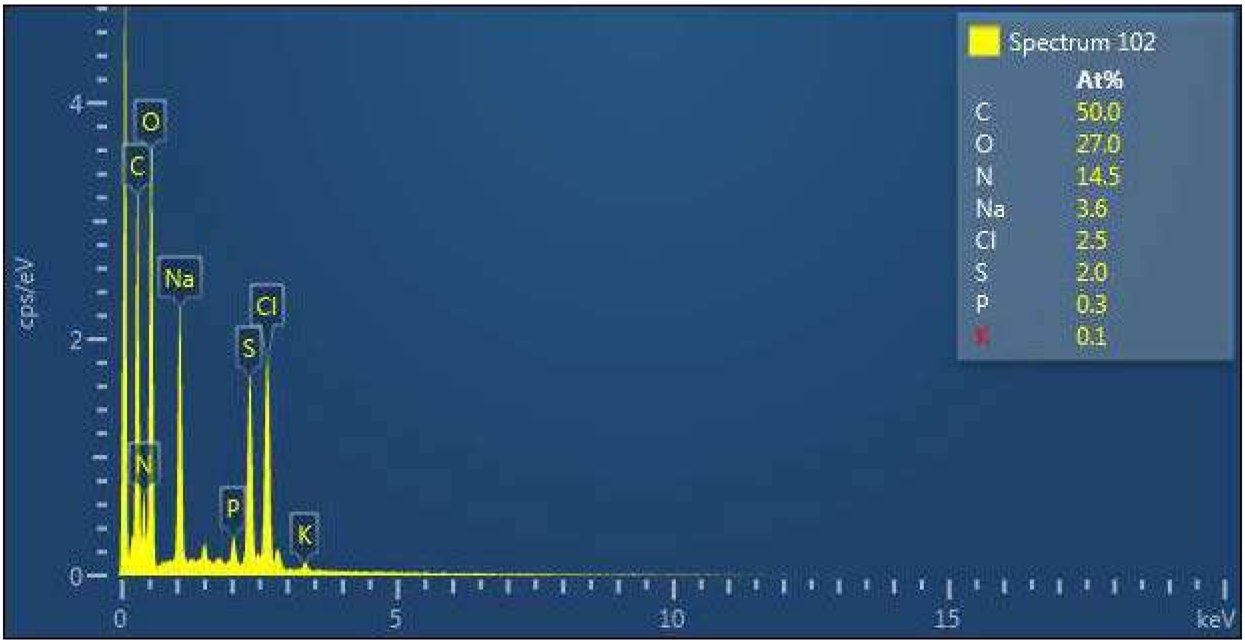

Label:	Spectrum 102
Source:	Acquired
Created:	29/11/2019 10:48:59
Livetime:	60.1s
Process Time:	4
Accelerating Voltage:	15.00kV
Magnification:	78 x
Working Distance:	9.9mm
Specimen Tilt (degrees):	0.0
Elevation (degrees):	35.0
Azimuth (degrees):	0.0
Number Of Channels:	2048
Energy Range (keV):	20 keV
Energy per Channel (eV):	10.0eV
Detector Type Id:	29
Detector Type:	X-Max
Window Type:	SATW
Pulse Pile Up Correction:	Succeeded
Primary Detector:	2617
Primary Detector Serial Number:	77871-X080

Element	Line Type	Apparent Concentration	k Ratio	Wt%	Wt% Sigma	Standard Label	Factory Standard	Standard Calibration Date
C	K series	5.97	0.05970	40.43	0.72	C Vit	Yes	
N	K series	6.27	0.01117	13.66	0.75	BN	Yes	
O	K series	10.54	0.03546	29.07	0.48	SiO2	Yes	
Na	K	3.77	0.01592	5.55	0.12	Albite	Yes	

	series							
P	K series	0.50	0.00279	0.59	0.04	GaP	Yes	
S	K series	2.53	0.02182	4.40	0.10	FeS2	Yes	
Cl	K series	3.24	0.02829	6.02	0.13	NaCl	Yes	
K	K series	0.15	0.00130	0.27	0.04	KBr	Yes	
Total:				100.00				

Electron Image 37

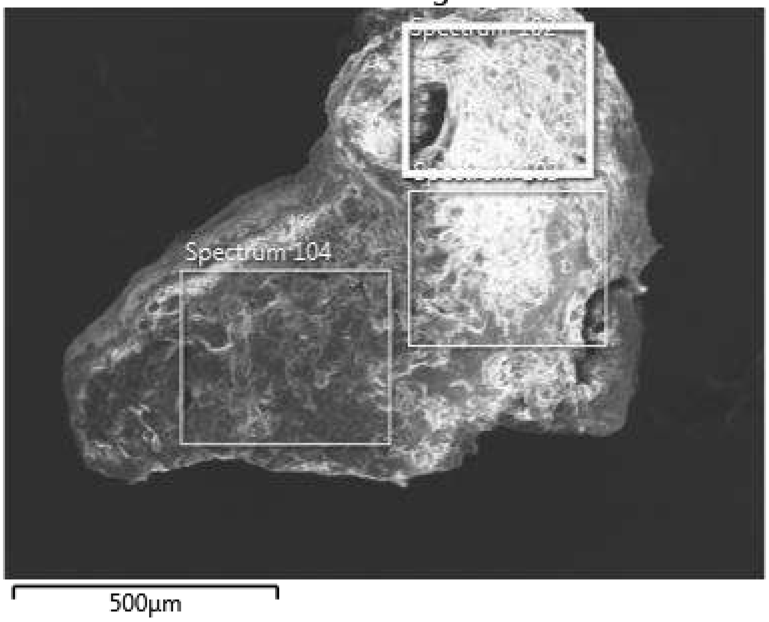

29/11/2019 10:50:07
Project 1
Cemento Mexico T3
CM4 15 Kv

Electron Image 37

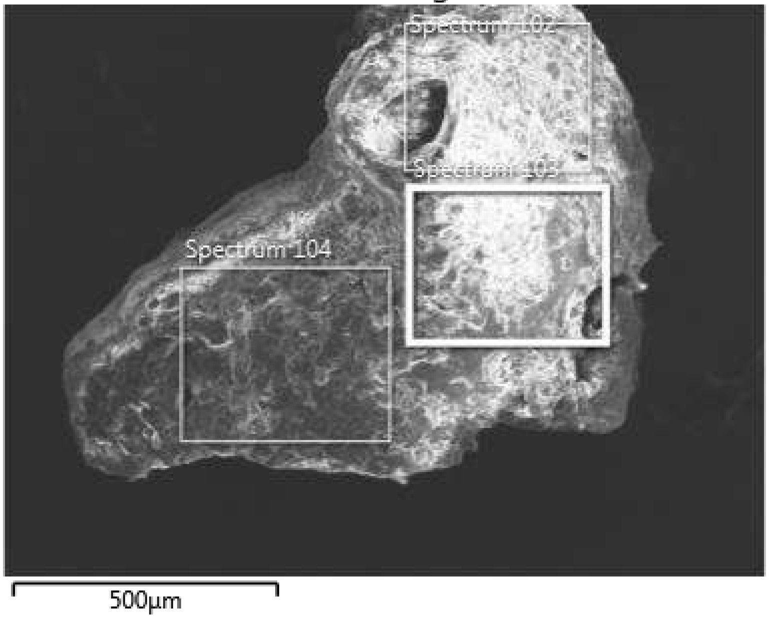

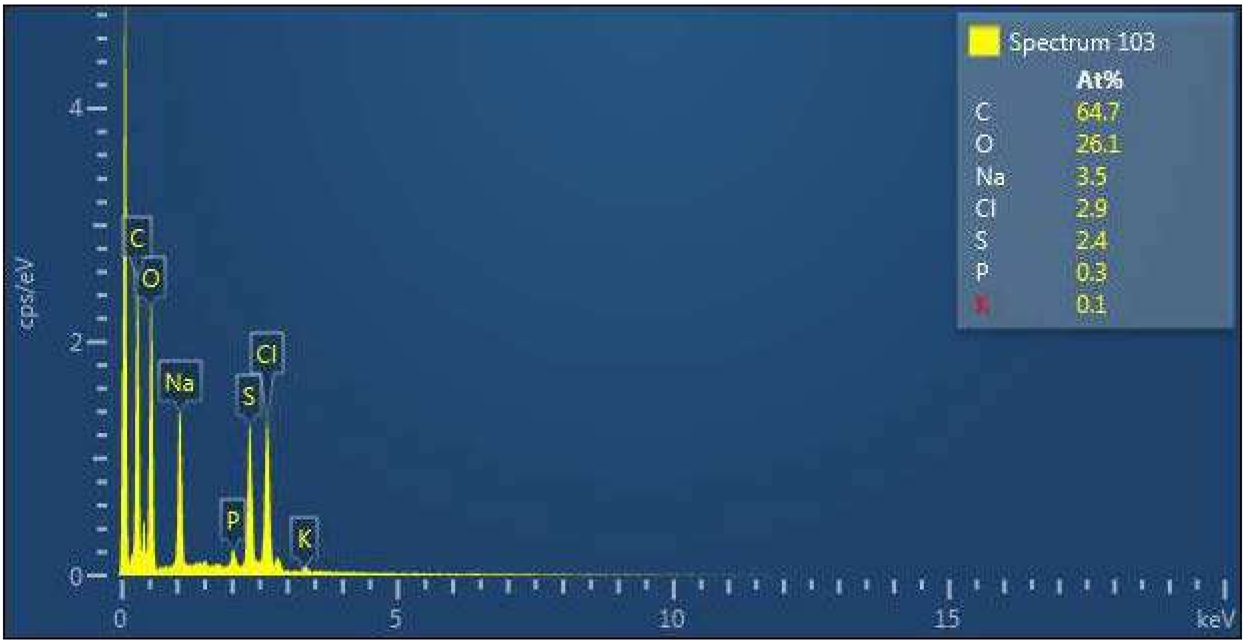

Label:	Spectrum 103
Source:	Acquired
Created:	29/11/2019 10:50:07
Livetime:	60.1s
Process Time:	4
Accelerating Voltage:	15.00kV
Magnification:	78 x
Working Distance:	9.9mm
Specimen Tilt (degrees):	0.0
Elevation (degrees):	35.0
Azimuth (degrees):	0.0
Number Of Channels:	2048
Energy Range (keV):	20 keV
Energy per Channel (eV):	10.0eV
Detector Type Id:	29
Detector Type:	X-Max
Window Type:	SATW
Pulse Pile Up Correction:	Succeeded
Primary Detector:	2617
Primary Detector Serial Number:	77871-X080

Element	Line Type	Apparent Concentration	k Ratio	Wt%	Wt% Sigma	Standard Label	Factory Standard	Standard Calibration Date
C	K series	4.40	0.04401	52.89	0.78	C Vit	Yes	
O	K series	6.76	0.02274	28.39	0.57	SiO2	Yes	
Na	K series	2.37	0.01001	5.53	0.15	Albite	Yes	
P	K	0.32	0.00176	0.61	0.06	GaP	Yes	

	series							
S	K series	1.88	0.01618	5.32	0.14	FeS2	Yes	
Cl	K series	2.26	0.01973	6.90	0.17	NaCl	Yes	
K	K series	0.12	0.00106	0.37	0.06	KBr	Yes	
Total:				100.00				

Electron Image 37

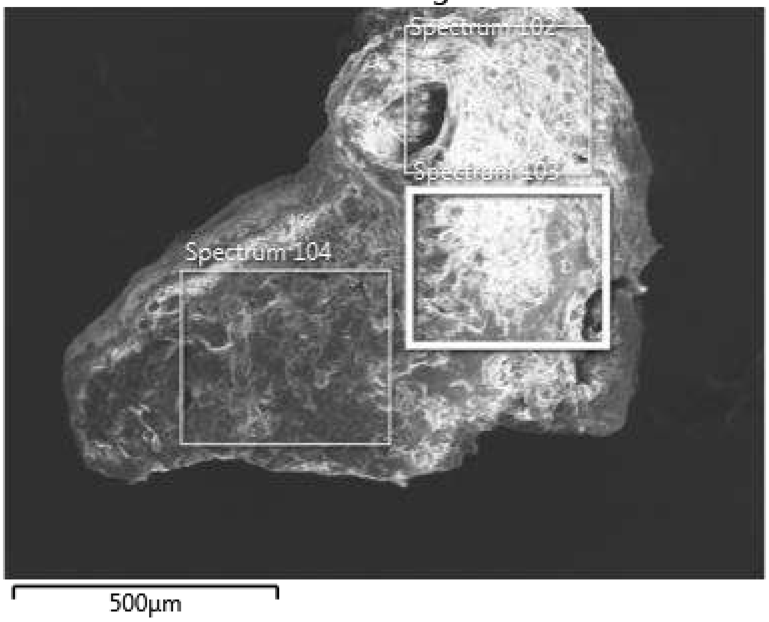

29/11/2019 10:51:15
Project 1
Cemento Mexico T3
CM4 15 Kv

Electron Image 37

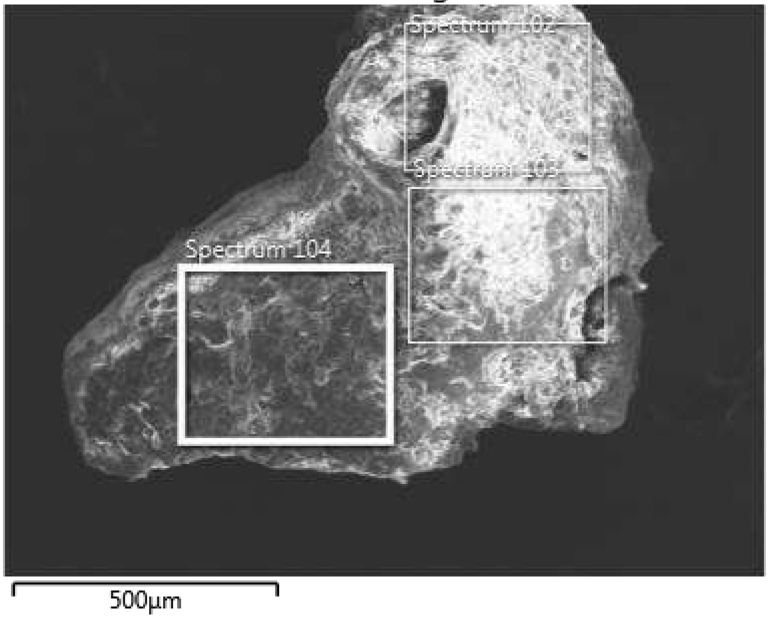

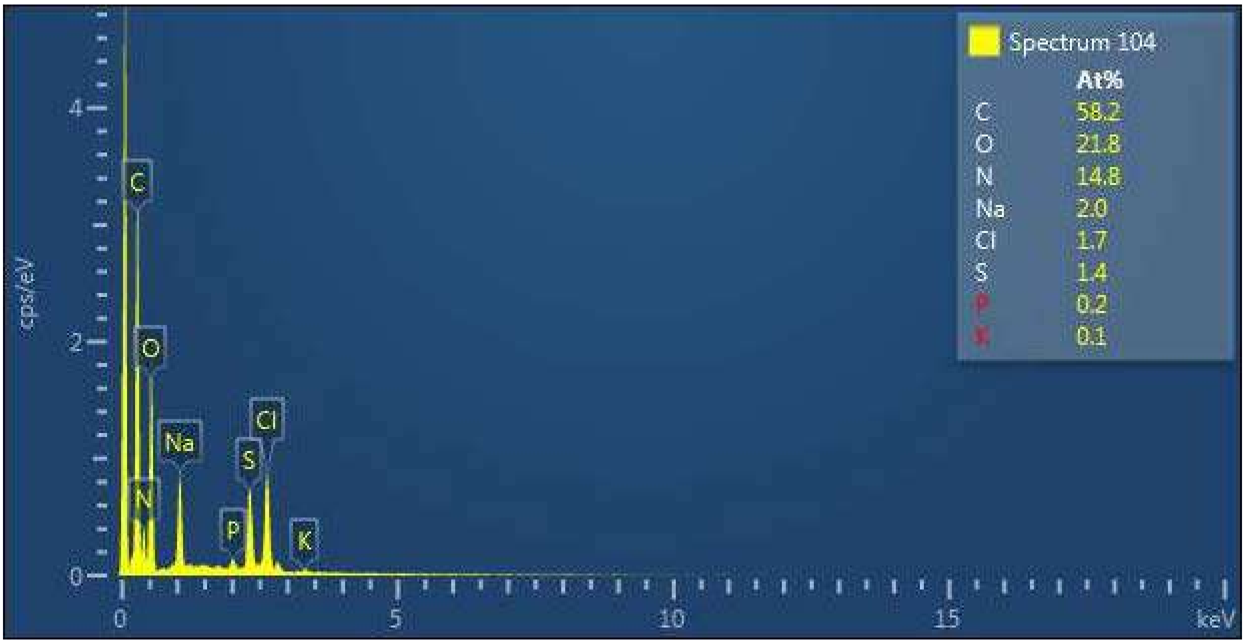

Label:	Spectrum 104
Source:	Acquired
Created:	29/11/2019 10:51:15
Livetime:	60.5s
Process Time:	4
Accelerating Voltage:	15.00kV
Magnification:	78 x
Working Distance:	9.9mm
Specimen Tilt (degrees):	0.0
Elevation (degrees):	35.0
Azimuth (degrees):	0.0
Number Of Channels:	2048
Energy Range (keV):	20 keV
Energy per Channel (eV):	10.0eV
Detector Type Id:	29
Detector Type:	X-Max
Window Type:	SATW
Pulse Pile Up Correction:	Succeeded
Primary Detector:	2617
Primary Detector Serial Number:	77871-X080

Element	Line Type	Apparent Concentration	k Ratio	Wt%	Wt% Sigma	Standard Label	Factory Standard	Standard Calibration Date
C	K series	6.00	0.06005	49.52	0.87	C Vit	Yes	
N	K series	3.79	0.00675	14.66	1.02	BN	Yes	
O	K series	5.08	0.01711	24.69	0.56	SiO2	Yes	
Na	K	1.37	0.00578	3.21	0.11	Albite	Yes	

	series							
P	K series	0.18	0.00100	0.34	0.05	GaP	Yes	
S	K series	1.12	0.00961	3.09	0.10	FeS2	Yes	
Cl	K series	1.47	0.01283	4.33	0.13	NaCl	Yes	
K	K series	0.06	0.00047	0.16	0.05	KBr	Yes	
Total:				100.00				

Electron Image 37

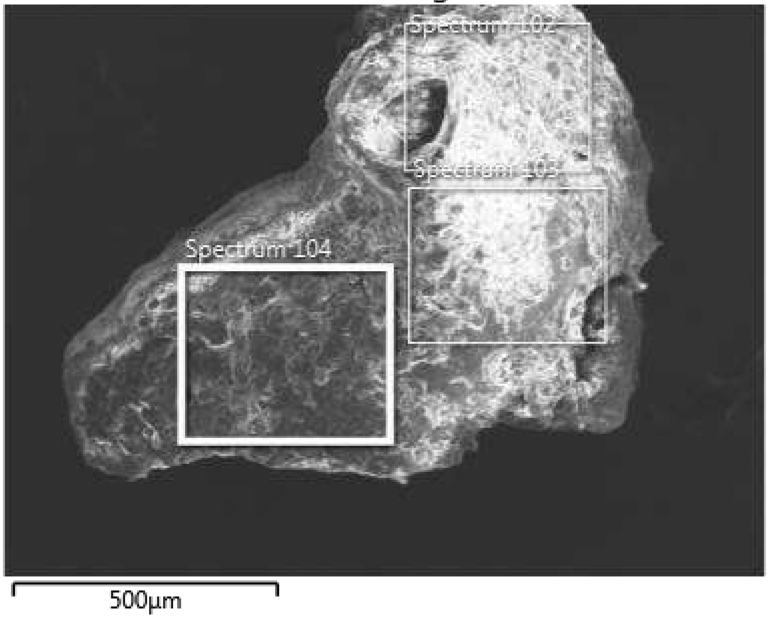

29/11/2019 12:13:20
Project 1
Glandulas salivares Mexico T1
GSM1 15 Kv

Electron Image 38

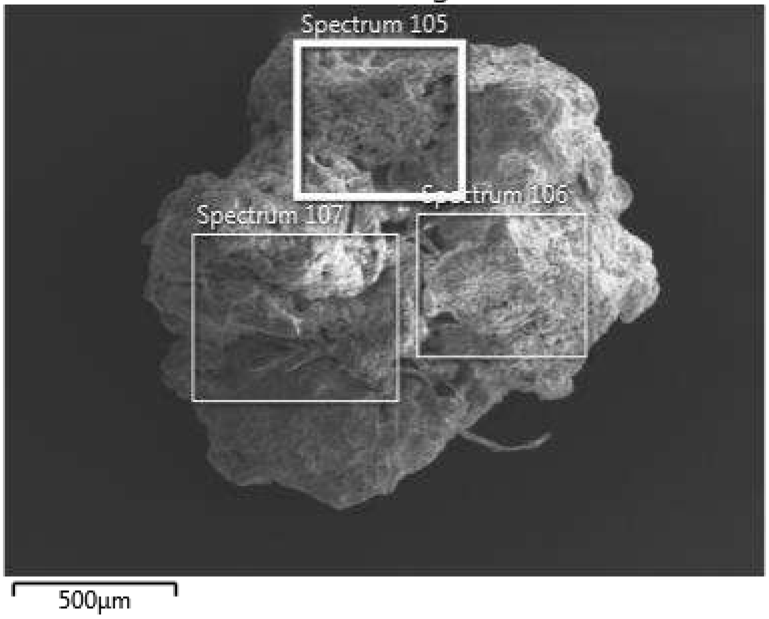

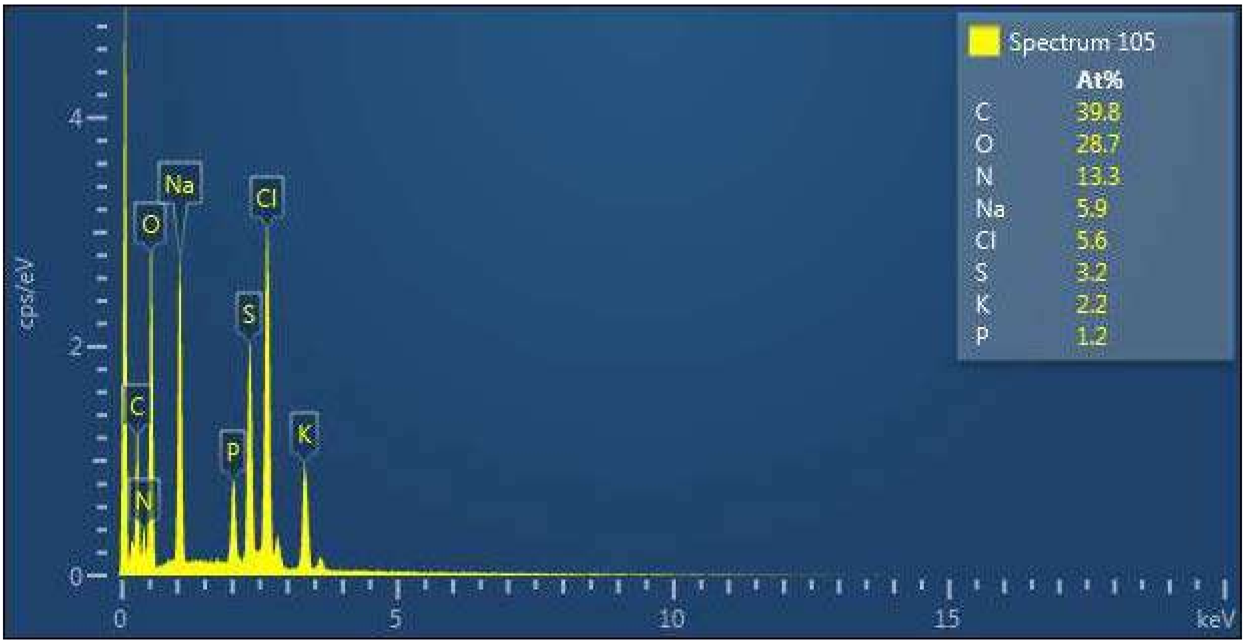

Label:	Spectrum 105
Source:	Acquired
Created:	29/11/2019 12:13:20
Livetime:	60.4s
Process Time:	4
Accelerating Voltage:	15.00kV
Magnification:	48 x
Working Distance:	9.8mm
Specimen Tilt (degrees):	0.0
Elevation (degrees):	35.0
Azimuth (degrees):	0.0
Number Of Channels:	2048
Energy Range (keV):	20 keV
Energy per Channel (eV):	10.0eV
Detector Type Id:	29
Detector Type:	X-Max
Window Type:	SATW
Pulse Pile Up Correction:	Succeeded
Primary Detector:	2617
Primary Detector Serial Number:	77871-X080

Element	Line Type	Apparent Concentration	k Ratio	Wt%	Wt% Sigma	Standard Label	Factory Standard	Standard Calibration Date
C	K series	2.22	0.02216	28.38	0.95	C Vit	Yes	
N	K series	3.98	0.00709	11.07	0.75	BN	Yes	
O	K series	8.26	0.02779	27.28	0.52	SiO2	Yes	
Na	K	4.67	0.01970	8.02	0.17	Albite	Yes	

	series							
P	K series	1.58	0.00886	2.23	0.08	GaP	Yes	
S	K series	3.00	0.02584	6.18	0.14	FeS2	Yes	
Cl	K series	5.29	0.04623	11.75	0.23	NaCl	Yes	
K	K series	2.36	0.02000	5.09	0.13	KBr	Yes	
Total:				100.00				

Electron Image 38

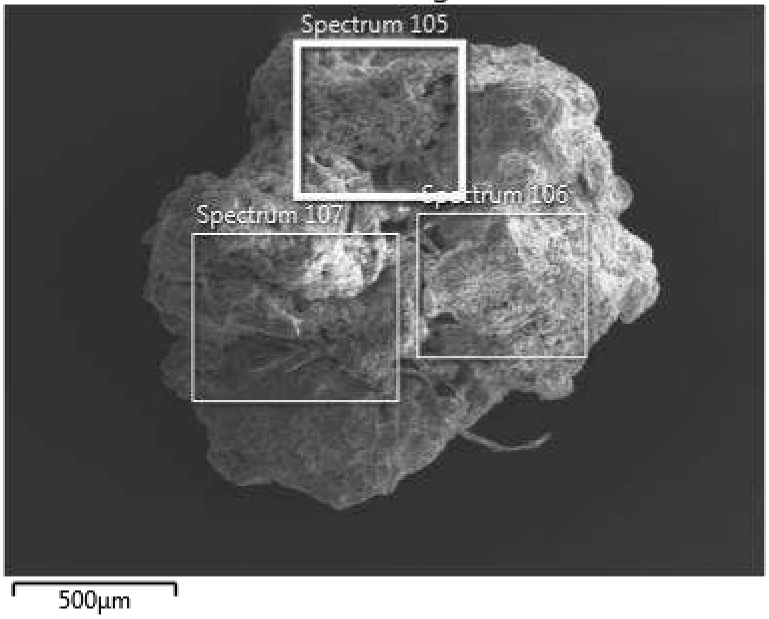

29/11/2019 12:14:29
Project 1
Glandulas salivares Mexico T1
GSM1 15 Kv

Electron Image 38

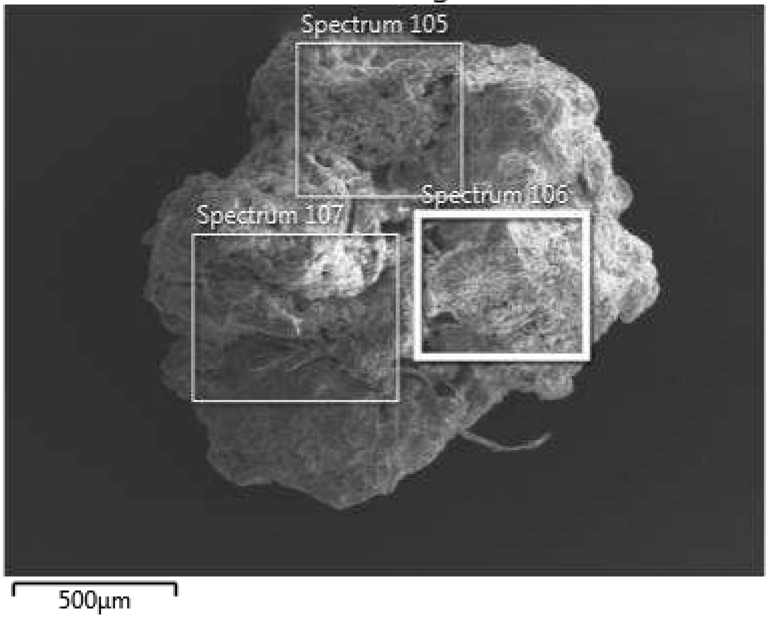

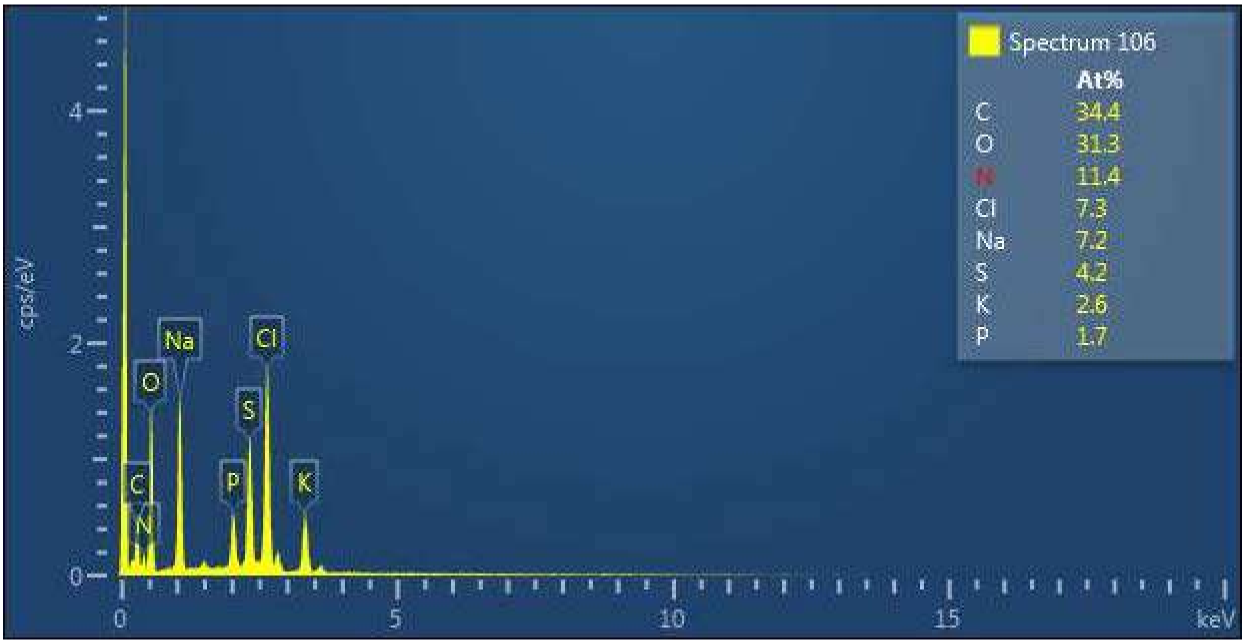

Label:	Spectrum 106
Source:	Acquired
Created:	29/11/2019 12:14:29
Livetime:	60.1s
Process Time:	4
Accelerating Voltage:	15.00kV
Magnification:	48 x
Working Distance:	9.8mm
Specimen Tilt (degrees):	0.0
Elevation (degrees):	35.0
Azimuth (degrees):	0.0
Number Of Channels:	2048
Energy Range (keV):	20 keV
Energy per Channel (eV):	10.0eV
Detector Type Id:	29
Detector Type:	X-Max
Window Type:	SATW
Pulse Pile Up Correction:	Succeeded
Primary Detector:	2617
Primary Detector Serial Number:	77871-X080

Element	Line Type	Apparent Concentration	k Ratio	Wt%	Wt% Sigma	Standard Label	Factory Standard	Standard Calibration Date
C	K series	0.72	0.00717	23.12	1.63	C Vit	Yes	
N	K series	1.53	0.00272	8.91	1.07	BN	Yes	
O	K series	4.20	0.01414	28.03	0.81	SiO2	Yes	
Na	K	2.59	0.01095	9.26	0.29	Albite	Yes	

	series							
P	K series	1.00	0.00561	2.95	0.14	GaP	Yes	
S	K series	1.74	0.01496	7.49	0.25	FeS2	Yes	
Cl	K series	3.09	0.02703	14.48	0.41	NaCl	Yes	
K	K series	1.27	0.01073	5.77	0.22	KBr	Yes	
Total:				100.00				

Electron Image 38

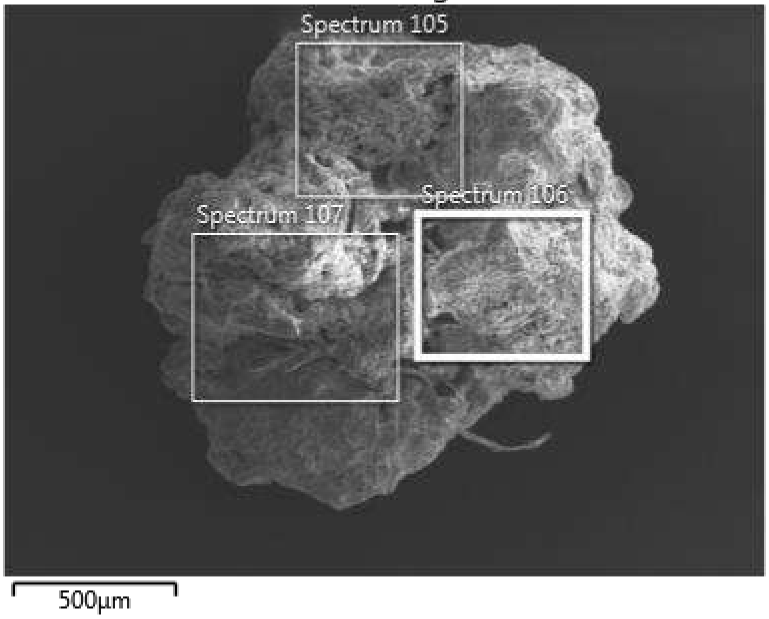

29/11/2019 12:15:36
Project 1
Glandulas salivares Mexico T1
GSM1 15 Kv

Electron Image 38

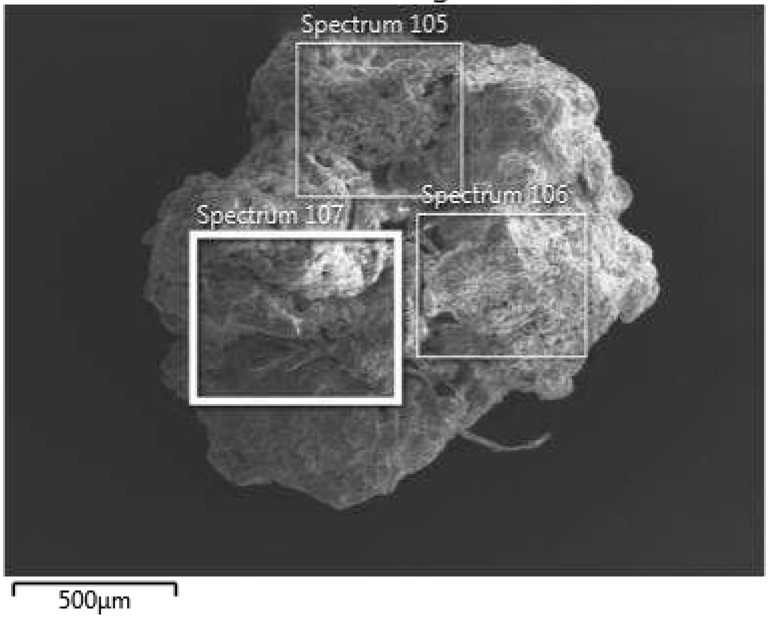

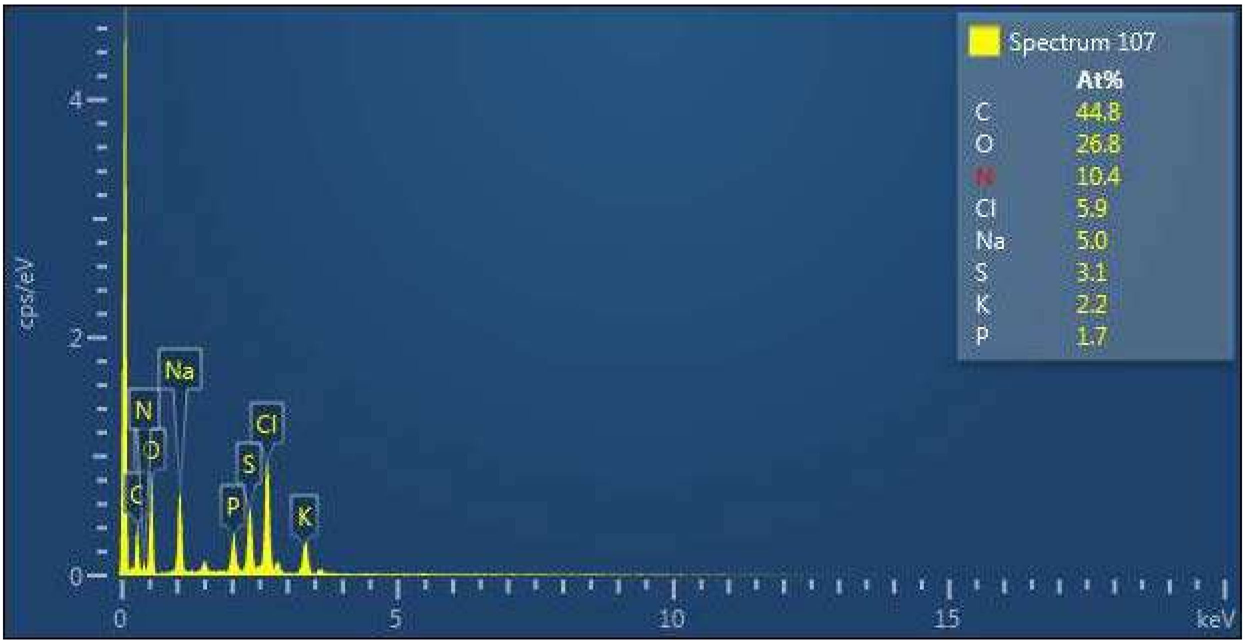

Label:	Spectrum 107
Source:	Acquired
Created:	29/11/2019 12:15:36
Livetime:	60.4s
Process Time:	4
Accelerating Voltage:	15.00kV
Magnification:	48 x
Working Distance:	9.8mm
Specimen Tilt (degrees):	0.0
Elevation (degrees):	35.0
Azimuth (degrees):	0.0
Number Of Channels:	2048
Energy Range (keV):	20 keV
Energy per Channel (eV):	10.0eV
Detector Type Id:	29
Detector Type:	X-Max
Window Type:	SATW
Pulse Pile Up Correction:	Succeeded
Primary Detector:	2617
Primary Detector Serial Number:	77871-X080

Element	Line Type	Apparent Concentration	k Ratio	Wt%	Wt% Sigma	Standard Label	Factory Standard	Standard Calibration Date
C	K series	0.73	0.00726	32.12	1.83	C Vit	Yes	
N	K series	0.86	0.00153	8.65	1.43	BN	Yes	
O	K series	2.27	0.00765	25.60	0.96	SiO2	Yes	
Na	K	1.21	0.00512	6.91	0.29	Albite	Yes	

	series							
P	K series	0.66	0.00370	3.10	0.17	GaP	Yes	
S	K series	0.87	0.00746	5.98	0.26	FeS2	Yes	
Cl	K series	1.68	0.01464	12.47	0.46	NaCl	Yes	
K	K series	0.72	0.00606	5.18	0.25	KBr	Yes	
Total:				100.00				

Electron Image 38

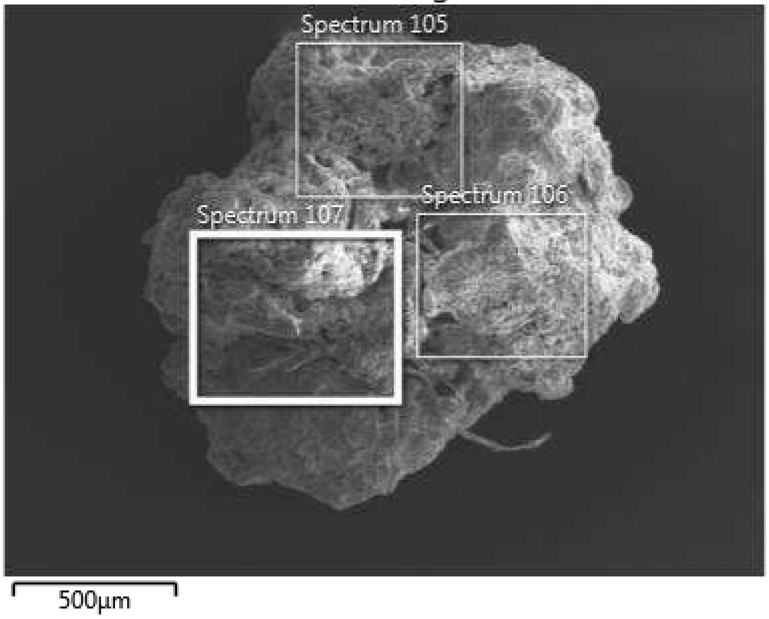

29/11/2019 12:24:12
Project 1
Glandulas salivares Mexico T1
GSM2 15 Kv

Electron Image 39

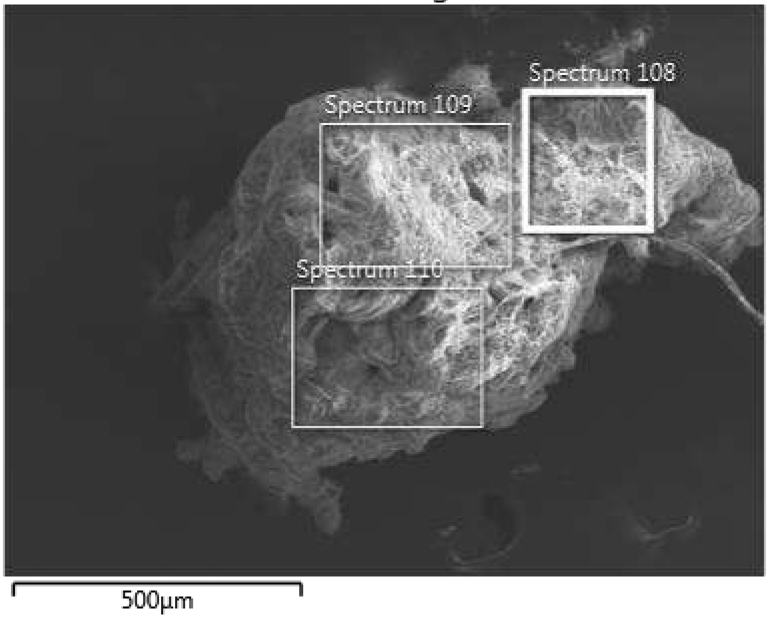

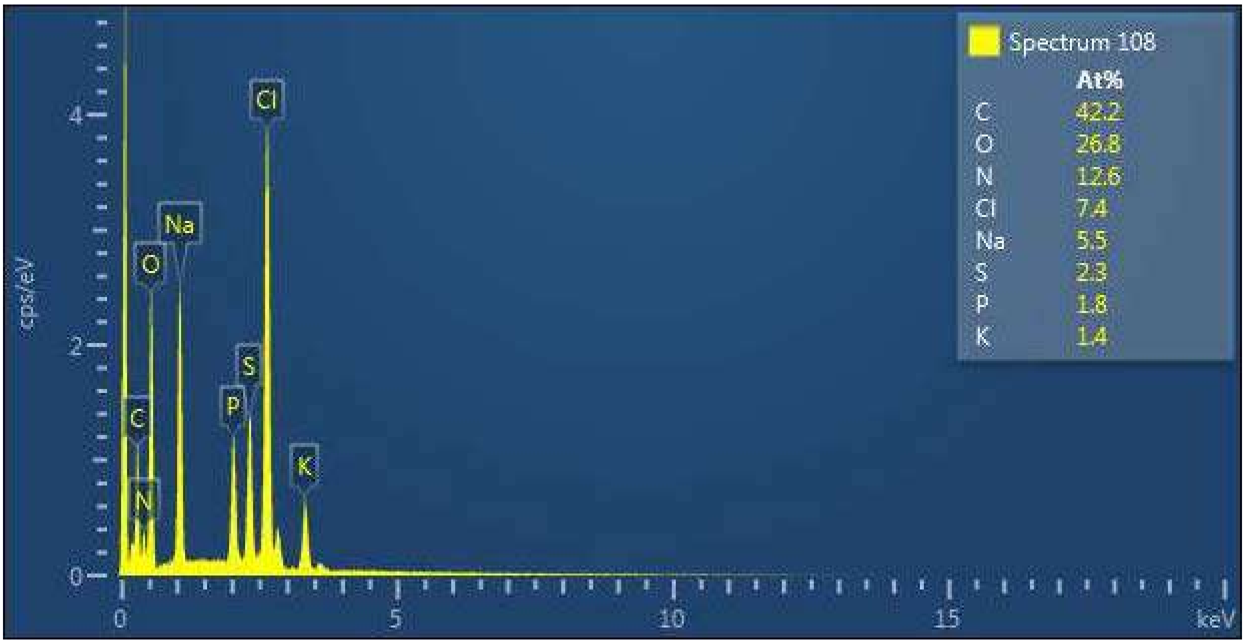

Label:	Spectrum 108
Source:	Acquired
Created:	29/11/2019 12:24:12
Livetime:	60.5s
Process Time:	4
Accelerating Voltage:	15.00kV
Magnification:	85 x
Working Distance:	9.8mm
Specimen Tilt (degrees):	0.0
Elevation (degrees):	35.0
Azimuth (degrees):	0.0
Number Of Channels:	2048
Energy Range (keV):	20 keV
Energy per Channel (eV):	10.0eV
Detector Type Id:	29
Detector Type:	X-Max
Window Type:	SATW
Pulse Pile Up Correction:	Succeeded
Primary Detector:	2617
Primary Detector Serial Number:	77871-X080

Element	Line Type	Apparent Concentration	k Ratio	Wt%	Wt% Sigma	Standard Label	Factory Standard	Standard Calibration Date
C	K series	2.01	0.02014	30.08	1.03	C Vit	Yes	
N	K series	3.47	0.00618	10.43	0.79	BN	Yes	
O	K series	7.28	0.02448	25.40	0.53	SiO2	Yes	
Na	K	4.25	0.01793	7.49	0.17	Albite	Yes	

	series							
P	K series	2.29	0.01278	3.32	0.10	GaP	Yes	
S	K series	2.05	0.01762	4.38	0.12	FeS2	Yes	
Cl	K series	6.79	0.05933	15.58	0.30	NaCl	Yes	
K	K series	1.47	0.01248	3.33	0.11	KBr	Yes	
Total:				100.00				

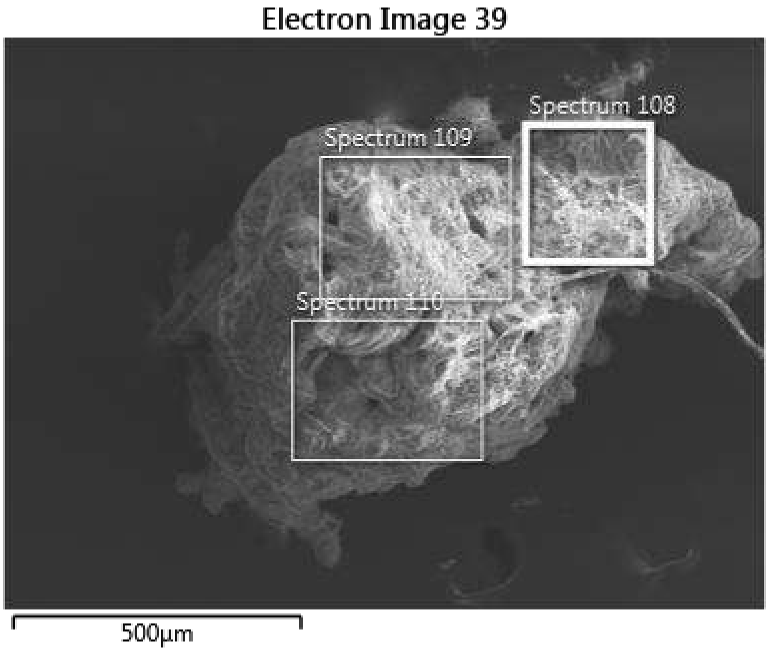

29/11/2019 12:25:22
Project 1
Glandulas salivares Mexico T1
GSM2 15 Kv

Electron Image 39

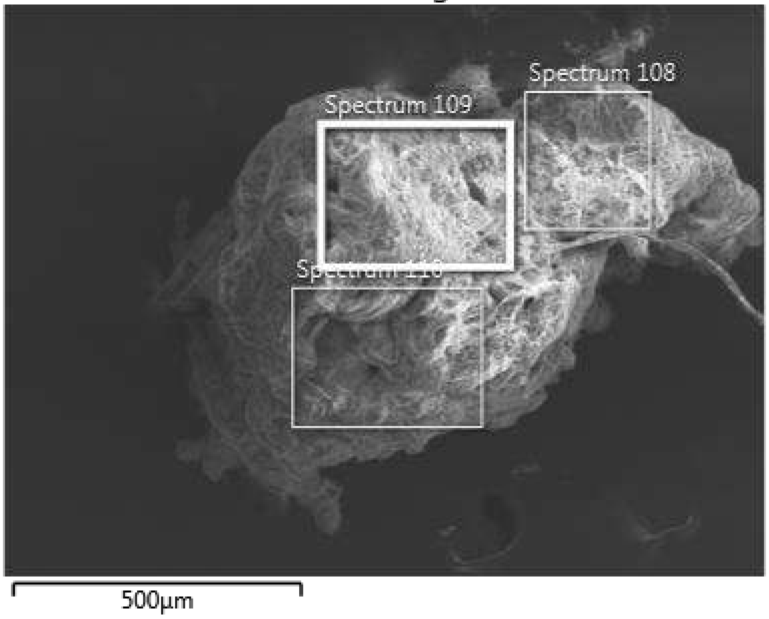

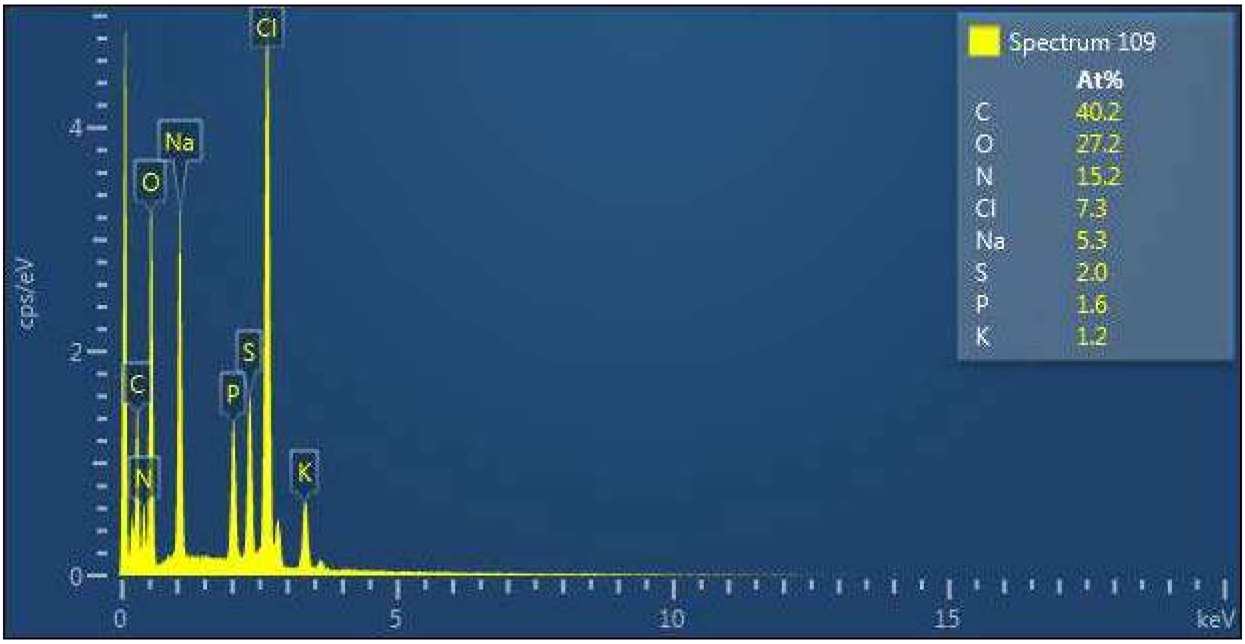

Label:	Spectrum 109
Source:	Acquired
Created:	29/11/2019 12:25:22
Livetime:	60.3s
Process Time:	4
Accelerating Voltage:	15.00kV
Magnification:	85 x
Working Distance:	9.8mm
Specimen Tilt (degrees):	0.0
Elevation (degrees):	35.0
Azimuth (degrees):	0.0
Number Of Channels:	2048
Energy Range (keV):	20 keV
Energy per Channel (eV):	10.0eV
Detector Type Id:	29
Detector Type:	X-Max
Window Type:	SATW
Pulse Pile Up Correction:	Succeeded
Primary Detector:	2617
Primary Detector Serial Number:	77871-X080

Element	Line Type	Apparent Concentration	k Ratio	Wt%	Wt% Sigma	Standard Label	Factory Standard	Standard Calibration Date
C	K series	2.58	0.02585	28.86	0.91	C Vit	Yes	
N	K series	5.80	0.01033	12.72	0.69	BN	Yes	
O	K series	9.66	0.03252	26.07	0.48	SiO2	Yes	
Na	K	5.34	0.02252	7.30	0.15	Albite	Yes	

	series							
P	K series	2.66	0.01487	2.98	0.08	GaP	Yes	
S	K series	2.37	0.02041	3.90	0.10	FeS2	Yes	
Cl	K series	8.78	0.07669	15.45	0.27	NaCl	Yes	
K	K series	1.56	0.01325	2.71	0.09	KBr	Yes	
Total:				100.00				

Electron Image 39

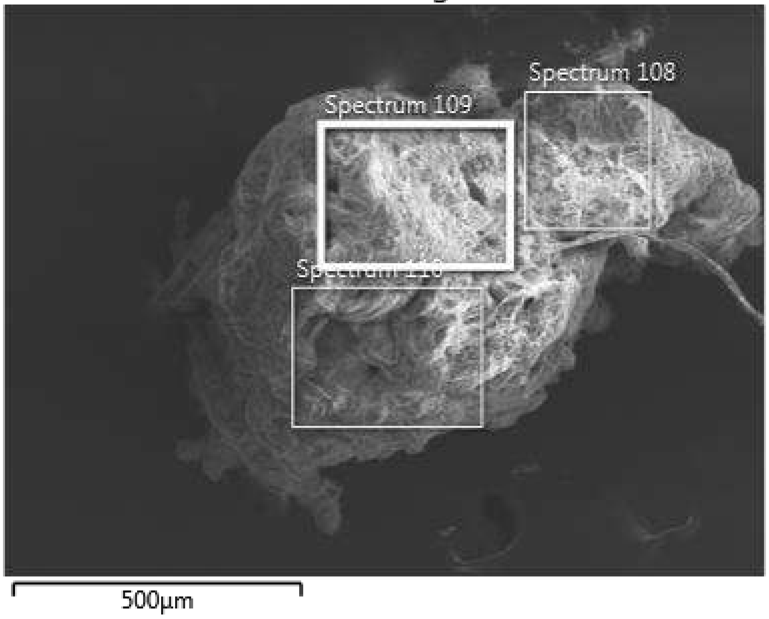

29/11/2019 12:26:31
Project 1
Glandulas salivares Mexico T1
GSM2 15 Kv

Electron Image 39

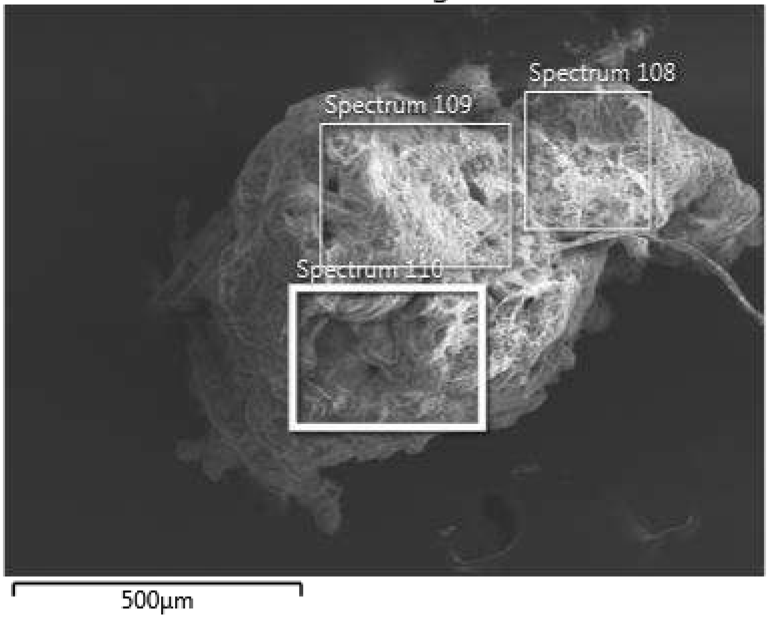

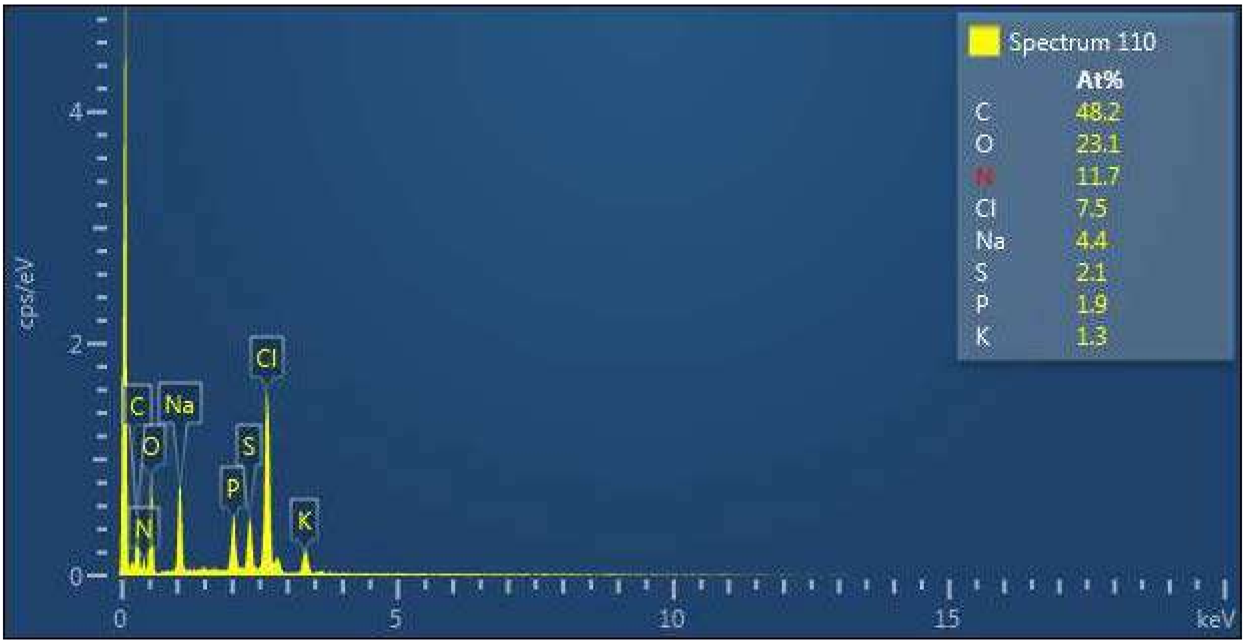

Label:	Spectrum 110
Source:	Acquired
Created:	29/11/2019 12:26:31
Livetime:	60.4s
Process Time:	4
Accelerating Voltage:	15.00kV
Magnification:	85 x
Working Distance:	9.8mm
Specimen Tilt (degrees):	0.0
Elevation (degrees):	35.0
Azimuth (degrees):	0.0
Number Of Channels:	2048
Energy Range (keV):	20 keV
Energy per Channel (eV):	10.0eV
Detector Type Id:	29
Detector Type:	X-Max
Window Type:	SATW
Pulse Pile Up Correction:	Succeeded
Primary Detector:	2617
Primary Detector Serial Number:	77871-X080

Element	Line Type	Apparent Concentration	k Ratio	Wt%	Wt% Sigma	Standard Label	Factory Standard	Standard Calibration Date
C	K series	0.93	0.00927	35.02	1.68	C Vit	Yes	
N	K series	1.20	0.00214	9.89	1.30	BN	Yes	
O	K series	2.39	0.00806	22.36	0.81	SiO2	Yes	
Na	K	1.37	0.00576	6.07	0.24	Albite	Yes	

	series							
P	K series	0.99	0.00554	3.64	0.17	GaP	Yes	
S	K series	0.73	0.00631	3.99	0.18	FeS2	Yes	
Cl	K series	2.76	0.02408	16.06	0.52	NaCl	Yes	
K	K series	0.52	0.00438	2.98	0.17	KBr	Yes	
Total:				100.00				

Electron Image 39

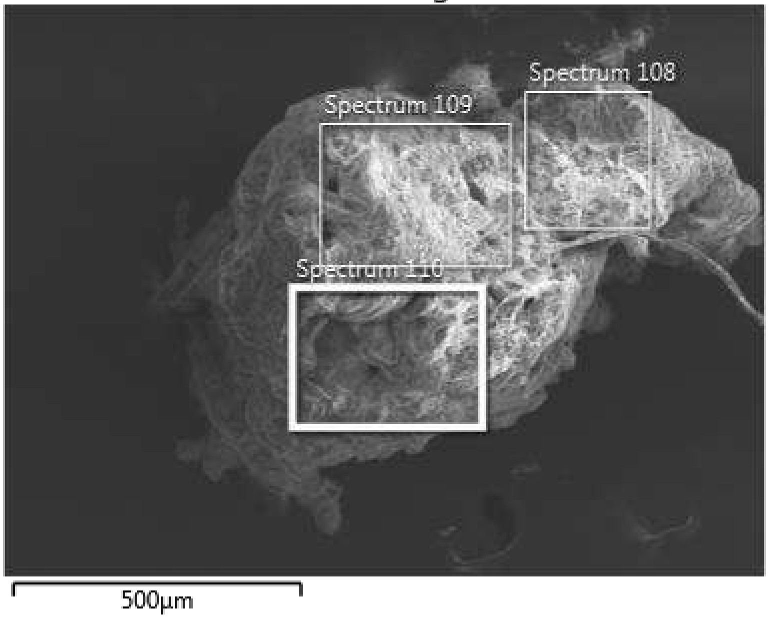

29/11/2019 12:29:30
Project 1
Glandulas salivares Mexico T2
GSM1 15 Kv

Electron Image 40

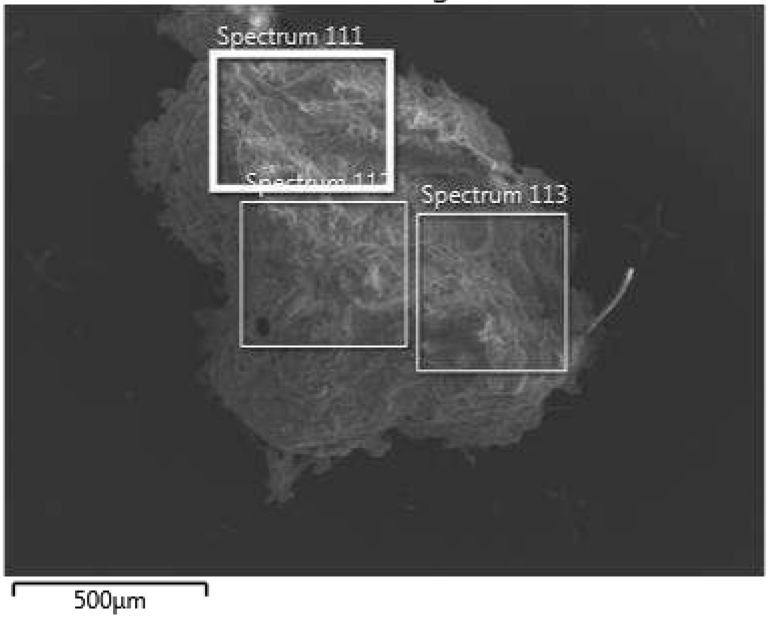

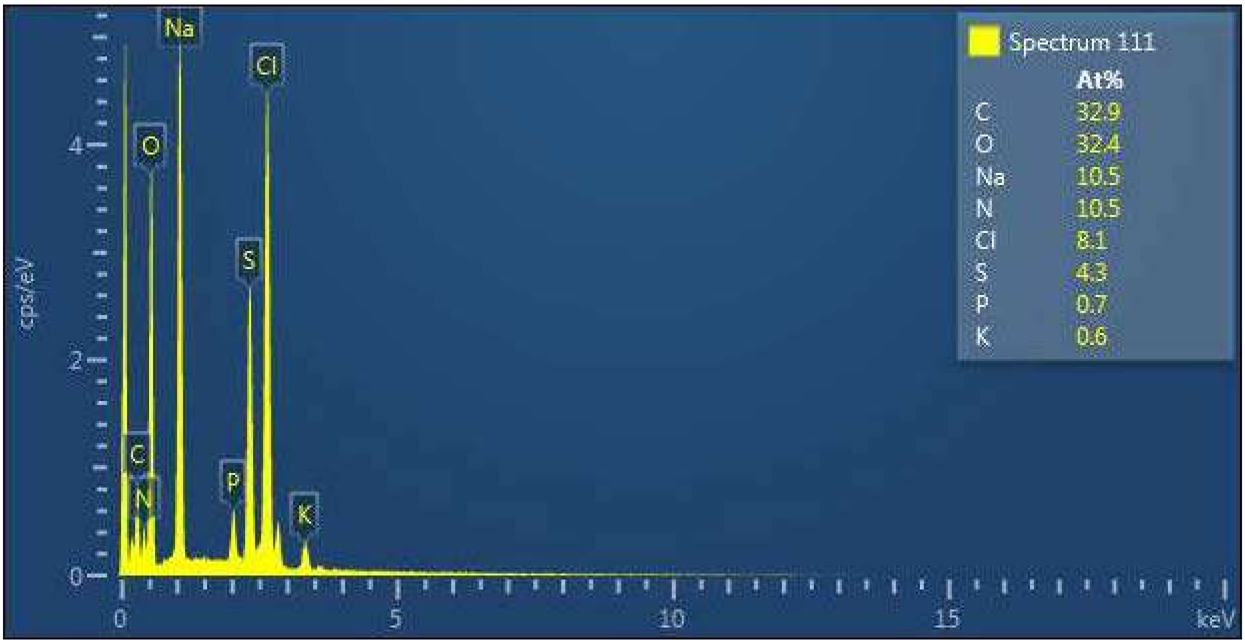

Label:	Spectrum 111
Source:	Acquired
Created:	29/11/2019 12:29:30
Livetime:	60.2s
Process Time:	4
Accelerating Voltage:	15.00kV
Magnification:	57 x
Working Distance:	9.8mm
Specimen Tilt (degrees):	0.0
Elevation (degrees):	35.0
Azimuth (degrees):	0.0
Number Of Channels:	2048
Energy Range (keV):	20 keV
Energy per Channel (eV):	10.0eV
Detector Type Id:	29
Detector Type:	X-Max
Window Type:	SATW
Pulse Pile Up Correction:	Succeeded
Primary Detector:	2617
Primary Detector Serial Number:	77871-X080

Element	Line Type	Apparent Concentration	k Ratio	Wt%	Wt% Sigma	Standard Label	Factory Standard	Standard Calibration Date
C	K series	1.47	0.01474	22.30	1.07	C Vit	Yes	
N	K series	3.58	0.00638	8.28	0.66	BN	Yes	
O	K series	11.05	0.03717	29.26	0.54	SiO2	Yes	
Na	K	8.93	0.03767	13.61	0.25	Albite	Yes	

	series							
P	K series	0.96	0.00535	1.25	0.06	GaP	Yes	
S	K series	4.11	0.03537	7.74	0.16	FeS2	Yes	
Cl	K series	7.92	0.06919	16.24	0.29	NaCl	Yes	
K	K series	0.66	0.00561	1.33	0.07	KBr	Yes	
Total:				100.00				

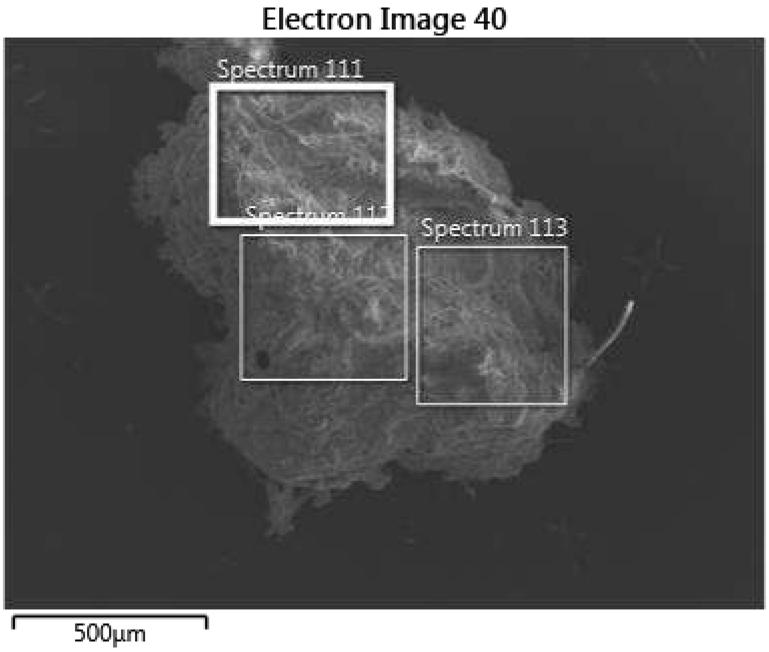

29/11/2019 12:30:38
Project 1
Glandulas salivares Mexico T2
GSM1 15 Kv

Electron Image 40

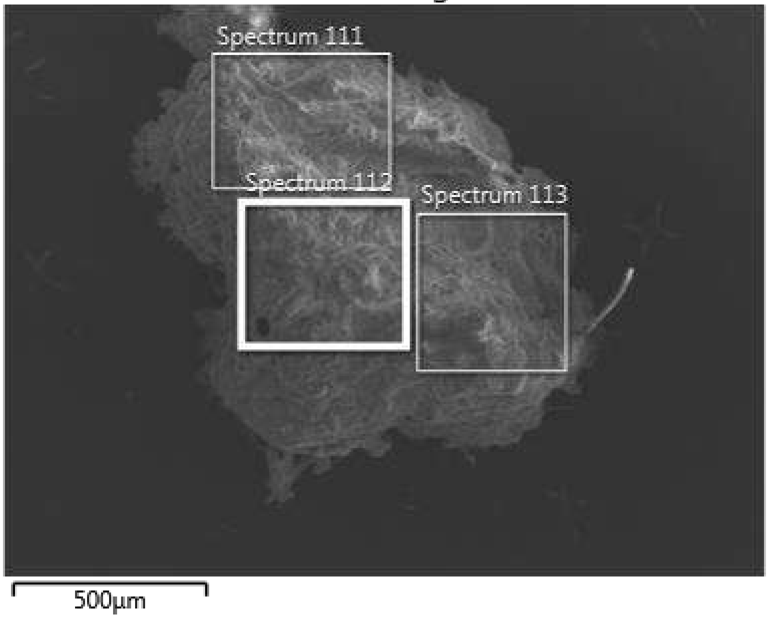

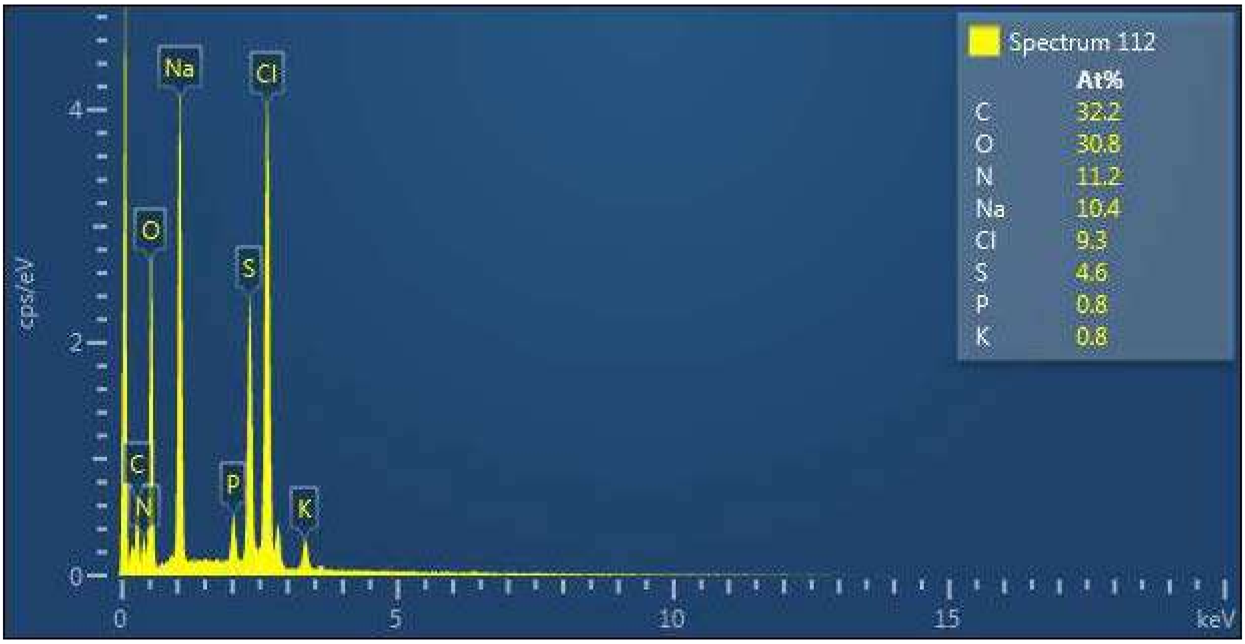

Label:	Spectrum 112
Source:	Acquired
Created:	29/11/2019 12:30:38
Livetime:	60.2s
Process Time:	4
Accelerating Voltage:	15.00kV
Magnification:	57 x
Working Distance:	9.8mm
Specimen Tilt (degrees):	0.0
Elevation (degrees):	35.0
Azimuth (degrees):	0.0
Number Of Channels:	2048
Energy Range (keV):	20 keV
Energy per Channel (eV):	10.0eV
Detector Type Id:	29
Detector Type:	X-Max
Window Type:	SATW
Pulse Pile Up Correction:	Succeeded
Primary Detector:	2617
Primary Detector Serial Number:	77871-X080

Element	Line Type	Apparent Concentration	k Ratio	Wt%	Wt% Sigma	Standard Label	Factory Standard	Standard Calibration Date
C	K series	1.07	0.01065	21.43	1.22	C Vit	Yes	
N	K series	2.93	0.00523	8.70	0.74	BN	Yes	
O	K series	8.01	0.02695	27.27	0.57	SiO2	Yes	
Na	K	7.09	0.02993	13.28	0.28	Albite	Yes	

	series							
P	K series	0.84	0.00469	1.34	0.07	GaP	Yes	
S	K series	3.51	0.03020	8.13	0.19	FeS2	Yes	
Cl	K series	7.18	0.06271	18.20	0.36	NaCl	Yes	
K	K series	0.66	0.00560	1.64	0.08	KBr	Yes	
Total:				100.00				

Electron Image 40

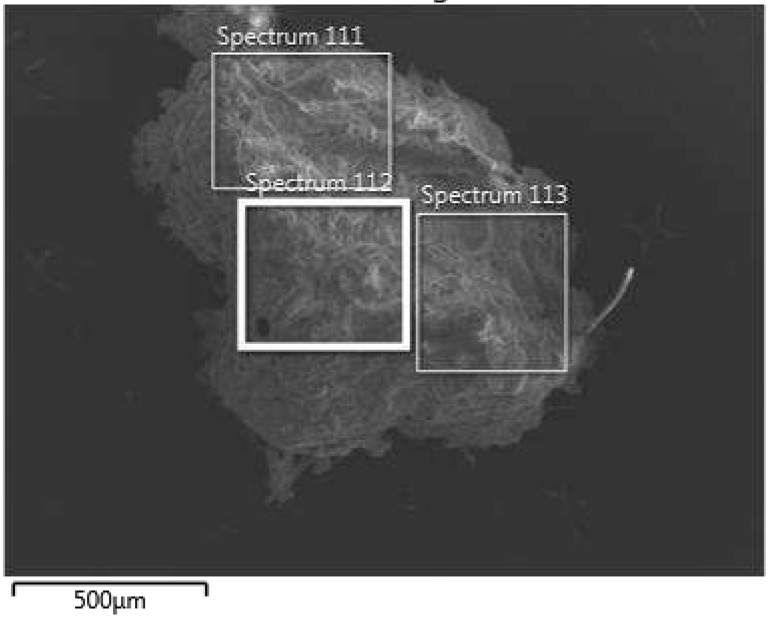

29/11/2019 12:31:46
Project 1
Glandulas salivares Mexico T2
GSM1 15 Kv

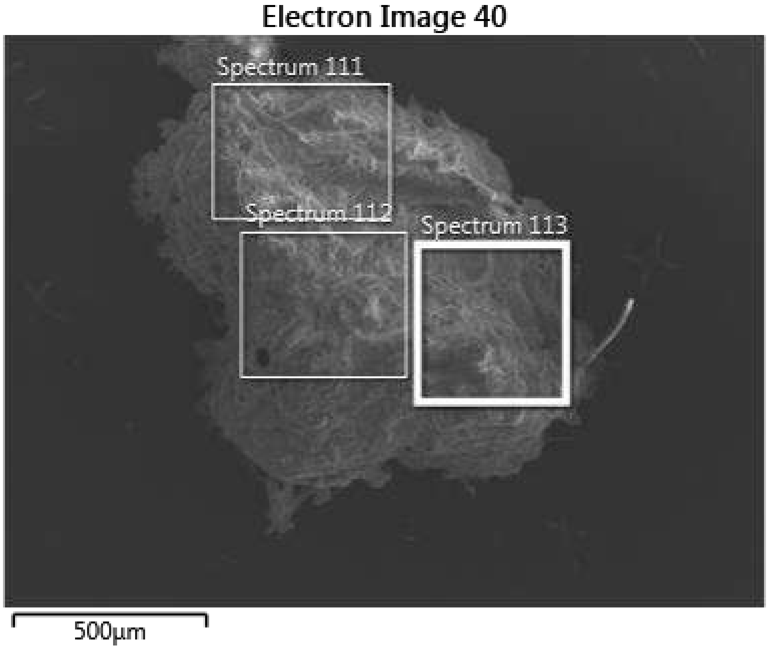

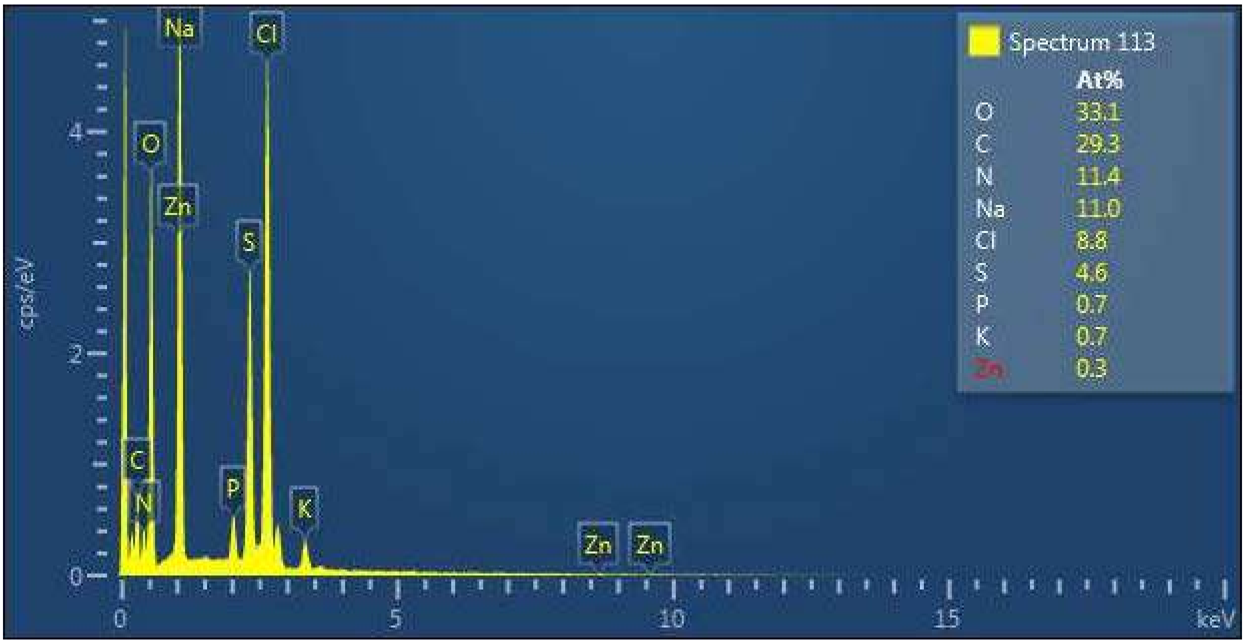

Label:	Spectrum 113
Source:	Acquired
Created:	29/11/2019 12:31:46
Livetime:	60.3s
Process Time:	4
Accelerating Voltage:	15.00kV
Magnification:	57 x
Working Distance:	9.8mm
Specimen Tilt (degrees):	0.0
Elevation (degrees):	35.0
Azimuth (degrees):	0.0
Number Of Channels:	2048
Energy Range (keV):	20 keV
Energy per Channel (eV):	10.0eV
Detector Type Id:	29
Detector Type:	X-Max
Window Type:	SATW
Pulse Pile Up Correction:	Succeeded
Primary Detector:	2617
Primary Detector Serial Number:	77871-X080

Element	Line Type	Apparent Concentration	k Ratio	Wt%	Wt% Sigma	Standard Label	Factory Standard	Standard Calibration Date
C	K series	1.16	0.01160	19.26	1.12	C Vit	Yes	
N	K series	3.75	0.00668	8.79	0.65	BN	Yes	
O	K series	10.62	0.03575	29.06	0.55	SiO2	Yes	
Na	K	8.61	0.03635	13.89	0.29	Albite	Yes	

	series							
P	K series	0.88	0.00495	1.21	0.06	GaP	Yes	
S	K series	4.09	0.03525	8.06	0.17	FeS2	Yes	
Cl	K series	8.00	0.06994	17.16	0.32	NaCl	Yes	
K	K series	0.67	0.00568	1.40	0.07	KBr	Yes	
Zn	L series	0.32	0.00317	1.18	0.37	Zn	Yes	
Total:				100.00				

Electron Image 40

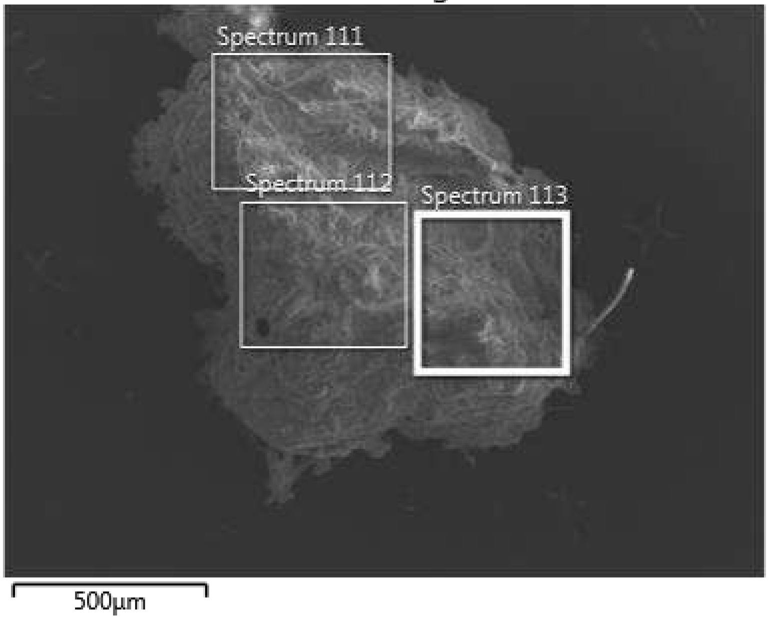

29/11/2019 12:33:38
Project 1
Glandulas salivares Mexico T2
GSM2 15 Kv

Electron Image 41

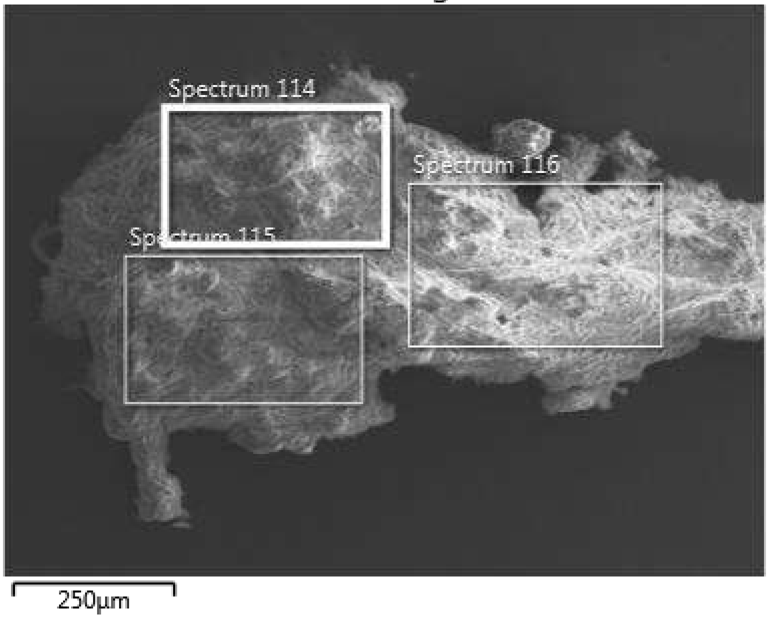

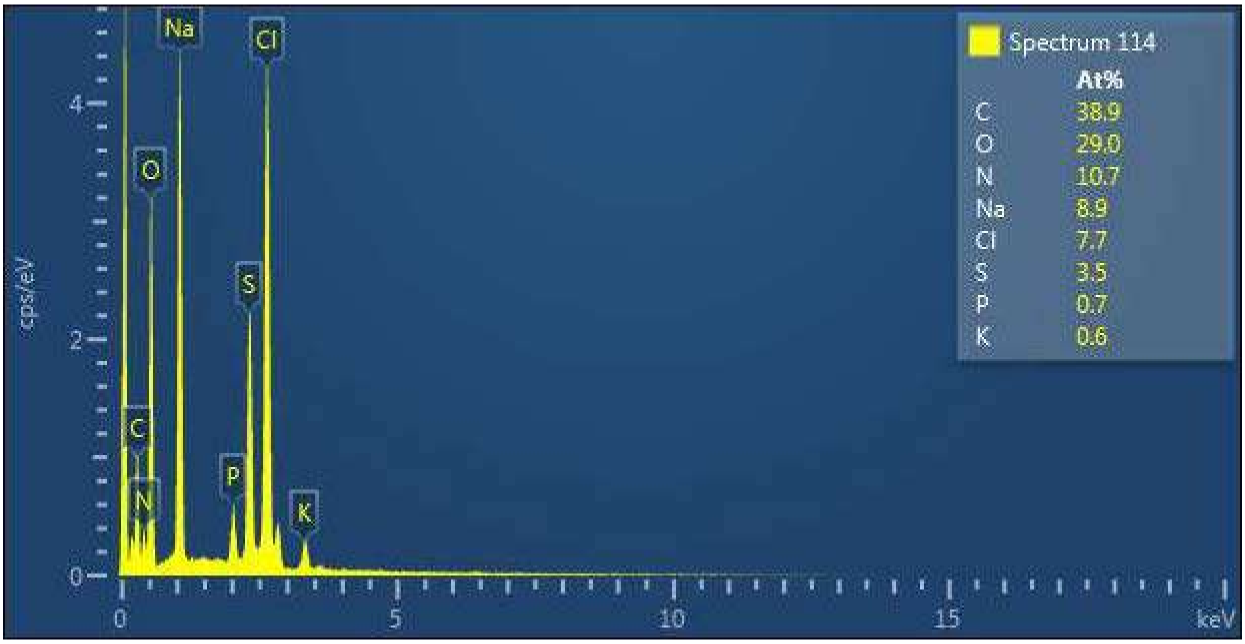

Label:	Spectrum 114
Source:	Acquired
Created:	29/11/2019 12:33:38
Livetime:	60.1s
Process Time:	4
Accelerating Voltage:	15.00kV
Magnification:	95 x
Working Distance:	9.8mm
Specimen Tilt (degrees):	0.0
Elevation (degrees):	35.0
Azimuth (degrees):	0.0
Number Of Channels:	2048
Energy Range (keV):	20 keV
Energy per Channel (eV):	10.0eV
Detector Type Id:	29
Detector Type:	X-Max
Window Type:	SATW
Pulse Pile Up Correction:	Succeeded
Primary Detector:	2617
Primary Detector Serial Number:	77871-X080

Element	Line Type	Apparent Concentration	k Ratio	Wt%	Wt% Sigma	Standard Label	Factory Standard	Standard Calibration Date
C	K series	1.84	0.01838	27.22	1.04	C Vit	Yes	
N	K series	3.41	0.00606	8.73	0.70	BN	Yes	
O	K series	9.26	0.03116	27.08	0.52	SiO2	Yes	
Na	K	7.48	0.03157	11.90	0.23	Albite	Yes	

	series							
P	K series	0.97	0.00544	1.32	0.06	GaP	Yes	
S	K series	3.34	0.02881	6.56	0.15	FeS2	Yes	
Cl	K series	7.48	0.06533	15.91	0.30	NaCl	Yes	
K	K series	0.61	0.00518	1.27	0.07	KBr	Yes	
Total:				100.00				

Electron Image 41

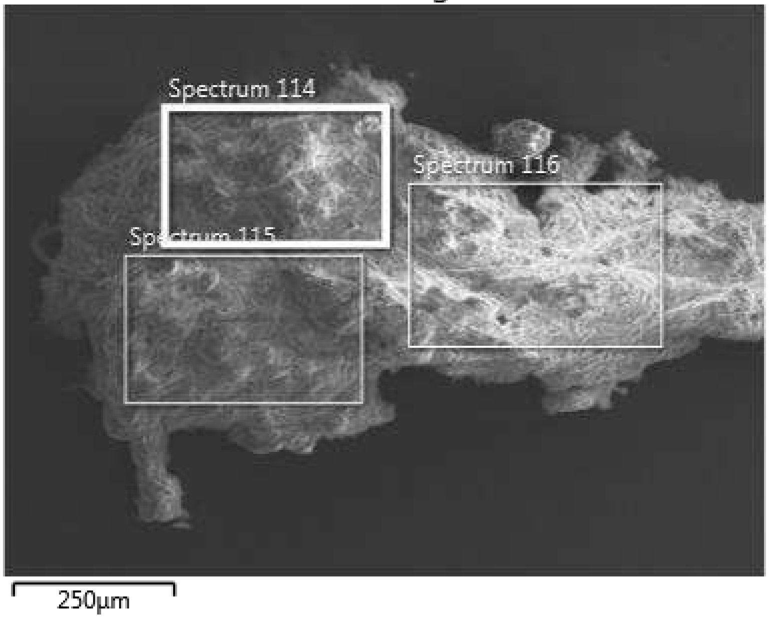

29/11/2019 12:34:45
Project 1
Glandulas salivares Mexico T2
GSM2 15 Kv

Electron Image 41

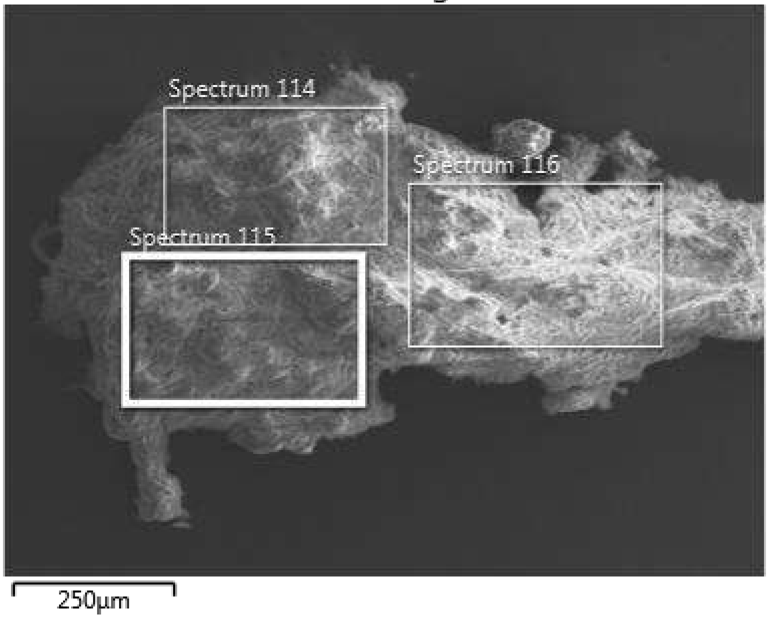

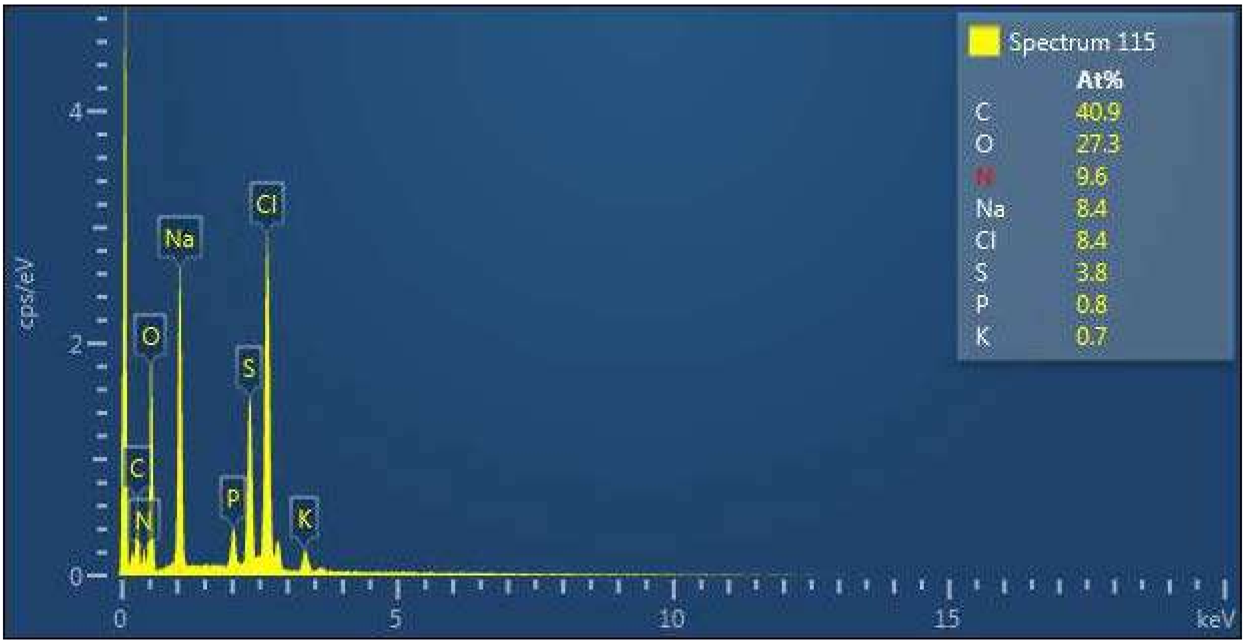

Label:	Spectrum 115
Source:	Acquired
Created:	29/11/2019 12:34:45
Livetime:	60.1s
Process Time:	4
Accelerating Voltage:	15.00kV
Magnification:	95 x
Working Distance:	9.8mm
Specimen Tilt (degrees):	0.0
Elevation (degrees):	35.0
Azimuth (degrees):	0.0
Number Of Channels:	2048
Energy Range (keV):	20 keV
Energy per Channel (eV):	10.0eV
Detector Type Id:	29
Detector Type:	X-Max
Window Type:	SATW
Pulse Pile Up Correction:	Succeeded
Primary Detector:	2617
Primary Detector Serial Number:	77871-X080

Element	Line Type	Apparent Concentration	k Ratio	Wt%	Wt% Sigma	Standard Label	Factory Standard	Standard Calibration Date
C	K series	1.15	0.01153	28.41	1.34	C Vit	Yes	
N	K series	1.81	0.00322	7.76	0.90	BN	Yes	
O	K series	5.33	0.01793	25.25	0.64	SiO2	Yes	
Na	K	4.51	0.01905	11.20	0.28	Albite	Yes	

	series							
P	K series	0.71	0.00398	1.50	0.08	GaP	Yes	
S	K series	2.32	0.01995	7.12	0.20	FeS2	Yes	
Cl	K series	5.13	0.04481	17.18	0.41	NaCl	Yes	
K	K series	0.48	0.00404	1.57	0.10	KBr	Yes	
Total:				100.00				

Electron Image 41

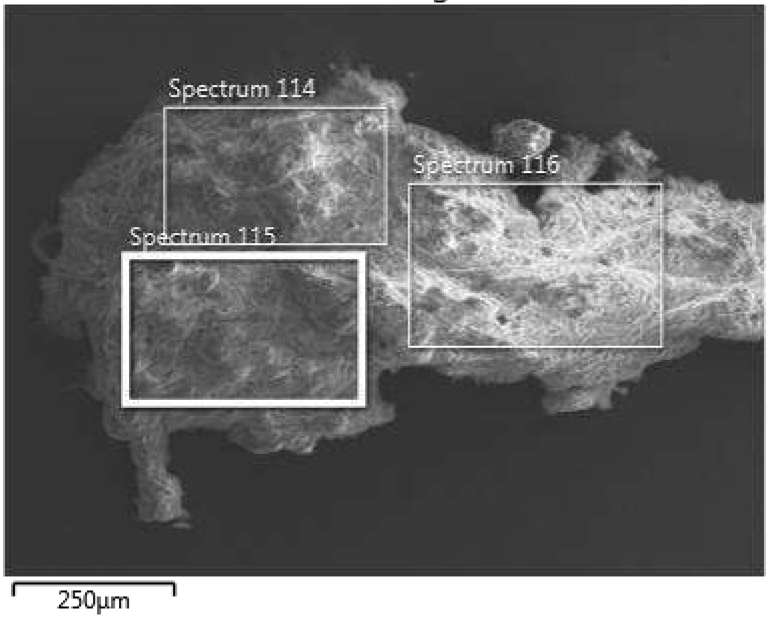

29/11/2019 12:35:52
Project 1
Glandulas salivares Mexico T2
GSM2 15 Kv

Electron Image 41

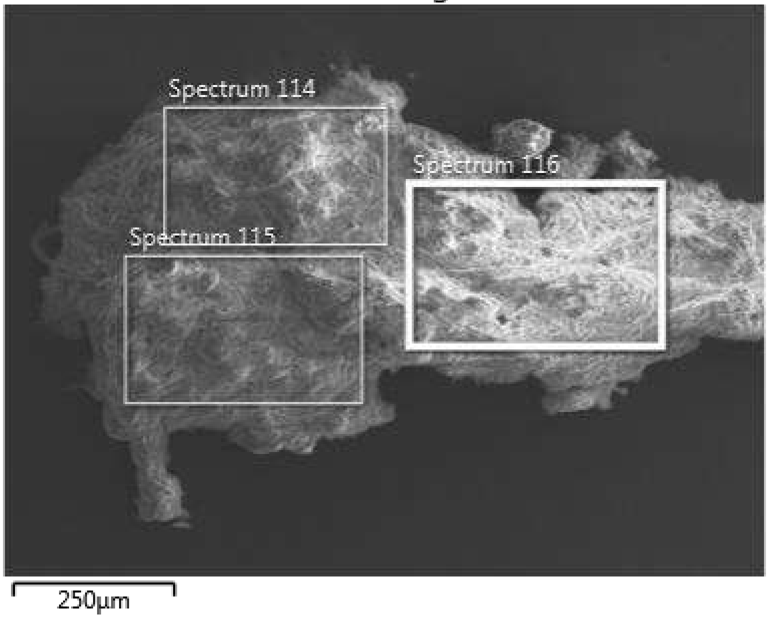

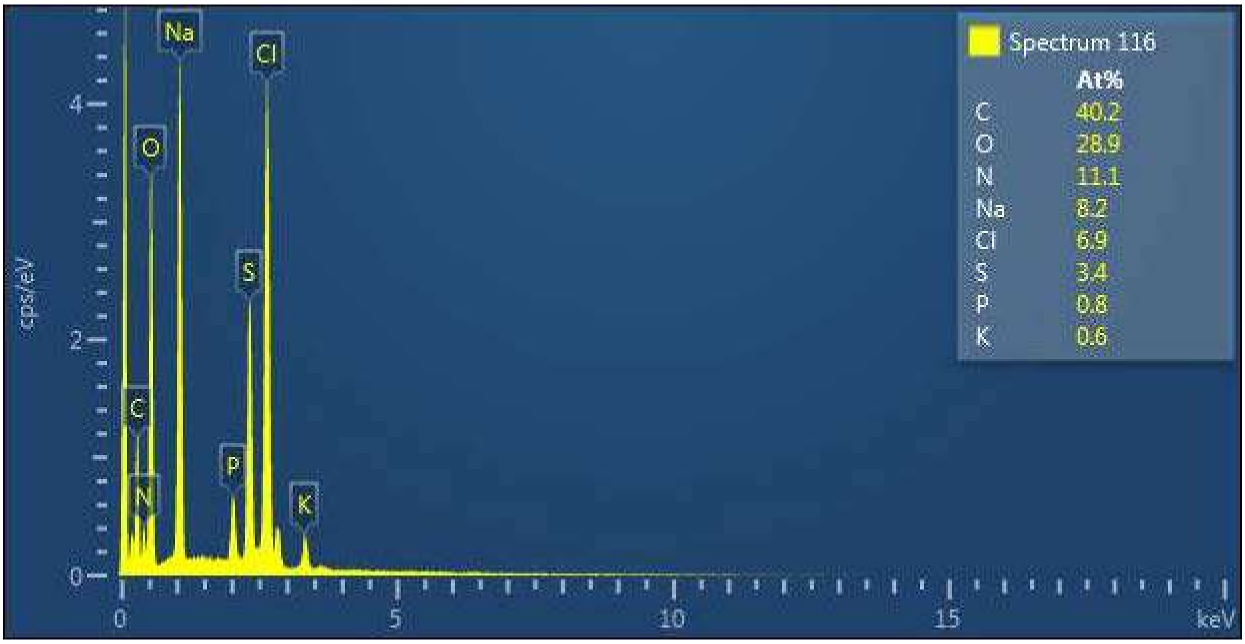

Label:	Spectrum 116
Source:	Acquired
Created:	29/11/2019 12:35:52
Livetime:	60.2s
Process Time:	4
Accelerating Voltage:	15.00kV
Magnification:	95 x
Working Distance:	9.8mm
Specimen Tilt (degrees):	0.0
Elevation (degrees):	35.0
Azimuth (degrees):	0.0
Number Of Channels:	2048
Energy Range (keV):	20 keV
Energy per Channel (eV):	10.0eV
Detector Type Id:	29
Detector Type:	X-Max
Window Type:	SATW
Pulse Pile Up Correction:	Succeeded
Primary Detector:	2617
Primary Detector Serial Number:	77871-X080

Element	Line Type	Apparent Concentration	k Ratio	Wt%	Wt% Sigma	Standard Label	Factory Standard	Standard Calibration Date
C	K series	2.19	0.02190	28.55	0.95	C Vit	Yes	
N	K series	3.84	0.00684	9.17	0.67	BN	Yes	
O	K series	9.96	0.03352	27.32	0.50	SiO2	Yes	
Na	K	7.47	0.03151	11.12	0.21	Albite	Yes	

	series							
P	K series	1.18	0.00662	1.49	0.06	GaP	Yes	
S	K series	3.54	0.03046	6.48	0.14	FeS2	Yes	
Cl	K series	7.32	0.06394	14.52	0.26	NaCl	Yes	
K	K series	0.69	0.00587	1.34	0.07	KBr	Yes	
Total:				100.00				

Electron Image 41

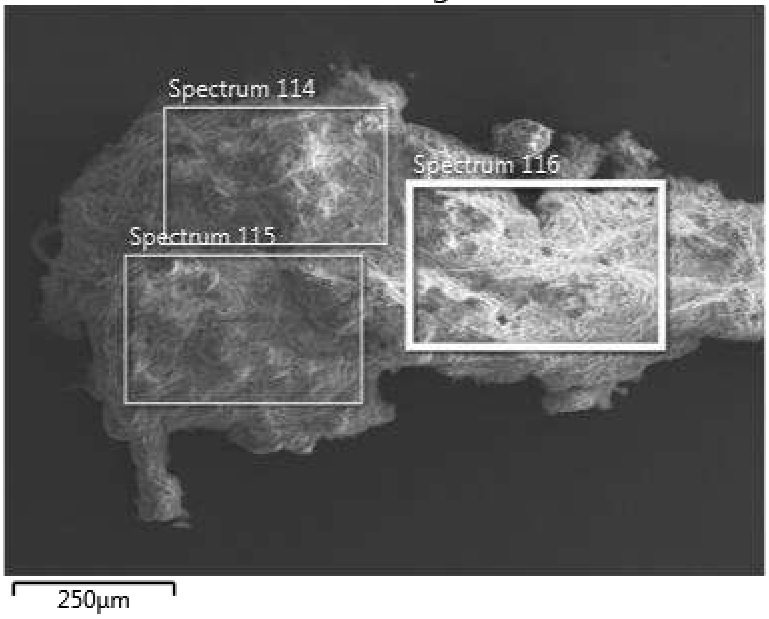

29/11/2019 12:38:09
Project 1
Glandulas salivares Mexico T3
GSM3 15 Kv

Electron Image 42

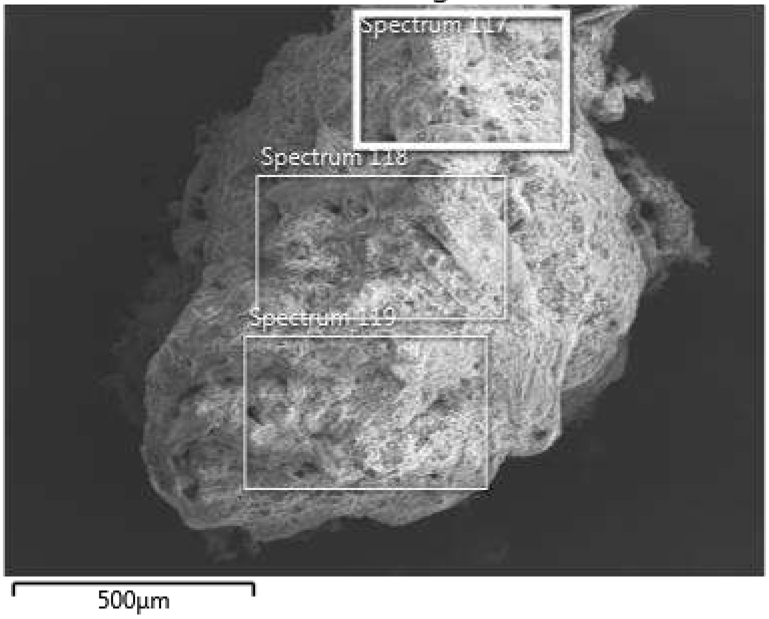

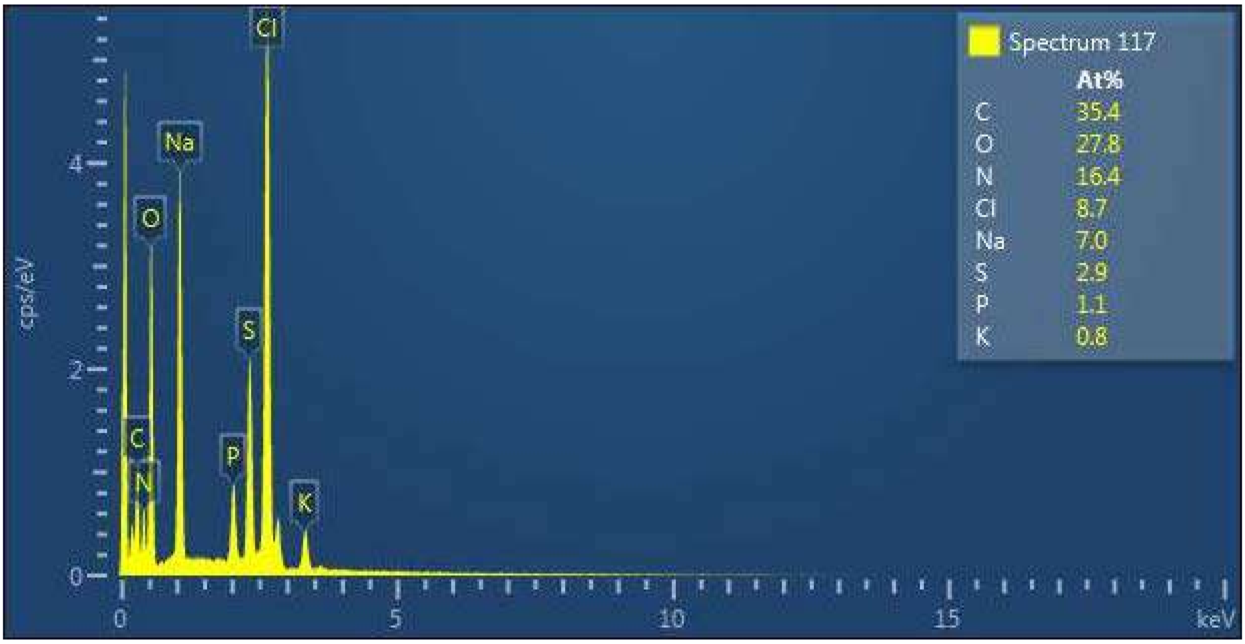

Label:	Spectrum 117
Source:	Acquired
Created:	29/11/2019 12:38:09
Livetime:	60.0s
Process Time:	4
Accelerating Voltage:	15.00kV
Magnification:	71 x
Working Distance:	9.8mm
Specimen Tilt (degrees):	0.0
Elevation (degrees):	35.0
Azimuth (degrees):	0.0
Number Of Channels:	2048
Energy Range (keV):	20 keV
Energy per Channel (eV):	10.0eV
Detector Type Id:	29
Detector Type:	X-Max
Window Type:	SATW
Pulse Pile Up Correction:	Succeeded
Primary Detector:	2617
Primary Detector Serial Number:	77871-X080

Element	Line Type	Apparent Concentration	k Ratio	Wt%	Wt% Sigma	Standard Label	Factory Standard	Standard Calibration Date
C	K series	1.85	0.01846	24.69	1.02	C Vit	Yes	
N	K series	6.02	0.01072	13.31	0.69	BN	Yes	
O	K series	9.25	0.03112	25.76	0.49	SiO2	Yes	
Na	K	6.61	0.02788	9.40	0.19	Albite	Yes	

	series							
P	K series	1.62	0.00905	1.91	0.07	GaP	Yes	
S	K series	3.12	0.02688	5.37	0.12	FeS2	Yes	
Cl	K series	9.57	0.08366	17.80	0.31	NaCl	Yes	
K	K series	0.95	0.00809	1.76	0.07	KBr	Yes	
Total:				100.00				

Electron Image 42

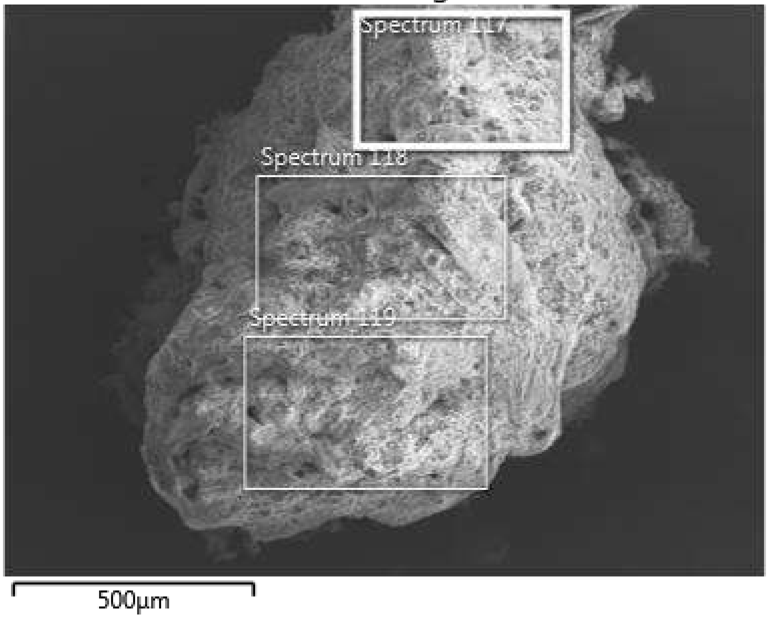

29/11/2019 12:39:17
Project 1
Glandulas salivares Mexico T3
GSM3 15 Kv

Electron Image 42

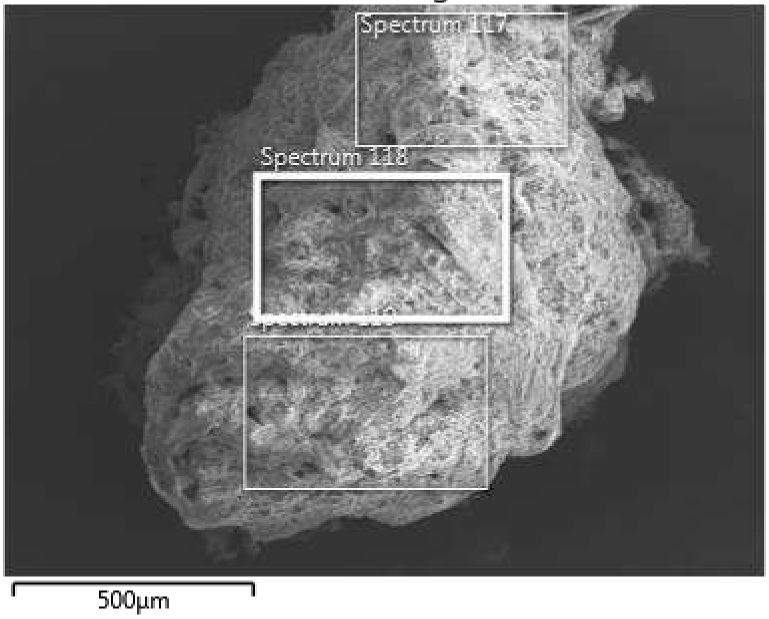

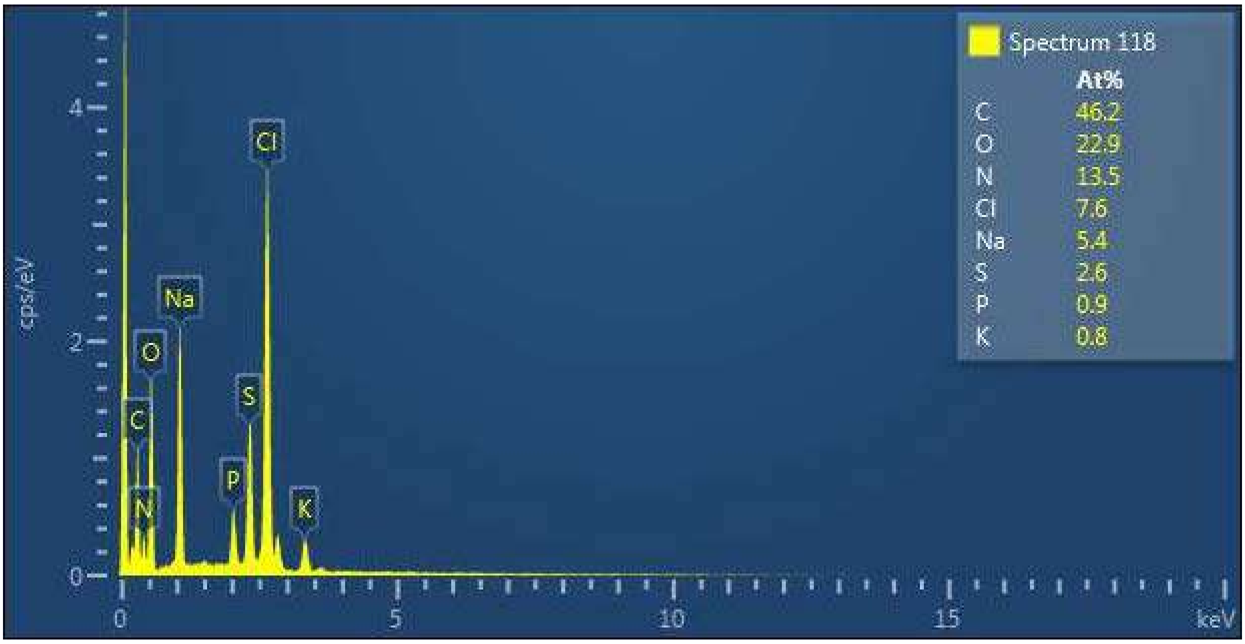

Label:	Spectrum 118
Source:	Acquired
Created:	29/11/2019 12:39:17
Livetime:	60.0s
Process Time:	4
Accelerating Voltage:	15.00kV
Magnification:	71 x
Working Distance:	9.8mm
Specimen Tilt (degrees):	0.0
Elevation (degrees):	35.0
Azimuth (degrees):	0.0
Number Of Channels:	2048
Energy Range (keV):	20 keV
Energy per Channel (eV):	10.0eV
Detector Type Id:	29
Detector Type:	X-Max
Window Type:	SATW
Pulse Pile Up Correction:	Succeeded
Primary Detector:	2617
Primary Detector Serial Number:	77871-X080

Element	Line Type	Apparent Concentration	k Ratio	Wt%	Wt% Sigma	Standard Label	Factory Standard	Standard Calibration Date
C	K series	1.86	0.01859	33.62	1.16	C Vit	Yes	
N	K series	3.08	0.00548	11.48	0.90	BN	Yes	
O	K series	5.07	0.01705	22.23	0.56	SiO2	Yes	
Na	K	3.60	0.01518	7.52	0.19	Albite	Yes	

	series							
P	K series	1.01	0.00564	1.76	0.08	GaP	Yes	
S	K series	1.97	0.01698	5.02	0.15	FeS2	Yes	
Cl	K series	5.97	0.05218	16.43	0.36	NaCl	Yes	
K	K series	0.71	0.00605	1.94	0.09	KBr	Yes	
Total:				100.00				

Electron Image 42

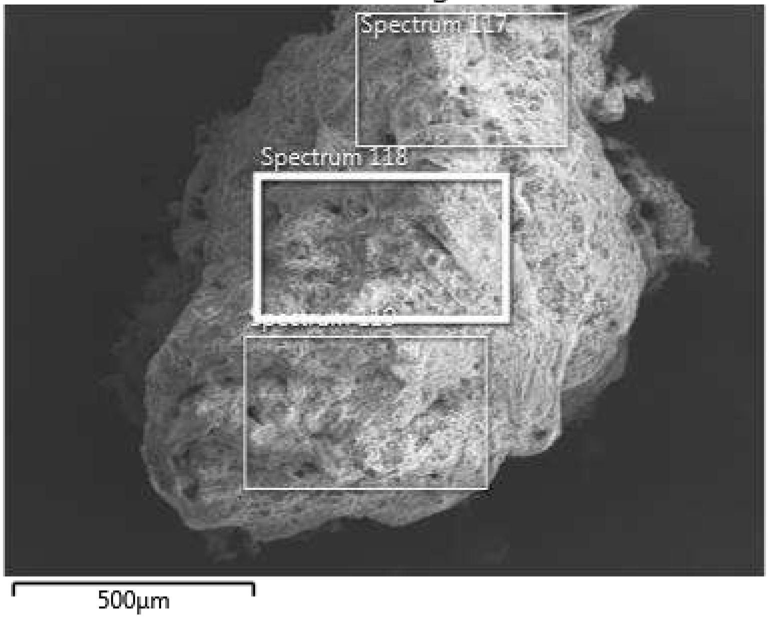

29/11/2019 12:40:23
Project 1
Glandulas salivares Mexico T3
GSM3 15 Kv

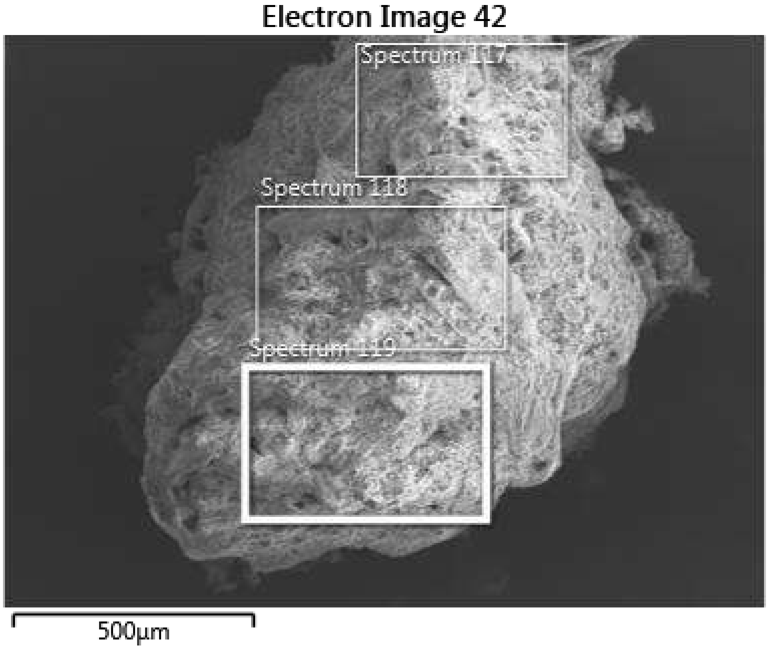

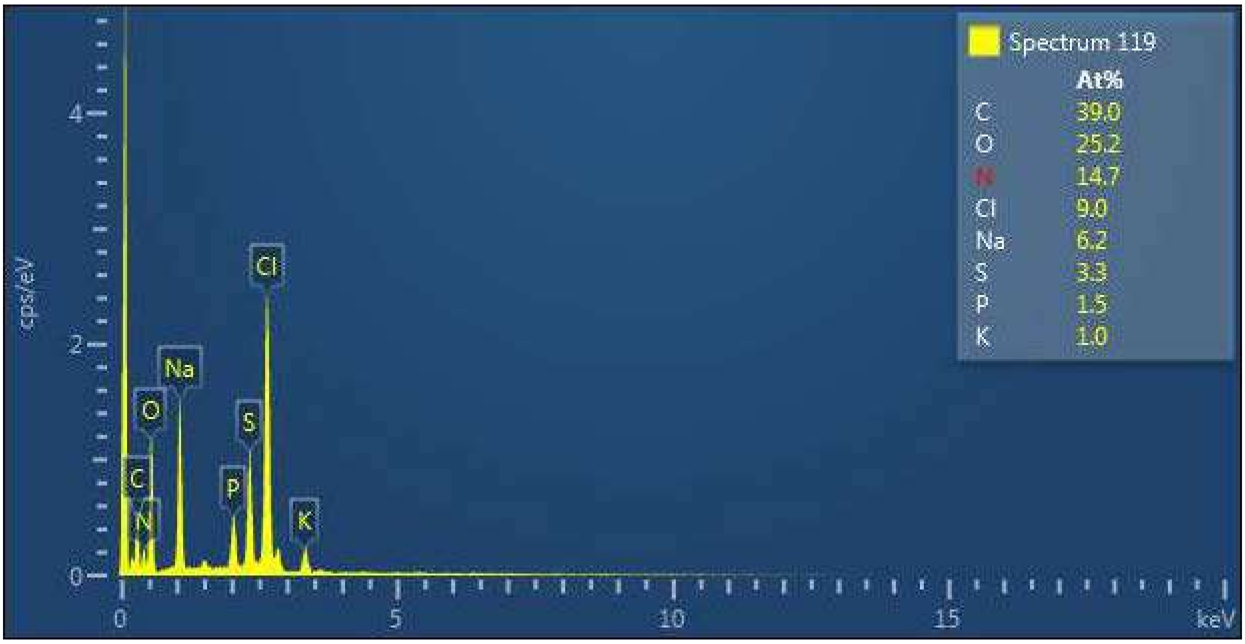

Label:	Spectrum 119
Source:	Acquired
Created:	29/11/2019 12:40:23
Livetime:	60.4s
Process Time:	4
Accelerating Voltage:	15.00kV
Magnification:	71 x
Working Distance:	9.8mm
Specimen Tilt (degrees):	0.0
Elevation (degrees):	35.0
Azimuth (degrees):	0.0
Number Of Channels:	2048
Energy Range (keV):	20 keV
Energy per Channel (eV):	10.0eV
Detector Type Id:	29
Detector Type:	X-Max
Window Type:	SATW
Pulse Pile Up Correction:	Succeeded
Primary Detector:	2617
Primary Detector Serial Number:	77871-X080

Element	Line Type	Apparent Concentration	k Ratio	Wt%	Wt% Sigma	Standard Label	Factory Standard	Standard Calibration Date
C	K series	0.82	0.00825	27.05	1.62	C Vit	Yes	
N	K series	2.11	0.00375	11.89	1.09	BN	Yes	
O	K series	3.44	0.01158	23.27	0.73	SiO2	Yes	
Na	K	2.51	0.01060	8.28	0.26	Albite	Yes	

	series							
P	K series	0.98	0.00547	2.68	0.13	GaP	Yes	
S	K series	1.53	0.01317	6.16	0.21	FeS2	Yes	
Cl	K series	4.21	0.03675	18.37	0.51	NaCl	Yes	
K	K series	0.53	0.00450	2.29	0.13	KBr	Yes	
Total:				100.00				

Electron Image 42

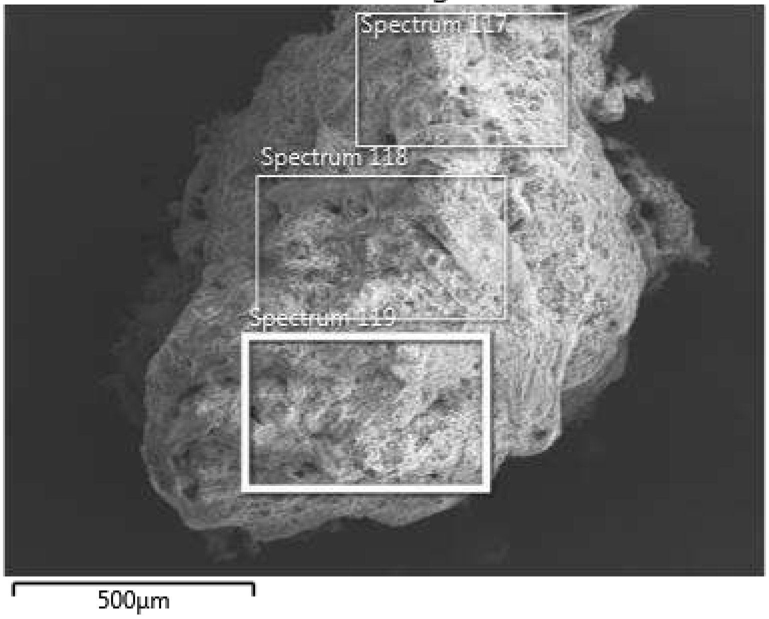

29/11/2019 12:42:16
Project 1
Glandulas salivares Mexico T3
GSM2 15 Kv

Electron Image 43

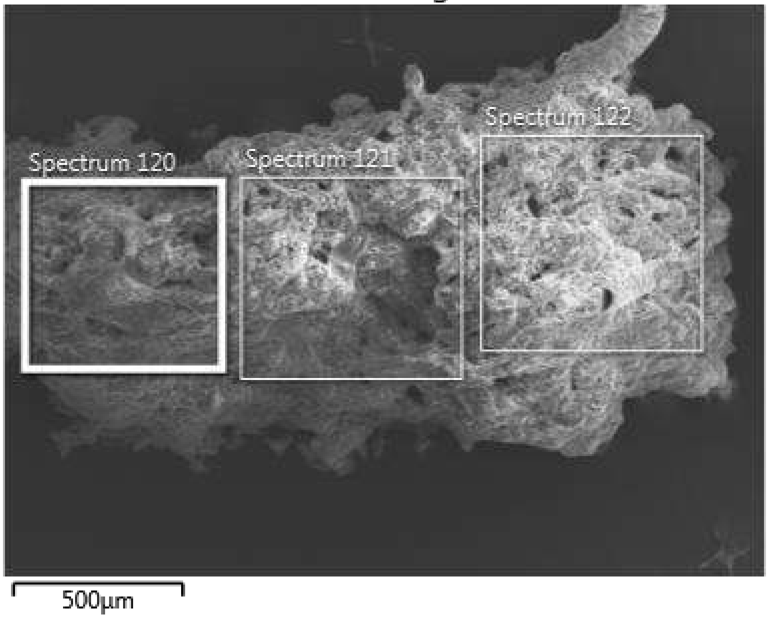

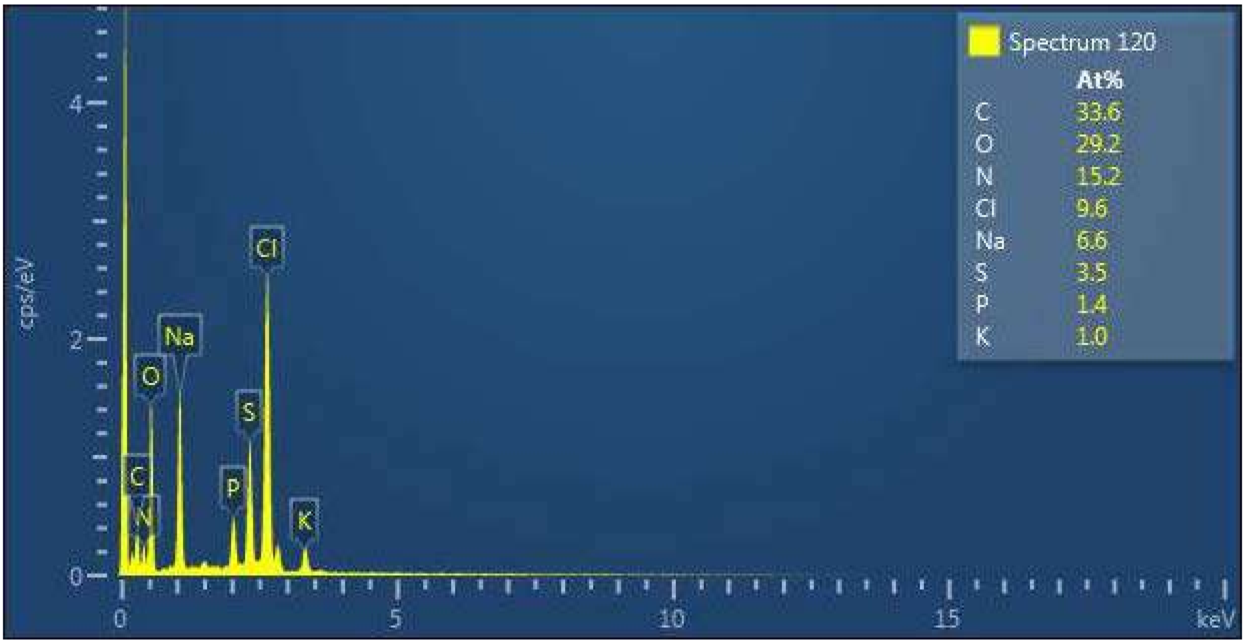

Label:	Spectrum 120
Source:	Acquired
Created:	29/11/2019 12:42:16
Livetime:	60.5s
Process Time:	4
Accelerating Voltage:	15.00kV
Magnification:	50 x
Working Distance:	9.8mm
Specimen Tilt (degrees):	0.0
Elevation (degrees):	35.0
Azimuth (degrees):	0.0
Number Of Channels:	2048
Energy Range (keV):	20 keV
Energy per Channel (eV):	10.0eV
Detector Type Id:	29
Detector Type:	X-Max
Window Type:	SATW
Pulse Pile Up Correction:	Succeeded
Primary Detector:	2617
Primary Detector Serial Number:	77871-X080

Element	Line Type	Apparent Concentration	k Ratio	Wt%	Wt% Sigma	Standard Label	Factory Standard	Standard Calibration Date
C	K series	0.69	0.00686	22.83	1.65	C Vit	Yes	
N	K series	2.33	0.00414	12.03	1.04	BN	Yes	
O	K series	4.18	0.01408	26.42	0.77	SiO2	Yes	
Na	K	2.63	0.01110	8.58	0.27	Albite	Yes	

	series							
P	K series	0.90	0.00504	2.42	0.12	GaP	Yes	
S	K series	1.63	0.01406	6.41	0.21	FeS2	Yes	
Cl	K series	4.50	0.03934	19.21	0.52	NaCl	Yes	
K	K series	0.50	0.00422	2.11	0.12	KBr	Yes	
Total:				100.00				

Electron Image 43

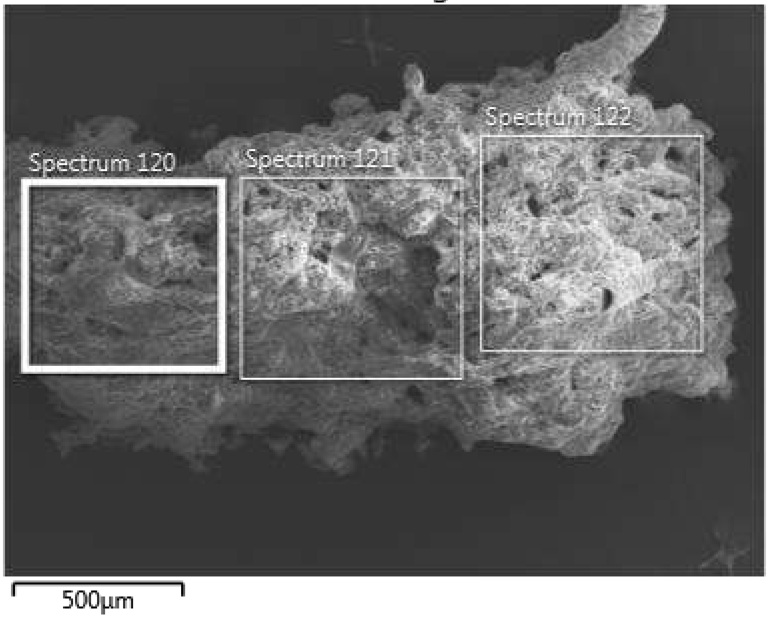

29/11/2019 12:43:23
Project 1
Glandulas salivares Mexico T3
GSM2 15 Kv

Electron Image 43

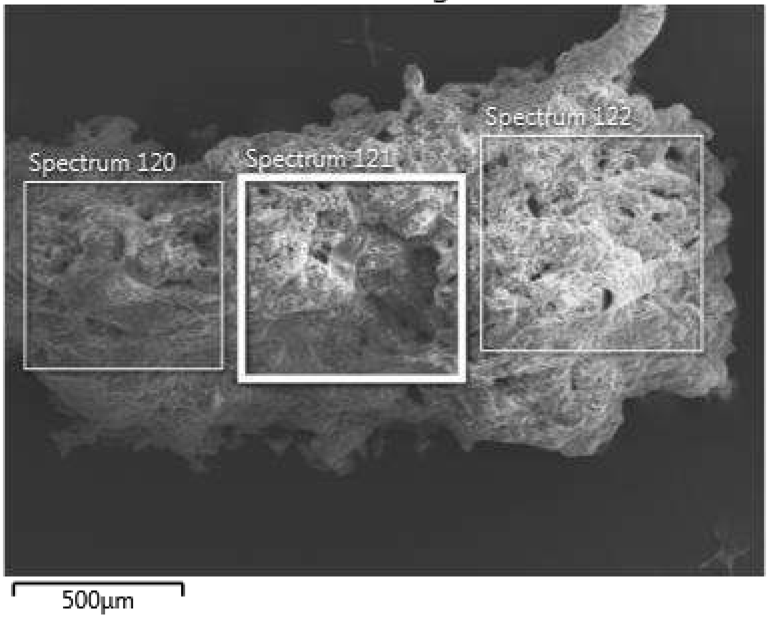

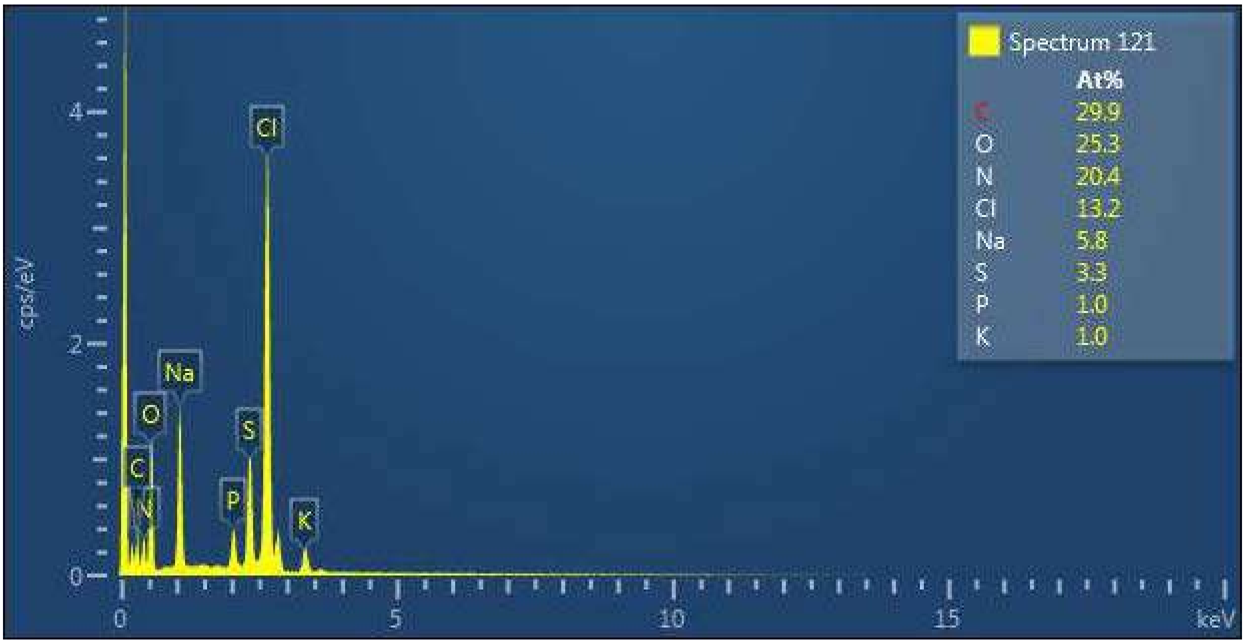

Label:	Spectrum 121
Source:	Acquired
Created:	29/11/2019 12:43:23
Livetime:	60.4s
Process Time:	4
Accelerating Voltage:	15.00kV
Magnification:	50 x
Working Distance:	9.8mm
Specimen Tilt (degrees):	0.0
Elevation (degrees):	35.0
Azimuth (degrees):	0.0
Number Of Channels:	2048
Energy Range (keV):	20 keV
Energy per Channel (eV):	10.0eV
Detector Type Id:	29
Detector Type:	X-Max
Window Type:	SATW
Pulse Pile Up Correction:	Succeeded
Primary Detector:	2617
Primary Detector Serial Number:	77871-X080

Element	Line Type	Apparent Concentration	k Ratio	Wt%	Wt% Sigma	Standard Label	Factory Standard	Standard Calibration Date
C	K series	0.53	0.00530	19.62	1.77	C Vit	Yes	
N	K series	3.05	0.00542	15.61	1.08	BN	Yes	
O	K series	3.32	0.01118	22.15	0.71	SiO2	Yes	
Na	K	2.35	0.00993	7.33	0.24	Albite	Yes	

	series							
P	K series	0.69	0.00386	1.76	0.10	GaP	Yes	
S	K series	1.56	0.01342	5.76	0.20	FeS2	Yes	
Cl	K series	6.34	0.05539	25.59	0.69	NaCl	Yes	
K	K series	0.53	0.00453	2.19	0.12	KBr	Yes	
Total:				100.00				

Electron Image 43

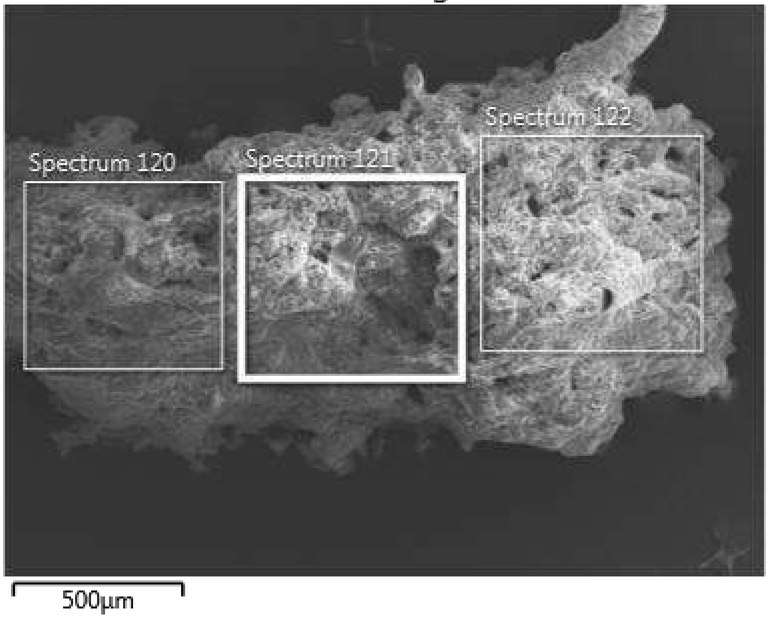

29/11/2019 12:44:30
Project 1
Glandulas salivares Mexico T3
GSM2 15 Kv

Electron Image 43

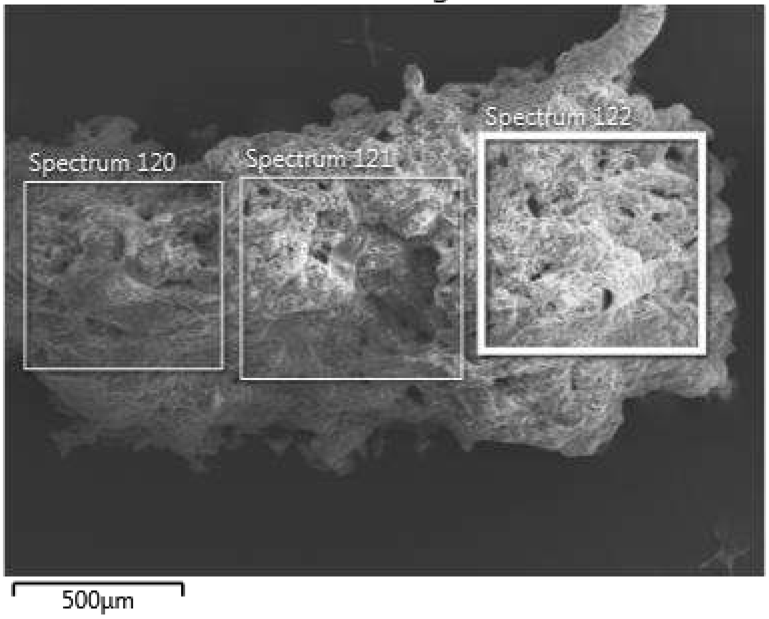

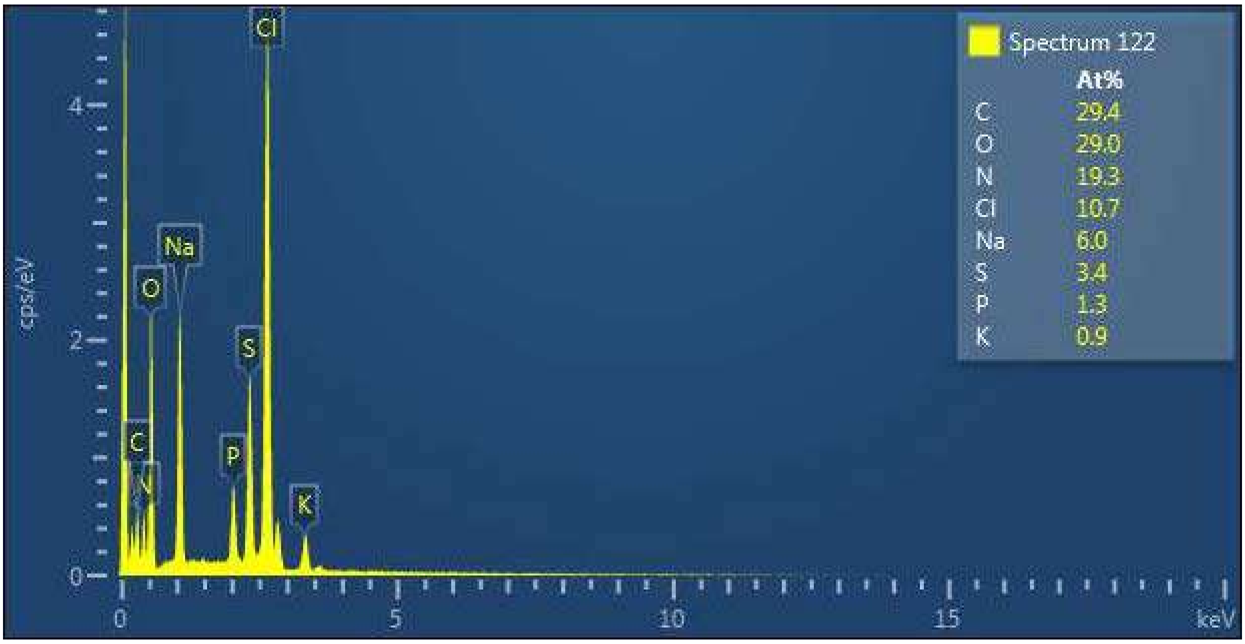

Label:	Spectrum 122
Source:	Acquired
Created:	29/11/2019 12:44:30
Livetime:	60.3s
Process Time:	4
Accelerating Voltage:	15.00kV
Magnification:	50 x
Working Distance:	9.8mm
Specimen Tilt (degrees):	0.0
Elevation (degrees):	35.0
Azimuth (degrees):	0.0
Number Of Channels:	2048
Energy Range (keV):	20 keV
Energy per Channel (eV):	10.0eV
Detector Type Id:	29
Detector Type:	X-Max
Window Type:	SATW
Pulse Pile Up Correction:	Succeeded
Primary Detector:	2617
Primary Detector Serial Number:	77871-X080

Element	Line Type	Apparent Concentration	k Ratio	Wt%	Wt% Sigma	Standard Label	Factory Standard	Standard Calibration Date
C	K series	0.92	0.00917	19.71	1.31	C Vit	Yes	
N	K series	4.90	0.00872	15.11	0.82	BN	Yes	
O	K series	6.42	0.02161	25.96	0.60	SiO2	Yes	
Na	K	3.77	0.01592	7.66	0.19	Albite	Yes	

	series							
P	K series	1.38	0.00774	2.29	0.09	GaP	Yes	
S	K series	2.49	0.02144	6.00	0.16	FeS2	Yes	
Cl	K series	8.10	0.07081	21.24	0.44	NaCl	Yes	
K	K series	0.78	0.00659	2.04	0.09	KBr	Yes	
Total:				100.00				

Electron Image 43

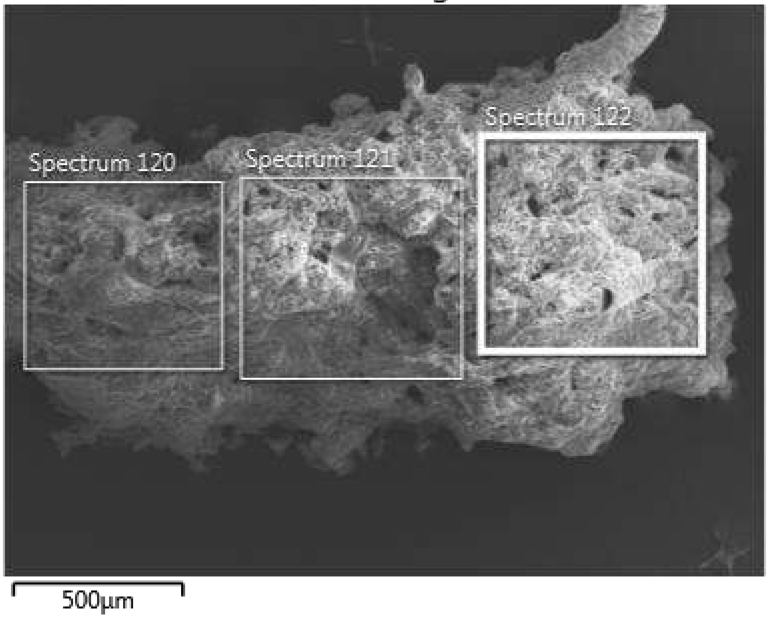

29/11/2019 12:46:40
Project 1
Glandulas salivares Mexico T3
GSM3 15 Kv

Electron Image 44

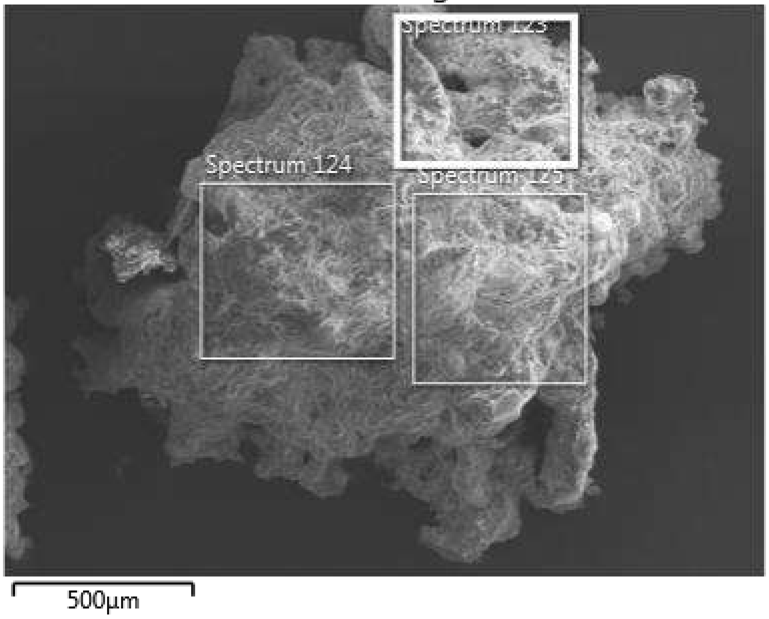

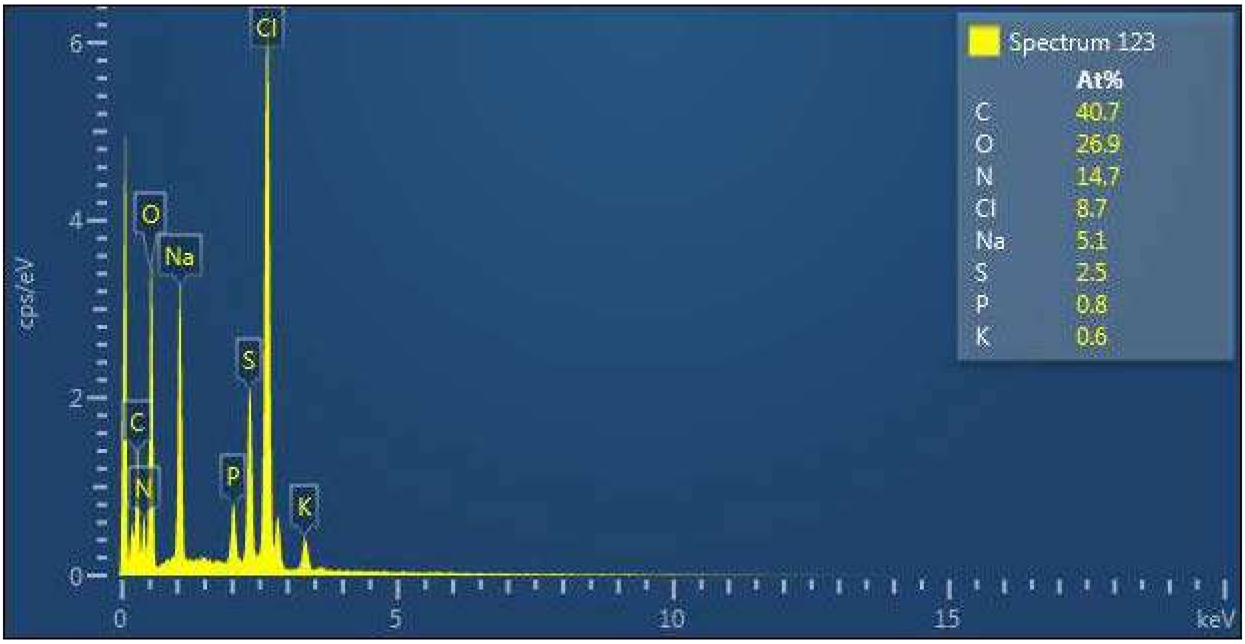

Label:	Spectrum 123
Source:	Acquired
Created:	29/11/2019 12:46:40
Livetime:	60.5s
Process Time:	4
Accelerating Voltage:	15.00kV
Magnification:	53 x
Working Distance:	9.8mm
Specimen Tilt (degrees):	0.0
Elevation (degrees):	35.0
Azimuth (degrees):	0.0
Number Of Channels:	2048
Energy Range (keV):	20 keV
Energy per Channel (eV):	10.0eV
Detector Type Id:	29
Detector Type:	X-Max
Window Type:	SATW
Pulse Pile Up Correction:	Succeeded
Primary Detector:	2617
Primary Detector Serial Number:	77871-X080

Element	Line Type	Apparent Concentration	k Ratio	Wt%	Wt% Sigma	Standard Label	Factory Standard	Standard Calibration Date
C	K series	2.53	0.02531	29.11	0.94	C Vit	Yes	
N	K series	5.85	0.01042	12.24	0.68	BN	Yes	
O	K series	10.11	0.03402	25.67	0.47	SiO2	Yes	
Na	K	5.44	0.02296	6.95	0.14	Albite	Yes	

	series							
P	K series	1.39	0.00777	1.45	0.06	GaP	Yes	
S	K series	3.13	0.02695	4.76	0.11	FeS2	Yes	
Cl	K series	11.15	0.09744	18.33	0.31	NaCl	Yes	
K	K series	0.91	0.00768	1.48	0.07	KBr	Yes	
Total:				100.00				

Electron Image 44

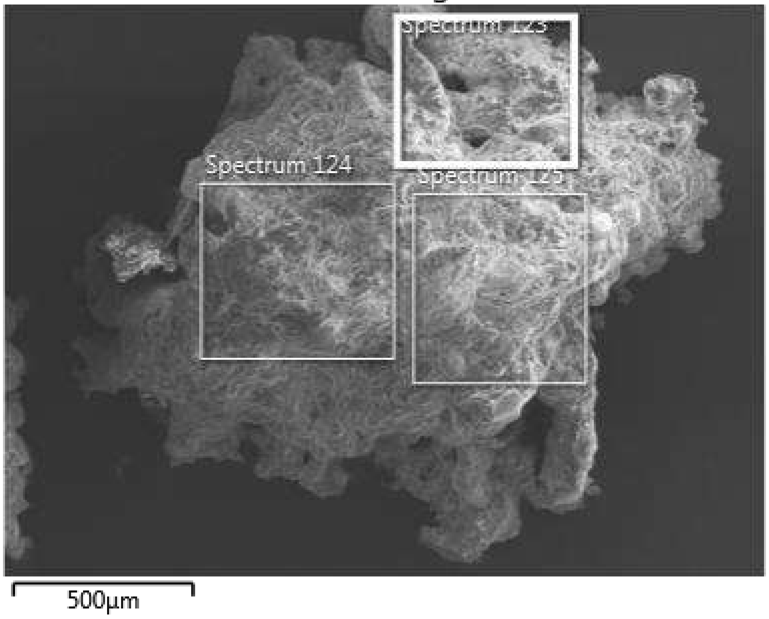

29/11/2019 12:47:49
Project 1
Glandulas salivares Mexico T3
GSM3 15 Kv

Electron Image 44

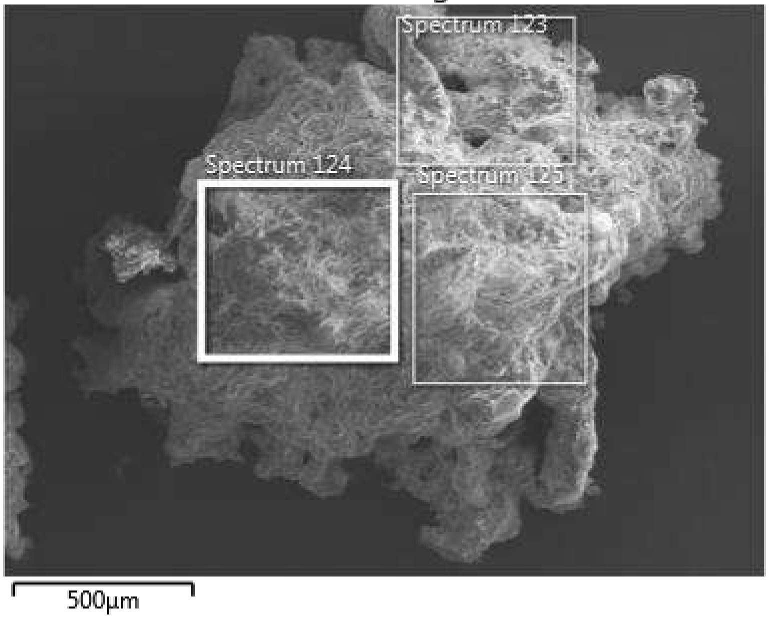

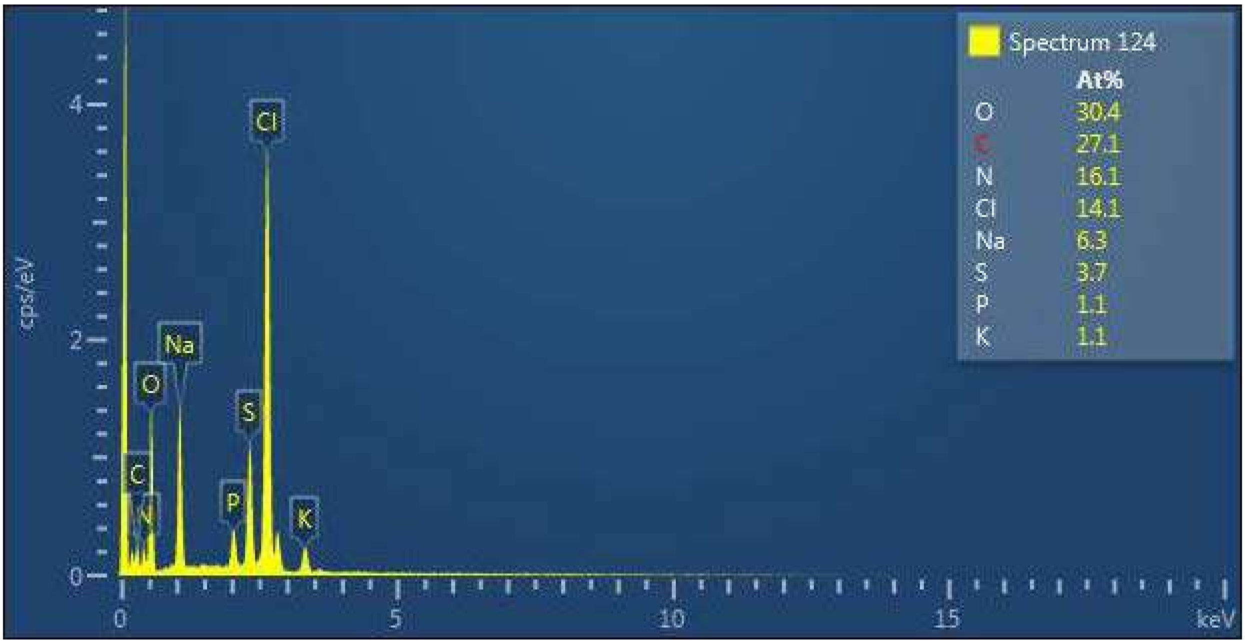

Label:	Spectrum 124
Source:	Acquired
Created:	29/11/2019 12:47:49
Livetime:	60.3s
Process Time:	4
Accelerating Voltage:	15.00kV
Magnification:	53 x
Working Distance:	9.8mm
Specimen Tilt (degrees):	0.0
Elevation (degrees):	35.0
Azimuth (degrees):	0.0
Number Of Channels:	2048
Energy Range (keV):	20 keV
Energy per Channel (eV):	10.0eV
Detector Type Id:	29
Detector Type:	X-Max
Window Type:	SATW
Pulse Pile Up Correction:	Succeeded
Primary Detector:	2617
Primary Detector Serial Number:	77871-X080

Element	Line Type	Apparent Concentration	k Ratio	Wt%	Wt% Sigma	Standard Label	Factory Standard	Standard Calibration Date
C	K series	0.43	0.00427	17.28	1.82	C Vit	Yes	
N	K series	2.27	0.00404	12.01	1.04	BN	Yes	
O	K series	4.01	0.01349	25.89	0.78	SiO2	Yes	
Na	K	2.37	0.01002	7.75	0.25	Albite	Yes	

	series							
P	K series	0.70	0.00392	1.85	0.11	GaP	Yes	
S	K series	1.65	0.01420	6.34	0.22	FeS2	Yes	
Cl	K series	6.31	0.05512	26.57	0.71	NaCl	Yes	
K	K series	0.54	0.00457	2.31	0.13	KBr	Yes	
Total:				100.00				

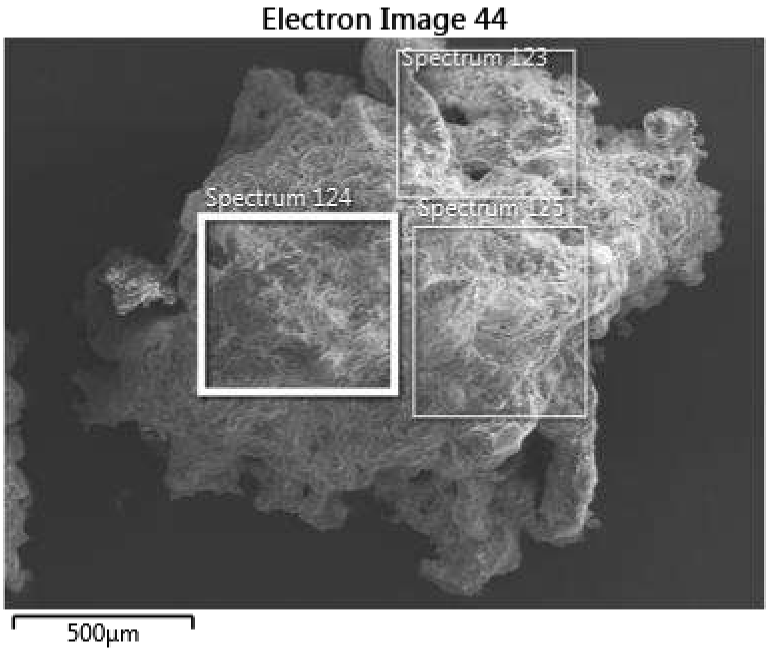

29/11/2019 12:48:56
Project 1
Glandulas salivares Mexico T3
GSM3 15 Kv

Electron Image 44

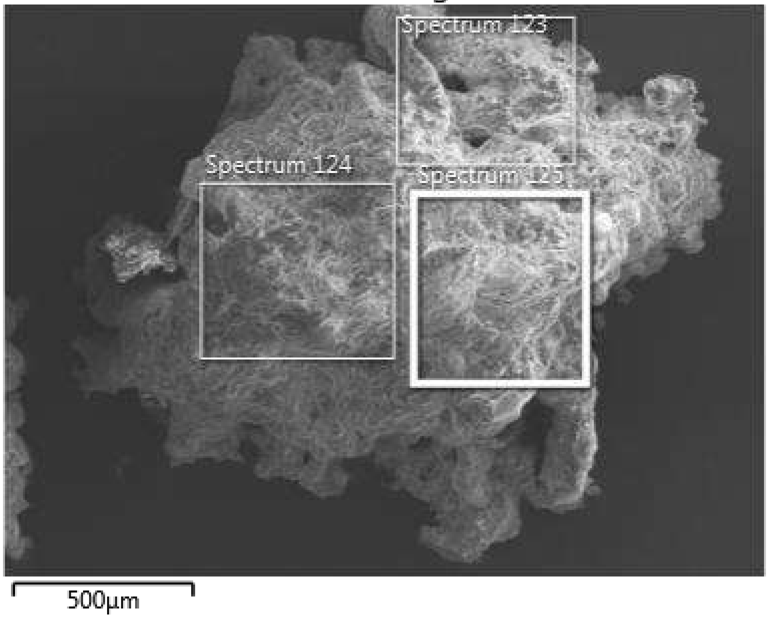

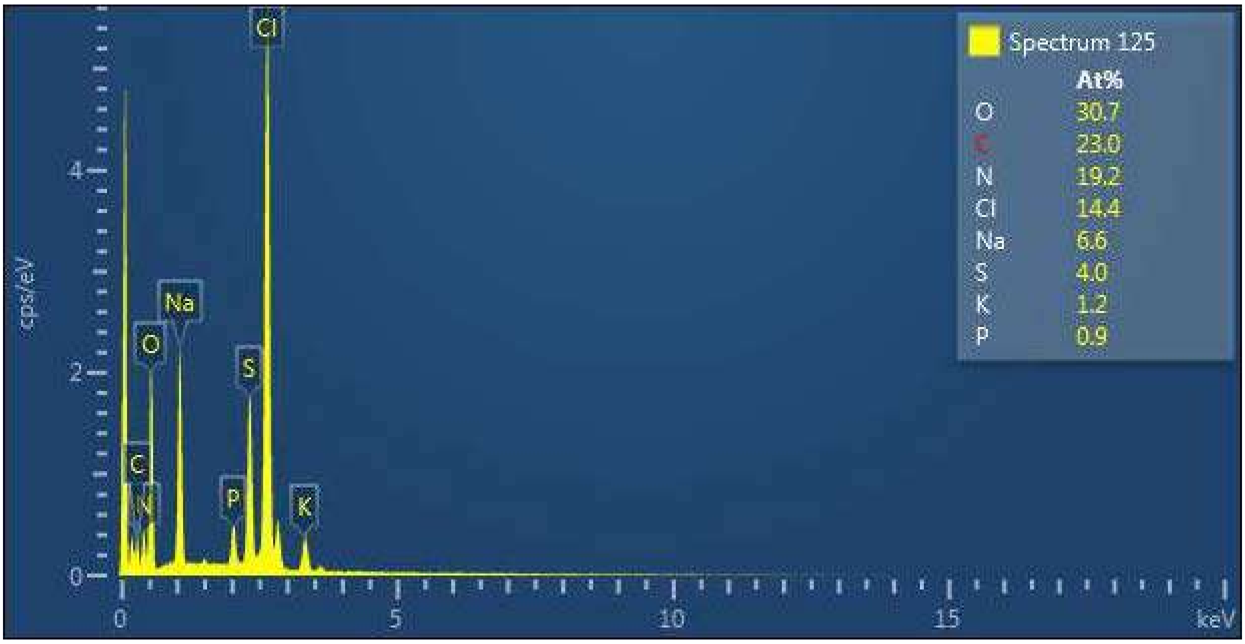

Label:	Spectrum 125
Source:	Acquired
Created:	29/11/2019 12:48:56
Livetime:	60.1s
Process Time:	4
Accelerating Voltage:	15.00kV
Magnification:	53 x
Working Distance:	9.8mm
Specimen Tilt (degrees):	0.0
Elevation (degrees):	35.0
Azimuth (degrees):	0.0
Number Of Channels:	2048
Energy Range (keV):	20 keV
Energy per Channel (eV):	10.0eV
Detector Type Id:	29
Detector Type:	X-Max
Window Type:	SATW
Pulse Pile Up Correction:	Succeeded
Primary Detector:	2617
Primary Detector Serial Number:	77871-X080

Element	Line Type	Apparent Concentration	k Ratio	Wt%	Wt% Sigma	Standard Label	Factory Standard	Standard Calibration Date
C	K series	0.54	0.00536	14.53	1.50	C Vit	Yes	
N	K series	4.21	0.00749	14.18	0.85	BN	Yes	
O	K series	5.98	0.02011	25.88	0.63	SiO2	Yes	
Na	K	3.66	0.01546	7.96	0.21	Albite	Yes	

	series							
P	K series	0.85	0.00474	1.49	0.08	GaP	Yes	
S	K series	2.62	0.02258	6.67	0.18	FeS2	Yes	
Cl	K series	9.59	0.08383	26.79	0.58	NaCl	Yes	
K	K series	0.88	0.00745	2.49	0.11	KBr	Yes	
Total:				100.00				

Electron Image 44

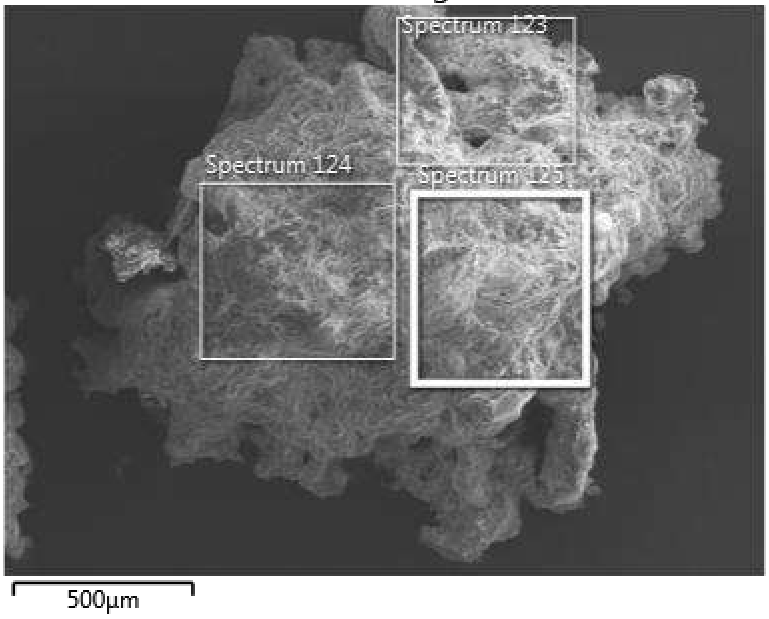

Supplement: Supplementary file 1 [file biomolecules-10-00555-s001.zip › Data S5.pdf]
